# Regioselective Synthesis of 9‑*O*‑Arylboldine Derivatives Using the Copper-Catalyzed Chan–Lam Reaction†

**DOI:** 10.1021/acsomega.5c09458

**Published:** 2026-06-09

**Authors:** Cristian Suárez-Rozas, Daniel A. A. Araya-Santelices, Oriel A. Sánchez-Velasco, Cristóbal Hormazábal-Campos, JeanLuc Bertrand, Petra Krňávková, Veronika Vojáčková, Vladimir Kryštof, Bruce K. Cassels, Edwin G. Pérez

**Affiliations:** † Department of Organic Chemistry, Faculty of Chemistry and Pharmacy, 28033 Pontificia Universidad Católica de Chile, Santiago 7820436, Chile; ‡ Centro de Química Médica, Facultad de Medicina Clínica Alemana, 14655 Universidad del Desarrollo, Santiago 7610315, Chile; § Department of Experimental Biology, Faculty of Science, 48207 Palacký University, Šlechtitelů 27, 77900 Olomouc, Czech Republic; ∥ Department of Chemistry, Faculty of Sciences, University of Chile, Santiago 7800003, Chile

## Abstract

While alkylated and acylated aporphine derivatives are well-represented in the literature, *O*-arylated analogues remain scarce. We report here a practical and regioselective methodology for the synthesis of 9-*O*-arylboldine derivatives. Our approach utilizes a copper-catalyzed Chan–Lam coupling reaction, enabling the efficient preparation of 30 novel 9-*O*-arylboldine derivatives. This operationally simple procedure proceeds at room temperature under ambient air conditions, employing readily available phenylboronic acids as coupling partners. Extensive one- and two-dimensional NMR studies unequivocally confirmed the desired 9-*O*-regioselectivity. Further demonstrating the scope of this method, we synthesized five new 3-bromo-9-*O*-arylboldine derivatives starting from 3-bromoboldine. Additionally, some 9-*O*-phenylboldine derivatives served as versatile intermediates, undergoing transformations to access *N*,*N*-dimethylammonium derivatives and enabling the conversion of 4′-formylphenyl derivative **6w** into four aminoaporphine derivatives via reductive amination. This study confirms the usefulness of the Chan–Lam cross-coupling reaction as a powerful and flexible synthetic tool for the derivatization of natural products, particularly with the goal of expanding the chemical diversity of aporphine alkaloids.

## Graphical abstract

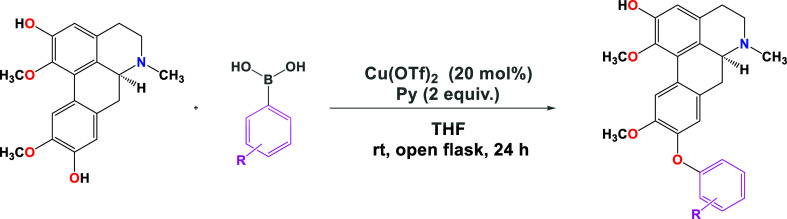

## Introduction

*S*-(+)-Boldine (**1**) is the predominant alkaloid in the bark of *Peumus boldus* Molina (‘boldo’ in the vernacular and internationally),^1^ and during the last decades, it has received significant attention as a result of its wide range of pharmacological activities, including α_1A_-adrenergic antagonism in vascular tissue,^2^ nonselective (D_1_- and D_2_-like) dopaminergic antagonism,^3^ hepato- and gastro-protective effects, anti-inflammatory and antipyretic activity, and antiproliferative effect, among others.^4,5^

Not surprisingly, a significant number of studies have appeared, associated with its use as a starting material for the preparation of related alkaloids and unnatural analogues,^6–10^ obtaining more potent semisynthetic derivatives,^11,12^ preserving its antioxidant character, and coupling it to a cytotoxic moiety,^13,14^ or to generate novel heterocyclic systems.^15,16^ In this regard, *O*-functionalization methods of boldine have limitations regarding their regioselectivity due to competition of the C-2 and C-9 hydroxyl groups. In the case of alkylation reactions (diazomethane treatment and Mitsunobu conditions),^7,10^ isomeric mixtures of mono-*O*-alkyl products predicentrine (**2**), *N*-methyl-laurotetanine (**3**), and di-*O*-alkylated glaucine (**4**) are formed (Figure 1). In addition, under Williamson conditions, the quaternization of the tertiary nitrogen and subsequent cleavage of ring B to afford a phenanthrene ring system is the outcome.^17^ In spite of this lack or selectivity, the phenol group at C-9 appeared to be more reactive,^7,10^ which could be explained in part by the differences in the acidity of the C-2 and C-9 hydroxyl groups (p*K*_a_ 10.44 and 9.16, respectively), as expected due to the electron delocalization from the phenol group at C-9 over the whole biphenyl system, which is not possible for the C-2 OH group.^18^

**Figure 1:**
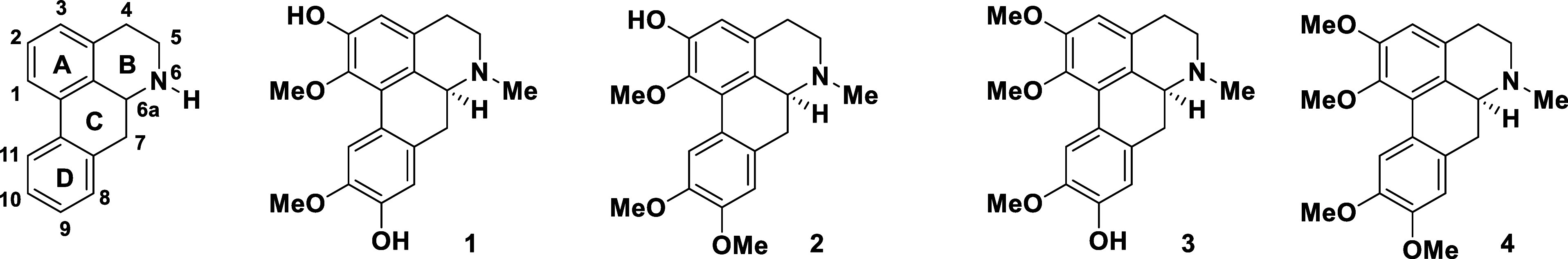
Lettering and numbering of the aporphine scaffold, and structures of boldine (**1**), predicentrine (**2**), *N*-methyl-laurotetanine (**3**), and glaucine (**4**).

In past decades with the development of synthetic methodology to enable the regioselective preparation of a wide range of derivatives of aporphines, including their *O-*alkyl structural modification, to extend the chemical diversity in this class of compounds subjecting the products to biological testing has led to interesting structure–activity relationships (SAR).^10,19–27^ However, these reactions, though often alkylating the phenol groups, have never aimed at the formation of diaryl ether moieties. To the best of our knowledge, there is a single, nearly 50-year-old report of *O*-arylation of boldine under Ullmann conditions (stoichiometric amount of CuO, excess of Py and bromobenzene) by Shamma and co-workers, resulting in a <5% yield of 9-*O*-phenylboldine, although the substitution at the C-9 phenol group was not demonstrated unequivocally.^28^

The Chan–Lam coupling reaction is a copper-mediated oxidative cross-coupling of amines, phenols, and other heteroatom nucleophiles with arylboron species, enabling the formation of C–N and C–O bonds under comparatively mild conditions. In contrast to classical Ullmann–Goldberg reactions and Pd-catalyzed Buchwald–Hartwig etherifications, which generally rely on aryl halides or pseudohalides and often require elevated temperatures, strong base, and careful ligand/catalyst optimization, Chan–Lam couplings can frequently be performed in the presence of air and at or near room temperature. Modern Buchwald–Hartwig etherification has undoubtedly become a powerful method for aryl ether synthesis, including for sterically demanding phenols when appropriate bulky electron-rich phosphine ligands are used; however, its application to highly functionalized and sterically congested natural-product scaffolds may be limited by the need for prefunctionalized electrophilic partners and by the sensitivity of complex substrates to basic or thermal conditions. In the present case, boldine is a hindered, multifunctional phenolic alkaloid, and the only previously reported *O*-arylation of boldine, performed under Ullmann-type conditions, proceeded in very low yield. Therefore, Chan–Lam coupling was selected as a complementary and operationally simple approach because it uses readily available arylboronic acids, tolerates halogen substituents and other functional groups, and employs inexpensive, relatively nontoxic copper salts under milder oxidative conditions. These features make Chan–Lam coupling particularly attractive for the late-stage diversification of highly functionalized natural products such as boldine.^29–36^ Nevertheless, despite these advantages and the continuing demand for increased chemical diversity in the search for compounds with improved biological activities, the application of Chan–Lam coupling to natural-product modification remains comparatively underexplored.^37–39^

In this paper, we report a novel methodology for the regioselective synthesis of 9-*O*-arylboldine derivatives. Seeking potential uses for the newly synthesized compounds, preliminary cytotoxicity assays were performed on the MV4–11 cancer cell line. Boldine was practically inactive in these cells, but all of its 9-*O*-aryl derivatives exhibited significant activity.

## Results and Discussion

Boldine (**1**) was isolated from *P. boldus* bark as a 1:1 complex with chloroform^12^ and was used as such. Optimization of the regioselective Chan–Lam C–O cross-coupling was conducted at room temperature employing boldine (**1**-CHCl_3_), various bases, and phenylboronic acid (**5a**) in an open vessel for 24 h. The representative results from this study are summarized in Table 1. The initial attempt was carried out using 10 mol % Cu­(OAc)_2_ as a catalyst, triethylamine (Et_3_N) as a base, tetrahydrofuran (THF) as a solvent, and 3 Å molecular sieves as an additive at room temperature for 24 h. Under these conditions, the arylated boldine (**6a**) was obtained in 30% yield (Table 1, entry 1). The identity of this product as 9-*O*-phenylboldine was confirmed by NMR and HR-MS techniques (vide infra). No reaction occurred in the absence of Cu­(OAc)_2_ (Table 1, entry 2). Other solvents, including dichloromethane (DCM), 1,4-dioxane, and *N*,*N*-dimethylformamide (DMF) were surveyed, but none gave a better yield of **6a** than that obtained in THF (Table 1, entries 3–5). Different copper salts were tried but only Cu­(OTf)_2_ gave better results (36% yield) than Cu­(OAc)_2_ (Table 1, entries 6–10). Then, using Cu­(OTf)_2_ as the catalyst, different bases were tried, including DIPEA, DMAP, and DBU, but were found to be less effective (Table 1, entries 11–13). Surprisingly, the addition of pyridine (Py) as the base led to the desired product **6a** in 56% yield (Table 1, entry 14). Eliminating the use of molecular sieves and increasing the amount of phenylboronic acid to 2 equiv, the yield increased slightly to 62 and 63%, respectively (Table 1, entries 15 and 16). This result suggests that the presence of moisture is not a significant factor, and the absence of solids in the reaction mixture substantially improves both effective stirring and the oxidation step in the catalytic cycle. Finally, the best conditions were found using 1 mmol of **1**-CHCl_3_ and 2 mmol of phenylboronic acid in tetrahydrofuran (6.0 mL) with Cu­(OTf)_2_ (0.2 mmol) and 2 mmol of pyridine, followed by stirring at room temperature for 24 h under air (open flask) to give 76% yield (Table 1, entry 17). Although under these conditions, the yield obtained was 76%, the amount of self-coupling of **5a** observed by thin-layer chromatography (TLC) also increased. Additionally, it is important to point out that 2-*O*-arylation was not observed during the optimization of the reaction.

**Table 1:**
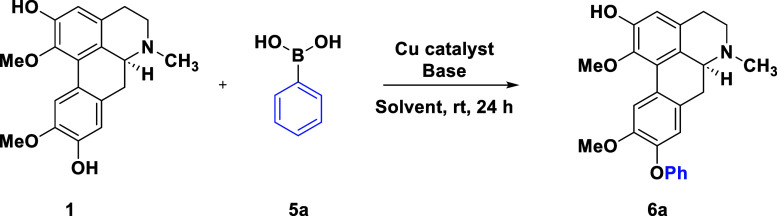
Optimization of the Chan–Lam Regioselective *O*-Arylation of the C-9 Hydroxyl Group of Boldine (**1**).^a^

entry	ratio (1:5a)	catalyst (mol %)	base	solvent	additive	isolated yield (%)^b^
1	1:1	Cu(OAc)_2_ (10)	Et_3_N	THF	MS, 3 Å	30
2	1:1	-------------	Et_3_N	THF	MS, 3 Å	NR^c^
3	1:1	Cu(OAc)_2_ (10)	Et_3_N	CH_2_Cl_2_	MS, 3 Å	trace^d^
4	1:1	Cu(OAc)_2_ (10)	Et_3_N	1,4-dioxane	MS, 3 Å	26
5	1:1	Cu(OAc)_2_ (10)	Et_3_N	DMF	MS, 3 Å	22
6	1:1	Cu(BF_4_)_2_·H_2_O (10)	Et_3_N	THF	MS, 3 Å	11
7	1:1	CuOAc (10)	Et_3_N	THF	MS, 3 Å	NR^c^
8	1:1	CuCl (10)	Et_3_N	THF	MS, 3 Å	NR^c^
9	1:1	CuCl_2_ (10)	Et_3_N	THF	MS, 3 Å	trace^d^
10	1:1	Cu(OTf)_2_ (10)	Et_3_N	THF	MS, 3 Å	36
11	1:1	Cu(OTf)_2_ (10)	DIPEA	THF	MS, 3 Å	16
12	1:1	Cu(OTf)_2_ (10)	DMAP	THF	MS, 3 Å	16
13	1:1	Cu(OTf)_2_ (10)	DBU	THF	MS, 3 Å	trace^d^
14	1:1	Cu(OTf)_2_ (10)	Py	THF	MS, 3 Å	56
15	1:1	Cu(OTf)_2_ (10)	Py	THF	-------	63
16	1:2	Cu(OTf)_2_ (10)	Py	THF	-------	62
17	1:2	Cu(OTf)_2_ (20)	Py	THF	-------	76

a Reactions were carried out with boldine (**1**-CHCl_3_, 1.0 mmol) in 6.0 mL of solvent, PhB­(OH)_2_ **5a** (1 or 2 mmol), catalyst (10 or 20 mol·%), and base (2 mmol) in an open flask at room temperature for 24 h.

b After column chromatographic purification.

c No reaction was observed.

d Determined by TLC.

The mono-*O*-arylation of boldine was demonstrated by the HR-MS, ^1^H, and ^13^C NMR spectra of **6a**. The **6a** HR-MS measurements gave the *m*/*z* of the parent ion [M + H]^+^ as 404.1868, corresponding to the molecular formula C_25_H_25_NO_4_ (calcd for C_25_H_25_NO_4_+H^+^: 404.1857), i.e., boldine (C_19_H_21_NO_4_) with an additional C_6_H_5_ moiety replacing a H atom. Relative to the boldine extracted for this study (Figures S1–S3), the ^1^H NMR spectrum of product **6a** presented three new signals: a doublet of doublets at 7.34 ppm (2H, *J* = 7.33, 8.55), a triplet at 7.10 ppm (1H, *J* = 7.41), and a multiplet at 7.04 ppm (2H), totaling five new protons. These protons correlated with four new signals in the ^13^C NMR spectrum (157.5, 129.7, 123.1, 118.1 ppm), confirming the incorporation of a single benzene ring.

Subsequently, the methylation of the remaining –OH group in **6a** with trimethylanilinium chloride to obtain **7a** (Scheme 1)^9^ was also confirmed by the presence of a new singlet (in **7a**) at 3.89 ppm (3H) in the ^1^H NMR spectrum and an additional signal at 55.9 ppm in the ^13^C NMR spectra, corresponding to the new methoxy group.

**Scheme 1:**
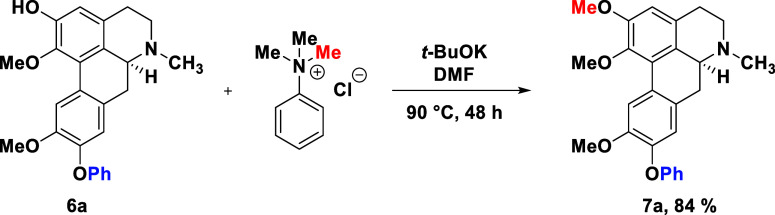
*O*-Methylation of **6a** [Fn s1fn1]

a
**Reaction conditions**: **6a** (2 mmol), *N*,*N*,*N*-trimethylanilinium chloride (7 mmol), *t*-BuOK (7 mmol), and DMF (7.3 mL) under N_2_, 48 h.

To determine whether *O*-methylation and *O*-arylation took place, respectively, at the O-2 and O-9 positions in boldine or in the opposite order, a NOESY experiment was used. Here, the most relevant NOESY correlations in **7a** were detected for H-3 and H-8 (Figure 2). Two significant interactions from H-3 (6.62 ppm) were identified: one cross-peak with the MeO signal at 3.89 ppm from C-2 and another with the H-4 signal (2.68 ppm). Additionally, H-8 only showed a cross-peak with H-7, indicating no methylation of the –OH group at C-9. Thus, the suggested *O*-arylation at C-9 and *O*-methylation at C-2 in compound **7a** are supported by the NOESY correlations. Furthermore, the interaction of H-11 with the methoxy group at C-10 remained unchanged. Our NMR analyses and assignments (^1^H, ^13^C, NOESY) agree well with the detailed 2D NMR studies on glaucine (**4**) and *N*-methyl-laurotetanine (**3**).^10,40^ Figure 2.

**Figure 2:**
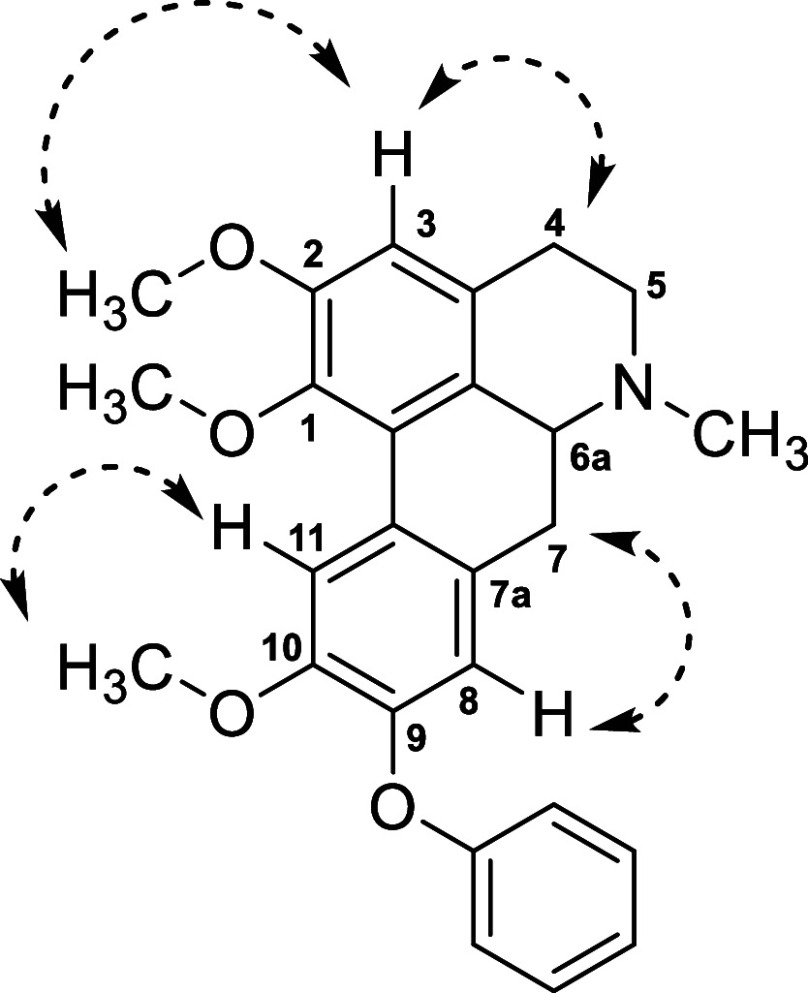
Most relevant NOESY correlations of compound **7a**.

With the optimized conditions in hand, 30 different new 9-*O*-arylboldine derivatives (**6a–6ad**) were synthesized on a 1 mmol scale or larger using a variety of *ortho-*, *meta-*, and *para-*substituted phenylboronic acids, in yields ranging from 35 to 80% (Scheme 2).

**Scheme 2:**
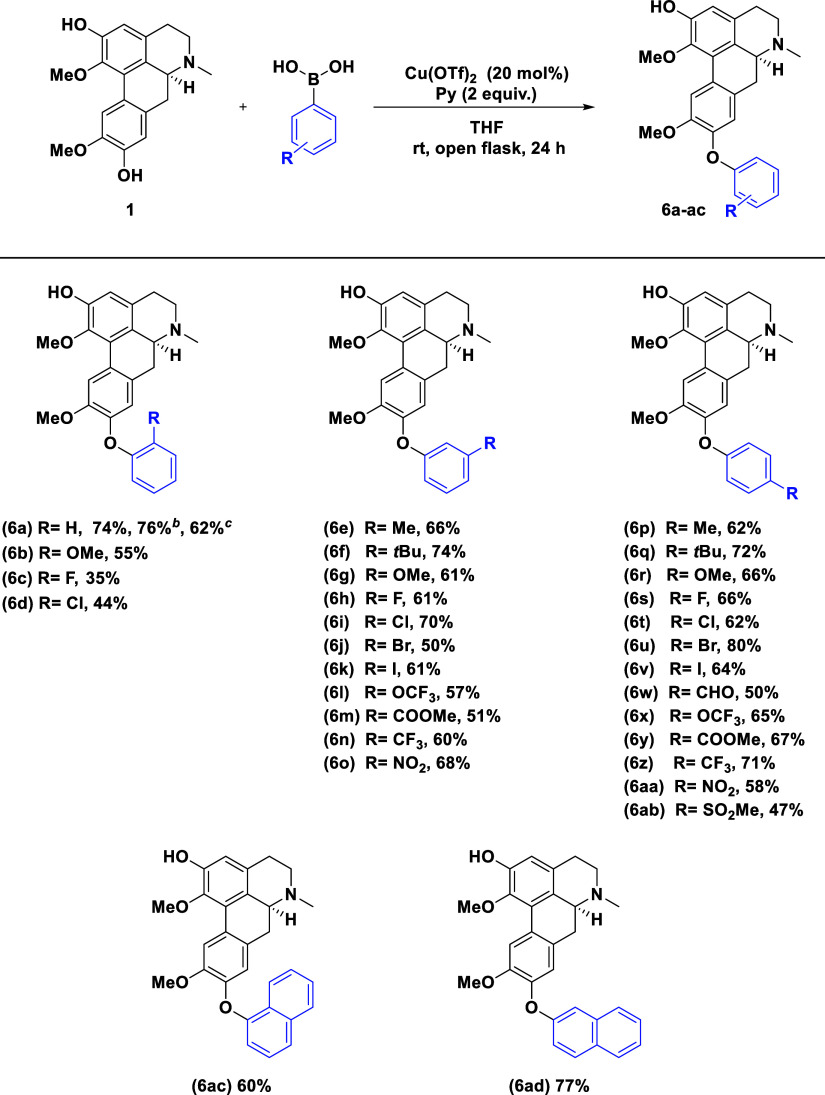
Scope of Regioselective 9-*O*-Arylation of Boldine[Fn s2fn1]–^c^

a
**Reaction conditions**: boldine (**1**-CHCl_3_, 1.0 mmol), THF (6.0 mL), phenylboronic acid (2 mmol), Cu­(OTf)_2_ (20 mol·%), and pyridine (2 mmol) in an open flask at room temperature for 24 h. Isolated yield after column chromatography.
b At a 5 mmol scale.
c At a 10 mmol scale.

We found that a wide range of functionalized groups, such as alkyl, halogen, alkoxy, ester, nitro, 1- and 2-naphthyl, and methyl mesylate, were tolerated in this methodology. Regarding the *ortho*-substituted products prepared (**6b**–**d**), yields tended to decrease in response to the increasing size and electronegativity of the substituent. Diphenyl ether **6c** (35%) was obtained in the lowest yield among all the substitution patterns tested. We next turned our attention to the analysis of those reaction products that presented methoxy (**6b**, **6g**, and **6r**), fluoro (**6c**, **6h**, and **6s**), or chloro (**6d**, **6i**, and **6t**) groups at all three positions of the phenyl ring. Our findings confirmed that substitution in the *ortho* position is the least favorable, due to its proximity to the coupling site, resulting in lower reaction yields for these compounds.^38,41^ In general, it was seen that methoxy- and fluoro-substituted derivatives in the *para* position (**6r** and **6s**, respectively) provided the best yields (66%, in both cases), presumably by minimizing steric effects and presenting favorable electronic effects in the boronic acid substrates.^42^ The reaction proceeded smoothly with all the halo-phenylboronic acids (with the exception of **6c**), giving a particularly high yield of 9-*O*-(4-bromophenyl)­boldine (**6u**), i.e., 80% yield. These results indicate that the electronic effects (inductive and resonance) of the halogen in the reaction are not clearly correlated for this particular type of substituent. When phenylboronic acids with weakly electron-donating substituents, i.e., *meta*- and *para*-methyl (**6e** and **6p**, respectively) and *meta*- and *para*-*tert*-butyl (**6f** and **6q**, respectively) were utilized, yields ranging from 62 to 74% were obtained. Regardless of their position, the highest yields were obtained for the more sterically demanding substituent. On the other hand, electron-withdrawing substituents, i.e., formyl, trifluoromethoxy, methoxycarbonyl, trifluoromethyl, nitro, and mesyl, were also tolerated, providing the desired products in yields of 47%–71%. While several *para*-substituted derivatives showed higher yields than their *meta*-counterparts, notable exceptions were observed for the methyl (**6e** vs **6p**), *tert*-butyl (**6f** vs **6q**), chloro (**6i** vs **6t**), and nitro (**6o** vs **6aa**) series. Regarding mesyl substitution, only the *para*-substituted boronic acid was used, affording **6ab** with a moderate yield of 47%. The scope of this methodology was extended to bicyclic aromatic systems and found to be compatible with both naphthylboronic acid isomers, which produced **6ac** (α-derived) and **6ad** (β-derived) in yields of 60 and 77%, respectively. The effects of different substituents on boronic acids have been reviewed by the Sporzyński group.^41,42^

To demonstrate the application and viability of this regioselective Chan–Lam coupling reaction of boldine (**3**), multigram scale-up reactions were performed under the optimized reaction conditions (Scheme 1). The reaction with phenylboronic acid afforded 9-*O*-phenylboldine (**6a**) in good yields (76% yield, 5 mmol and 62% yield, 10 mmol scale). To further explore the chemical space of boldine and address the feasibility of functionalizing the less reactive O2 position, we conducted an additional experiment using **6a** and 4-methylphenylboronic acid as substrates to perform a second Chan–Lam cross-coupling. While O2-arylation was successfully achieved under modified conditions, specifically at elevated temperatures (50 °C), a diarylated product was obtained with significantly lower yielding (**6ae**, 15%) compared to the O9-regioselective reaction for the same boronic acid (**6p**, 62%). These results are consistent with the differential acidity of the hydroxyl groups, supporting the observation that O9 remains as the more reactive site. Although the synthesis of diarylated derivatives represents a viable chemical route, the diminished yields suggest that specific optimization would be required.

Halogenated derivatives of boldine (**1**) can be viewed as attractive structures in the design and synthesis of ligands for monoaminergic central nervous system receptors. An example is 3-bromoboldine (**8**), which exhibits several-fold improved affinity and selectivity for α_1A_-adrenergic and D_1_-like dopaminergic receptors compared to its unsubstituted counterpart (**1**).^11,12^ Therefore, we decided to explore the applicability of the optimized catalytic system employing 3-bromoboldine (**8**) as a substrate for coupling with different phenylboronic acids (Scheme 3). Thus, the reaction of 3-bromoboldine (**8**) with phenylboronic acid afforded **9a** in 37% yield. When *para*-*tert*-butyl- and *para*-fluorophenylboronic acids were employed, the corresponding products **9b** and **9c** were obtained in 29% and 26% yields, respectively. Additionally, using *para*-trifluoromethyl- and *para*-trifluoromethoxyphenylboronic acids led to the formation of products **9d** and **9e**, both in 37% yield. Accordingly, the reaction conditions tolerated substitution at the C-3 position of the boldine scaffold with a bromine atom. However, the products were formed in lower yields compared to those obtained with the natural substrate. This observation may be attributable to alterations in *p*Ka values induced by the presence of the bromine substituent at C-3. Regardless of this last point, the halogen function could be further functionalized, for example, into a biaryl moiety, through Suzuki–Miyaura coupling reactions. Such extensions would enable the application of this methodology to the construction of more complex building blocks.^43,44^

**Scheme 3:**
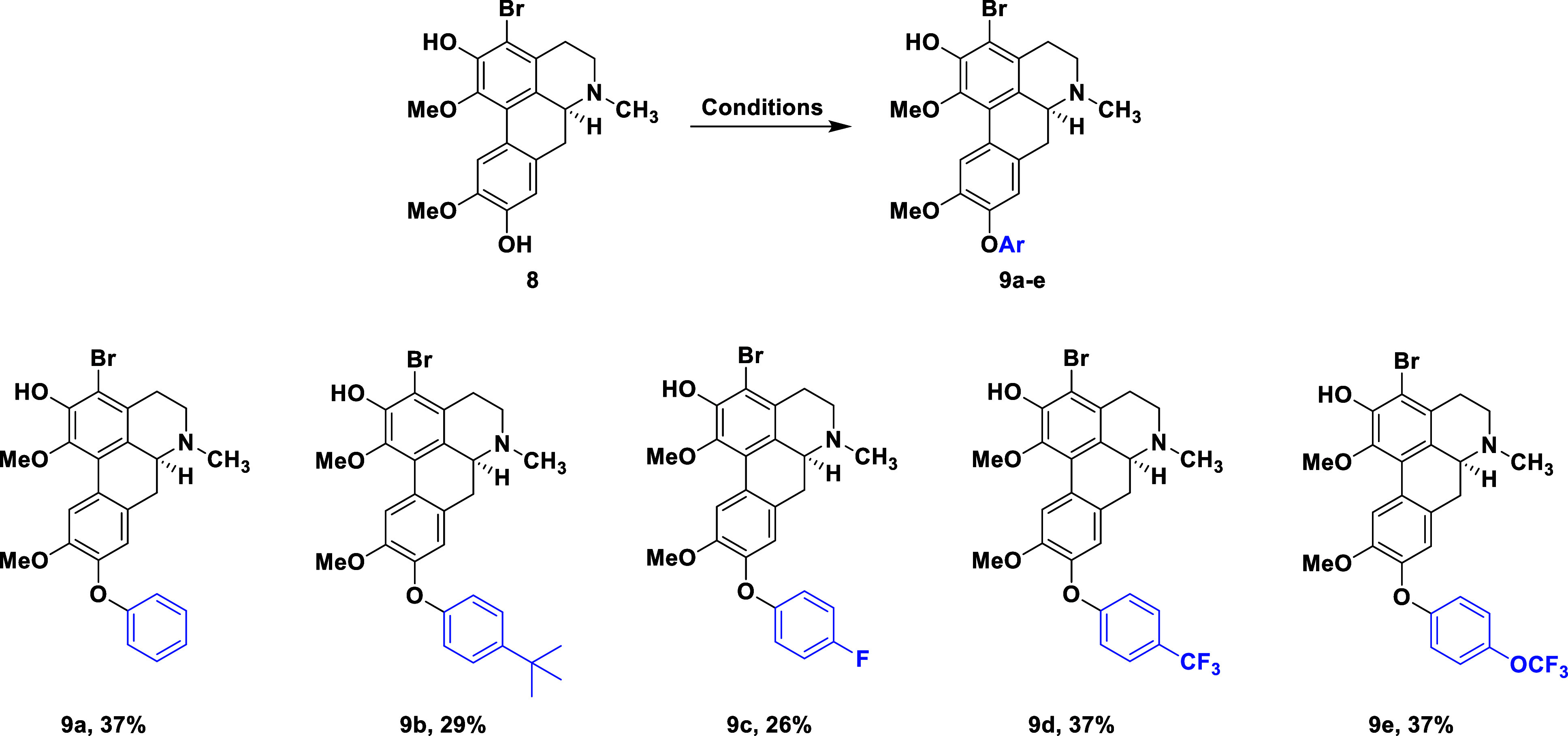
9-*O*-Arylation of 3-Bromoboldine[Fn s3fn1]

a
**Reaction conditions**: 3-Bromoboldine (**8**, 1.0 mmol), THF (6.0 mL), phenylboronic acid (2 mmol), Cu­(OTf)_2_ (20 mol·%), and pyridine (2 mmol) in an open flask at room temperature for 24 h. Isolated yield after column chromatography.

Another structural modification of aporphine alkaloids associated with potentially useful pharmacological activities is quaternization of the basic nitrogen atom and more specifically through methylation. *N*-Methylboldinium and other closely related aporphine derivatives inhibit neuronal nicotinic acetylcholine receptors at low micromolar concentrations.^45^ The natural quaternary aporphinoid magnoflorine (*N*-methylcorytuberinium) has been widely studied and exhibits varied pharmacology including, in recent years, possible beneficial effects in metabolic syndrome^46^ and in an in vivo model of Alzheimer’s disease.^47^ Given their ready preparation, some *N*,*N*-dimethylammonium iodide derivatives were synthesized using methyl iodide in acetone. Thus, **6a** and **7a** furnished quaternary products **11a** and **11b** in 90 and 93% yields, respectively. In addition, acetylated derivative **10a** produced **11c** in 59% yield under the same experimental conditions. See Scheme 4.

**Scheme 4:**
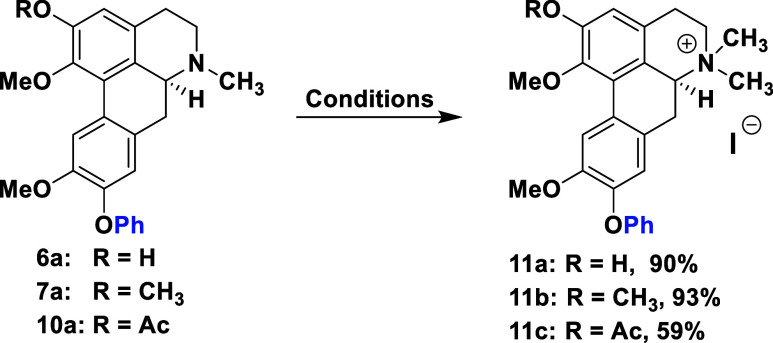
Synthesis of Quaternary Compounds[Fn s4fn1]

a
**Reaction conditions**: **6a**, **7a**, or **10a** (0.25 mmol), CH_3_I (8 mmol), acetone (1 mL), rt, 8 h.

Molecular diversity is extremely important in the search for new biologically active chemical entities, and natural products are important starting materials for the synthesis of new compounds showing interesting bioactivities.^48–50^ Inspired by this concept, we decided to use compound **6w** to synthesize some new variabiline analogues. The extremely unusual, highly lipophilic dibenzylaminoaporphine variabiline was isolated from the plant *Ocotea variabilis* (Lauraceae)^51^ and was recently reported to enhance the activity of the polymyxin antibiotic colistin against colistin-susceptible strains of *Klebsiella pneumoniae* and *Acinetobacter baumannii*.^52^ Subsequently, the same authors reported the synthesis of variabiline enantiomers and analogues confirming that colistin potentiation is most likely achieved through permeabilization of the bacterial outer membrane, allowing the antibiotic to access the inner membrane at reduced extracellular concentrations.^53^ Inspired by these results, **6w** was used to obtain new amino-functionalized aporphine derivatives using anilines and benzylamines under reductive amination conditions. Accordingly, **6w** reacted with benzylamine and *para*-methoxybenzylamine, producing **12a** and **12b** in 44 and 77% yields, respectively. Finally, the reactions of **6w** with *para*-toluidine and 2-naphthylamine furnished **12c** and **12d** in 68 and 71% yields, respectively. See Scheme 5.

**Scheme 5:**
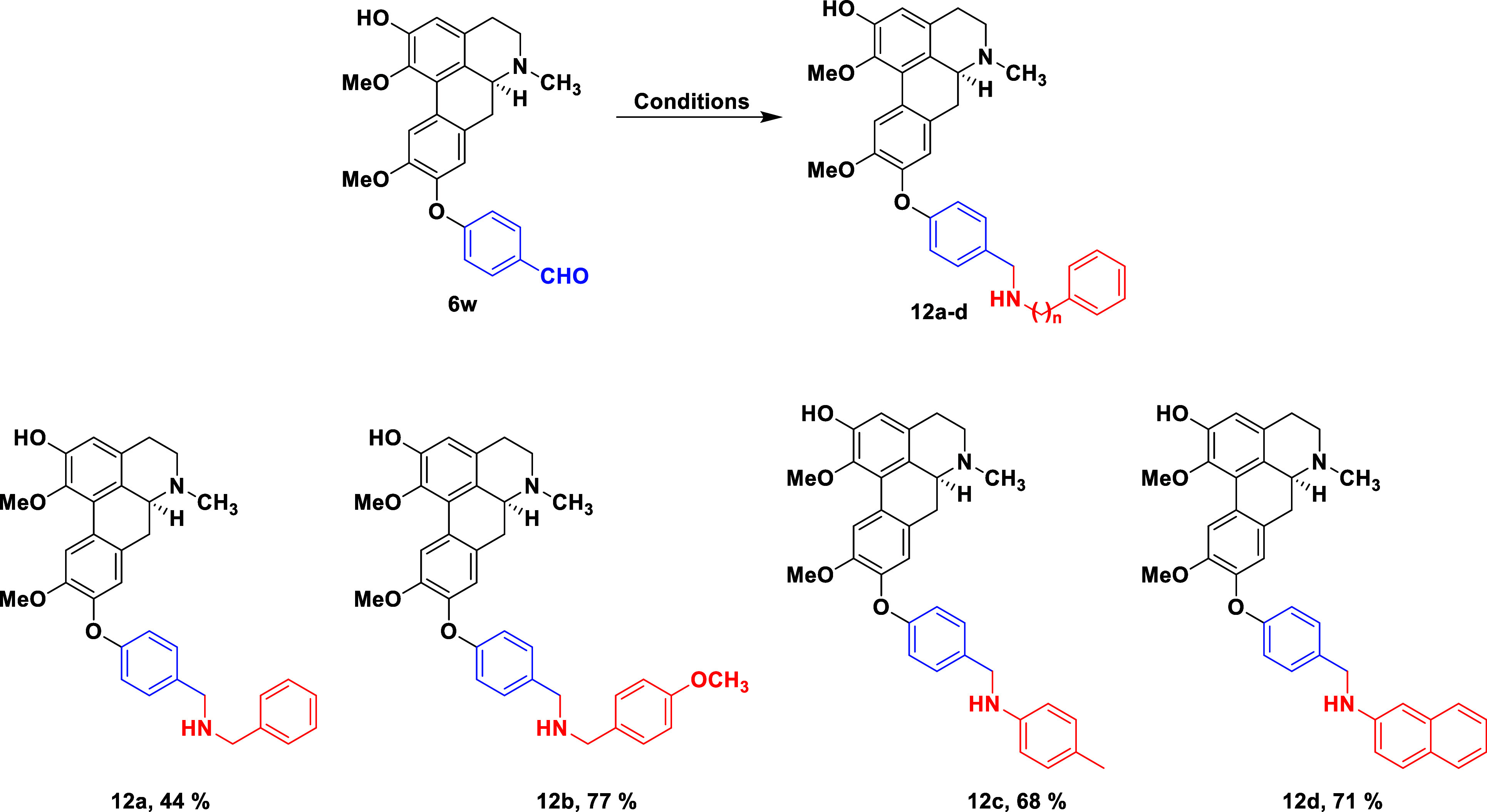
Synthesis of Aporphine Amine Derivatives[Fn s5fn1]

a
**Reaction Conditions: 6w** (0.3 mmol), benzylamine or aniline (0.32 mmol), MeOH (1.2 mL), 4 h, rt; **ii**. NaBH_4_ (0.48 mmol), 20 min.

Boldine exhibits modest antiproliferative activity in several different cancer cell lines,^4^ while none of the published studies address the mechanisms involved. Taking this into account and seeking possible applications of the newly synthesized compounds, all our derivatives listed in Scheme 2 were screened for cytotoxic activity against the acute myelogenous leukemia cell line MV4-11. Most of the new boldine derivatives yielded low micromolar GI_50_ values (Table S1). In general, they were all much weaker cytotoxic agents than the reference drugs sorafenib (a multiprotein kinase inhibitor) and cisplatin (a DNA binder). However, derivatization at the 9-*O* position enhanced the cytotoxic properties of the parent compound boldine in several analogs as can be seen for 9-*O*-phenylboldine **6a**, with the lowest GI_50_ of the series at 10.6 μM, followed by **6z** (12.1 μM), **6x** (13.5 μM), **6k** (13.6 μM), **6t** (14.2 μM), and **6f** (14.9 μM) as the most promising derivatives. Although most of our compounds presented GI_50_ values between 10 and 20 μM, 2′-methoxy **6b**, 2′-methoxycarbonyl **6m**, and bulkier 9-*O*-aryl derivatives **6aa**–**6ad** showed no clear improvement over boldine in the MV4-11 cancer cell line. Regarding the 3-bromoboldine analogs, although their synthesis demonstrates the versatility of our regioselective method, a comprehensive SAR study for these halogenated scaffolds derivatives was considered beyond the current scope of this article. Given that halogenation significantly alters the electronic profile and potentially the biological targets of the aporphine skeleton, these specific analogs deserve a dedicated follow-up investigation.

Based on their MV4-11 cytotoxic profiles, 9-*O*-arylboldine derivatives are stronger anticancer agents than boldine, even if they show no obvious structure–activity relationship. Therefore, some of these new compounds are promising agents for further analysis. These preliminary results provide insights into potential modifications to enhance the activity of the boldine derivatives.

This study reports a novel synthetic methodology for the regioselective modification of boldine, putting to good use a copper-catalyzed Chan–Lam coupling reaction. The regioselectivity of the transformation was carefully confirmed through a combination of chemical derivatization and complete one- and two-dimensional NMR analysis. Furthermore, the synthetic usefulness of this method was demonstrated by generating selected products to diversify the boldine scaffold. This led to the successful production of new aporphinium salts and aminoaporphine derivatives, which hold promise as potential biologically active compounds. Finally, boldine and 30 new 9-*O*-arylboldine derivatives were tested in a cytotoxicity assay against MV4-11 cancer cells, most of them exhibiting low micromolar GI_50_ values (between 10 and 20 μM) with better anticancer profiles than unmodified boldine, suggesting a novel route to potential anticancer therapeutics.

Finally, in an effort to further assess the generality and applicability of the Chan–Lam reaction, the use of other natural products as substrates is currently under investigation.

## Supplementary Material



## Notes

Detailed experimental procedures for the synthesis of 9-*O*-arylboldine derivatives (**6a**–**ad**), 2-*O*-(4-methylphenyl)-9-*O*-phenylboldine (**6ae**), (*S*)-1,2,10-trimethoxy-6-methyl-9-phenoxy-5,6,6a,7-tetrahydro-4*H*-dibenzo­[*de*,*g*]­quinoline (**7a**), 9-*O*-aryl derivatives of 3-bromoboldine (**9a**–**e**), (*S*)-1,10-dimethoxy-6-methyl-9-phenoxy-5,6,6a,7-tetrahydro-4*H*-dibenzo­[*de*,*g*]­quinolin-2-yl acetate (**10a**), quaternary derivatives of functionalized 9-*O*-arylboldines (**11a**–**c**), and aminoaporphine derivatives (**12a**–**d**); compound characterization of all compounds and corresponding ^1^H and ^13^C DEPT-135 NMR spectra; and detailed procedure for the cytotoxic assay and growth inhibition on the MV4-11 cancer cell line (Table S1) (PDF) (PDF)

## ⊥

B.K. Cassels Deceased on September 17, 2025.

## Notes

Dedicated to the memory of Prof. Bruce K. Cassels, a pioneer in medicinal chemistry and natural products research in Chile.
