# Supplementary material for: Regioselective Synthesis of 9‑O‑Arylboldine Derivatives Using the Copper-Catalyzed Chan–Lam Reaction†
Source: ACS Omega. 2026 Jun 9;11(24):36302–10. doi: 10.1021/acsomega.5c09458 (PMC13294923; doi:10.1021/acsomega.5c09458)
Supplement: Supplementary file 1 [file ao5c09458_si_001.pdf]

Supplementary Information

Regioselective Synthesis of 9-*O*-arylboldine derivatives using the Copper-Catalyzed Chan-Lam reaction

Cristian Suárez-Rozas,[a,b§] Daniel A. A. Araya-Santelices,[a§] Oriel A. Sánchez-Velasco,[a] Cristóbal Hormazábal-Campos,[a] JeanLuc Bertrand,[a] Petra Krňávková,[c] Veronika Vojáčková,[c] Vladimir Kryštof,[c] Bruce K. Cassels[d♦] and Edwin G. Pérez[a*]

[a]*Department of Organic Chemistry, Faculty of Chemistry and Pharmacy, Pontificia Universidad Católica de Chile, Santiago 7820436, Chile.*

[b]*Centro de Química Médica, Facultad de Medicina Clínica Alemana, Universidad del Desarrollo, Santiago 7610315, Chile.*

[c]*Department of Experimental Biology, Faculty of Science, Palacký University, Šlechtitelů 27, 77900 Olomouc, Czech Republic*

[d]*Department of Chemistry, Faculty of Sciences, University of Chile, Santiago 7800003, Chile.*

♦B. K. Cassels Deceased on September 17, 2025.

§C. S-R. and D. A. A. A-S. contributed equally to this work.

* Corresponding author. E-mail: eperezh@uc.cl

List of the Contents

1.- Experimental Section........1

1.1.- General........1

1.2.- General synthesis procedures........1

1.2.1- Synthesis of 9-*O*-arylboldine derivatives (**6a-ad)**........2

1.2.2- Synthesis of 2-*O*-(4-methylphenyl)-9-*O*-phenylboldine (**6ae)**........2

1.2.3- Synthesis of (*S*)-1,2,10-trimethoxy-6-methyl-9-phenoxy-5,6,6a,7-tetrahydro-4*H*-dibenzo[*de*,*g*]quinoline (**7a**)........2

1.2.4- Synthesis of 9-*O*-aryl derivatives of 3-bromoboldine (**9a-e**)........3

1.2.5- Synthesis of (*S*)-1,10-dimethoxy-6-methyl-9-phenoxy-5,6,6a,7-tetrahydro-4*H*-dibenzo[*de*,*g*]quinolin-2-yl acetate (**10a**)........3

1.2.6- Synthesis of quaternary derivatives of functionalized 9-*O*-arylboldines (**11a-c**)........3

1.2.7- Synthesis of aminoaporphine derivatives (**12a-d**)........3

2.- Compounds characterization........2

3.- ^{1}H NMR, ^{13}C NMR and ^{19}F NMR spectra of all compounds........18

4.- Cytotoxic Assay........90

1.- Experimental Section

1.1.- General

All solvents including deuterated solvents were purchased from Merck. Other reagents were from Aldrich, Merck or AK Scientific. Reactions run at room temperature were performed between 20 and 25 °C. Solvent evaporations were conducted under reduced pressure at temperatures less than 55 °C unless otherwise noted. Anhydrous Na_2SO_4 was used to dry organic layers after extraction, and it was removed by filtration through a cotton pad. The filtrate was concentrated and subjected to further purification protocols if necessary. All reactions were magnetically stirred and monitored for completion by normal phase Thin Layer Chromatography (TLC). Analytical TLC was performed on Merck precoated silica gel 60 F254 plates. TLC was visualised by one of the following methods: use of UV light (254 nm) or immersion into an iodine vapor or Dragendorff's stain solution followed by heating. Column chromatography was carried out under positive pressure using 40-63 µm silica gel (Merck) and the indicated solvents [v/v; used without purification]. NMR spectra were recorded on a Bruker Avance 400 MHz spectrometer. All chemical shifts in NMR experiments are reported as ppm downfield from TMS. The following calibrations were used: $CDCl_3$ $\delta = 7.26$ and 77.16 ppm for ^1H NMR and ^{13}C NMR respectively and DMSO-d_6 $\delta =$ 2.50 and 39.52 ppm for ^1H NMR and ^{13}C NMR respectively. Coupling constants (J) are given in Hertz (Hz), and the following abbreviations are used to describe the signal multiplicity: s (singlet), bs (broad singlet), d (doublet), dd (doublet of doublet), t (triplet), td (triplet of doublets), q (quartet), quint (quintet) and m (multiplet). All NMR spectra were processed and analysed using MestRe Nova 14.1.2. Melting points were determined on a Reichert Galen III hot plate microscope apparatus and are uncorrected. High resolution mass spectrometric (HRMS) were performed using a Bruker "Compact" quadrupole time-of-flight mass spectrometry (qTOF-MS, Germany) coupled with an Apollo II ion funnel electrospray ionisation (ESI) source from the "Unidad de Secuenciación y Tecnologías Ómicas" (Facultad de Ciencias Biológicas, Pontificia Universidad Católica de Chile), Chile.

1.2.- General synthesis procedures

1.2.1- Synthesis of 9-*O*-arylboldine derivatives (**6a-ad)**

A 50 mL round bottomed flask was charged with boldine (**1**) 1:1 complex with chloroform (447 mg, 1.0 mmol), $Cu(OTf)_2$ (72 mg, 0.2 mmol), (substituted)-phenyl boronic acid (2.0 mmol) and THF (6.0 mL). The suspension was stirred for 1 min and Py (161 µL, 2.0 mmol) was added. The reaction mixture, in an open flask under air, was stirred for 24 h at room temperature. After this time, the reaction was concentrated under reduced pressure. The crude product was subjected to silica gel column chromatography (MeOH/EtOAc, 1:4 as the eluent) to provide the corresponding products in yields ranging from 35 to 80%.

1.2.2- Synthesis of 2-*O*-(4-methylphenyl)-9-*O*-phenylboldine (**6ae)**

A 50 mL round bottomed flask was charged with 9-*O*-phenylboldine (**6a**, 80.7 mg, 0.2 mmol), $Cu(OTf)_2$ (14.4 mg, 0.04 mmol), 4-methylphenyl boronic acid (55.5 mg, 0.4 mmol) and THF (2.0 mL). The suspension was stirred for 5 min and Py (32 µL, 0.4 mmol) was added. The reaction mixture, in an open flask under air, was stirred for 24 h at 50 °C. After this time, the reaction was concentrated under reduced pressure. The crude product was subjected to silica gel column chromatography (MeOH/EtOAc, 1:5 as the eluent) to provide the corresponding diarylated product **6ae** as a green solid (14.5 mg, 15%).

1.2.3- Synthesis of (*S*)-1,2,10-trimethoxy-6-methyl-9-phenoxy-5,6,6a,7-tetrahydro-4*H*-dibenzo[*de*,*g*]quinoline (**7a**)

In a dry Schlenk flask, under an N_2 atmosphere, **6a** (807 mg, 2.0 mmol) was dissolved in dried DMF (7.3 mL) and *N*,*N*,*N*-trimethylanilinium chloride was added (1.20 g, 7.0 mmol). The resulting mixture was stirred at room temperature for 2 minutes, and *t*BuOK (785 mg, 7.0 mmol) was added. After being stirred at 90 °C for 48 h, the reaction mixture was dried under vacuum, toluene was added, dried in a vacuum again, and finally, hexane was added and dried (to remove DMF). The crude product was subjected to silica gel column chromatography (DCM/MeOH, 96:4 as the eluent) to afford **7a** as a yellow solid (701 mg, 84%).

1.2.4- Synthesis of 9-*O*-aryl derivatives of 3-bromoboldine (**9a-e**)

A 50 mL round bottomed flask was charged with 3-bromoboldine (**8**) (406 mg, 1.0 mmol), $Cu(OTf)_2$ (72 mg, 0.2 mmol), (substituted)-phenyl boronic acid (2.0 mmol) and THF (6.0 mL). The suspension was stirred for 1 min and Py (161 µL, 2.0 mmol) was added. The reaction mixture, in an open flask under air, was stirred for 24 h at room temperature. After this time, the reaction was concentrated under reduced pressure. The crude product was subjected to silica gel column chromatography (*n*-hexane/EtOAc, 1:1 as the eluent) to provide the corresponding products in yields ranging from 26 to 37%.

1.2.5- Synthesis of (*S*)-1,10-dimethoxy-6-methyl-9-phenoxy-5,6,6a,7-tetrahydro-4*H*-dibenzo[*de*,*g*]quinolin-2-yl acetate (**10a**)

A 10 mL round bottomed flask was charged with 9-phenylboldine (**6a**, 0.5 mmol), acetic anhydride (1.5 mmol), DMAP (5 mol%) and DCM (5.0 mL). The reaction mixture, in an open flask under air, was stirred for 3 h at room temperature. Then, 5 mL of DCM was added, and the product was washed with 10% $NH_4OH_{ac.}$ (3 × 15 mL). The organic phase was dried over anhydrous Na_2SO_4, filtered, and concentrated to give the product.

1.2.6- Synthesis of quaternary derivatives of functionalized 9-*O*-arylboldines (**11a-c**)

A 10 mL round bottomed flask was charged with **6a**, **7a**, or **10a** (0.25 mmol), methyl iodide (8 mmol) and acetone (1 mL). The reaction mixture, in an open flask under air, was stirred for 8 h protected from light at room temperature. Then, the product was collected by filtration as a precipitated solid.

1.2.7- Synthesis of aminoaporphine derivatives (**12a-d**)

A mixture of **6w** (0.3 mmol) and benzylamine or aniline (0.32 mmol) in MeOH (1.2 mL) was stirred for 4 h at room temperature. Subsequently, solid $NaBH_4$ was added (0.48 mmol) portion-wise and the stirring continued at ambient temperature for 20 min. After the reaction was complete, the mixture was poured into water (30 mL) and extracted with EtOAc (50 mL

× 2). The combined organic layer-phases were dried over anhydrous Na_2SO_4, filtered and concentrated in vacuo. After removal the solvent, the crude was purified by column chromatography (EtOAc/MeOH 4:1) to give a Dark vanilla solid.

2.- Compounds characterization

(*S*)-1,10-dimethoxy-6-methyl-5,6,6a,7-tetrahydro-4*H*-dibenzo[*de*,*g*]quinoline-2,9-diol ((+)-boldine, **1**-$CHCl_3$). Beige solid; [α]=+85.6 (1.84 x 10^{-4}, $CHCl_3$). ^{1}H NMR (400 MHz, $CDCl_3$) δ 7.90 (H-11, s, 1H), 6.80 (H-8, s, 1H), 6.59 (H-3, s, 1H), 3.88 (OCH_3-10, s, 3H), 3.58 (OCH_3-10, s, 3H), 3.14 – 2.92 (H-4α, H-7α, H-5α, H-6a, m, 4H), 2.64 – 2.57 (H-4β, H-7β, m, 2H), 2.52 (s, 3H), 2.47 (H-5β, dd, J = 11.4, 4.2 Hz, 1H). ^{13}C NMR (101 MHz, $CDCl_3$) δ 148.3 (C-2), 145.8 (C-10), 145.2 (C-10), 142.3 (C-1), 130.3 (C-7a), 130.0 (C-3a), 126.9 (C-1a), 126.1 (C-1b), 123.8 (C-11a), 114.4 (C-8), 113.4 (C-3), 110.4 (C-11), 62.7 (C-6a), 60.4 (C-1, OCH_3), 56.2 (C-10, OCH_3), 53.5 (C-5), 44.0 (NCH_3), 34.2 (C-7), 29.0 (C-4).

(*S*)-1,10-dimethoxy-6-methyl-9-phenoxy-5,6,6a,7-tetrahydro-4*H*-dibenzo[*de*,*g*]quinolin-2-ol (**6a**). Hazel solid; mp: 97-100 °C. ^{1}H NMR (400 MHz, $CDCl_3$) δ 8.06 (H-11, s, 1H), 7.34 (OPh(H-3, H-5), td, *J* = 7.5, 2.0 Hz, 2H), 7.09 (OPh(H-4), t, *J* = 7.4 Hz, 1H), 7.04 (OPh(H-2, H-6) d, *J* = 7.7 Hz, 2H), 6.82 (H-8, s, 1H), 6.64 (H-3, s, 1H), 3.88 (OCH_3-1, s, 3H), 3.64 (OCH_3-10, s, 3H), 3.12 – 2.99 (H-4α, H-5α, H-7α, m, 3H), 2.92 (H-6a, dd, *J* = 13.8, 4.1 Hz, 1H), 2.68 – 2.52 (H-4β, H-7β, H-5β, m, 3H), 2.51 (NCH_3, s, 3H). ^{13}C NMR (101 MHz, $CDCl_3$) δ 157.5 (OPh(C-1)), 150.0 (C-2), 148.4 (C-9), 144.8 (C-10), 142.7 (C-1), 130.0 (C-7a), 129.7 (OPh(C-3, C5)), 129.6 (C-3a), 127.8 (C-1a), 126.9 (C-1b), 125.7 (C-11a), 123.1 (OPh(C-4)), 119.6 (C-8), 118.1 (OPh(C-2, C6)), 114.2 (C-3), 112.2 (C-11), 62.6 (C-6), 60.5 (C-1, OCH_3), 56.4 (C-10, OCH_3), 53.5 (C-5), 43.9 (NCH_3), 34.0 (C-7), 28.9 (C-4). Calcd. for $C_{25}H_{25}NO_4+H^+$: 404.1857. Found: 404.1868.

(*S*)-1,10-dimethoxy-9-(2-methoxyphenoxy)-6-methyl-5,6,6a,7-tetrahydro-4*H*-dibenzo[*de*,*g*]quinolin-2-ol (**6b**). Beige solid; mp: 84-85 °C. ^{1}H NMR (400 MHz, $CDCl_3$) δ 8.02 (H-11, s, 1H), 7.12 (OPh(H-4), t, *J* = 7.6 Hz, 1H), 7.02 (OPh(H-3), d, *J* = 8.1 Hz, 1H), 6.98 (OPh(H-6), d, *J* = 7.8 Hz, 1H), 6.92 (OPh(H-5), t, *J* = 7.5 Hz, 1H), 6.64 (H-3, H-8 s, 2H), 3.91 (OPh(OCH_3), s, 3H), 3.87 (OCH_3-1, s, 3H), 3.62 (OCH_3-10, s, 3H), 3.15 – 2.97 (H-4α, H-5α, H-7α, H-4β, m, 4H), 2.87 (H-6a, dd, *J* = 13.8, 4.1 Hz, 1H), 2.63 (H-7β, dd, *J* = 16.0, 3.7 Hz, 1H), 2.53 (H-5β d, *J* = 13.3 Hz, 1H), 2.49 (NCH_3, s, 3H).^{13}C NMR (101 MHz,

$CDCl_3$) δ 151.0, 149.0, 148.3, 145.8, 145.3, 142.5, 129.7, 129.2, 126.7, 126.5, 125.7, 124.5, 121.0, 120.1, 117.1, 113.8, 112.8, 111.8, 77.1, 62.4, 60.4, 56.2, 56.0, 53.3, 43.7, 33.8, 28.7. Calcd. for $C_{26}H_{27}NO_5+H^+$: 434.1962. Found: 434.1964.

(*S*)-9-(2-fluorophenoxy)-1,10-dimethoxy-6-methyl-5,6,6a,7-tetrahydro-4*H*-dibenzo[*de*,*g*]quinolin-2-ol (**6c**). Beige solid; mp: 103-106 °C. ^{1}H NMR (400 MHz, $CDCl_3$) δ 8.01 (H-11, s, 1H), 7.17 (OPh(H-5), t, *J* = 10.2 Hz, 1H), 7.12 – 7.06 (OPh(H-3, H-6), m, 2H), 7.02 (OPh(H-4), t, *J* = 7.7 Hz, 1H), 6.68 (H-8, s, 1H), 6.65 (H-3, s, 1H), 3.85 (OCH_3-1, s, 3H), 3.75 (H-4α, d, *J* = 14.7 Hz, 1H), 3.61 (OCH_3-10, s, 3H), 3.49 (H-5α, dd, *J* = 12.0, 5.7 Hz, 1H), 3.28 (H-7α, t, *J* = 17.9 Hz, 1H), 3.09 (H-4β, t, *J* = 10.7 Hz, 1H), 2.96 (H-6a, dd, *J* = 13.7, 4.3 Hz, 1H), 2.87 (NCH_3, 3H), 2.85 – 2.74 (H-7β, H-5β, m, 2H). ^{13}C NMR (101 MHz, $CDCl_3$) δ 154.0 (d, $^1J_{C\text{-}F}$ = 248.4 Hz), 149.3, 148.4, 145.2, 144.3 (d, $^2J_{C\text{-}F}$ = 11.4 Hz), 142.7, 129.9, 129.5, 127.6, 125.6, 124.7 (d, $^3J_{C\text{-}F}$ = 3.9 Hz), 124.5 (d, $^3J_{C\text{-}F}$ = 6.8 Hz), 121.0, 120.0, 117.7, 117.1 (d, $^2J_{C\text{-}F}$ = 18.1 Hz), 114.1, 112.2, 62.5, 60.6, 56.5, 53.5, 43.9, 34.0, 28.8. ^{19}F NMR (376 MHz, $CDCl_3$) δ -131.57 (s, 1F). Calcd. for $C_{25}H_{24}FNO_4+H^+$: 422.1762. Found: 422.1771.

(S)-9-(2-chlorophenoxy)-1,10-dimethoxy-6-methyl-5,6,6a,7-tetrahydro-4*H*-dibenzo[*de*,*g*]quinolin-2-ol (**6d**). Taupe solid; mp: 114-116 °C. ^{1}H NMR (400 MHz, $CDCl_3$) δ 8.05 (H-11, s, 1H), 7.47 (OPh(H-3), d, *J* = 8.0 Hz, 1H), 7.21 (OPh(H-5), t, *J* = 7.8 Hz, 1H), 7.07 (OPh(H-4), t, *J* = 7.7 Hz, 1H), 6.94 (OPh(H-6), d, *J* = 8.1 Hz, 1H), 6.74 (H-8, s, 1H), 6.69 (H-3, s, 1H), 3.90 (OCH_3-1, s, 3H), 3.64 (OCH_3-10, s, 3H), 3.09 (H-4α, H-5α, H-7α, m, 3H), 2.93 (H-6a, dd, *J* = 14.2, 4.0 Hz, 1H), 2.64 (H-4β, H-7β, H-5β, m, 3H), 2.54 (NCH_3, s, 3H). ^{13}C NMR (101 MHz, $CDCl_3$) δ 153.1, 149.7, 148.4, 144.4, 142.7, 130.8 (2C), 130.0, 129.6, 128.1, 127.9, 125.6, 124.9, 124.2, 119.3, 119.0, 114.2, 112.5, 62.5, 60.6, 56.5, 53.4, 43.9, 33.9, 28.8. Calcd. for $C_{25}H_{24}ClNO_4+H^+$: 438.1467. Found: 438.1469.

(*S*)-1,10-dimethoxy-6-methyl-9-(*m*-tolyloxy)-5,6,6a,7-tetrahydro-4*H*-dibenzo[*de*,*g*]quinolin-2-ol (**6e**). Light brown solid; mp: 102-104 °C. ^{1}H NMR (400 MHz, $CDCl_3$) δ 8.05 (H-11, s, 1H), 7.21 (OPh(H-5), t, *J* = 7.8 Hz, 1H), 6.92 (OPh(H-6), d, *J* = 7.6 Hz, 1H), 6.88 (OPh(H-2), s, 1H), 6.83 (OPh(H-4), d, *J* = 8.1 Hz, 1H), 6.80 (H-8, s, 1H), 6.65 (H-3, s, 1H), 3.89 (OCH_3-1, s, 3H), 3.64 (OCH_3-10, s, 3H), 3.19 – 3.00 (H-4α, H-5α, H-7α, m, 3H), 2.93 (H-6a, dd, *J* = 13.8, 4.0 Hz, 1H), 2.67 – 2.54 (H-4β, H-7β, H-5β, m, 3H), 2.52

(NCH_3, s, 3H), 2.35 (OPh(CH_3), s, 3H). ^{13}C NMR (101 MHz, $CDCl_3$) δ 157.4, 150.0, 148.4, 145.1, 142.7, 140.0, 130.0, 129.6, 129.5, 127.6, 126.9, 125.7, 124.0, 119.4, 119.0, 115.2, 114.1, 112.2, 62.6, 60.6, 56.4, 53.5, 43.9, 34.0, 28.9, 21.6. Calcd. for $C_{26}H_{27}NO_4$+H^+: 418.2013. Found: 418.2017.

(*S*)-9-(3-(tert-butyl)phenoxy)-1,10-dimethoxy-6-methyl-5,6,6a,7-tetrahydro-4*H*-dibenzo[*de*,*g*]quinolin-2-ol (**6f**). Beige solid; mp: 83-85 °C. ^1H NMR (400 MHz, $CDCl_3$) δ 8.08 (H-11, s, 1H), 7.32 – 7.26 (OPh(H-6), m, 1H), 7.19 (OPh(H-5), t, J = 2.1 Hz, 1H), 7.16 (OPh(H-4), d, J = 7.8 Hz, 1H), 6.86 – 6.79 (OPh(H-4), H-8 ,m, 2H), 6.67 (H-3 ,s, 1H), 3.92 (OCH_3-1, s, 3H), 3.67 (OCH_3-10, s, 3H), 3.19 – 3.02 (H-4α, H-5α, H-7α, m, 3H), 2.94 (H-6a, dd, J = 13.8, 4.0 Hz, 1H), 2.71 – 2.55 (H-4β, H-7β, H-5β, m, 3H), 2.54 (NCH_3, s, 3H), 1.35 (OPh(*t*-Bu), s, 9H). ^{13}C NMR (101 MHz, $CDCl_3$) δ 161.0, 158.8, 150.1, 148.5, 144.5, 142.8, 130.1, 129.9, 129.5, 128.0, 126.7, 125.7, 119.8, 114.2, 112.2, 110.2, 108.6, 104.3, 62.6, 60.6, 56.4, 55.5, 53.5, 43.9, 33.9, 28.8. Calcd. for $C_{29}H_{33}NO_4$+H^+: 460.2482. Found: 460.2488.

(*S*)-1,10-dimethoxy-9-(3-methoxyphenoxy)-6-methyl-5,6,6a,7-tetrahydro-4*H*-dibenzo[*de*,*g*]quinolin-2-ol (**6g**). Beige solid; mp: 89-91 °C. ^1H NMR (400 MHz, $CDCl_3$) δ 8.05 (H-11, s, 1H), 7.27 – 7.18 (OPh(H-5), m, 1H), 6.85 (H-8, s, 1H), 6.68 – 6.61 (OPh(H-6), H-3, m, 2H), 6.64 – 6.57 (OPh(H-2, H-4), m, 2H), 3.87 (OCH_3-1, s, 3H), 3.79 (OPh(OCH_3), s, 3H), 3.63 (OCH_3-10, s, 3H), 3.14 – 3.01 (H-4α, H-5α, H-7α, m, 3H), 2.94 (H-6a, dd, J = 13.8, 4.0 Hz, 1H), 2.69 – 2.54 (H-4β, H-7β, H-5β, m, 3H), 2.53 (NCH_3, s, 3H). ^{13}C NMR (101 MHz, $CDCl_3$) δ 161.0, 158.8, 150.1, 148.5, 144.5, 142.8, 130.1, 129.9, 129.5, 128.0, 125.7, 119.8, 114.2, 112.2, 110.2, 108.6, 104.3, 62.6, 60.6, 56.4, 55.5, 53.5, 43.9, 33.9, 28.7. Calcd. for C26H27NO5: 434.1962. Found: 434.1963.

(*S*)-9-(3-fluorophenoxy)-1,10-dimethoxy-6-methyl-5,6,6a,7-tetrahydro-4*H*-dibenzo[*de*,*g*]quinolin-2-ol (**6h**). Light brown solid; mp: 83-85 °C. ^1H NMR (400 MHz, $CDCl_3$) δ 8.08 (H-11, s, 1H), 7.30 – 7.20 (OPh(H-2), m, 1H), 6.90 (H-8, s, 1H), 6.79 (OPh(H-4), d, J = 8.5 Hz, 1H), 6.75 (OPh(H-5), d, J = 8.3 Hz, 1H), 6.70 (OPh(H-6), d, J = 10.4 Hz, 1H), 6.63 (H-3, s, 1H) 3.85 (OCH_3-1, s, 3H), 3.63 (OCH_3-10, s, 3H), 3.14 – 3.01 (H-4α, H-5α, H-7α, m, 3H), 2.96 (H-6a, dd, J = 13.8, 4.1 Hz, 1H), 2.65 – 2.54 (H-4β, H-7β, H-5β, m, 3H), 2.52 (NCH_3, s, 3H). ^{13}C NMR (101 MHz, $CDCl_3$) δ 163.6 (d, $^1J_{C-F}$ = 246.1 Hz), 159.3

(d, $^{3}J_{C\text{-}F}$ = 10.6 Hz), 150.3, 148.5, 143.4, 142.9, 130.4 (d, $^{3}J_{C\text{-}F}$ = 9.8 Hz), 130.0, 129.8, 128.9, 127.0, 125.6, 120.7, 114.5, 113.0 (d, $^{4}J_{C\text{-}F}$ = 3.0 Hz), 109.5 (d, $^{2}J_{C\text{-}F}$ = 21.3 Hz), 105.0 (d, $^{2}J_{C\text{-}F}$ = 24.9 Hz), 62.5, 60.5, 56.3, 53.4, 44.0, 33.9, 28.9. ^{19}F NMR (376 MHz, $CDCl_3$) δ -111.34 (s, 1F). Calcd. for $C_{25}H_{24}FNO_4+H^+$: 422.1762. Found: 422.1771.

(*S*)-9-(3-chlorophenoxy)-1,10-dimethoxy-6-methyl-5,6,6a,7-tetrahydro-4*H*-dibenzo[*de*,*g*]quinolin-2-ol (**6i**). Beige solid; mp: 94-96 °C. ^{1}H NMR (400 MHz, $CDCl_3$) δ 8.08 (H-11, s, 1H), 7.23 (OPh(H-5), t, *J* = 8.0 Hz, 1H), 7.04 (OPh(H-4), d, *J* = 7.9 Hz, 1H), 6.98 (OPh(H-2), s, 1H), 6.90 (OPh(H-6), d, *J* = 8.8 Hz, 1H), 6.88 (H-8, s, 1H), 6.63 (H-3, s, 1H), 3.84 (OCH_3-1, s, 3H), 3.63 (OCH_3-10, s, 3H), 3.10 – 3.00 (H-4α, H-5α, H-7α, m, 3H), 2.96 (H-6a, dd, *J* = 13.9, 4.1 Hz, 1H), 2.65 – 2.54 ((H-4β, H-7β, H-5β, m, 3H), 2.52 (NCH_3, s, 3H). ^{13}C NMR (101 MHz, $CDCl_3$) δ 158.7, 150.2, 148.5, 143.4, 142.9, 135.0, 130.4, 130.0, 129.8, 128.9, 127.0, 125.6, 122.9, 120.6, 117.7, 115.7, 114.5, 112.4, 62.5, 60.5, 56.3, 53.4, 44.0, 33.9, 28.9. Calcd. for $C_{25}H_{24}ClNO_4+H^+$: 438.1467. Found: 438.1471.

(*S*)-9-(3-bromophenoxy)-1,10-dimethoxy-6-methyl-5,6,6a,7-tetrahydro-4*H*-dibenzo[*de*,*g*]quinolin-2-ol (**6j**). Light brown solid; mp: 81-82 °C. ^{1}H NMR (400 MHz, $CDCl_3$) δ 8.07 (H-11, s, 1H), 7.23 – 7.12 (OPh(H-2, H-4, H-5), m, 3H), 6.94 (OPh(H-6, dt, *J* = 7.2, 2.3 Hz, 1H), 6.87 (H-8, s, 1H), 6.66 (H-3, s, 1H), 3.85 (OCH_3-1, s, 3H), 3.64 (OCH_3-10, s, 3H), 3.11 – 3.00 (H-4α, H-5α, H-7α, m, 3H), 2.96 (H-6a, dd, *J* = 13.8, 3.9 Hz, 1H), 2.68 – 2.55 (H-4β, H-7β, H-5β, m, 3H), 2.51 (NCH_3, s, 3H). ^{13}C NMR (101 MHz, $CDCl_3$) δ 158.7, 150.2, 148.3, 143.4, 142.7, 130.8, 130.2, 129.9, 128.9, 127.2, 125.8, 125.5, 122.9, 120.7, 120.6, 116.2, 114.4, 112.3, 62.5, 60.6, 56.3, 53.5, 44.0, 34.0, 29.0. Calcd. for $C_{25}H_{24}BrNO_4+H^+$: 482.0961. Found: 482.0969.

(*S*)-9-(3-iodophenoxy)-1,10-dimethoxy-6-methyl-5,6,6a,7-tetrahydro-4*H*-dibenzo[*de*,*g*]quinolin-2-ol (**6k**). Light brown solid; mp: 103-106 °C. ^{1}H NMR (400 MHz, $CDCl_3$) δ 8.06 (H-11, s, 1H), 7.40 (OPh(H-4), dt, *J* = 7.8, 1.5 Hz, 1H), 7.35 – 7.34 (H-2, m, 1H), 7.03 (OPh(H-5), t, *J* = 8.0 Hz, 1H), 6.97 (OPh(H-6), ddd, *J* = 8.3, 2.3, 0.9 Hz, 1H), 6.86 (H-8, s, 1H), 6.66 (H-3, s, 1H), 3.85 (OCH_3-1, s, 3H), 3.64 (OCH_3-10, s, 3H), 3.12 – 3.03 (H-4α, H-5α, H-7α, m, 3H), 2.96 (H-6a, dd, *J* = 13.8, 4.0 Hz, 1H), 2.68 – 2.56 (H-4β, H-7β, H-5β, m, 3H), 2.54 (NCH_3, s, 3H). ^{13}C NMR (101 MHz, $CDCl_3$) δ 158.4, 150.2, 148.4, 143.6, 142.8, 131.9, 131.0, 130.0, 129.7, 128.7, 126.8, 126.6, 125.6, 120.4, 117.0, 114.4, 112.3,

94.3, 62.5, 60.6, 56.3, 53.5, 43.9, 33.9, 28.8. Calcd. for $C_{25}H_{24}INO_4+H^+$: 530.0823. Found:530.0827.

(*S*)-1,10-dimethoxy-6-methyl-9-(3-(trifluoromethoxy)phenoxy)-5,6,6a,7-tetrahydro-4*H*-dibenzo[de,g]quinolin-2-ol (**6l**). Beige solid; mp: 148-150 °C. ^{1}H NMR (400 MHz, $CDCl_3$) δ 8.08 (H-11, s, 1H), 7.31 (OPh(H-5), t, *J* = 8.3 Hz, 1H), 6.93 (OPh(H-4), d, *J* = 2.2 Hz, 1H), 6.91 (OPh(H-2, H-6), d, *J* = 2.8 Hz, 2H), 6.85 (H-8, s, 1H), 6.66 (H-3, s, 1H), 3.84 (OCH_3-1, s, 3H), 3.64 (OCH_3-10, s, 3H), 3.12 – 3.00 (H-4α, H-5α, H-7α, m, 3H), 2.97 (H-6a, dd, *J* = 13.8, 4.0 Hz, 1H), 2.69 – 2.52 (H-4β, H-7β, H-5β, m, 3H), 2.53 (NCH_3, s, 3H). ^{13}C NMR (101 MHz, $CDCl_3$) δ 159.1, 150.3, 150.2 (d, $^4J_{C\text{-}F}$ = 1.8 Hz), 148.4, 143.2, 142.8, 130.4, 130.1, 129.9, 129.1, 127.1, 125.5, 120.8, 120.6 (q, $^1J_{C\text{-}F}$ = 257.4 Hz), 115.4, 114.7, 114.5, 112.4, 110.3, 62.5, 60.6, 56.2, 53.5, 44.0, 34.0, 28.9. ^{19}F NMR (376 MHz, $CDCl_3$) δ -57.79 (s, 3F). Calcd. for $C_{26}H_{24}F_3NO_5+H^+$: 488.1679. Found: 488.1687.

(*S*)-3-((2-hydroxy-1,10-dimethoxy-6-methyl-5,6,6a,7-tetrahydro-4*H*-dibenzo[de,g]quinolin-9-yl)oxy)benzoate (**6m**). Beige solid; mp: 90-93 °C. ^{1}H NMR (400 MHz, $CDCl_3$) δ 8.07 (H-11, s, 1H), 7.76 (OPh(H-4), dt, *J* = 7.6, 1.1 Hz, 1H), 7.65 (OPh(H-2), dd, *J* = 2.6, 1.6 Hz, 1H), 7.39 (OPh(H-5), t, *J* = 7.9 Hz, 1H), 7.22 (OPh(H-6), ddd, *J* = 8.2, 2.6, 0.8 Hz, 1H), 6.84 (H-8, s, 1H), 6.66 (H-3, s, 1H), 3.89 (OPh(CO_2CH_3), s, 3H), 3.85 (OCH_3-1, s, 4H), 3.64 (OCH_3-10, s, 3H), 3.11 (H-4α, H-5α, H-7α, m, 3H), 2.94 (H-6a, dd, *J* = 13.9, 4.0 Hz, 1H), 2.69 – 2.55 (H-4β, H-7β, H-5β, m, 3H), 2.54 (NCH_3, s, 3H). ^{13}C NMR (101 MHz, $CDCl_3$) δ 175.6, 166.8, 157.8, 150.2, 148.6, 144.0, 142.8, 131.9, 129.7, 129.5, 128.5, 126.4, 125.6, 124.1, 122.4, 120.2, 118.6, 114.4, 112.4, 62.4, 60.6, 56.3, 53.3, 52.4, 43.6, 33.7, 28.5. Calcd. for $C_{27}H_{27}NO_6+H^+$: 462.1911. Found: 462.1920.

(*S*)-1,10-dimethoxy-6-methyl-9-(3-(trifluoromethyl)phenoxy)-5,6,6a,7-tetrahydro-4*H*-dibenzo[*de*,*g*]quinolin-2-ol (**6n**). Beige solid; mp: 84-86 °C. ^{1}H NMR (400 MHz, $CDCl_3$) δ 8.08 (H-11, s, 1H), 7.42 (OPh(H-5), t, *J* = 8.0 Hz, 1H), 7.32 (OPh(H-4), d, *J* = 7.7 Hz, 1H), 7.24 (OPh(H-2), s, 1H), 7.15 (OPh(H-6), dd, *J* = 8.2, 2.2 Hz, 1H), 6.90 (H-8, s, 1H), 6.67 (H-3, s, 1H), 3.84 (OCH_3-1, s, 3H), 3.65 (OCH_3-10, s, 3H), 3.17 – 3.06 (H-4α, H-5α, H-7α, m, 3H), 2.98 (H-6a, dd, *J* = 13.8, 4.0 Hz, 1H), 2.69 – 2.58 (H-4β, H-7β, H-5β, m, 3H), 2.56 (NCH_3, s, 3H). ^{13}C NMR (101 MHz, $CDCl_3$) δ 158.2, 150.4, 148.5, 143.2, 142.9, 132.2 (q, $^2J_{C\text{-}F}$ = 32.6 Hz), 130.2 (2C), 129.9, 129.7, 129.1, 125.6, 123.9 (q, $^1J_{C\text{-}F}$ = 272.5 Hz), 120.7,

120.4 (d, $^4J_{C\text{-}F}$ = 1.1 Hz), 119.3 (q, $^3J_{C\text{-}F}$ = 3.9 Hz), 114.5, 114.3 (q, $^3J_{C\text{-}F}$ = 3.9 Hz), 112.4, 62.5, 60.6, 56.3, 53.4, 43.8, 33.8, 28.7. ^{19}F NMR (376 MHz, $CDCl_3$) δ -62.64 (s, 3F). Calcd. for $C_{26}H_{24}F_3NO_4+H^+$: 472.1730. Found: 472.1741.

(*S*)-1,10-dimethoxy-6-methyl-9-(3-nitrophenoxy)-5,6,6a,7-tetrahydro-4*H*-dibenzo[*de,g*]quinolin-2-ol (**6o**). Brown solid; mp: 114-117 °C. ^{1}H NMR (400 MHz, $CDCl_3$) δ 8.10 (H-11, s, 1H), 7.91 (OPh(H-4), ddd, *J* = 8.0, 1.9, 0.7 Hz, 1H), 7.75 (OPh(H-2), t, *J* = 2.3 Hz, 1H), 7.46 (OPh(H-5), t, *J* = 8.2 Hz, 1H), 7.32 (OPh(H-6), ddd, *J* = 8.3, 2.4, 0.7 Hz, 2H), 6.96 (H-8, s, 1H), 6.68 (H-3, s, 1H), 3.82 (OCH_3-1, s, 3H), 3.65 (OCH_3-10, s, 3H), 3.07 (H-4α, H-5α, H-7α, m, 3H), 2.99 (H-6a, dd, *J* = 13.8, 4.0 Hz, 1H), 2.70 – 2.55 (H-4β, H-7β, H-5β, m, 3H), 2.53 (NCH_3, s, 3H). ^{13}C NMR (101 MHz, $CDCl_3$) δ 158.9, 150.4, 149.4, 148.4, 142.8, 142.3, 130.2, 130.2, 130.0, 129.8, 127.1, 125.4, 123.1, 121.3, 117.3, 114.6, 112.5, 111.6, 62.4, 60.7, 56.2, 53.4, 43.9, 33.9, 28.9. Calcd. for $C_{25}H_{24}N_2O_6+H^+$: 449.1707. Found: 449.1717.

(*S*)-1,10-dimethoxy-6-methyl-9-(p-tolyloxy)-5,6,6a,7-tetrahydro-4*H*-dibenzo[*de,g*]quinolin-2-ol (**6p**). Light brown solid; mp: 92-94 °C. ^{1}H NMR (400 MHz, $CDCl_3$) δ 8.04 (H-11, s, 1H), 7.14 (OPh(H-3, H-5), d, *J* = 8.2 Hz, 2H), 6.96 (OPh(H-2, H-6), d, *J* = 8.5 Hz, 2H), 6.75 (H-8, s, 1H), 6.63 (H-3, s, 1H), 3.89 (OCH_3-1, s, 3H), 3.63 (OCH_3-10, s, 3H), 3.08 – 2.95 (H-4α, H-5α, H-7α, m, 3H), 2.90 (H-6a, dd, *J* = 13.8, 4.1 Hz, 1H), 2.66 – 2.50 (H-4β, H-7β, H-5β, m, 3H), 2.49 (NCH_3, s, 3H), 2.34 (OPh(CH_3), s, 3H). ^{13}C NMR (101 MHz, $CDCl_3$) δ 155.0, 149.7, 148.3, 145.6, 142.6, 132.8, 130.3 (2C), 130.0, 129.7, 127.3, 127.0, 125.7, 118.7, 118.6 (2C), 114.1, 112.1, 62.6, 60.5, 56.4, 53.5, 44.0, 34.1, 29.0, 20.8. Calcd. for $C_{26}H_{27}NO_4+H^+$: 418.2013. Found: 418.2017.

(*S*)-9-(4-(*tert*-butyl)phenoxy)-1,10-dimethoxy-6-methyl-5,6,6a,7-tetrahydro-4*H*-dibenzo[*de,g*]quinolin-2-ol (**6q**). Light brown solid; mp: 107-110 °C. ^{1}H NMR (400 MHz, $CDCl_3$) δ 8.05 (H-11, s, 1H), 7.35 (OPh(H-3, H-5), d, *J* = 8.8 Hz, 2H), 6.97 (OPh(H-2, H-6), d, *J* = 8.8 Hz, 2H), 6.81 (H-8, s, 1H), 6.64 (H-3, s, 1H), 3.89 (OCH_3-1, s, 3H), 3.64 (OCH_3-10, s, 3H), 3.12 – 2.98 (H-4α, H-5α, H-7α, m, 3H), 2.92 (H-6a, dd, *J* = 13.8, 4.0 Hz, 1H), 2.69 – 2.53 (H-4β, H-7β, H-5β, m, 3H), 2.52 (NCH_3, s, 3H), 1.33 (OPh(*t*-Bu), s, 9H).^{13}C NMR (101 MHz, $CDCl_3$) δ 154.9, 149.9, 148.3, 145.8, 145.1, 142.6, 129.8, 129.4, 127.4,

126.7, 126.5 (2C), 125.6, 119.1, 117.6 (2C), 114.0, 112.1, 62.5, 60.4, 56.3, 53.4, 43.8, 34.3, 33.9, 31.5 (3C), 28.7. Calcd. for $C_{29}H_{33}NO_4+H^+$: 460.2482. Found: 460.2488.

(*S*)-1,10-dimethoxy-9-(4-methoxyphenoxy)-6-methyl-5,6,6a,7-tetrahydro-4*H*-dibenzo[*de,g*]quinolin-2-ol (**6r**). Light brown solid; mp: 82-84 °C. ^{1}H NMR (400 MHz, $CDCl_3$) δ 8.03 (H-11, s, 1H), 7.02 (OPh(H-3, H-5), d, *J* = 9.0 Hz, 2H), 6.89 (OPh(H-2, H-6), d, *J* = 9.1 Hz, 2H), 6.67 (H-8, s, 1H), 6.63 (H-3, s, 1H), 3.91 (OCH_3-1, s, 3H), 3.81 (OPh(OCH_3), s, 3H), 3.62 (OCH_3-10, s, 3H), 3.16 – 3.08 (H-4α, H-5α, H-7α, m, 3H), 2.88 (H-6a, dd, *J* = 13.8, 4.0 Hz, 1H), 2.67 – 2.56 (H-4β, H-7β, H-5β, m, 3H), 2.54 (NCH_3, s, 3H). ^{13}C NMR (101 MHz, $CDCl_3$) δ 156.0, 150.3, 149.3, 148.6, 146.6, 142.8, 129.5, 129.1, 127.1, 126.6, 125.8, 120.4 (2C), 117.5, 114.9 (2C), 114.0, 112.0, 62.4, 60.5, 56.4, 55.8, 53.2, 43.4, 33.6, 28.3. Calcd. for $C_{26}H_{27}NO_5+H^+$: 434.1962. Found: 434.1966.

(*S*)-9-(4-fluorophenoxy)-1,10-dimethoxy-6-methyl-5,6,6a,7-tetrahydro-4*H*-dibenzo[*de,g*]quinolin-2-ol (**6s**). Beige solid; mp: 97-98 °C. ^{1}H NMR (400 MHz, $CDCl_3$) δ 8.06 (H-11, s, 1H), 7.03 – 6.99 (OPh(H-2, H-3, H-5, H-6), m, 4H), 6.76 (H-8, s, 1H), 6.61 (H-3, s, 1H), 3.87 (OCH_3-1, s, 1H), 3.62 (OCH_3-10, s, 1H), 3.08 – 2.98 (H-4a, H-5a, H-7a, m, 3H), 2.91 (H-6a, dd, *J* = 13.8, 4.1 Hz, 1H), 2.63 – 2.51 (H-4b, H-7b, H-5b, m, 3H), 2.49 (NCH_3, s, 3H). ^{13}C NMR (101 MHz, $CDCl_3$) δ 158.7 (d, $^1J_{C-F}$ = 241.0 Hz), 153.3 (d, $^4J_{C-F}$ = 2.4 Hz), 149.7, 148.4, 145.1, 142.8, 130.0, 129.7, 127.8, 126.9, 125.7, 119.6 (d, $^3J_{C-F}$ = 8.2 Hz, 2C), 119.0, 116.2 (d, $^2J_{C-F}$ = 23.3 Hz, 2C), 114.3, 112.2, 62.5, 60.5, 56.3, 53.4, 44.0, 34.0, 28.9. ^{19}F NMR (376 MHz, $CDCl_3$) δ -120.66 (s, 1F). Calcd. for $C_{25}H_{24}FNO_4+H^+$: 422.1762. Found: 422.1769.

(*S*)-9-(4-chlorophenoxy)-1,10-dimethoxy-6-methyl-5,6,6a,7-tetrahydro-4*H*-dibenzo[*de,g*]quinolin-2-ol (**6t**). Light brown solid; mp: 94-98 °C. ^{1}H NMR (400 MHz, $CDCl_3$) δ 8.05 (H-11, s, 1H), 7.25 (OPh(H-3, H-5), d, *J* = 7.6 Hz, 2H), 6.93 (OPh(H-2, H-6), d, *J* = 7.4 Hz, 2H), 6.81 (H-8, s, 1H), 6.60 (H-3, s, 1H), 3.83 (OCH_3-1, s, 3H), 3.61 (OCH_3-10, s, 3H), 3.07 – 2.97 (H-4α, H-5α, H-7α, m, 3H), 2.92 (H-6a, dd, *J* = 13.8, 4.1 Hz, 1H), 2.64 – 2.51 (H-4β, H-7β, H-5β, m, 3H), 2.49 (NCH_3, s, 3H). ^{13}C NMR (101 MHz, $CDCl_3$) δ 156.3, 150.0, 148.5, 144.1, 142.8, 130.0, 129.7, 129.6 (2C), 128.4, 127.8, 126.9, 125.6, 119.9, 119.1 (2C), 114.4, 112.3, 62.5, 60.5, 56.3, 53.4, 43.9, 33.9, 28.9. Calcd. for $C_{25}H_{24}ClNO_4+H^+$: 438.1467. Found: 438.1471.

(S)-9-(4-bromophenoxy)-1,10-dimethoxy-6-methyl-5,6,6a,7-tetrahydro-4H-dibenzo[*de,g*]quinolin-2-ol (**6u**). Light brown solid; mp: 129-130 °C. ^{1}H NMR (400 MHz, $CDCl_3$) δ 8.05 (H-11, s, 1H), 7.41 (OPh(H-3, H-5), d, J = 8.6 Hz, 2H), 6.88 (OPh(H-2, H-6), d, J = 8.6 Hz, 2H), 6.83 (H-8, s, 1H), 6.67 (H-3, s, 1H), 3.84 (OCH_3-1, s, 3H), 3.64 (OCH_3-10, H-4α, s, 4H), 3.42 (H-5α, dd, J = 11.8, 5.8 Hz, 1H), 3.33 – 3.20 (H-7α, m, 1H), 3.03 – 2.90 (H-6a, H-4β, H-7β, m, 3H), 2.81 (NCH_3, s, 3H), 2.74 (H-5β, d, J = 13.4 Hz, 1H). ^{13}C NMR (101 MHz, $CDCl_3$) δ 156.6, 150.4, 149.7, 144.6, 143.5, 132.7 (2C), 127.8, 127.3, 125.9, 121.9, 119.9, 119.6 (2C), 115.6, 114.5, 112.4, 62.5, 60.7, 56.3, 53.2, 42.2, 32.5, 26.7. Calcd. for $C_{25}H_{24}BrNO_4$+H^+: 482.0961. Found: 482.0967.

(S)-9-(4-iodophenoxy)-1,10-dimethoxy-6-methyl-5,6,6a,7-tetrahydro-4H-dibenzo[*de,g*]quinolin-2-ol (**6v**). Beige solid; mp: 109-112 °C. ^{1}H NMR (400 MHz, $CDCl_3$) δ 8.06 (H-11, s, 1H), 7.59 (OPh(H-3, H-5), d, J = 8.8 Hz, 1H), 6.84 (H-8, s, 1H), 6.78 (OPh(H-2, H-6), d, J = 8.8 Hz, 2H), 6.64 (H-3, s, 1H), 3.84 (OCH_3-1, s, 3H), 3.63 (OCH_3-10, s, 3H), 3.12 – 3.01 (H-4α, H-5α, H-7α, m, 3H), 2.94 (H-6a, dd, J = 13.8, 4.1 Hz, 1H), 2.66 – 2.54 (H-4β, H-7β, H-5β, m, 3H), 2.52 (NCH_3, s, 3H). ^{13}C NMR (101 MHz, $CDCl_3$) δ 157.8, 150.1, 148.5, 143.8, 142.8, 138.6, 130.0, 129.7, 128.6, 126.8, 125.6, 120.1, 119.9, 114.4, 112.3, 85.5, 62.5, 60.6, 56.3, 53.4, 43.9, 33.9, 28.8. Calcd. for $C_{25}H_{24}INO_4$+H^+: 530.0823. Found: 530.0833.

(S)-4-((2-hydroxy-1,10-dimethoxy-6-methyl-5,6,6a,7-tetrahydro-4H-dibenzo[*de,g*]quinolin-9-yl)oxy)benzaldehyde (**6w**). Light brown solid; mp: 130-131 °C. ^{1}H NMR (400 MHz, $CDCl_3$) δ 9.90 (OPh(CHO), s, 1H), 8.10 (H-11, s, 1H), 7.83 (OPh(H-3, H-5), d, J = 8.3 Hz, 2H), 7.05 (OPh(H-2, H-6), d, J = 8.3 Hz, 2H), 6.97 (H-8, s, 1H), 6.65 (H-3, s, 1H), 3.80 (OCH_3-1, s, 3H), 3.64 (OCH_3-10, s, 3H), 3.16 – 3.06 (H-4α, H-5α, H-7α, m, 3H), 2.99 (H-6a, dd, J = 13.8, 3.9 Hz, 1H), 2.68 – 2.57 (H-4β, H-7β, H-5β, m, 3H), 2.55 (NCH_3, s, 3H). ^{13}C NMR (101 MHz, $CDCl_3$) δ 190.9, 163.4, 150.5, 148.7, 143.0, 142.1, 132.0, 131.2, 129.8, 129.8, 129.7, 126.5, 125.5, 121.6, 116.7, 114.7, 112.5, 62.3, 60.6, 56.2, 53.3, 43.6, 33.7, 28.5. Calcd. for $C_{26}H_{25}NO_5$+H^+: 432.1805. Found: 432.1811.

(S)-1,10-dimethoxy-6-methyl-9-(4-(trifluoromethoxy)phenoxy)-5,6,6a,7-tetrahydro-4H-dibenzo[*de,g*]quinolin-2-ol (**6x**). Beige solid; mp: 84-88 °C. ^{1}H NMR (400 MHz, $CDCl_3$) δ 8.07 (H-11, s, 1H), 7.17 (OPh(H-2, H-6), d, J = 8.6 Hz, 2H), 7.00 (OPh(H-3, H-5), d, J = 9.1

Hz, 2H), 6.87 (H-8, s, 1H), 6.67 (H-3, s, 1H), 3.86 (OCH_3-1, s, 3H), 3.64 (OCH_3-10, s, 3H), 3.14 – 3.04 (H-4α, H-5α, H-7α, m, 3H), 2.96 (H-6a, dd, J = 13.9, 4.0 Hz, 1H), 2.69 – 2.56 (H-4β, H-7β, H-5β, m, 3H), 2.55 (NCH_3, s, 3H). ^{13}C NMR (101 MHz, $CDCl_3$) δ 156.3, 150.3, 148.5, 144.3 (d, $^4J_{C\text{-}F}$ = 1.9 Hz144.0, 142.8, 129.9, 129.6, 128.6, 125.6, 122.6 (2C), 120.7 (d, $^1J_{C\text{-}F}$ = 256.5 Hz), 120.3, 118.4 (3C), 114.4, 112.3, 62.5, 60.6, 56.3, 53.4, 43.8, 33.8, 28.7. ^{19}F NMR (376 MHz, $CDCl_3$) δ -58.24 (s, 3F). Calcd. for $C_{26}H_{24}F_3NO_5$+H^+: 488.1679. Found: 488.1688.

(*S*)-4-((2-hydroxy-1,10-dimethoxy-6-methyl-5,6,6a,7-tetrahydro-4*H*-dibenzo[*de,g*]quinolin-9-yl)oxy)benzoate (**6y**). Light brown solid; mp: 108-110 °C. ^1H NMR (400 MHz, $CDCl_3$) δ 8.08 (H-11, s, 1H), 8.00 (OPh(H-3, H-5), d, J = 8.9 Hz, 2H), 6.99 (OPh(H-2, H-6), d, J = 8.9 Hz, 2H), 6.94 (H-8, s, 1H), 6.66 (H-3, s, 1H), 3.89 (OCH_3-1, s, 3H), 3.82 (OPh(CO_2CH_3), s, 3H), 3.64 (OCH_3-10, s, 3H), 3.11 – 3.00 (H-4α, H-5α, H-7α, m, 3H), 2.97 (H-6a, dd, J = 13.8, 4.0 Hz, 1H), 2.68 – 2.54 (H-4β, H-7β, H-5β, m, 3H), 2.52 (NCH_3, s, 3H). ^{13}C NMR (101 MHz, $CDCl_3$) δ 166.8, 162.1, 150.4, 148.4, 142.8, 142.7, 131.7 (2C), 130.1, 129.9, 129.3, 127.1, 125.5, 124.3, 121.3, 116.4 (2C), 114.5, 112.4, 62.5, 60.6, 56.3, 53.5, 52.1, 44.0, 34.0, 28.9. Calcd. for $C_{27}H_{27}NO_6$+H^+: 462.1911. Found: 462.1922.

(*S*)-1,10-dimethoxy-6-methyl-9-(4-(trifluoromethyl)phenoxy)-5,6,6a,7-tetrahydro-4*H*-dibenzo[*de,g*]quinolin-2-ol (**6z**). Pale yellow solid; mp: 100-102 °C. ^1H NMR (400 MHz, $CDCl_3$) δ 8.09 (H-11, s, 1H), 7.56 (OPh(H-3, H-5), d, J = 8.4 Hz, 2H), 7.04 (OPh(H-2, H-6), d, J = 8.3 Hz, 2H), 6.94 (H-8, s, 1H), 6.66 (H-3, s, 1H), 3.83 (OCH_3-1, s, 3H), 3.65 (OCH_3-10, s, 3H), 3.12 – 3.04 (H-4α, H-5α, H-7α, m, 3H), 2.98 (H-6a, dd, J = 13.9, 4.1 Hz, 1H), 2.67 – 2.55 (H-4β, H-7β, H-5β, m, 3H), 2.54 (NCH_3, s, 3H). ^{13}C NMR (101 MHz, $CDCl_3$) δ 160.8, 150.5, 148.5, 142.9, 142.7, 130.0, 129.8, 129.4, 127.1 (q, $^3J_{C\text{-}F}$ = 3.8 Hz, 2C), 126.9, 125.5, 124.6 (q, $^2J_{C\text{-}F}$ = 32.7 Hz), 124.4 (q, $^1J_{C\text{-}F}$ = 271.5 Hz), 121.2, 116.8 (2C), 114.6, 112.4, 62.5, 60.6, 56.3, 53.4, 43.9, 33.9, 28.8. ^{19}F NMR (376 MHz, $CDCl_3$) δ -61.61 (s, 3F). Calcd. for $C_{26}H_{24}F_3NO_4$+H^+: 472.1730. Found: 472.1741.

(*S*)-1,10-dimethoxy-6-methyl-9-(4-nitrophenoxy)-5,6,6a,7-tetrahydro-4*H*-dibenzo[*de,g*]quinolin-2-ol (**6aa**). Brown solid; mp: 124-126 °C. ^1H NMR (400 MHz, $CDCl_3$) δ 8.20 (OPh(H-3, H-5), d, J = 9.2 Hz, 1H), 8.10 (H-11, s, 1H), 7.03 – 6.98 (OPh(H-2, H-6), H-8, m, 2H), 6.68 (H-3, s, 1H), 3.81 (OCH_3-1, s, 3H), 3.65 (OCH_3-10, s, 3H), 3.18

– 3.02 (H-4α, H-5α, H-7α, m, 3H), 3.01 (H-6a, dd, J = 13.8, 4.0 Hz, 1H), 2.72 – 2.56 (H-4β, H-7β, H-5β, m, 3H), 2.55 (NCH_3, s, 3H). ^{13}C NMR (101 MHz, $CDCl_3$) δ 163.5, 150.5, 148.4, 142.9, 142.6, 141.7, 130.3, 130.2, 130.0, 127.0, 126.0 (2C), 125.3, 121.8, 116.2 (2C), 114.8, 112.4, 62.5, 60.7, 56.2, 53.5, 43.9, 33.9, 28.8. Calcd. for $C_{25}H_{24}N_2O_6$+H^+: 449.1707. Found: 449.1711.

(*S*)-1,10-dimethoxy-6-methyl-9-(4-(methylsulfonyl)phenoxy)-5,6,6a,7-tetrahydro-4*H*-dibenzo[*de,g*]quinolin-2-ol (**6ab**). Taupe solid; mp: 85-87 °C. ^{1}H NMR (400 MHz, $CDCl_3$) δ 8.09 (H-11, s, 1H), 7.87 (OPh(H-3, H-5), d, J = 8.9 Hz, 2H), 7.07 (OPh(H-2, H-6), d, J = 8.9 Hz, 2H), 6.97 (H-8, s, 1H), 6.68 (H-3, s, 1H), 3.81 (OCH_3-1, s, 3H), 3.64 (OCH_3-10, s, 3H), 3.15 – 3.05 (H-4α, H-5α, H-7α, m, 3H), 3.05 (OPh(SO_2CH_3), s, 3H), 2.99 (H-6a, dd, J = 13.8, 4.0 Hz, 1H), 2.70 – 2.54 (H-4β, H-7β, H-5β, m, 3H), 2.53 (NCH_3, s, 3H). ^{13}C NMR (101 MHz, $CDCl_3$) δ 162.7, 150.5, 148.4, 142.9, 141.9, 133.8, 130.2, 130.0, 130.0, 129.7 (2C), 127.1, 125.4, 121.7, 116.8 (2C), 114.7, 112.4, 62.4, 60.6, 56.2, 53.4, 44.9, 43.9, 33.9, 28.9. Calcd. for $C_{26}H_{27}NO_6S$+H^+: 482.1632. Found: 482.1633.

(*S*)-1,10-dimethoxy-6-methyl-9-(naphthalen-1-yloxy)-5,6,6a,7-tetrahydro-4H-dibenzo[*de,g*]quinolin-2-ol (**6ac**). Brown solid; mp: 115-117 °C. ^{1}H NMR (400 MHz, $CDCl_3$) δ 8.10 (H-11, s, 1H), 6.87 (H-8, s, 1H), 6.67 (H-3, s, 1H), 3.90 (OCH_3-1, s, 3H), 3.67 (OCH_3-10, s, 3H), 3.13 – 2.99 (H-4α, H-5α, H-7α, m, 3H), 2.93 (H-6a, dd, J = 13.9, 4.0 Hz, 1H), 2.69 – 2.55 (H-4β, H-7β, H-5β, m, 3H), 2.50 (NCH_3, s, 3H). 7.84 (t, J = 8.1 Hz, 2H), 7.72 (d, J = 8.1 Hz, 1H), 7.48 – 7.36 (m, 2H), 7.34 (dd, J = 6.6, 2.6 Hz, 2H). ^{13}C NMR (101 MHz, $CDCl_3$) δ 155.3, 150.1, 148.4, 144.9, 142.7, 134.5, 130.3, 130.1, 129.9, 129.7, 128.0, 127.9, 127.3, 127.0, 126.6, 125.7, 124.7, 119.7 (2C), 114.2, 113.4, 112.2, 62.6, 60.6, 56.4, 53.5, 44.0, 34.0, 28.9. Calcd. for $C_{29}H_{27}NO_4$+H^+: 454.2013. Found: 454.2021.

(*S*)-1,10-dimethoxy-6-methyl-9-(naphthalen-2-yloxy)-5,6,6a,7-tetrahydro-4*H*-dibenzo[*de,g*]quinolin-2-ol (**6ad**). Brown solid; mp: 122-124 °C. ^{1}H NMR (400 MHz, $CDCl_3$) 8.11 (H-11, s, 1H), 6.79 (H-8, s, 1H), 6.66 (H-3, s, 1H), 3.91 (OCH_3-1, s, 3H), 3.67 (OCH_3-10, s, 3H), 3.18 – 2.98 (H-4α, H-5α, H-7α, m, 3H), 2.88 (H-6a, dd, J = 13.9, 4.0 Hz, 1H), 2.70 – 2.53 (H-4β, H-7β, H-5β, m, 3H), 2.49 (NCH_3, s, 3H). 8.35 – 8.29 (m, 1H), 7.92 – 7.83 (m, 1H), 7.61 (d, J = 8.2 Hz, 1H), 7.55 – 7.50 (m, 2H), 7.39 (t, J = 7.9 Hz, 1H), 6.92 (d, J = 7.5 Hz, 1H). ^{13}C NMR (101 MHz, $CDCl_3$) δ 153.4, 149.9, 148.4, 145.5, 142.7, 135.0,

129.9, 129.7, 127.8 (2C), 126.7, 126.6, 126.0, 125.9, 125.7, 123.1, 122.3, 119.4, 114.2, 112.4, 112.2, 62.5, 60.6, 56.5, 53.5, 43.9, 33.9, 28.8. Calcd. for $C_{29}H_{27}NO_4+H^+$: 454.2013. Found: 454.2018.

(S)-1,10-dimethoxy-6-methyl-9-phenoxy-2-(p-tolyloxy)-5,6,6a,7-tetrahydro-4H dibenzo[de,g]quinoline (**6ae**). Green solid. ^{1}H NMR (400 MHz, $CDCl_3$) δ 8.15 (H-11, s, 1H), 7.36 – 7.31 (OPh(H-3, H-5), m, 2H), 7.14 (OTolyl(H-3, H-5), d, J = 8.6 Hz, 2H), 7.09 (OPh(H-4), t, J = 7.4 Hz, 1H), 7.04 (OTolyl(H-2, H-6), d, J = 7.7 Hz, 1H), 6.94 (OPh(H-2, H-6), d, J = 8.6 Hz, 2H), 6.83 (H-8, s, 1H), 6.65 (H-3, s, 1H), 3.87 (OCH_3-1, s, 3H), 3.74 (OCH_3-10, s, 3H), 3.14 – 3.03 (H-4α, H-5α, H-7α, m, 3H), 3.01 (H-4β, t, J = 5.4 Hz, 1H), 2.95 (H-6a, dd, J = 13.9, 4.2 Hz, 1H), 2.63 – 2.60 (H-7β, m, 1H), 2.59 – 2.56 (H-5β, m, 1H), 2.52 (NCH_3, s, 3H), 2.34 (CH_3(OTolyl), s, 3H).

(*S*)-1,2,10-trimethoxy-6-methyl-9-phenoxy-5,6,6a,7-tetrahydro-4*H*-dibenzo[*de*,*g*]quinoline (**7a**). Yellow solid; mp: 65-66 °C. ^{1}H NMR (400 MHz, $CDCl_3$) δ 8.20 (H-11, s, 1H), 7.33 (OPh(H-3, H-5), t, *J* = 7.7 Hz, 2H), 7.08 (OPh(H-4), t, *J* = 7.3 Hz, 1H), 7.04 (OPh(H-2, H-6), d, *J* = 8.4 Hz, 2H), 6.82 (H-8, s, 1H), 6.62 (H-3, s, 1H), 3.89 (OCH_3-1, s, 3H), 3.87 (OCH_3-2, s, 3H), 3.70 (OCH_3-10, s, 3H), 3.20 – 3.10 (H-4α, m, 1H), 3.07 – 2.99 (H-5α, H-7α, m, 2H), 2.93 (H-6a, dd, *J* = 13.7, 4.1, 1H), 2.68 (H-4β, dd, *J* = 16.2, 3.7, 1H), 2.57 – 2.52 (H-7β, m, 1H), 2.50 (NCH_3, s, 3H), 2.48 (H-5β, d, *J* = 4.8, 1H). ^{13}C NMR (101 MHz, $CDCl_3$) δ 157.7, 152.1, 149.8, 144.7, 144.4, 129.7 (2C), 129.7, 129.1, 128.2, 127.5, 126.7, 122.9, 119.4, 118.0 (2C), 113.1, 111.1, 62.5, 60.4, 56.2, 55.9, 53.4, 44.0, 34.2, 29.3. Calcd. for $C_{26}H_{27}NO_4+H^+$: 418.2013. Found: 418.2019.

(*S*)-3-bromo-1,10-dimethoxy-6-methyl-9-phenoxy-5,6,6a,7-tetrahydro-4*H*-dibenzo[*de,g*]quinolin-2-ol (**9a**). Brown solid; mp: 74-75 °C. ^{1}H NMR (400 MHz, $CDCl_3$) δ 8.02 (H-11, s, 1H), 7.35 (OPh(H-3, H-5), t, *J* = 7.7 Hz, 2H), 7.11 (OPh(H-4), t, *J* = 7.4 Hz, 1H), 7.05 (OPh(H-2, H-6), d, *J* = 8.0 Hz, 2H), 6.82 (H-8, s, 1H), 3.89 (OCH_3-1, s, 3H), 3.68 (OCH_3-10, s, 3H), 3.11 (H-4α, dd, *J* = 11.7, 6.1, 1H), 3.03 (H-5α, dd, *J* = 14.0, 3.9, 1H), 2.97 – 2.89 (H-6a, H-7α, m, 2H), 2.81 (H-4β, dd, *J* = 17.2, 4.0, 1H), 2.52 – 2.44 (NCH_3, H-7β, H-5β, m, 5H). ^{13}C NMR (101 MHz, $CDCl_3$) δ 157.4, 150.1, 146.0, 145.1, 142.9, 129.8, 129.7, 129.6, 128.6, 127.2, 125.3, 123.2, 119.4, 118.3, 112.3, 110.5, 62.5, 60.7, 56.4, 53.3, 43.7, 33.8, 30.1. Calcd. for $C_{25}H_{24}BrNO_4+H^+$: 482.0961. Found: 482.0966.

(*S*)-3-bromo-9-(4-(*tert*-butyl)phenoxy)-1,10-dimethoxy-6-methyl-5,6,6a,7-tetrahydro-4*H*-dibenzo[*de,g*]quinolin-2-ol (**9b**). Taupe solid; mp: 107-108 °C. ^{1}H NMR (400 MHz, $CDCl_3$) δ 8.00 (H-11, s, 1H), 7.35 (OPh(H-3, H-5), d, J = 7.0 Hz, 2H), 6.97 (OPh(H-2, H-6), d, J = 7.0 Hz, 2H), 6.80 (H-8, s, 1H), 3.89 (OCH_3-1, s, 3H), 3.67 (OCH_3-10, s, 3H), 3.14 (H-4α, dd, J = 11.7, 6.1, 1H), 3.05 (H-5α, dd, J = 13.4, 4.1, 1H), 2.97 – 2.88 (H-6a, H-7α, m, 2H), 2.82 (H-4β, dd, J = 17.1, 4.0, 1H), 2.56 – 2.45 (NCH_3, H-7β, H-5β, m, 5H), 1.33 (OPh(*t*-Bu), s, 9H). ^{13}C NMR (101 MHz, $CDCl_3$) δ 154.8, 150.0, 146.1, 146.0, 145.6, 142.9, 129.6, 129.4, 128.3, 126.8, 126.6 (2C), 125.3, 119.0, 117.8 (2C), 112.3, 110.4, 62.4, 60.7, 56.4, 53.1, 43.6, 34.4, 33.7, 31.6 (3C), 29.9. Calcd. for $C_{29}H_{32}BrNO_4$+H^+: 538.1587. Found: 538.1591.

(*S*)-3-bromo-9-(4-fluorophenoxy)-1,10-dimethoxy-6-methyl-5,6,6a,7-tetrahydro-4*H*-dibenzo[*de,g*]quinolin-2-ol (**9c**). Taupe solid; mp: 81-83 °C. ^{1}H NMR (400 MHz, $CDCl_3$) δ 8.01 (H-11, s, 1H), 7.06 – 6.98 (OPh(H-2, H-3, H-5, H-6), m, 8H), 6.76 (H-8, s, 1H), 3.89 (OCH_3-1, s, 3H), 3.67 (OCH_3-10, s, 3H), 3.09 (H-4α, dd, J = 11.8, 6.2, 1H), 2.99 (H-5α, d, J = 11.2, 1H), 2.95 – 2.87 (H-6a, H-7α, m, 2H), 2.81 (H-4β, d, J = 14.9, 1H), 2.54 – 2.44 (NCH_3, H-7β, H-5β, m, 2H). ^{13}C NMR (101 MHz, $CDCl_3$) δ 158.8 (d, J = 241.2 Hz), 153.2 (d, J = 2.5 Hz), 149.8, 145.9, 145.5, 142.9, 129.8, 129.6, 128.7, 127.2, 125.2, 119.8 (d, J = 8.3 Hz), 118.75, 116.32 (d, J = 23.4 Hz), 112.3, 110.6, 62.6, 60.7, 56.4, 53.3, 43.9, 33.9, 30.2. ^{19}F NMR (376 MHz, $CDCl_3$) δ -120.50 (s, 1F). Calcd. for $C_{25}H_{23}BrFNO_4$+H^+: 500.0867. Found: 500.0877.

(*S*)-3-bromo-1,10-dimethoxy-6-methyl-9-(4-(trifluoromethyl)phenoxy)-5,6,6a,7-tetrahydro-4*H*-dibenzo[*de,g*]quinolin-2-ol (**9d**). Beige solid; mp: 104-105 °C. ^{1}H NMR (400 MHz, $CDCl_3$) δ 8.05 (H-11, s, 1H), 7.57 (OPh(H-3, H-5), d, J = 8.3 Hz, 2H), 7.04 (OPh(H-2, H-6), d, J = 8.3 Hz, 2H), 6.94 (H-8, s, 1H), 3.84 (OCH_3-1, s, 3H), 3.69 (OCH_3-10, s, 3H), 3.11 (H-4α, dd, J = 11.7, 6.1 Hz, 1H), 3.06 – 2.98 (H-5α, H-7α, m, 2H), 2.98 – 2.94 (H-6a, m, 1H), 2.94 – 2.90 (H-4β, m, 1H), 2.82 (H-7β, d, J = 13.0 Hz, 1H), 2.52 (NCH_3, s, 3H), 2.46 (H-5β, dd, J = 11.8, 3.8 Hz, 1H). ^{13}C NMR (101 MHz, $CDCl_3$) δ 160.7, 150.5, 146.0, 143.0, 143.0, 129.9 (2C), 128.8, 128.8, 127.1 (q, $^3J_{C\text{-}F}$ = 3.8 Hz, 2C), 125.0, 124.7 (d, $^2J_{C\text{-}F}$ = 32.7 Hz), 124.4 (d, $^1J_{C\text{-}F}$ = 271.4 Hz), 121.1, 116.9 (2C), 112.6, 111.0, 62.6, 60.8, 56.3, 53.3, 43.9, 33.9, 30.3. ^{19}F NMR (376 MHz, $CDCl_3$) δ -61.63 (s, 3F). Calcd. for $C_{26}H_{23}BrF_3NO_4$+H^+: 550.0835. Found: 550.0837.

(*S*)-3-bromo-1,10-dimethoxy-6-methyl-9-(4-(trifluoromethoxy)phenoxy)-5,6,6a,7-tetrahydro-4*H*-dibenzo[*de,g*]quinolin-2-ol (**9e**). Beige solid; mp: 88-89 °C. ^{1}H NMR (400 MHz, $CDCl_3$) δ 8.03 (H-11, s, 1H), 7.17 (OPh(H-2, H-6), d, J = 8.4 Hz, 2H), 7.00 (OPh(H-3, H-5), d, J = 7.9 Hz, 2H), 6.87 (H-8, s, 1H), 3.86 (OCH_3-1, s, 3H), 3.68 (OCH_3-10, s, 3H), 3.11 (H-4α, dd, J = 11.4, 6.1 Hz, 1H), 3.06 – 2.88 (H-5α, H-7α, H-6a, H-4β, m, 4H), 2.81 (H-7β, d, J = 15.4 Hz, 1H), 2.52 (NCH_3, s, 3H), 2.49 – 2.42 (H-5β, m, 1H). ^{13}C NMR (101 MHz, $CDCl_3$) δ 156.2, 150.3, 146.0, 144.3 (d, $^3J_{C-F}$ = 1.4 Hz), 144.2, 142.9, 129.8, 129.8, 128.7, 128.1, 125.1, 122.6 (2C), 120.7 (d, $^1J_{C-F}$ = 256.4 Hz), 120.1, 118.5 (2C), 112.5, 110.8, 62.5, 60.7, 56.4, 53.3, 43.8, 33.9, 30.2. ^{19}F NMR (376 MHz, $CDCl_3$) δ -58.24 (s, 3F). Calcd. for $C_{26}H_{23}BrF_3NO_5+H^+$: 566.0784. Found: 566.0788.

(*S*)-1,10-dimethoxy-6-methyl-9-phenoxy-5,6,6a,7-tetrahydro-4*H*-dibenzo[*de,g*]quinolin-2-yl acetate (**10a**). Brown solid; mp: 73-74 °C. ^{1}H NMR (400 MHz, $CDCl_3$) δ 8.09 (H-11, s, 1H), 7.34 (OPh(H-3, H-5), t, J = 7.3 Hz, 2H), 7.09 (OPh(H-4), t, J = 7.4 Hz, 1H), 7.04 (OPh(H-2, H-6), d, J = 6.4 Hz, 2H), 6.81 (H-8, s, 1H), 6.80 (H-3, s, 1H), 3.87 (OCH_3-1, s, 3H), 3.63 (OCH_3-10, s, 3H), 3.19 – 3.08 (H-4α, m, 1H), 3.08 – 2.98 (H-5α, H-6a, m, 2H), 2.93 (H-7α, dt, J = 13.9, 3.1 Hz, 1H), 2.69 (H-4β, d, J = 16.2 Hz, 1H), 2.55 (H-7β, d, J = 13.9 Hz, 1H), 2.52 – 2.43 (NCH_3, H-5β, m, 4H), 2.37 ($OCOCH_3$, s, 3H). ^{13}C NMR (101 MHz, $CDCl_3$) δ 169.4, 157.4, 149.8, 147.0, 144.8, 143.2, 133.7, 129.7, 129.6 (2C), 129.5, 127.5, 127.4, 123.0, 121.7, 119.2, 118.1 (2C), 112.8, 77.0, 62.6, 60.5, 56.3, 53.0, 43.9, 33.7, 28.8, 20.9. Calcd. for $C_{27}H_{27}NO_5+H^+$: 446.1962. Found: 446.1967.

(*S*)-2-hydroxy-1,10-dimethoxy-6,6,6a-trimethyl-9-phenoxy-5,6,6a,7-tetrahydro-4*H*-dibenzo[*de,g*]quinolin-6-ium (**11a**). Light brown solid; mp: 156-157 °C. ^{1}H NMR (400 MHz, $CDCl_3$) δ 8.05 (H-11, s, 1H), 7.32 (OPh(H-3, H-5), t, J = 7.8 Hz, 2H), 7.08 (OPh(H-4), t, J = 7.4 Hz, 1H), 6.99 (OPh(H-2, H-6), d, J = 8.0 Hz, 2H), 6.90 (H-8, s, 1H), 6.83 (H-3, s, 1H), 4.49 (H-6a, d, J = 13.9 Hz, 1H), 4.27 (H-5α, dd, J = 13.1, 5.8 Hz, 1H), 3.83 (OCH_3-1, s, 3H), 3.80 – 3.72 (H-7α, m, 1H), 3.71 (NCH_3, s, 3H), 3.57 (OCH_3-10, s, 3H), 3.31 (H-4α, t, J = 12.7 Hz, 1H), 3.16 (NCH_3, s, 3H), 3.02 (H-4β, d, J = 13.6 Hz, 1H), 2.90 (H-7β, t, J = 13.6 Hz, 1H), 2.59 (H-5β, s, 1H). ^{13}C NMR (101 MHz, $CDCl_3$) δ 157.0, 151.2, 150.7, 145.3, 144.7, 129.8 (2C), 126.9, 126.9, 124.8, 124.2, 123.3, 119.6, 118.0 (2C), 118.0, 115.0, 112.6,

69.9, 61.7, 60.6, 56.3, 54.3, 49.3, 43.9, 29.1, 23.9. Calcd. for $C_{26}H_{28}NO_4^+$: 418.2013. Found: 418.2020.

(*S*)-1,2,10-trimethoxy-6,6-dimethyl-9-phenoxy-5,6,6a,7-tetrahydro-4*H*-dibenzo[*de,g*]quinolin-6-ium (**11b**). White solid; mp: 155-157 °C. ^{1}H NMR (400 MHz, $CDCl_3$) δ 8.13 (H-11, s, 1H), 7.40 – 7.28 (OPh(H-3, H-5), m, 2H), 7.17 – 7.05 (OPh(H-4), m, 1H), 7.01 (OPh(H-2, H-6), d, *J* = 6.6 Hz, 2H), 6.91 (H-8, s, 1H), 6.78 (H-3, s, 1H), 4.48 (H-6a, d, *J* = 13.6 Hz, 1H), 4.41 (H-5α, dd, *J* = 12.5, 8.0 Hz, 1H), 3.94 (OCH_3-2, s, 3H), 3.86 (OCH_3-1, s, 3H), 3.75 (NCH_3, s, 3H), 3.61 (OCH_3-10, s, 3H), 3.43 – 3.35 (H-7α, m, 1H), 3.22 (NCH_3, s, 3H), 3.17 (s, 1H), 3.09 (H-4β, d, *J* = 17.9 Hz, 1H), 2.91 (H-7β, t, *J* = 13.5 Hz, 1H), 2.79 (H-5β, s, 1H). ^{13}C NMR (101 MHz, $CDCl_3$) δ 156.9, 154.1, 150.5, 146.1, 145.4, 129.8, 127.3, 126.7, 124.5, 124.2, 123.4, 119.3, 118.8, 118.1, 113.2, 111.0, 69.9, 68.0, 61.6, 60.6, 56.1, 56.1, 54.3, 49.3, 43.8, 29.1, 25.5, 24.2. Calcd. for $C_{27}H_{30}NO_4^+$: 432.2169. Found: 432.2171.

(*S*)-2-acetoxy-1,10-dimethoxy-6,6-dimethyl-9-phenoxy-5,6,6a,7-tetrahydro-4*H*-dibenzo[*de,g*]quinolin-6-ium (**11c**). Pale green solid; mp: 168-169 °C. ^{1}H NMR (400 MHz, $CDCl_3$) δ 7.99 (H-11, s, 1H), 7.30 (OPh(H-3, H-5), t, *J* = 7.5 Hz, 2H), 7.05 (OPh(H-4), t, *J* = 7.3 Hz, 1H), 6.97 (OPh(H-2, H-6), d, *J* = 8.7 Hz, 2H), 6.95 (H-8, s, 1H), 6.94 (H-3, s, 1H), 4.58 (H-6a, H-5α, m, 2H), 3.88 (H-7α, dd, *J* = 12.9, 4.9 Hz, 1H), 3.81 (OCH_3-1, s, 3H), 3.71 (NCH_3, s, 3H), 3.64 (OCH_3-10, s, 3H), 3.41 – 3.28 (H-4α, H-4β, m, 2H), 3.26 (NCH_3, s, 3H), 3.11 – 3.01 (H-7β, m, 1H), 2.92 (H-5β, t, *J* = 13.8 Hz, 1H), 2.35 ($OCOCH_3$, s, 3H). ^{13}C NMR (101 MHz, $CDCl_3$) δ 169.1, 156.9, 150.8, 148.8, 145.8, 145.5, 129.9, 128.5, 126.2, 125.5, 125.0, 124.4, 123.5, 122.6, 119.7, 118.2, 112.8, 69.9, 61.0, 61.0, 56.4, 54.6, 44.4, 28.9, 23.9, 20.9. Calcd. for $C_{28}H_{30}NO_5^+$: 460.2118. Found: 460.2121.

(*S*)-9-(4-((benzylamino)methyl)phenoxy)-1,10-dimethoxy-6-methyl-5,6,6a,7-tetrahydro-4*H*-dibenzo[*de,g*]quinolin-2-ol (**12a**). Dark vanilla solid; mp: 98-99 °C. ^{1}H NMR (400 MHz, $CDCl_3$) δ 8.06 (H-11, s, 1H), .80 (H-8, s, 1H), 6.62 (H-3, s, 2H), 3.86 (OCH_3-1, s, 3H), 3.62 (OCH_3-10, s, 3H), 3.12 – 3.04 (H-4α, H-5α, H-7α, m, 3H), 2.92 (H-6a, dd, *J* = 13.8, 3.8 Hz, 2H), 2.64 – 2.53 (H-4β, H-7β, H-5β, m, 3H), 2.51 (NCH_3, s, 3H). 7.33 (d, *J* = 7.0 Hz, 5H), 7.30 (d, *J* = 4.0 Hz, 5H), 7.27 (d, *J* = 3.3 Hz, 2H), 7.00 (d, *J* = 8.2 Hz, 2H), 4.83 (s, 4H). ^{13}C NMR (101 MHz, $CDCl_3$) δ 156.7, 156.4, 149.9, 148.6, 144.8, 142.8, 130.2, 129.8, 129.8,

129.5, 129.1, 128.6, 128.5, 128.3, 127.8, 127.4, 126.9, 126.5, 125.8, 119.4, 118.2, 117.7, 114.3, 112.2, 62.4, 60.5, 56.3, 53.3, 52.8, 52.3, 43.7, 33.8, 28.6. Calcd. for $C_{33}H_{34}N_2O_4$+H^+: 523.2591. Found: 523.2599.

(*S*)-9-(4-((benzylamino)methyl)phenoxy)-1,10-dimethoxy-6-methyl-5,6,6a,7-tetrahydro-4*H*-dibenzo[*de,g*]quinolin-2-ol (**12b**). Dark vanilla solid; mp: 63-65 °C. ^{1}H NMR (400 MHz, $CDCl_3$) δ 8.06 (H-11, s, 1H), 6.80 (H-8, s, 1H), 6.60 (H-3, s, 1H), 3.87 (OCH_3-1, s, 3H), 3.62 (OCH_3-10, s, 3H), 3.04 – 2.96 (H-4α, H-5α, H-7α, m, 3H), 2.91 (H-6a, dd, *J* = 13.8, 3.9 Hz, 1H), 2.64 – 2.52 (H-4β, H-7β, H-5β, m, 3H), 2.49 (NCH_3, s, 3H). 7.30 (d, *J* = 8.3 Hz, 2H), 7.27 (s, 1H), 7.25 (s, 1H), 7.00 (d, *J* = 8.3 Hz, 2H), 6.87 (d, *J* = 8.2 Hz, 2H), 3.79 (s, 3H), 3.78 (s, 2H), 3.77 (s, 2H). ^{13}C NMR (101 MHz, $CDCl_3$) δ 158.8, 156.4, 149.8, 148.5, 144.9, 142.8, 134.9, 132.3, 129.9, 129.7, 129.6, 129.5 (2C), 128.4, 127.8, 127.0, 125.8, 119.3, 118.2, 114.4, 114.1, 113.9 (2C), 112.3, 62.5, 60.4, 56.3, 55.4, 53.4, 52.7, 52.6, 44.0, 34.1, 29.0. Calcd. for $C_{34}H_{36}N_2O_5$+H^+: 553.2697. Found: 553.2701.

(*S*)-1,10-dimethoxy-6-methyl-9-(4-((*p*-tolylamino)methyl)phenoxy)-5,6,6a,7-tetrahydro-4*H*-dibenzo[*de,g*]quinolin-2-ol (**12c**). Dark vanilla solid; mp: 92-94 °C. ^{1}H NMR (400 MHz, $CDCl_3$) δ 8.06 (H-11, s, 1H), 6.82 (H-8, s, 1H), 6.66 (H-3, s, 1H), 3.89 (OCH_3-1, s, 3H), 3.64 (OCH_3-10, s, 3H), 3.07 (H-4α, H-5α, H-7α, m, 3H), 2.93 (H-6a, dd, *J* = 13.8, 3.6 Hz, 1H), 2.69 – 2.55 (H-4β, H-7β, H-5β, m, 3H), 2.52 (NCH_3, s, 3H). 7.34 (d, *J* = 8.1 Hz, 2H), 7.05 – 6.95 (m, 4H), 6.58 (d, *J* = 7.9 Hz, 2H), 4.28 (s, 2H), 2.24 (s, 3H). ^{13}C NMR (101 MHz, $CDCl_3$) δ 156.7, 150.0, 148.4, 146.0, 144.8, 142.7, 134.3, 129.9 (2C), 129.6, 129.0 (2C), 127.8, 126.9, 125.7, 121.8, 119.5, 118.2 (2C), 114.2, 113.1 (2C), 112.2, 108.8, 62.5, 60.6, 56.4, 53.4, 48.3, 43.8, 33.9, 28.8, 20.5. Calcd. for $C_{33}H_{34}N_2O_4$+H^+: 523.2591. Found: 523.2598.

(*S*)-1,10-dimethoxy-6-methyl-9-(4-((naphthalen-2-ylamino)methyl)phenoxy)-5,6,6a,7-tetrahydro-4*H*-dibenzo[*de,g*]quinolin-2-ol (**12d**). Dark vanilla solid; mp: 109-111 °C. ^{1}H NMR (400 MHz, $CDCl_3$) δ 8.06 (H-11, s, 1H), 6.84 (H-8, s, 1H), 6.68 (H-3, s, 1H), 3.89 (OCH_3-1, s, 3H), 3.64 (OCH_3-10, s, 3H), 3.07 (H-4α, H-5α, H-7α, m, 3H), 2.94 (H-6a, dd, *J* = 13.8, 3.2 Hz, 1H), 2.62 (H-4β, H-7β, H-5β, m, 3H), 2.52 (NCH_3, s, 3H). 7.69 – 7.65 (m, 2H), 7.63 – 7.59 (m, 2H), 7.38 (t, *J* = 7.3 Hz, 3H), 7.20 (t, *J* = 7.6 Hz, 1H), 7.03 (d, *J* = 7.8 Hz, 2H), 6.92 (d, *J* = 8.9 Hz, 1H), 4.41 (s, 2H), ^{13}C NMR (101 MHz, $CDCl_3$) δ 156.9, 150.1,

148.4, 145.9, 144.7, 142.7, 135.3, 133.8, 130.0, 129.6, 129.3, 129.1, 127.9, 127.8, 126.5, 126.1, 125.9, 125.6, 122.6, 122.2, 119.7, 118.4, 118.2, 118.0, 114.2, 112.2, 108.7, 104.8, 62.5, 60.6, 56.4, 53.4, 48.0, 43.8, 33.9, 28.8. Calcd. for $C_{36}H_{34}N_2O_4+H^+$: 559.2591. Found: 559.2601.

3.- ^{1}H NMR, ^{13}C NMR and ^{19}F NMR spectra of all compounds

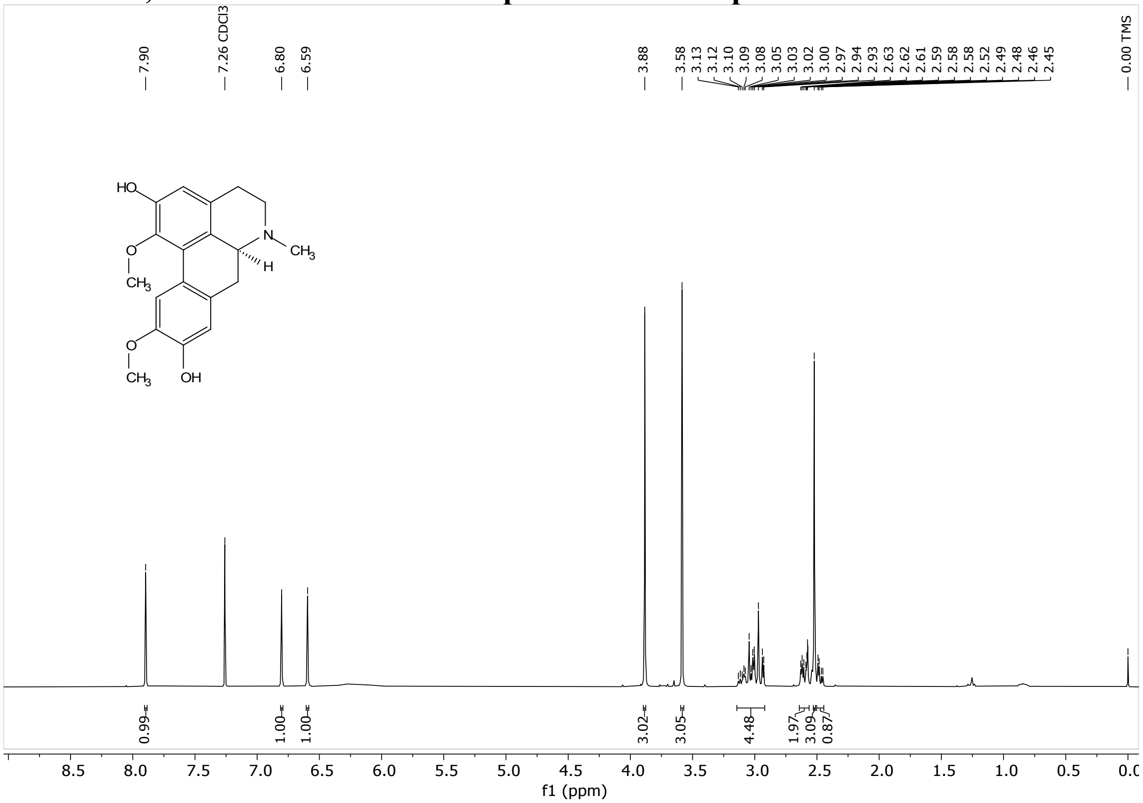

Figure S1. ^{1}H-NMR spectrum of (+)-boldine, **1**-$CHCl_3$ ($CDCl_3$, 400 MHz).

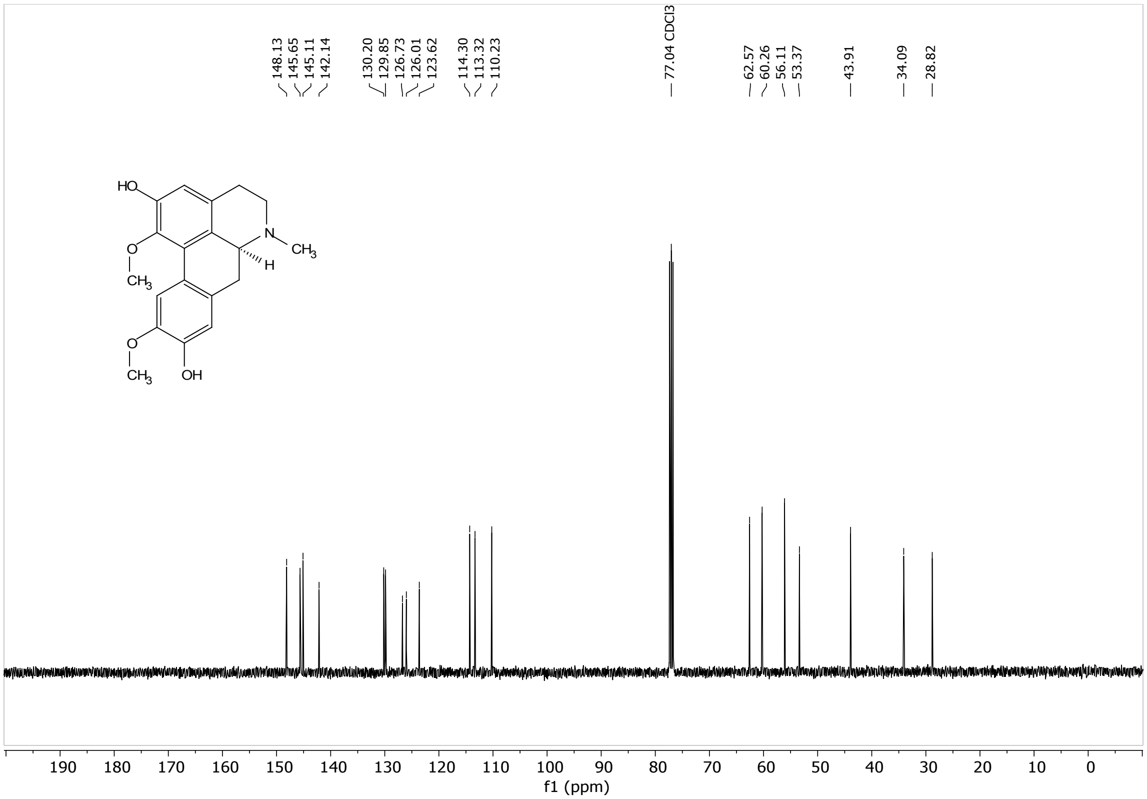

Figure S2. ^{13}C-NMR spectrum of (+)-boldine, **1**-$CHCl_3$ ($CDCl_3$, 101 MHz).

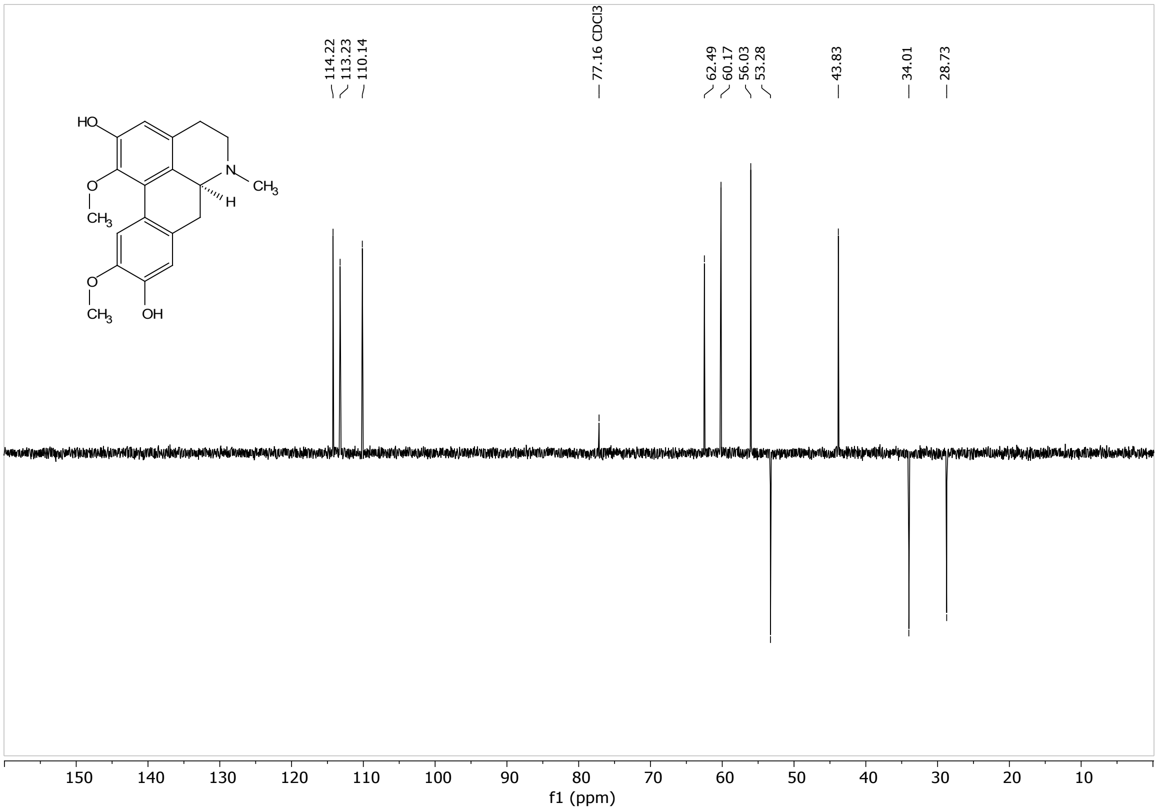

Figure S3. DEPT-135 NMR spectrum of (+)-boldine, **1**-$CHCl_3$ ($CDCl_3$, 101 MHz).

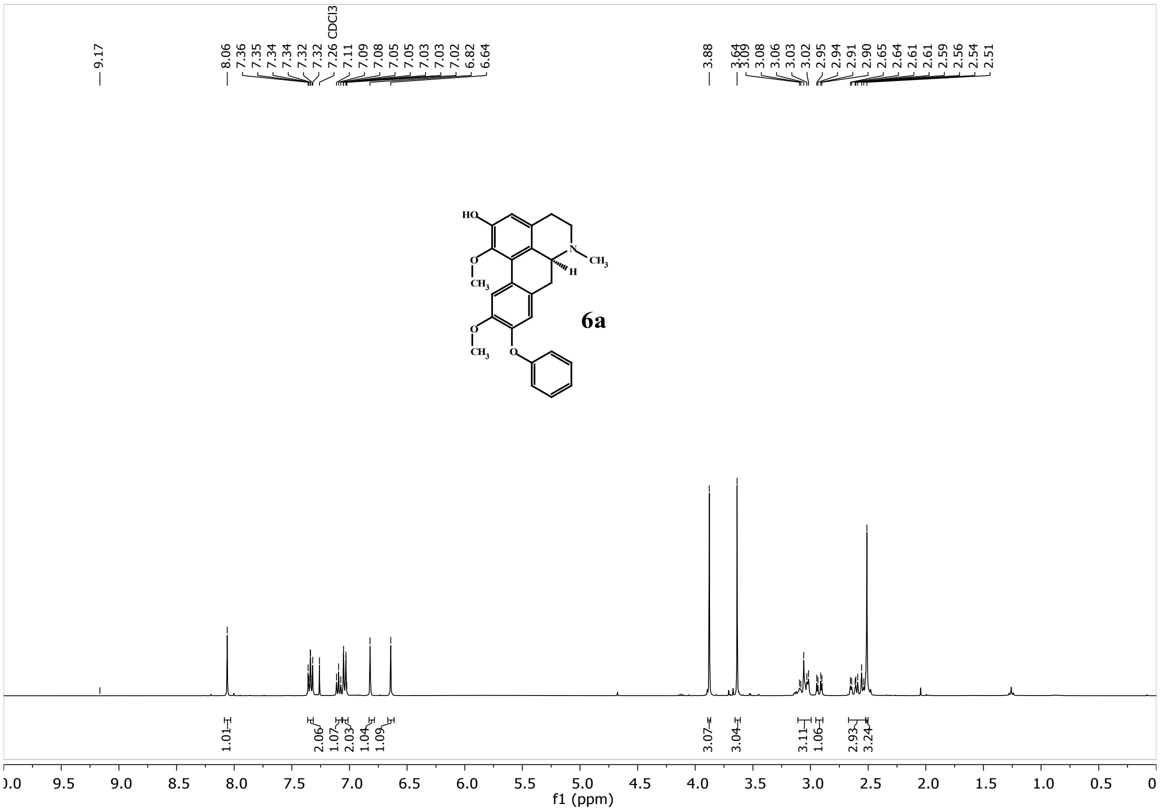

Figure S4. ^1H-NMR spectrum of **6a** ($CDCl_3$, 400 MHz).

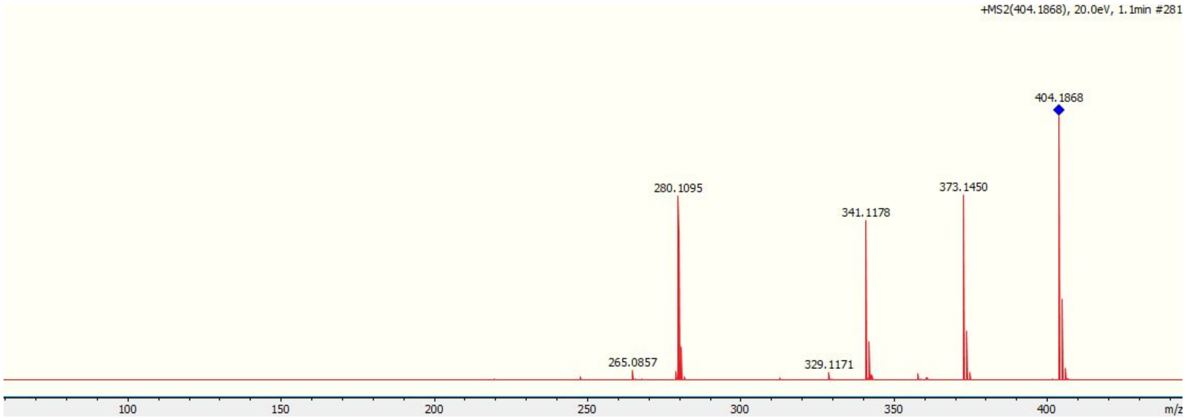

Figure S5. HR-MS of **6a**.

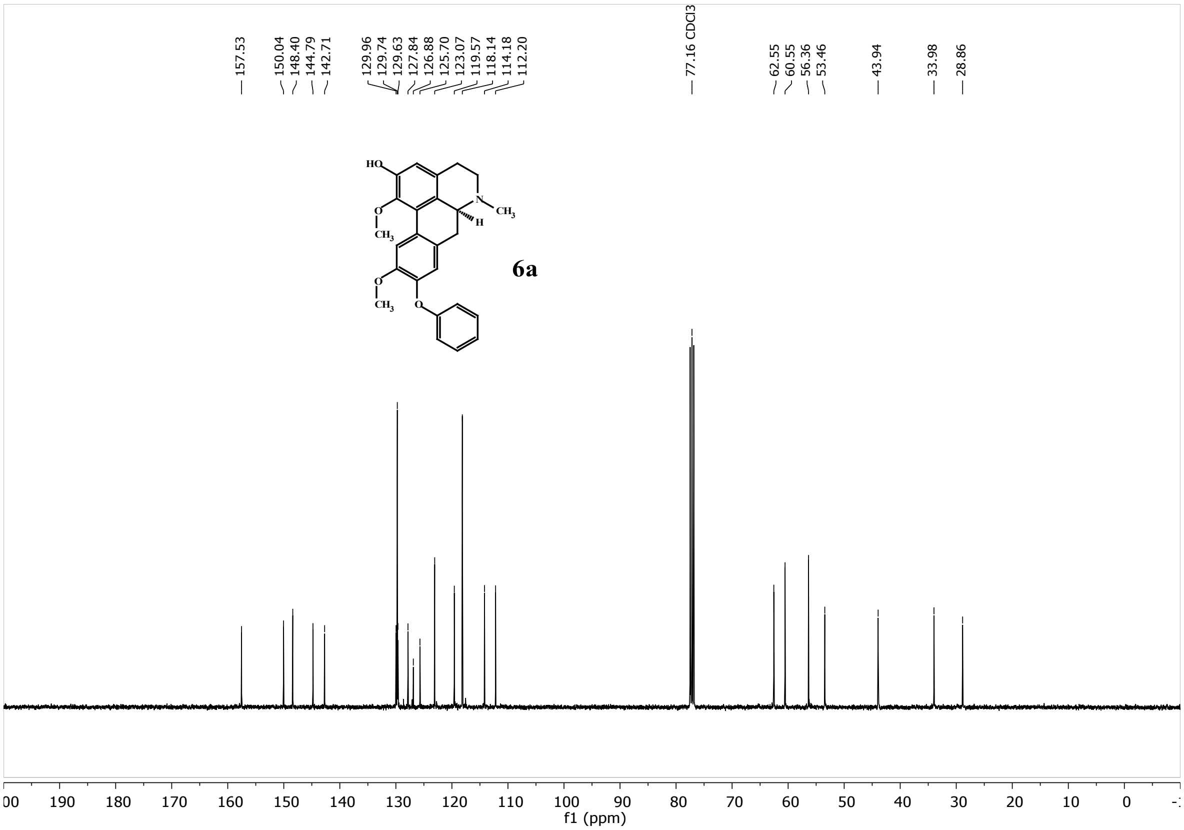

Figure S6. ^{13}C-NMR spectrum of **6a** ($CDCl_3$, 101 MHz).

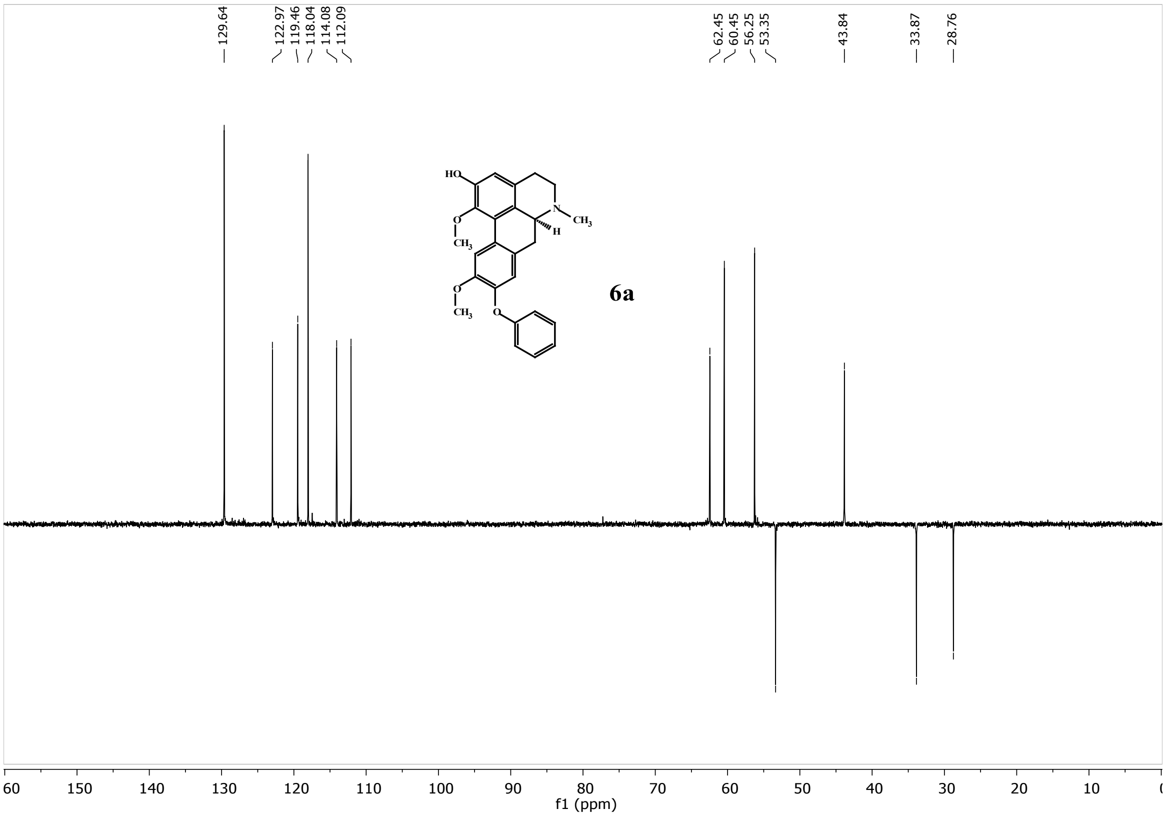

Figure S7. DEPT spectrum of **6a** ($CDCl_3$, 101 MHz).

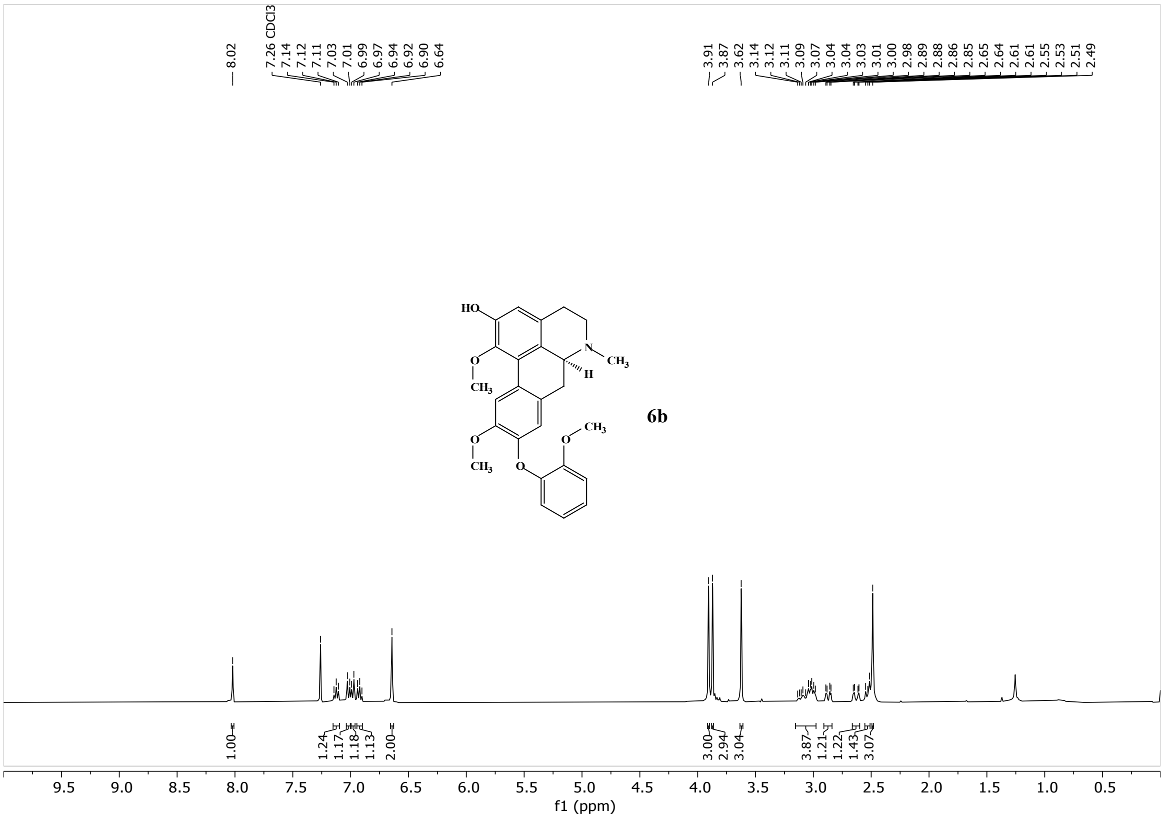

Figure S8. ^{1}H-NMR spectrum of **6b** ($CDCl_3$, 400 MHz).

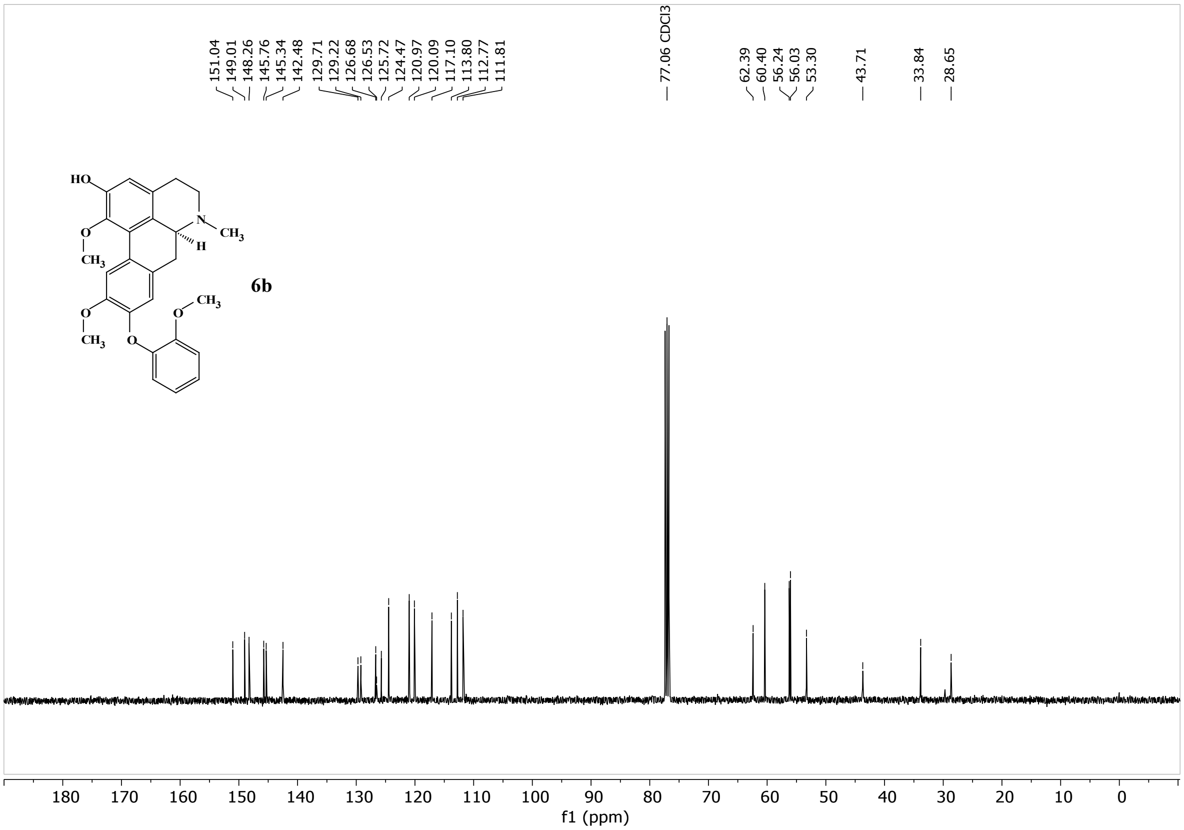

Figure S9. ^{13}C-NMR spectrum of **6b** ($CDCl_3$, 101 MHz).

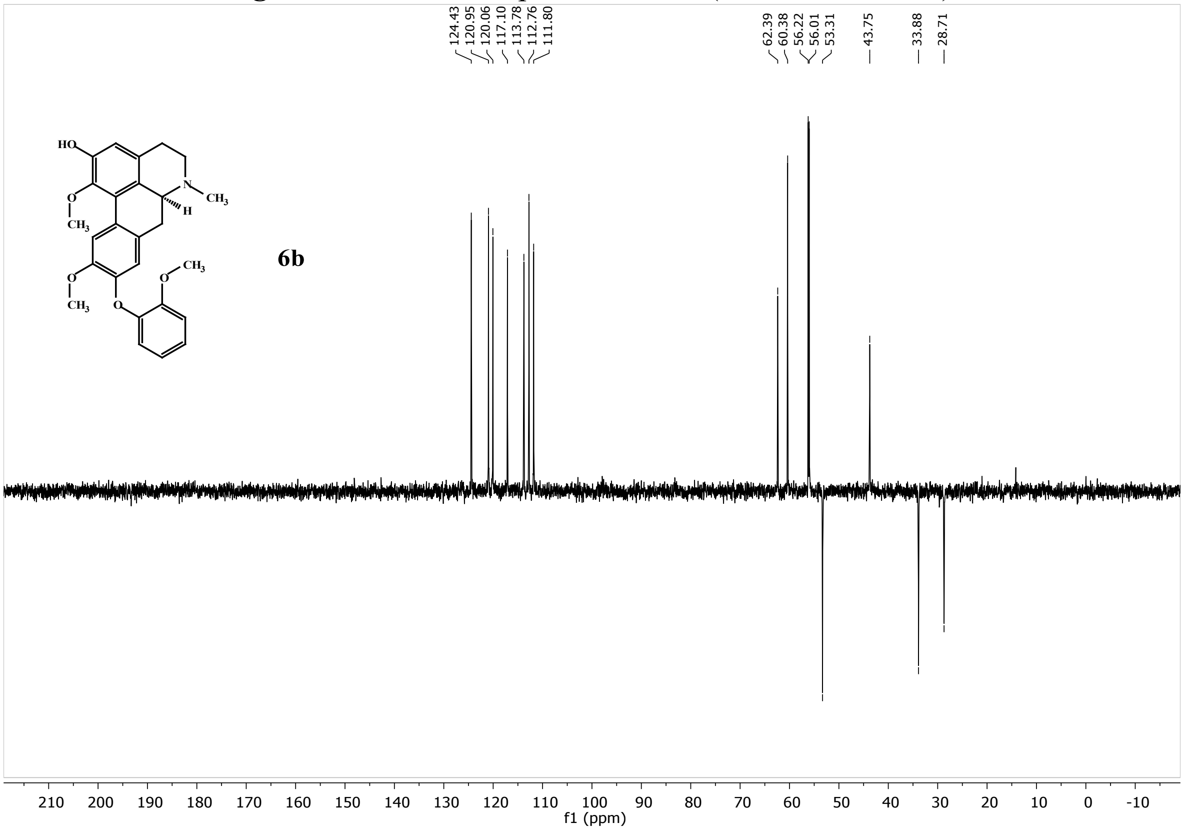

Figure S10. DEPT spectrum of **6b** ($CDCl_3$, 50 MHz).

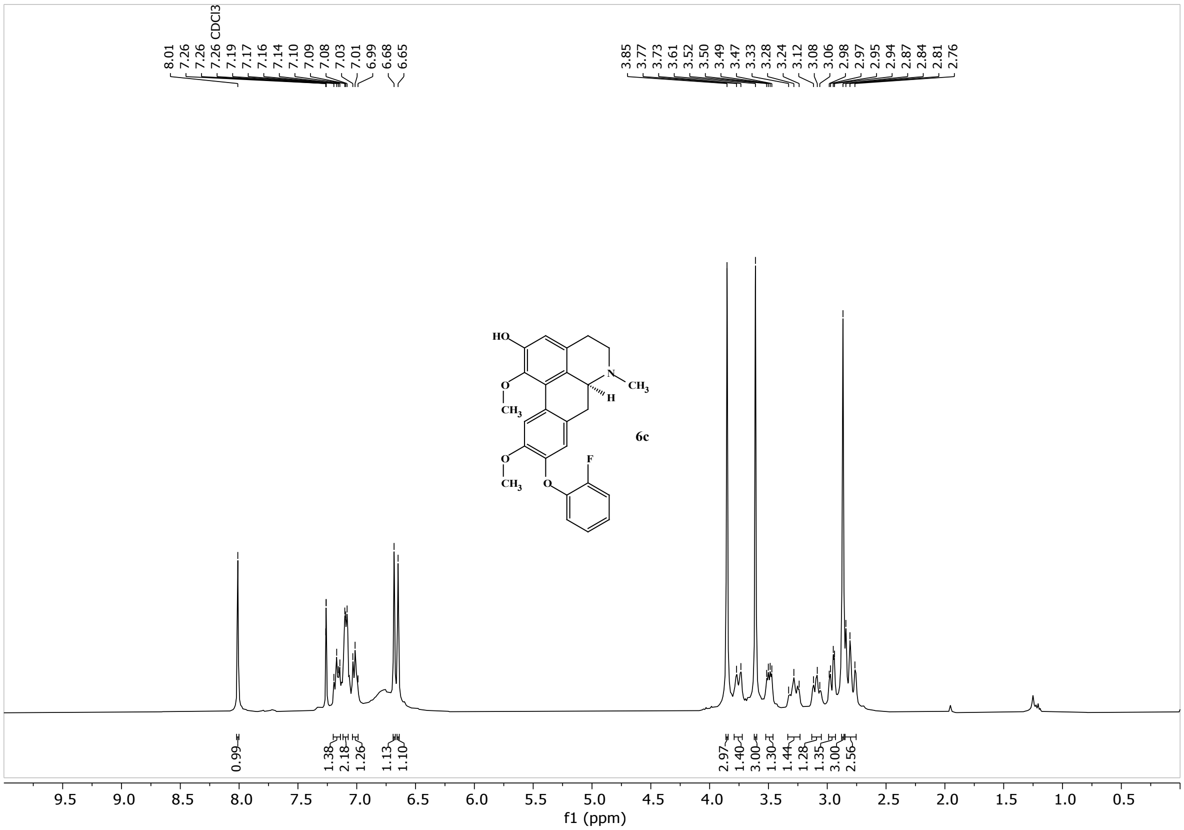

Figure S11. ^{1}H-NMR spectrum of **6c** ($CDCl_3$, 400 MHz).

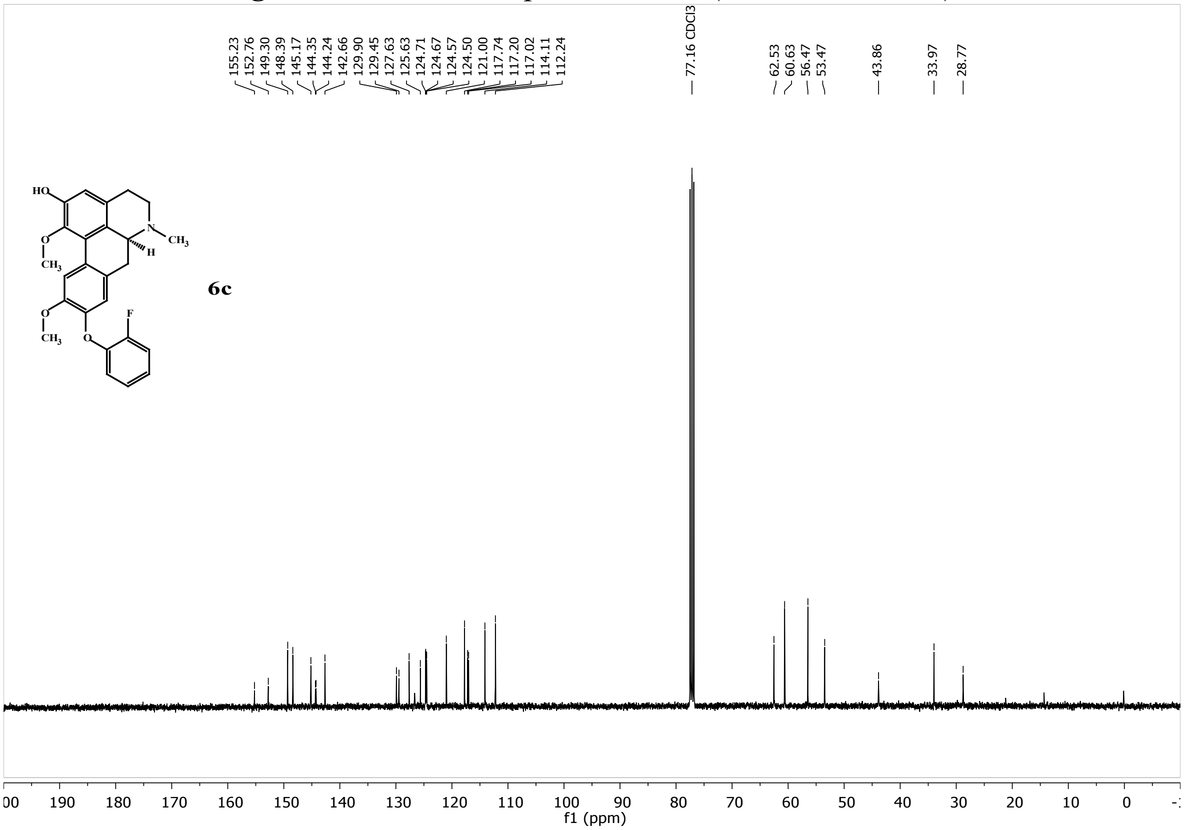

Figure S12. ^{13}C-NMR spectrum of **6c** ($CDCl_3$, 101 MHz).

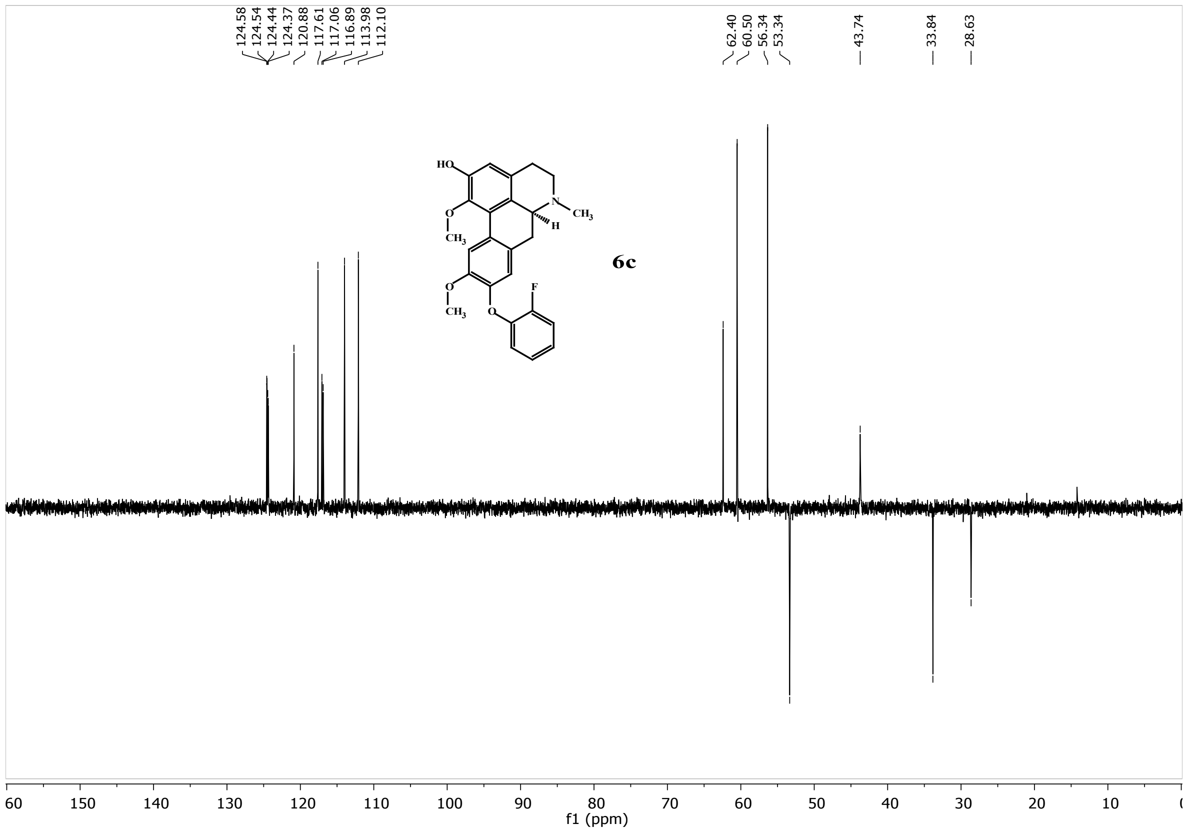

Figure S13. DEPT spectrum of **6c** ($CDCl_3$, 101 MHz).

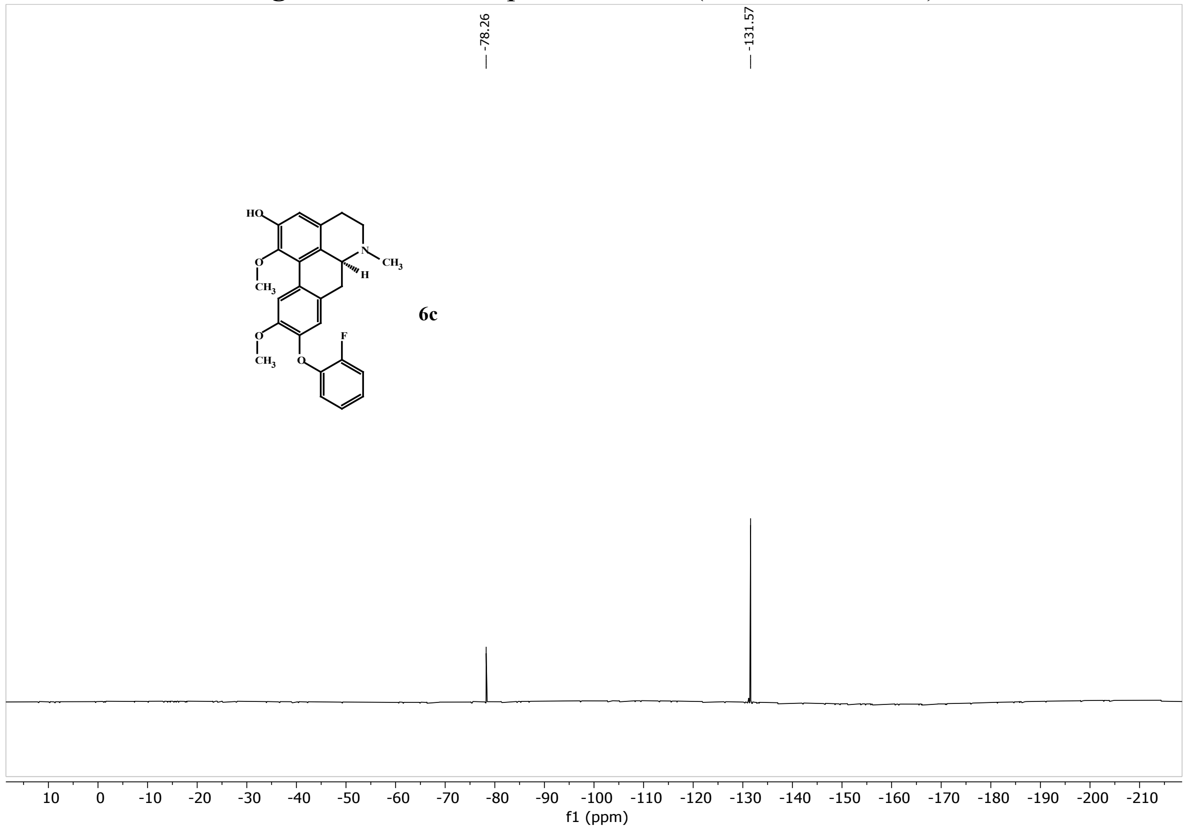

Figure S14. ^{19}F-NMR spectrum of **6c** ($CDCl_3$, 376 MHz).

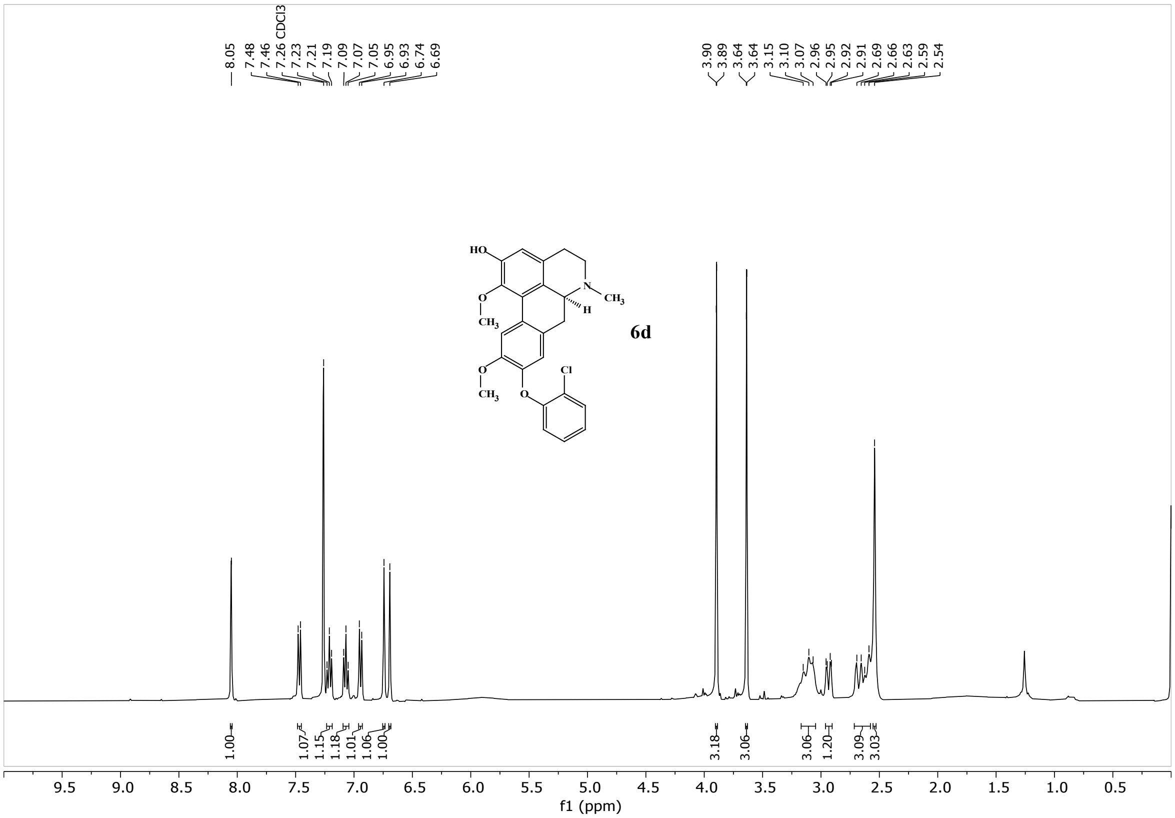

Figure S15. ^{1}H-NMR spectrum of **6d** ($CDCl_3$, 400 MHz).

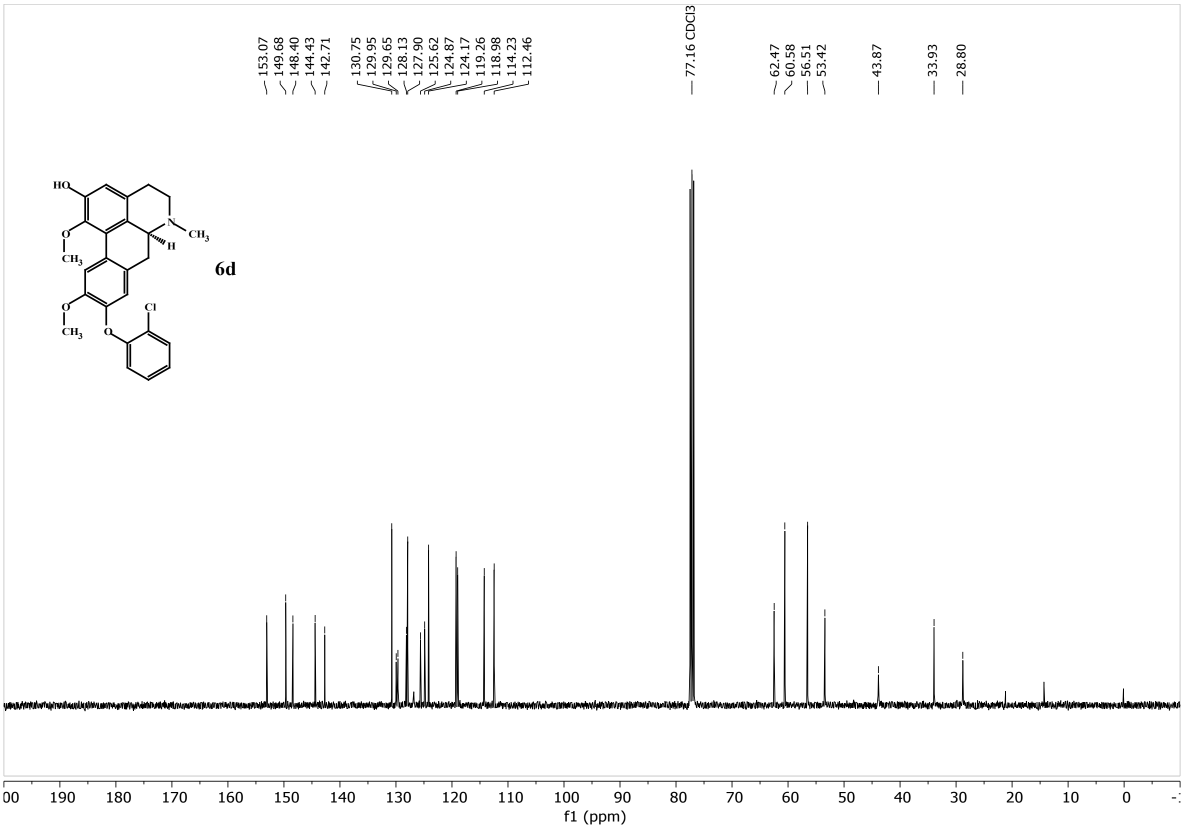

Figure S16. ^{13}C-NMR spectrum of **6d** ($CDCl_3$, 101 MHz).

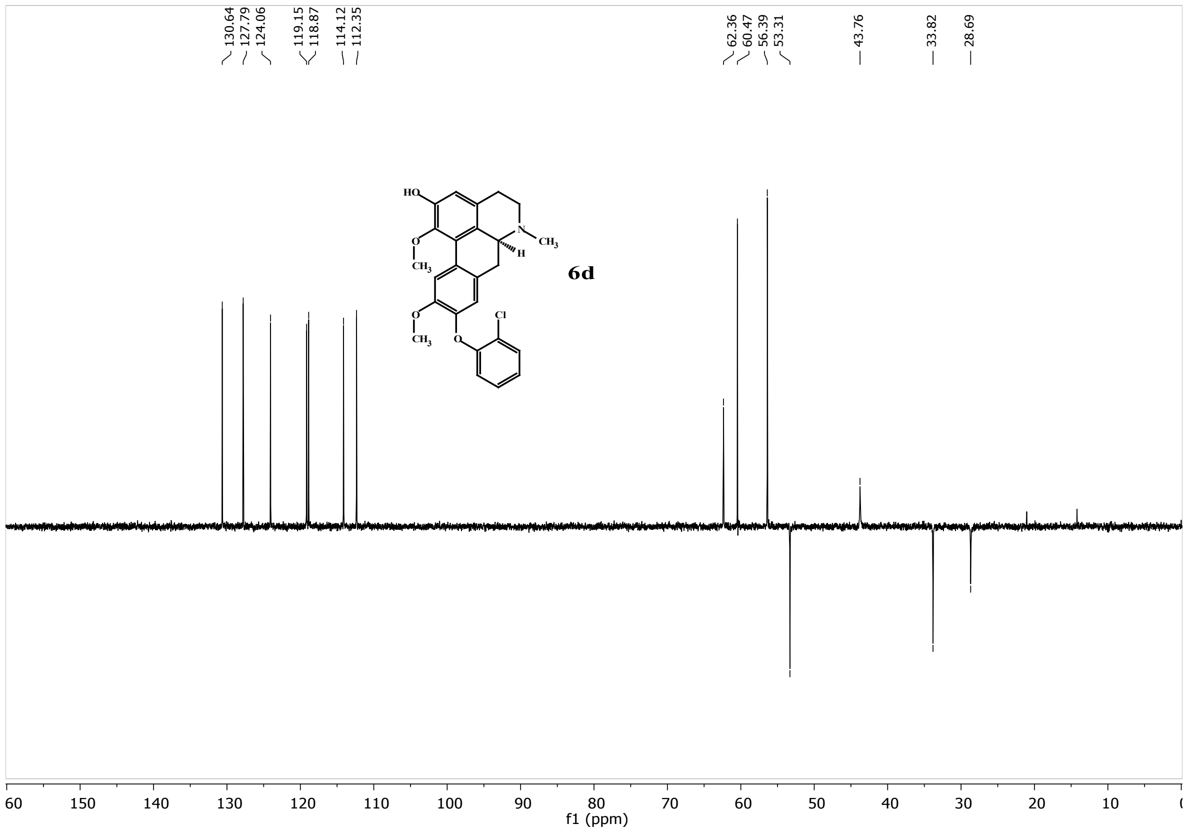

Figure S17. DEPT spectrum of **6d** ($CDCl_3$, 101 MHz).

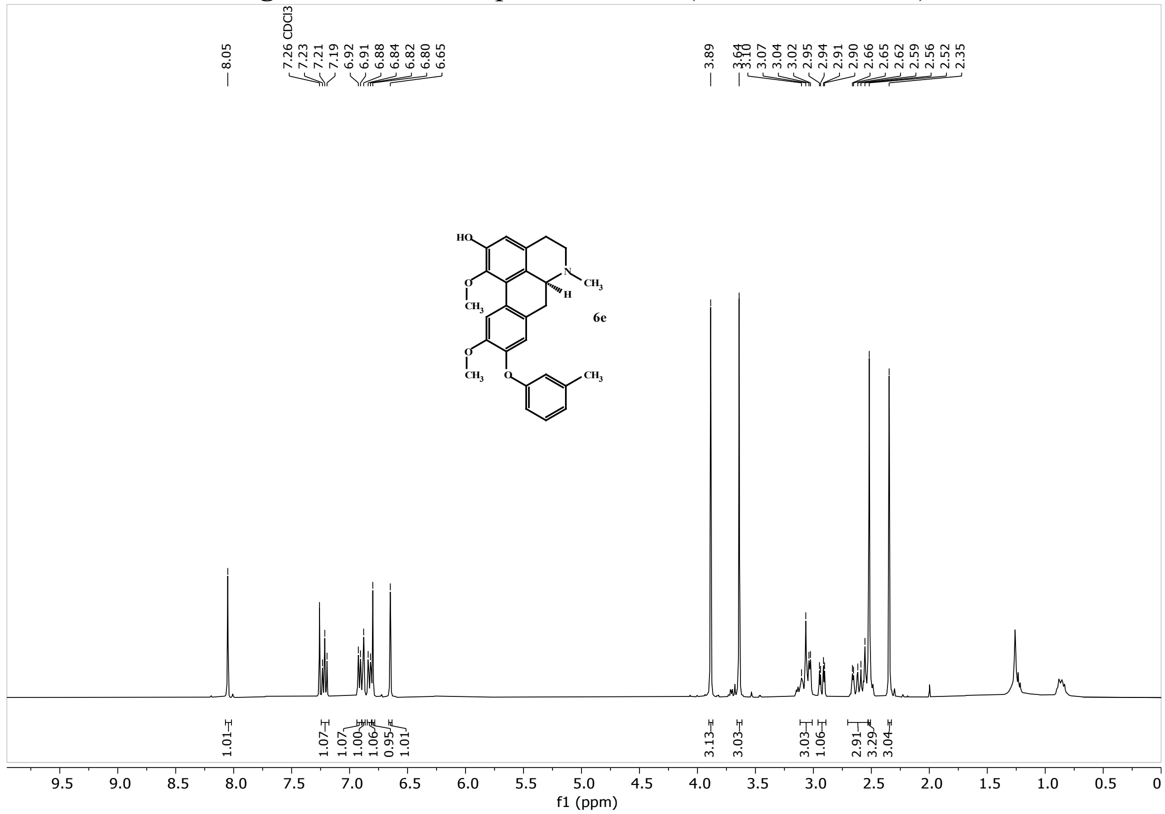

Figure S18. ^{1}H-NMR spectrum of **6e** ($CDCl_3$, 400 MHz).

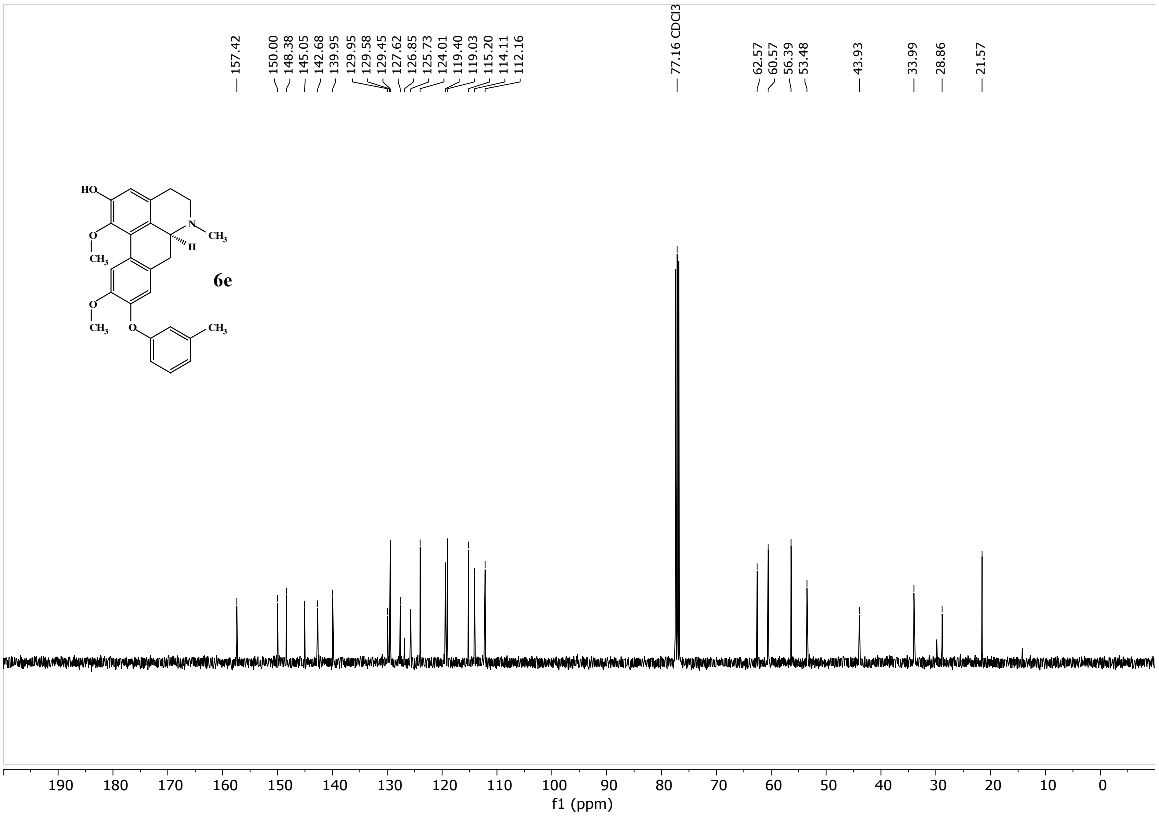

Figure S19. ^{13}C-NMR spectrum of **6e** ($CDCl_3$, 101 MHz).

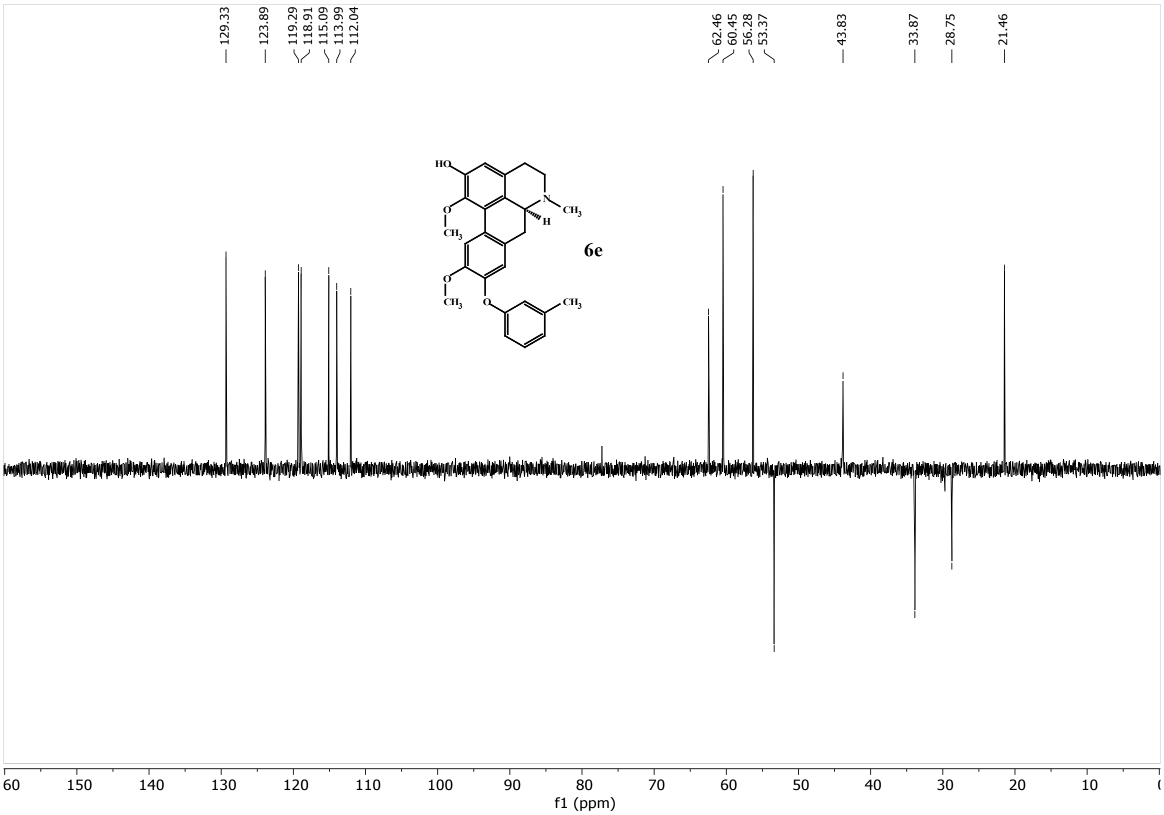

Figure S20. DEPT spectrum of **6e** ($CDCl_3$, 101 MHz).

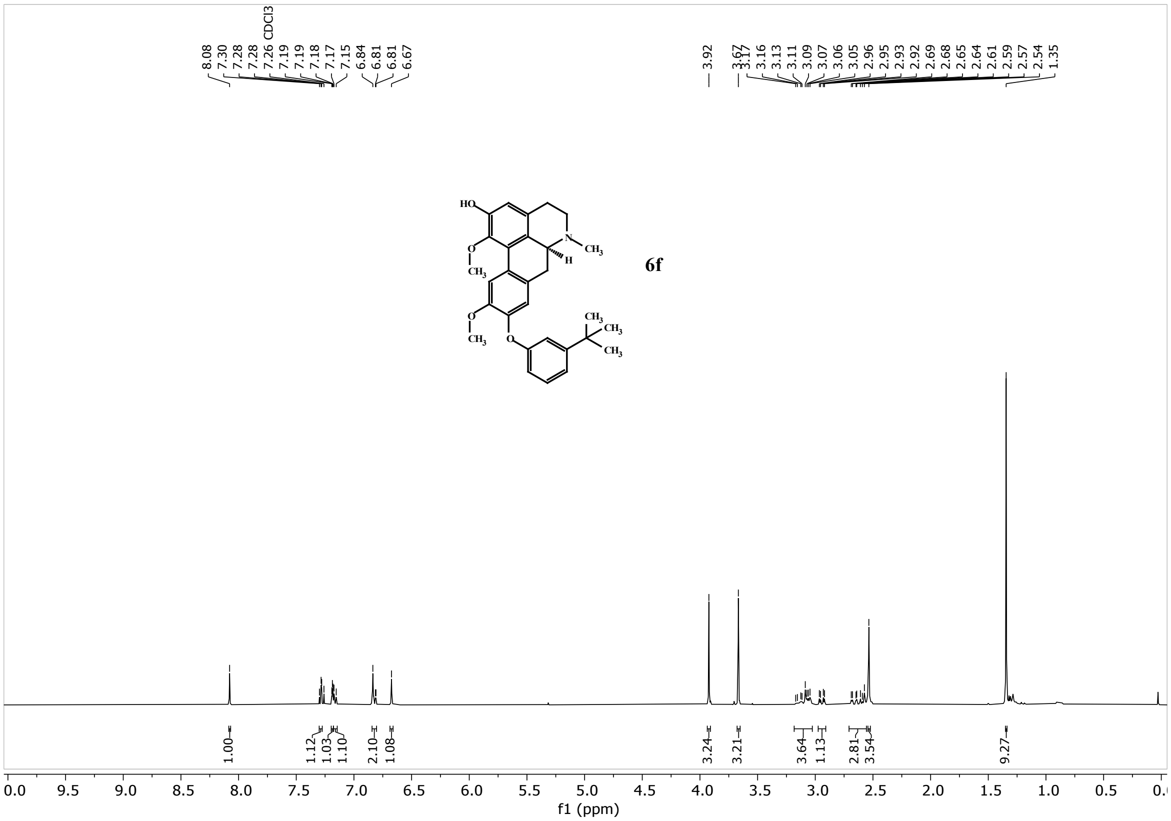

Figure S21. ^{1}H-NMR spectrum of **6f** ($CDCl_3$, 400 MHz).

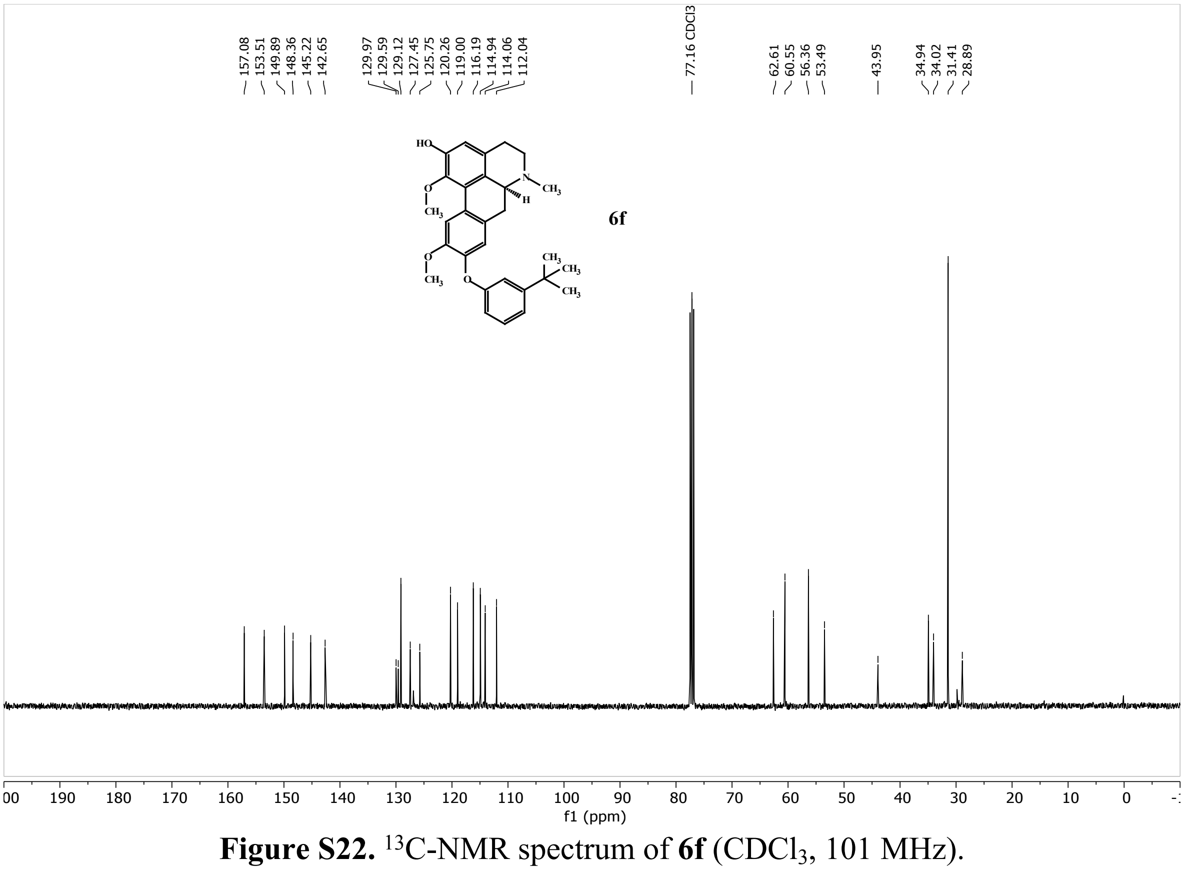

Figure S22. ^{13}C-NMR spectrum of **6f** ($CDCl_3$, 101 MHz).

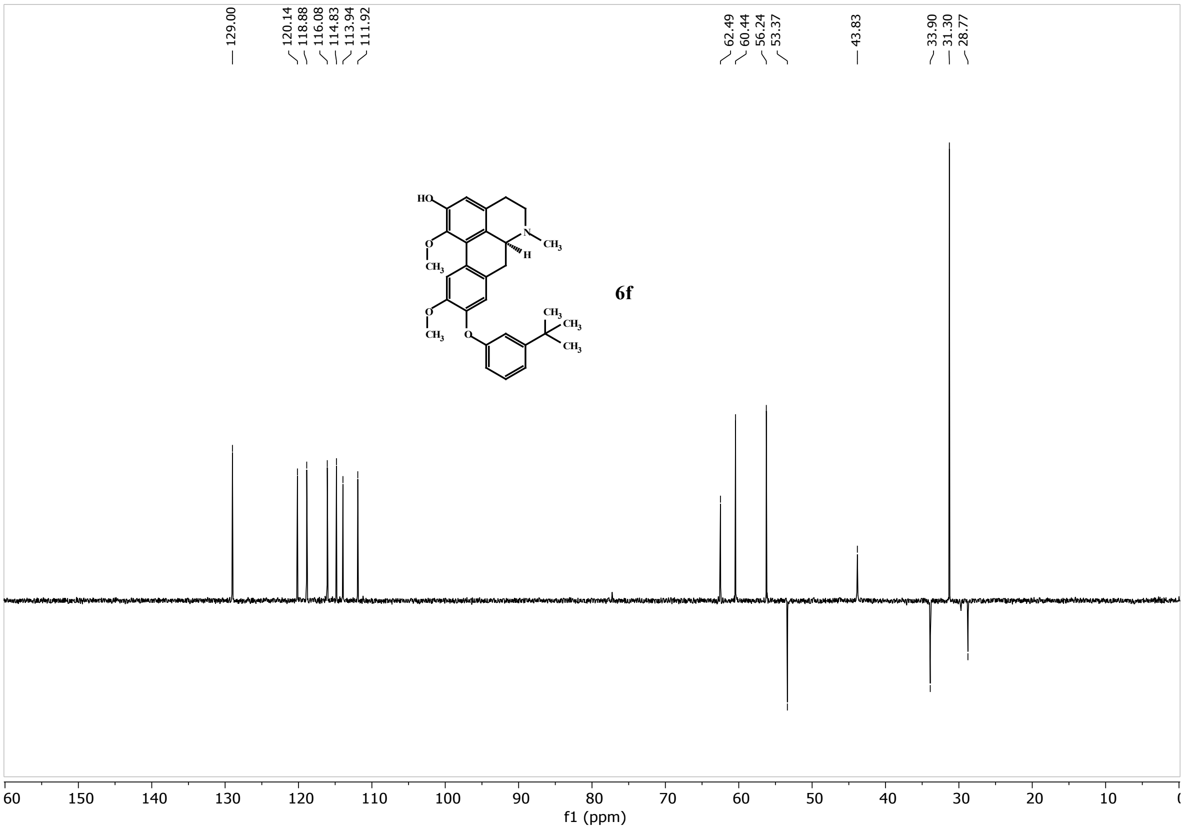

Figure S23. DEPT spectrum of **6f** ($CDCl_3$, 101 MHz).

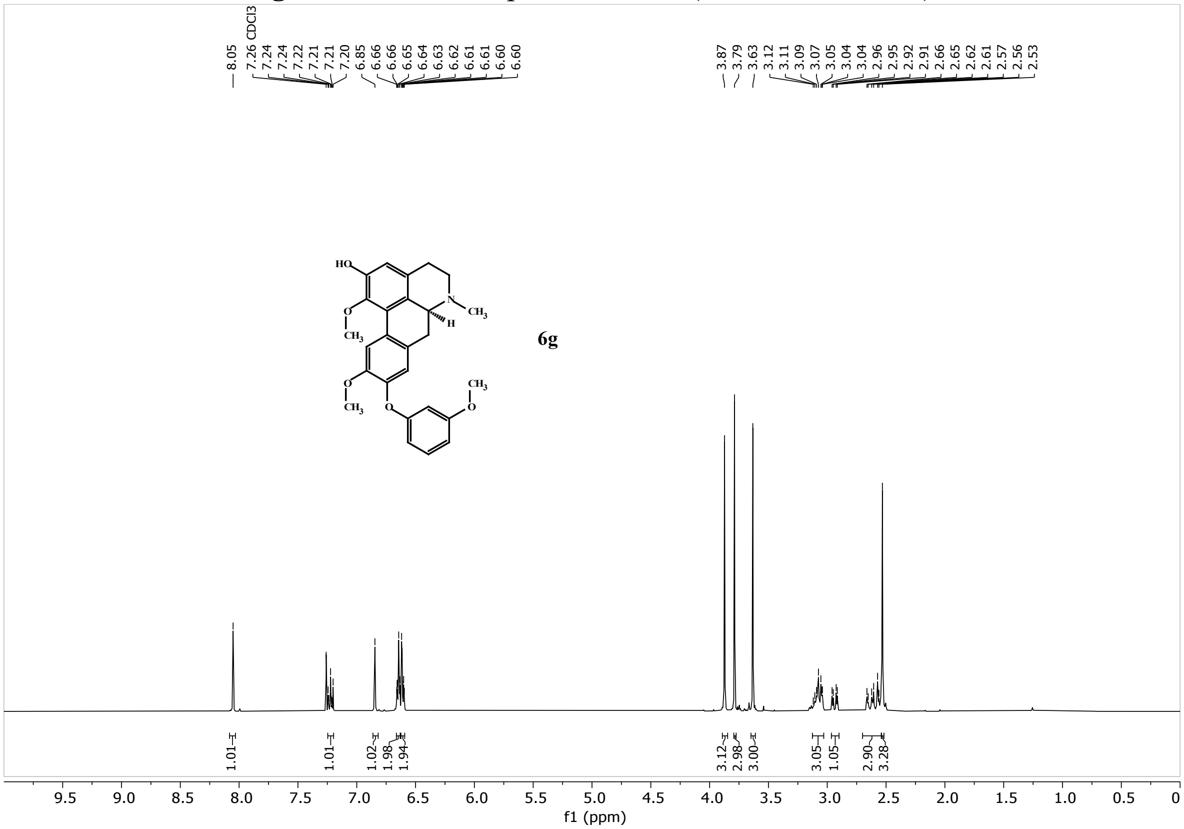

Figure S24. ^{1}H-NMR spectrum of **6g** ($CDCl_3$, 400 MHz).

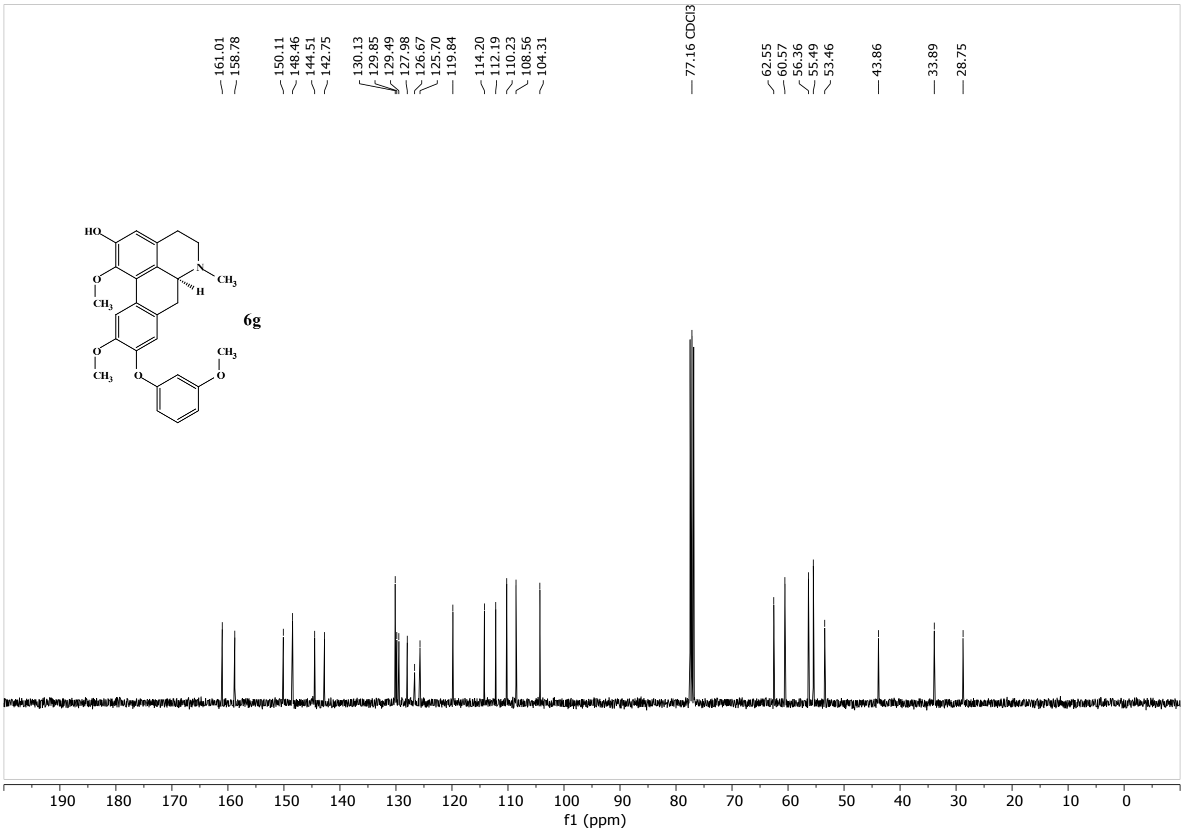

Figure S25. ^{13}C-NMR spectrum of **6g** ($CDCl_3$, 101 MHz).

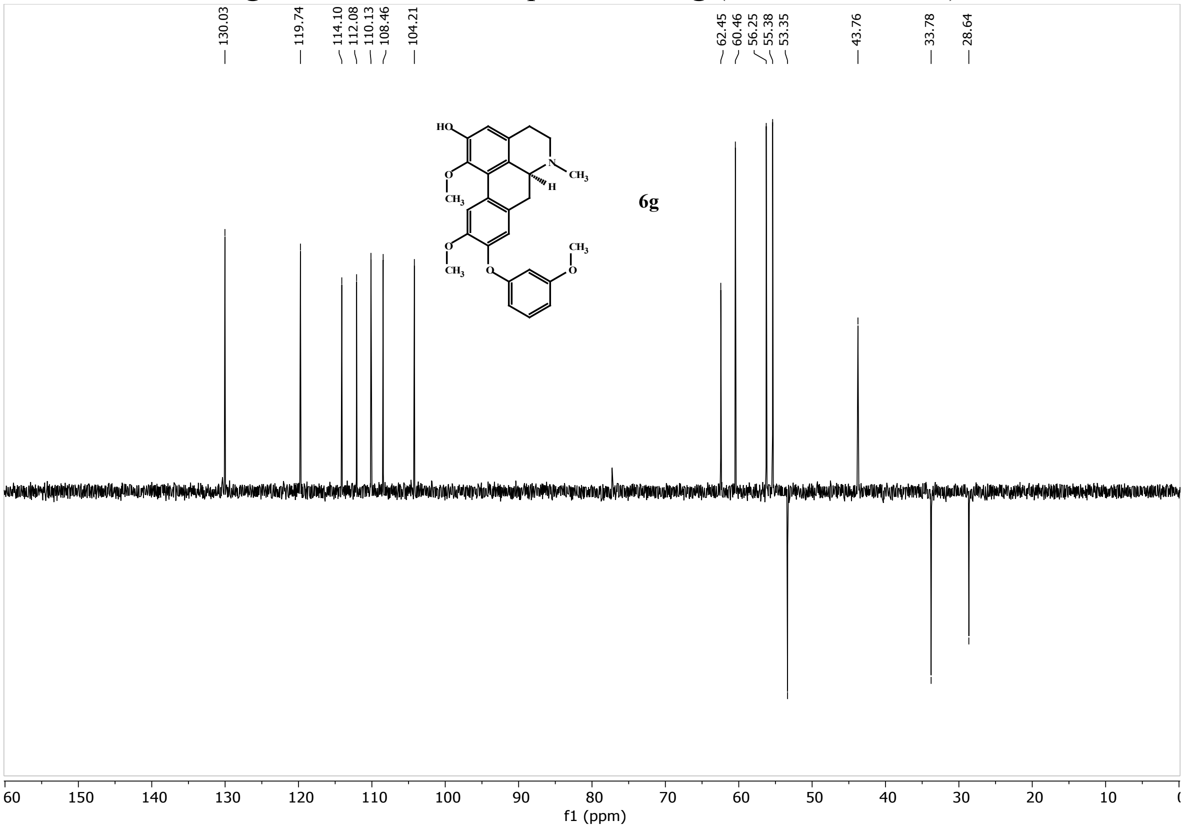

Figure S26. DEPT spectrum of **6g** ($CDCl_3$, 101 MHz).

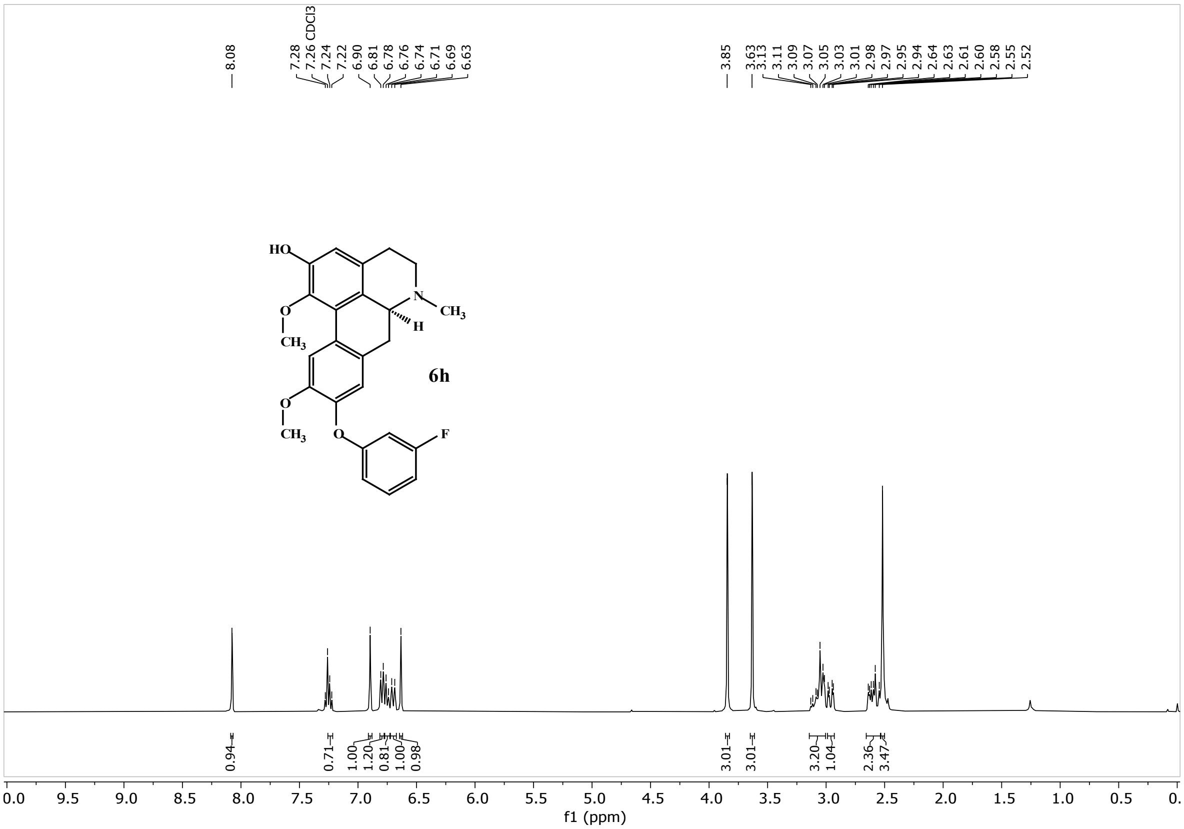

Figure S27. ^{1}H-NMR spectrum of **6h** ($CDCl_3$, 400 MHz).

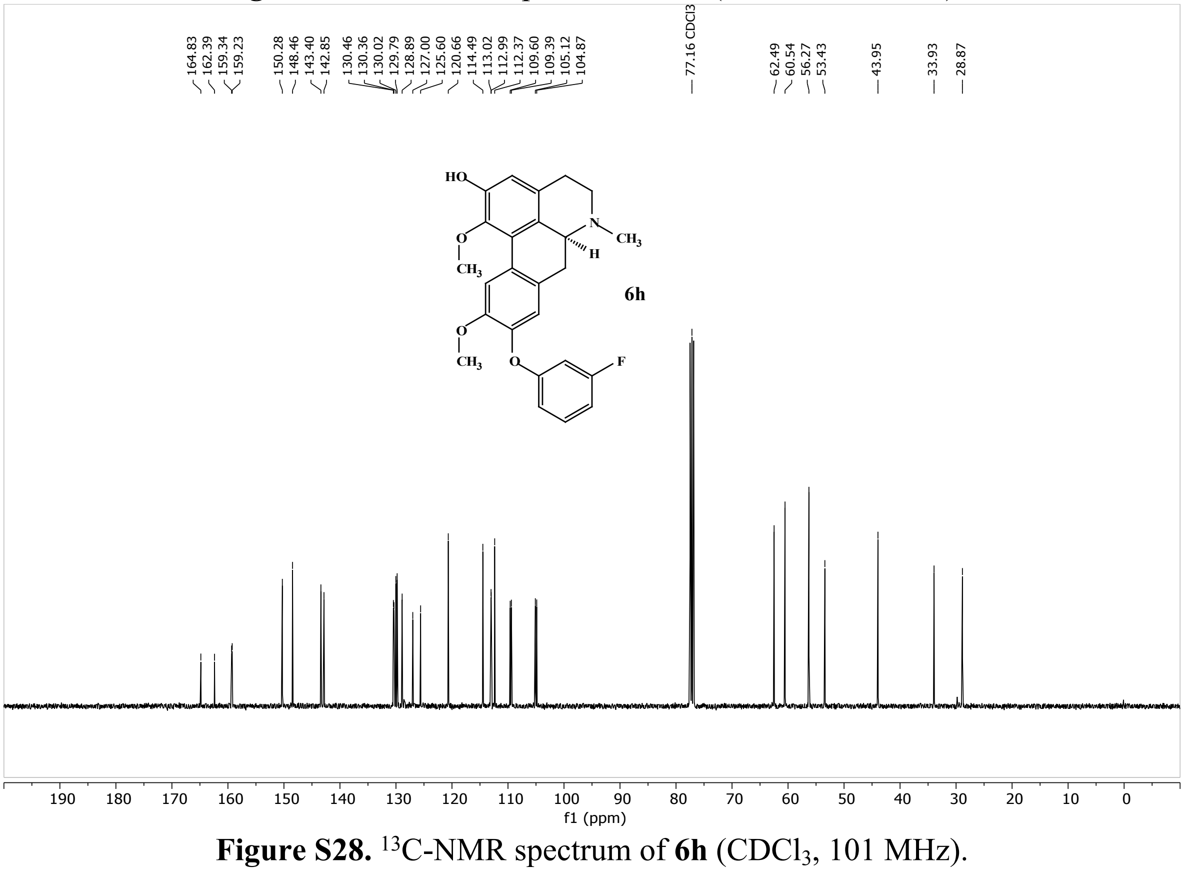

Figure S28. ^{13}C-NMR spectrum of **6h** ($CDCl_3$, 101 MHz).

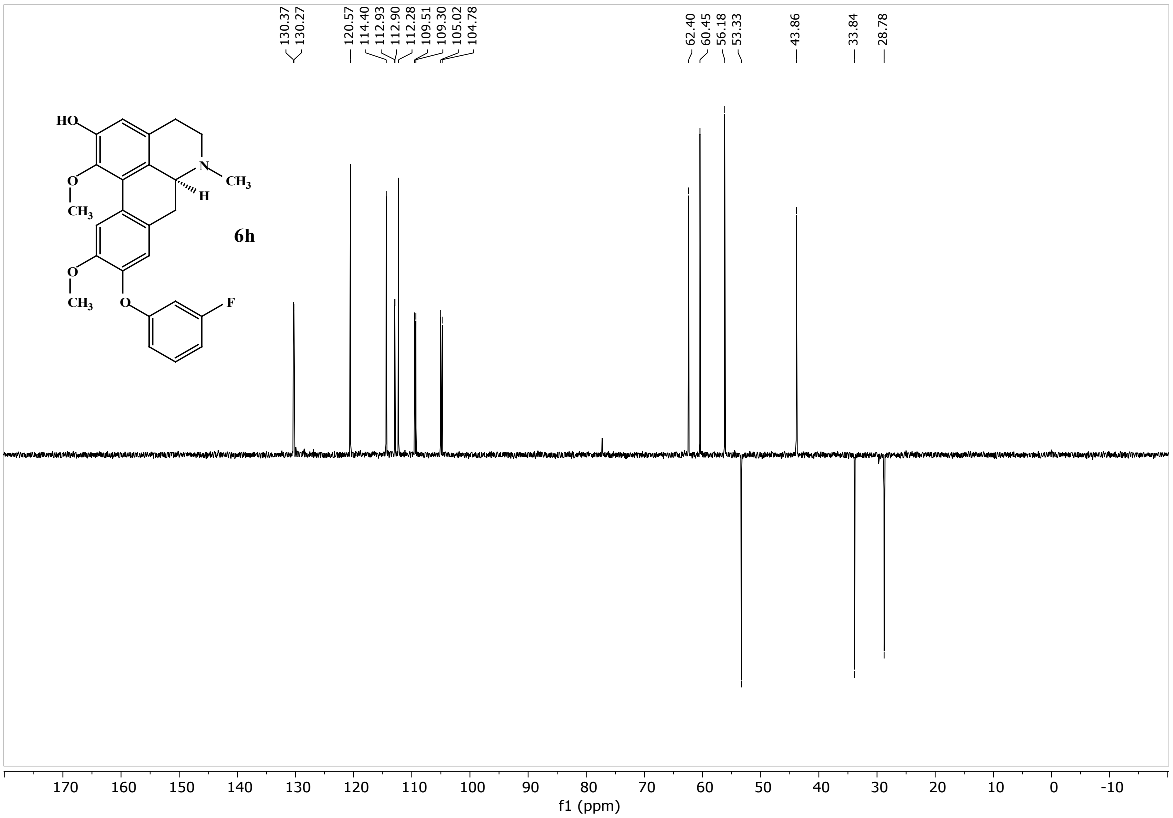

Figure S29. DEPT spectrum of **6h** ($CDCl_3$, 101 MHz).

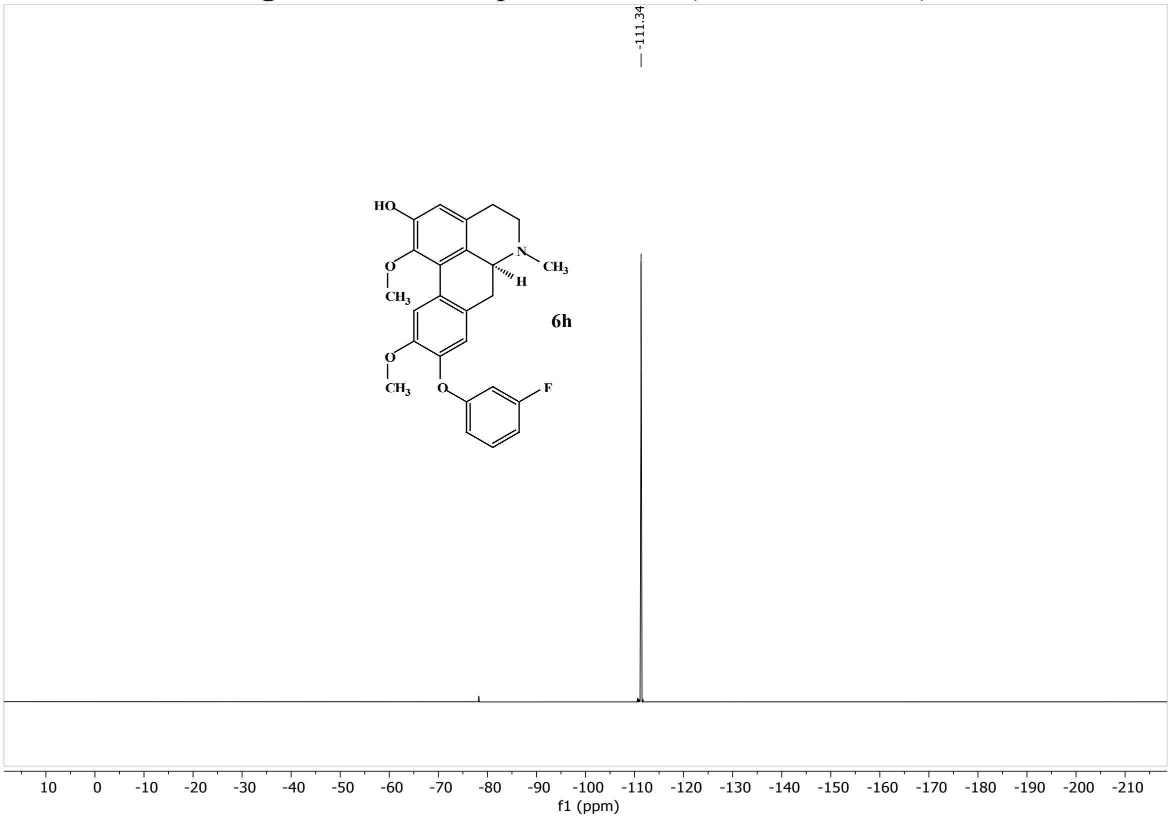

Figure S30. ^{19}F-NMR spectrum of **6h** ($CDCl_3$, 376 MHz).

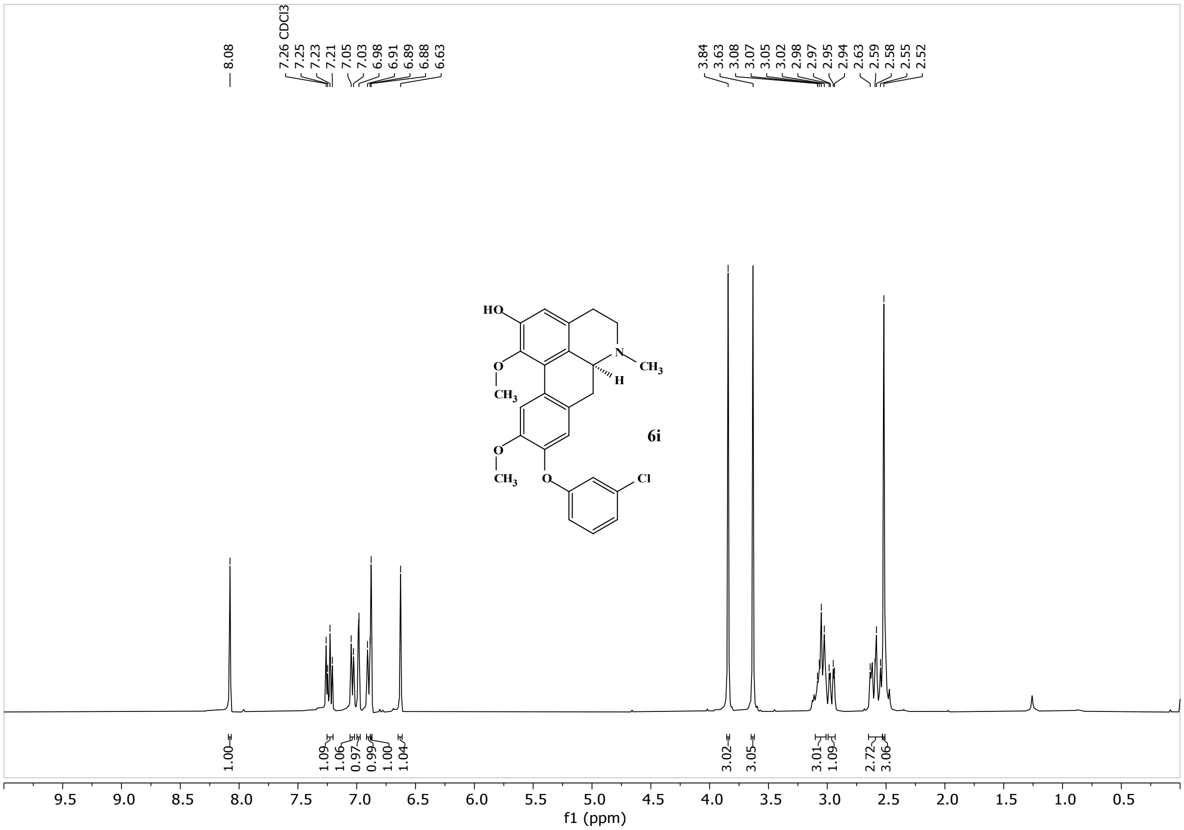

Figure S31. ^{1}H-NMR spectrum of **6i** ($CDCl_3$, 400 MHz).

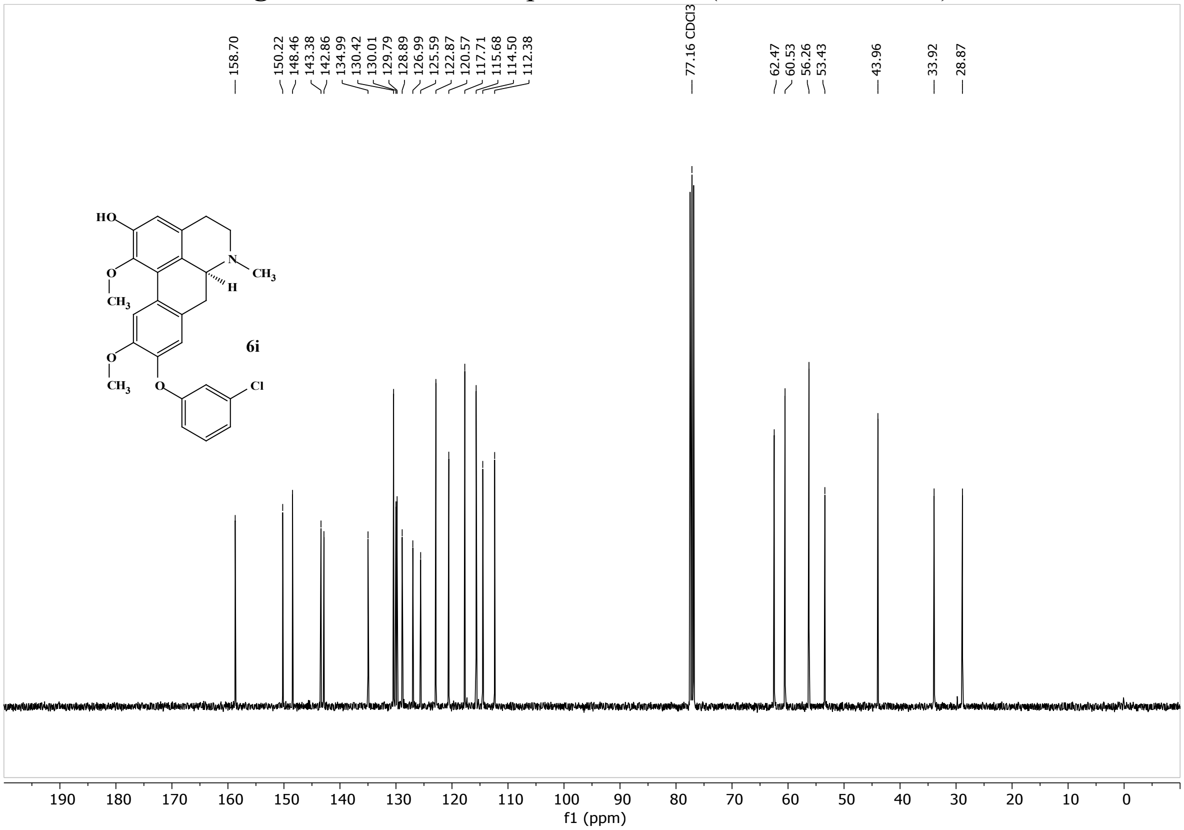

Figure S32. ^{13}C-NMR spectrum of **6i** ($CDCl_3$, 101 MHz).

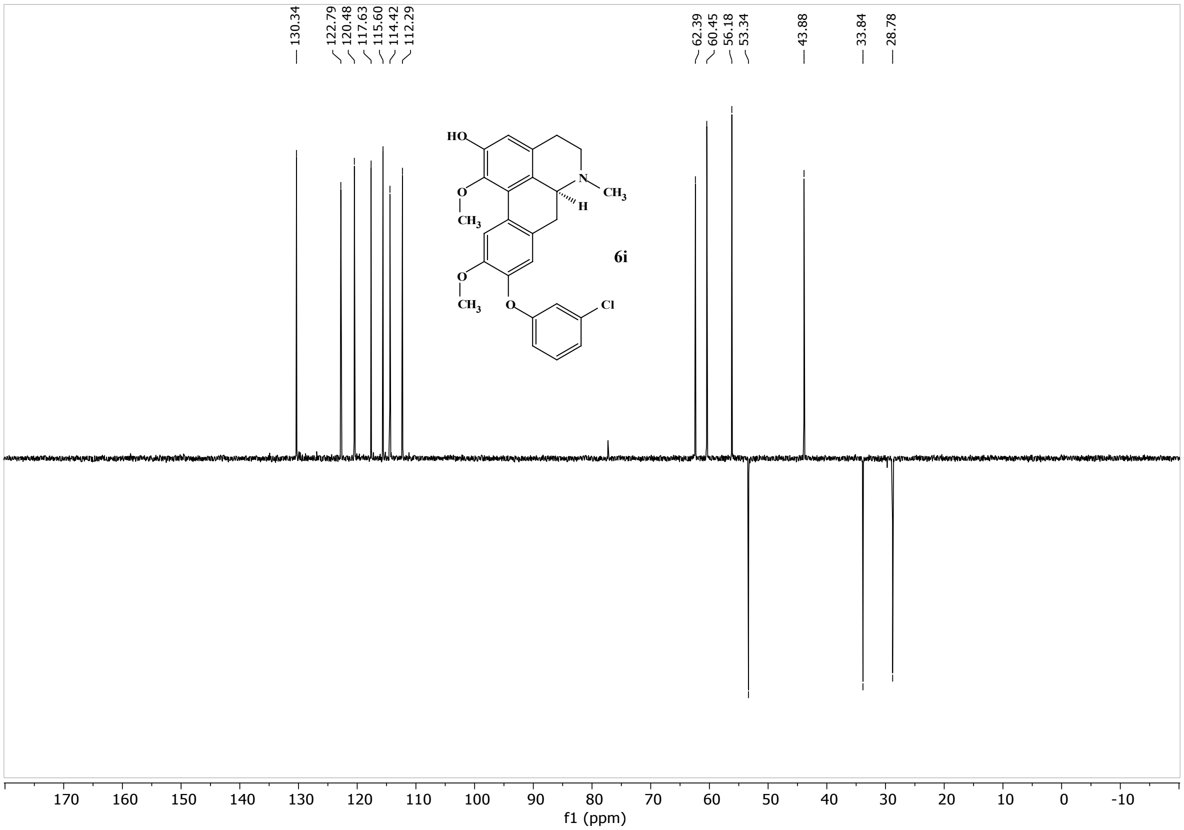

Figure S33. DEPT spectrum of **6i** ($CDCl_3$, 101 MHz).

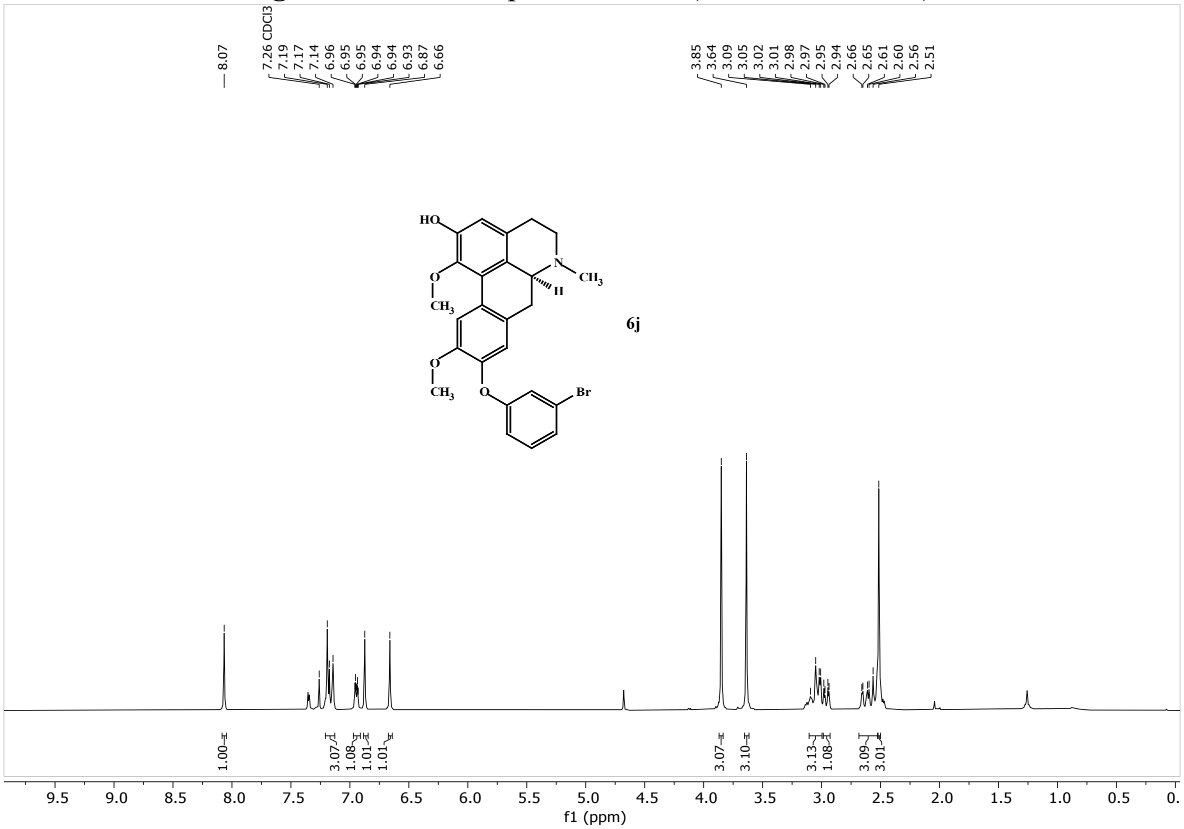

Figure S34. ^{1}H-NMR spectrum of **6j** ($CDCl_3$, 400 MHz).

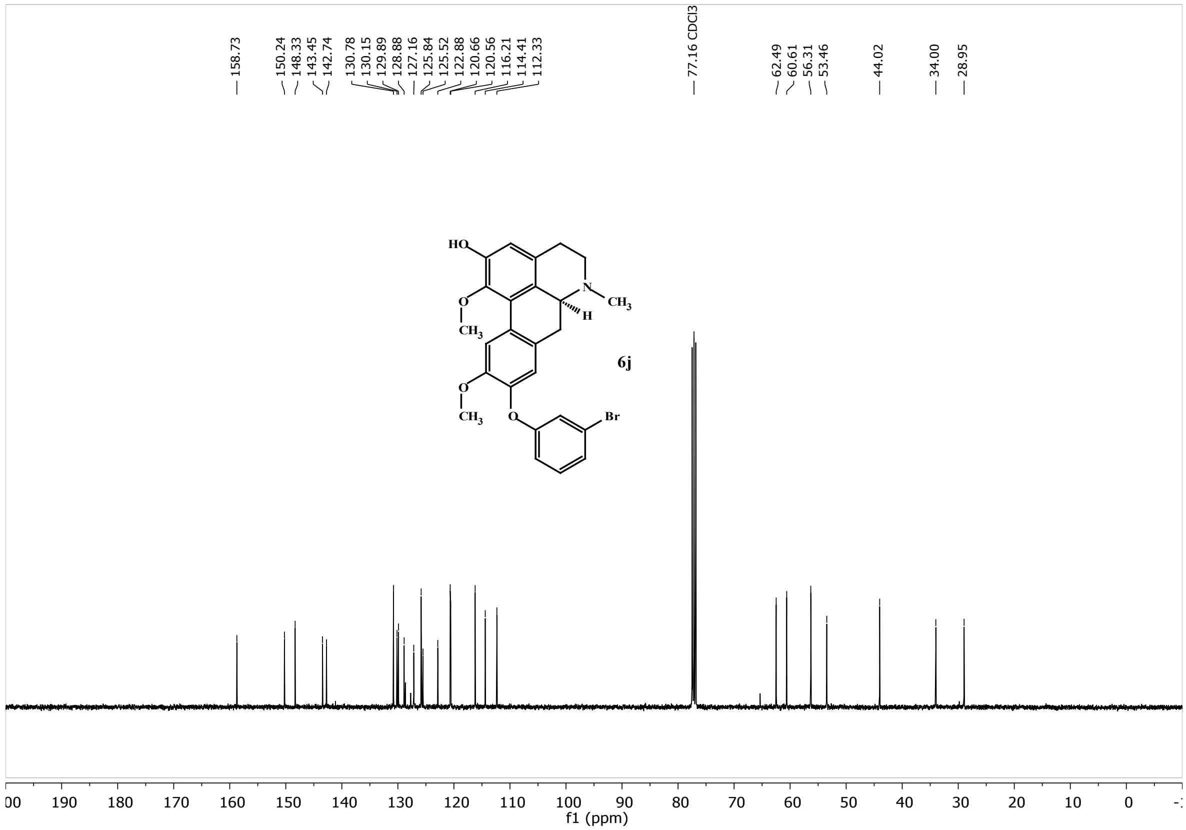

Figure S35. ^{13}C-NMR spectrum of **6j** ($CDCl_3$, 101 MHz).

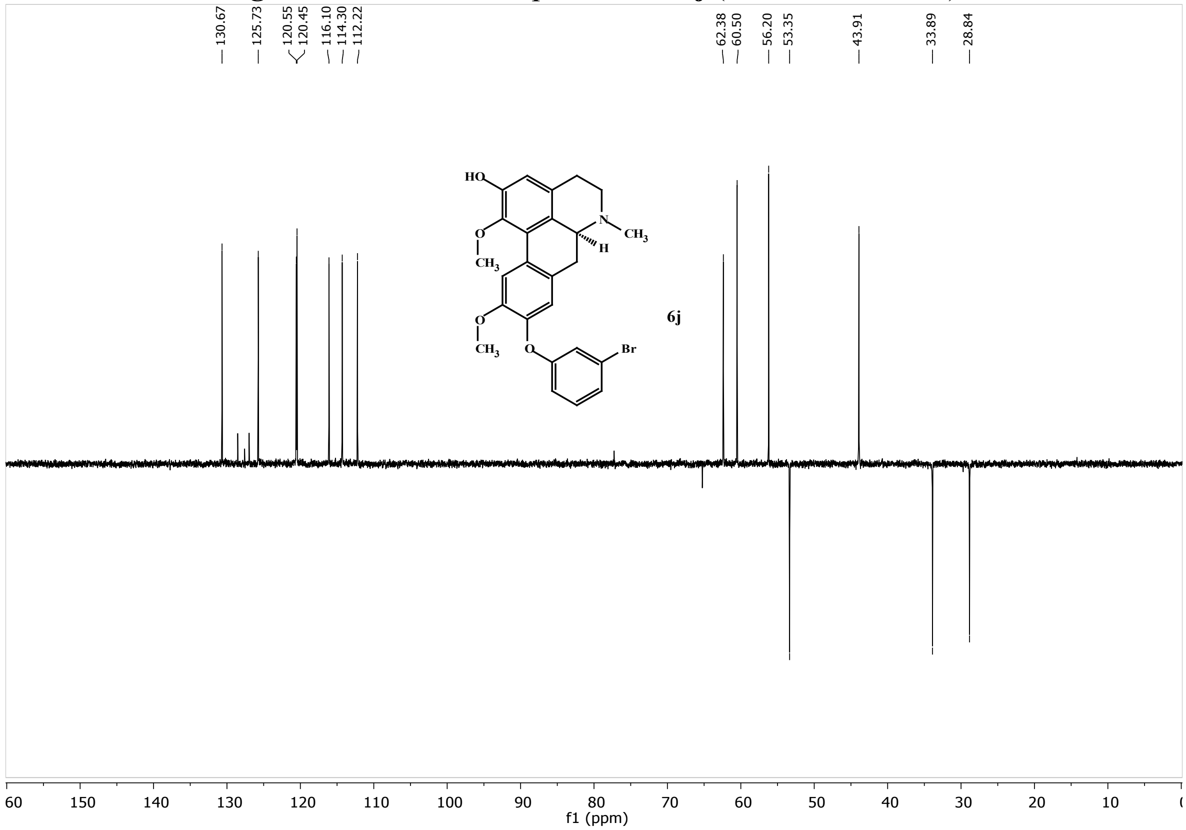

Figure S36. DEPT spectrum of **6j** ($CDCl_3$, 101 MHz).

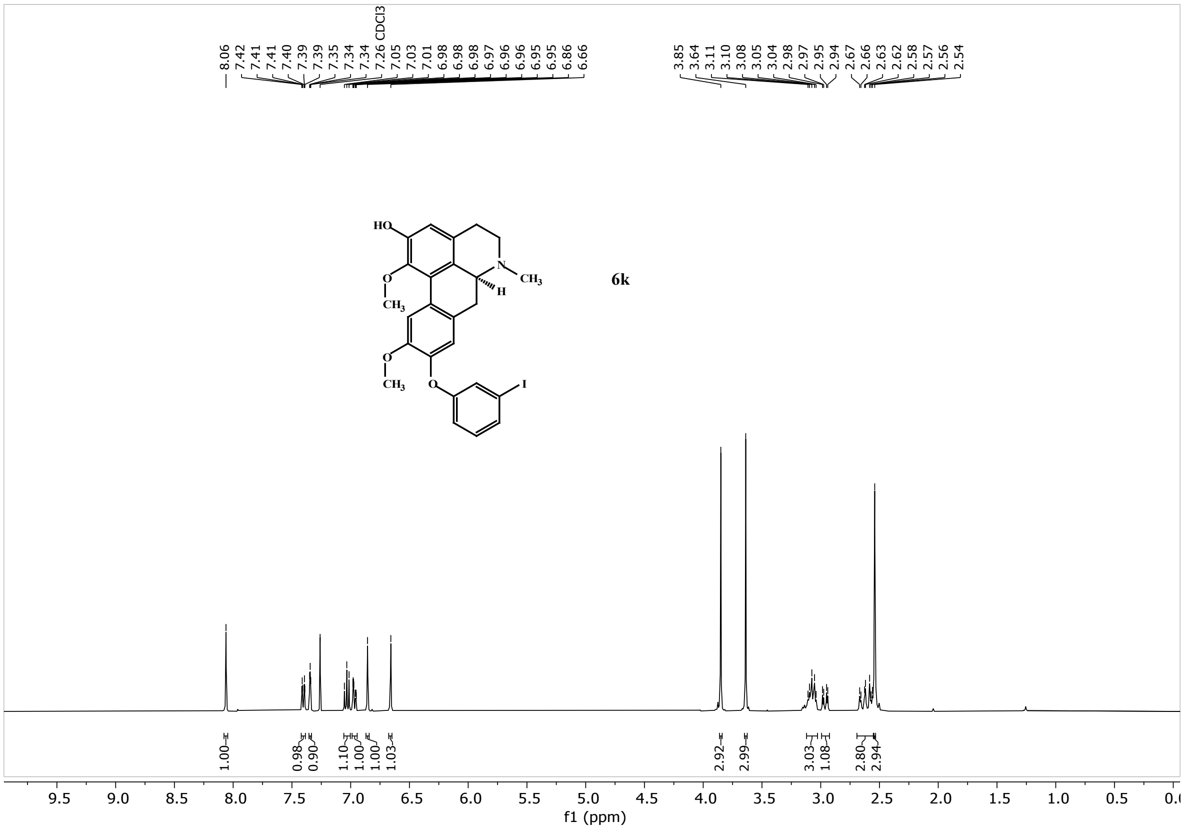

Figure S37. ^{1}H-NMR spectrum of **6k** ($CDCl_3$, 400 MHz).

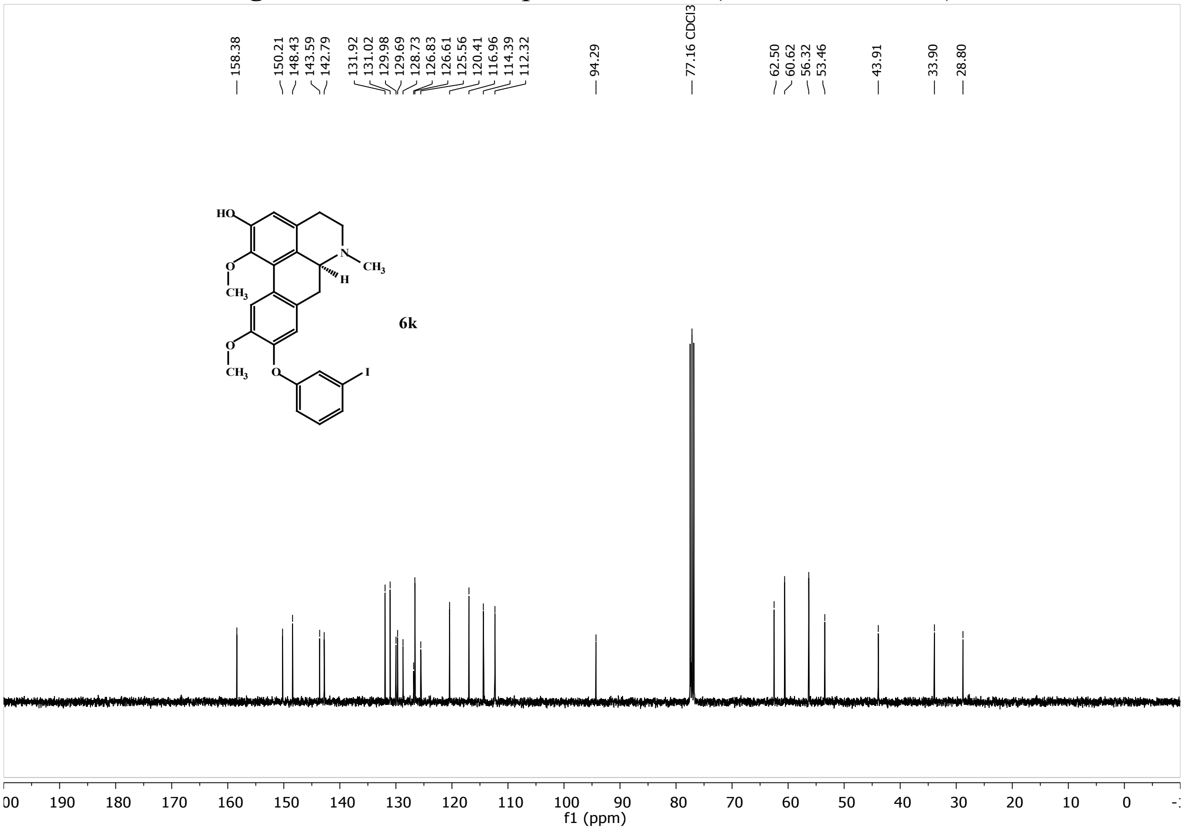

Figure S38. ^{13}C-NMR spectrum of **6k** ($CDCl_3$, 101 MHz).

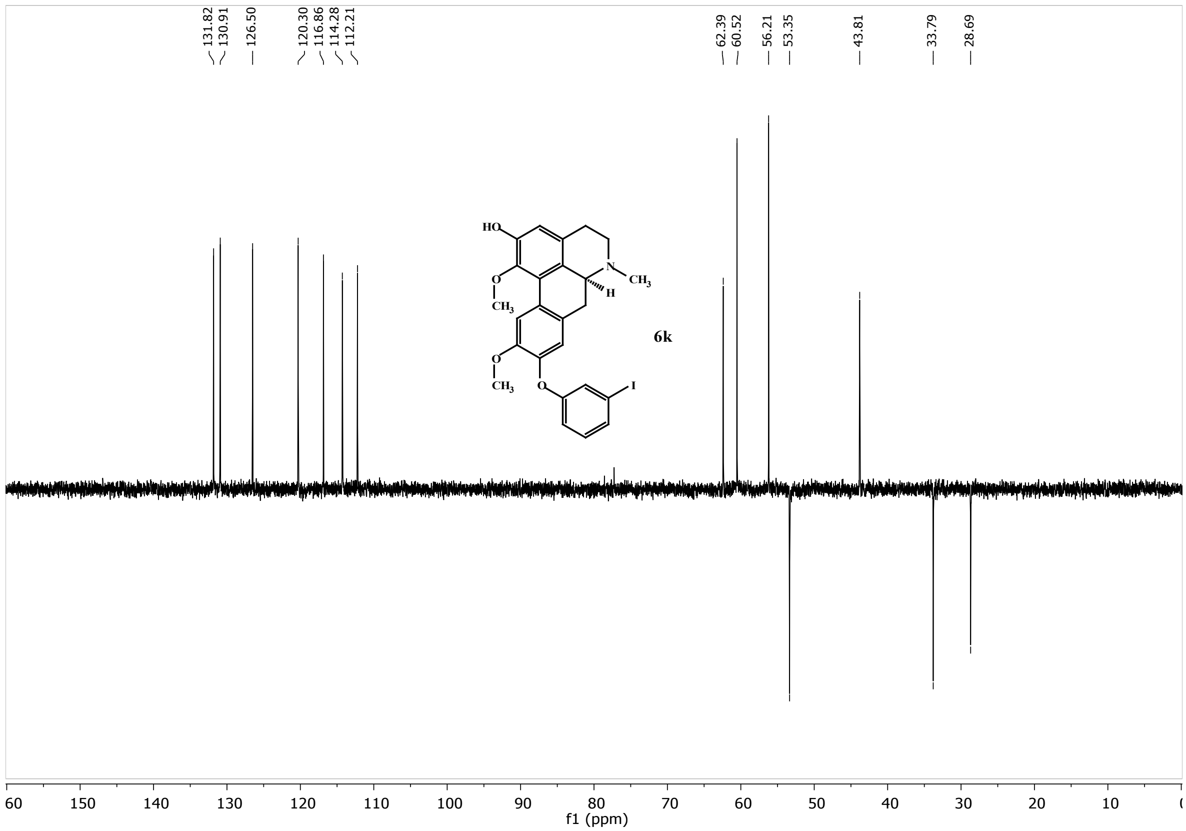

Figure S39. DEPT spectrum of **6k** ($CDCl_3$, 101 MHz).

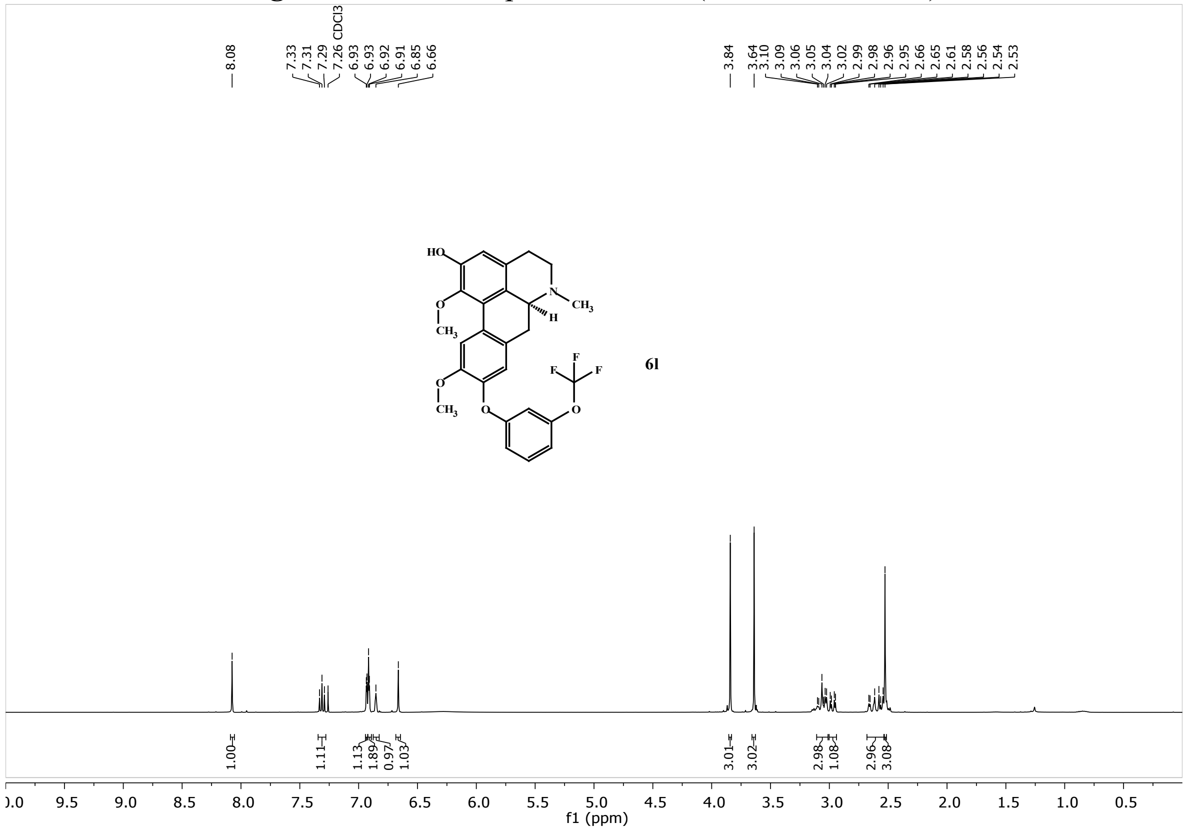

Figure S40. ^{1}H-NMR spectrum of **6l** ($CDCl_3$, 400 MHz).

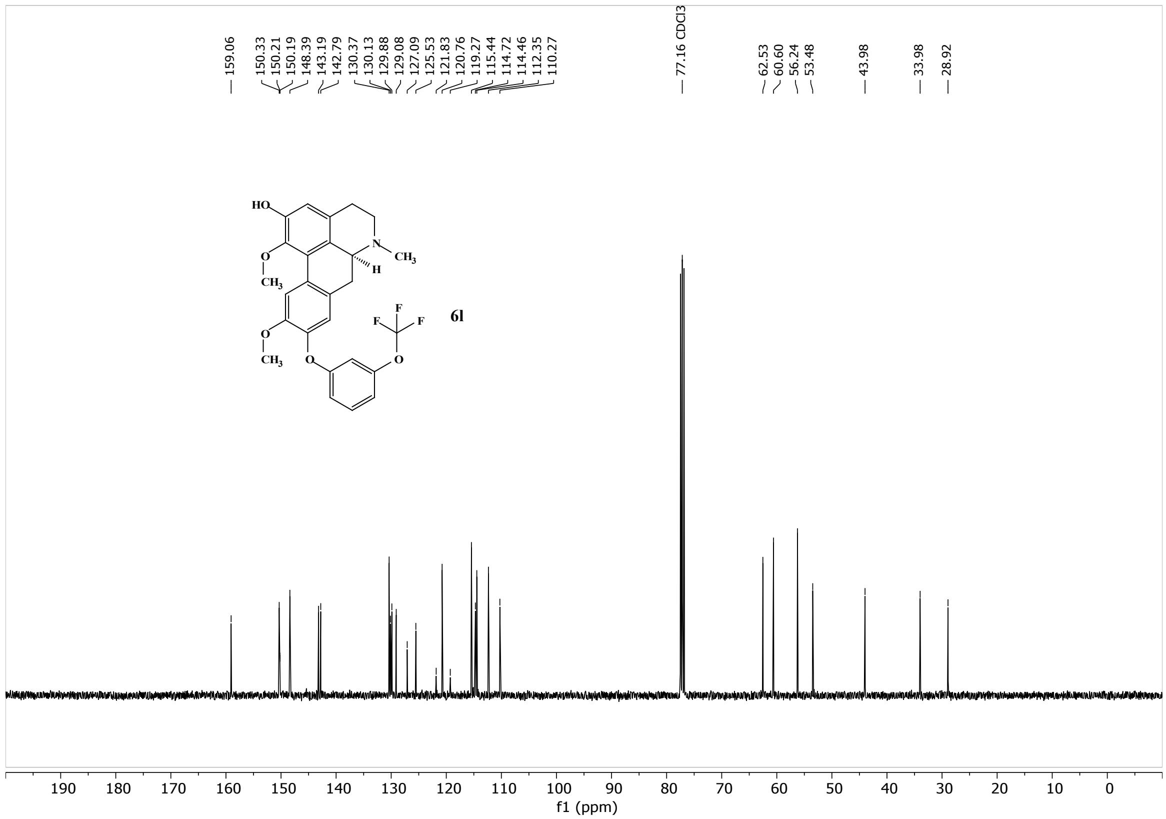

Figure S41. ^{13}C-NMR spectrum of **6l** ($CDCl_3$, 101 MHz).

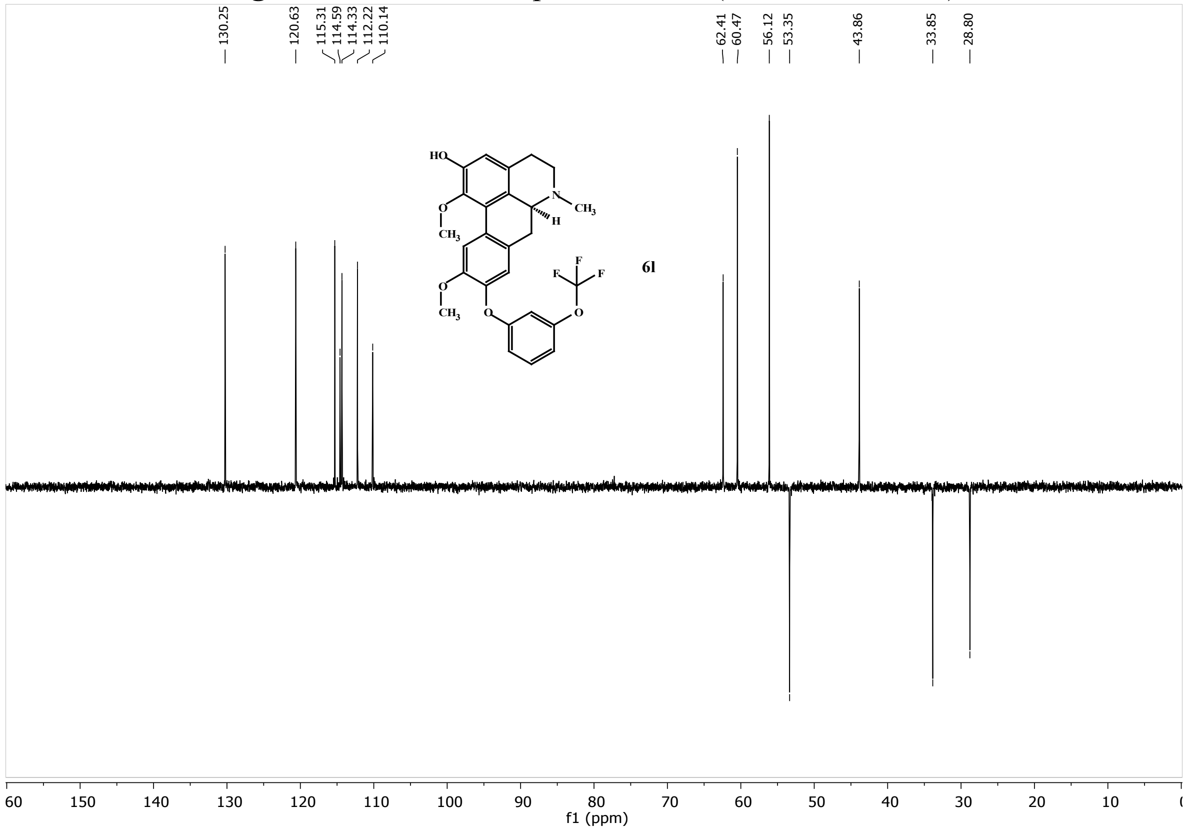

Figure S42. DEPT spectrum of **6l** ($CDCl_3$, 101 MHz).

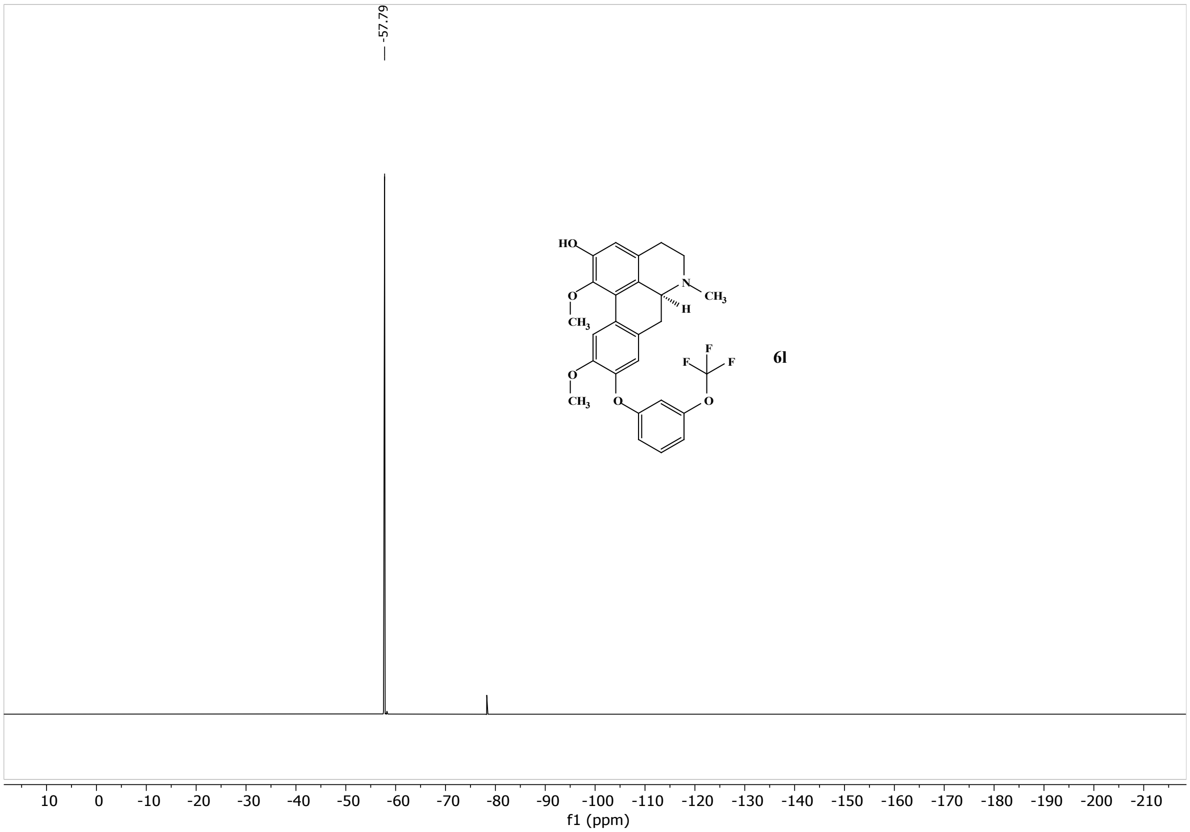

Figure S43. ^{19}F-NMR spectrum of **6l** ($CDCl_3$, 376 MHz).

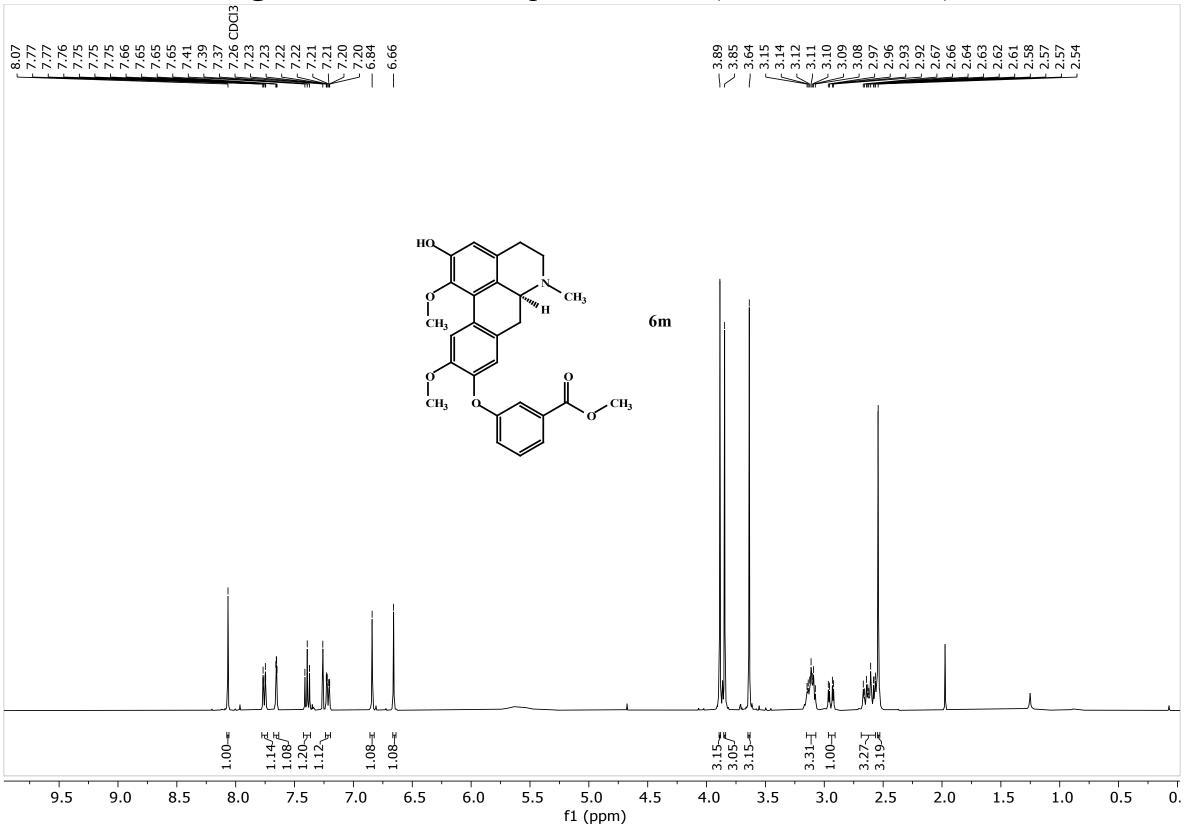

Figure S44. ^{1}H-NMR spectrum of **6m** ($CDCl_3$, 400 MHz).

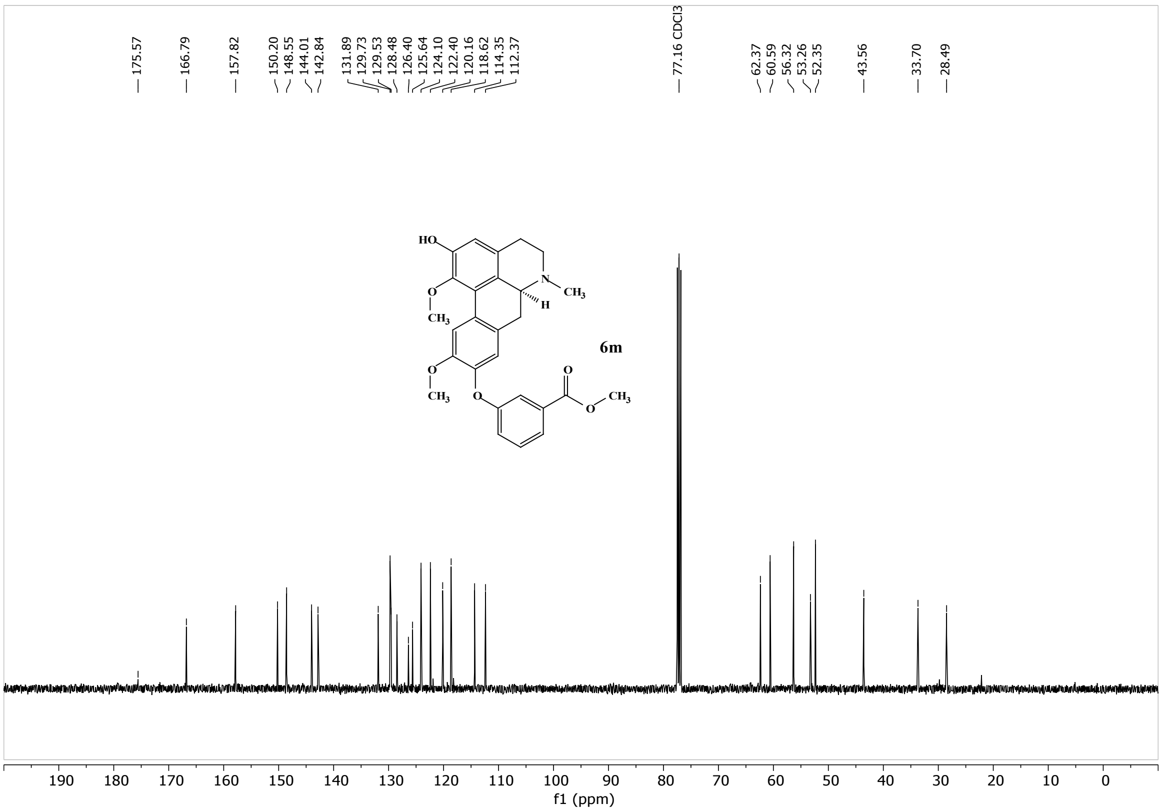

Figure S45. ^{13}C-NMR spectrum of **6m** ($CDCl_3$, 101 MHz).

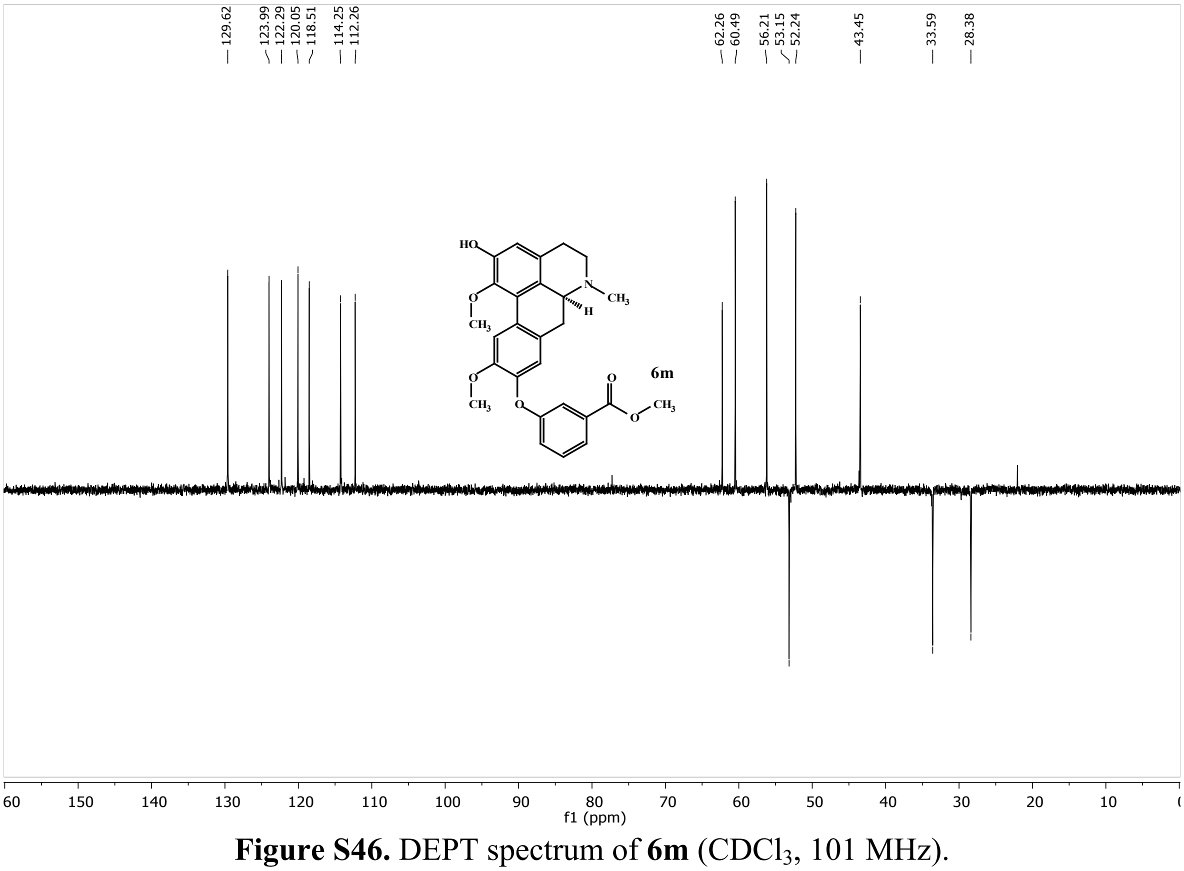

Figure S46. DEPT spectrum of **6m** ($CDCl_3$, 101 MHz).

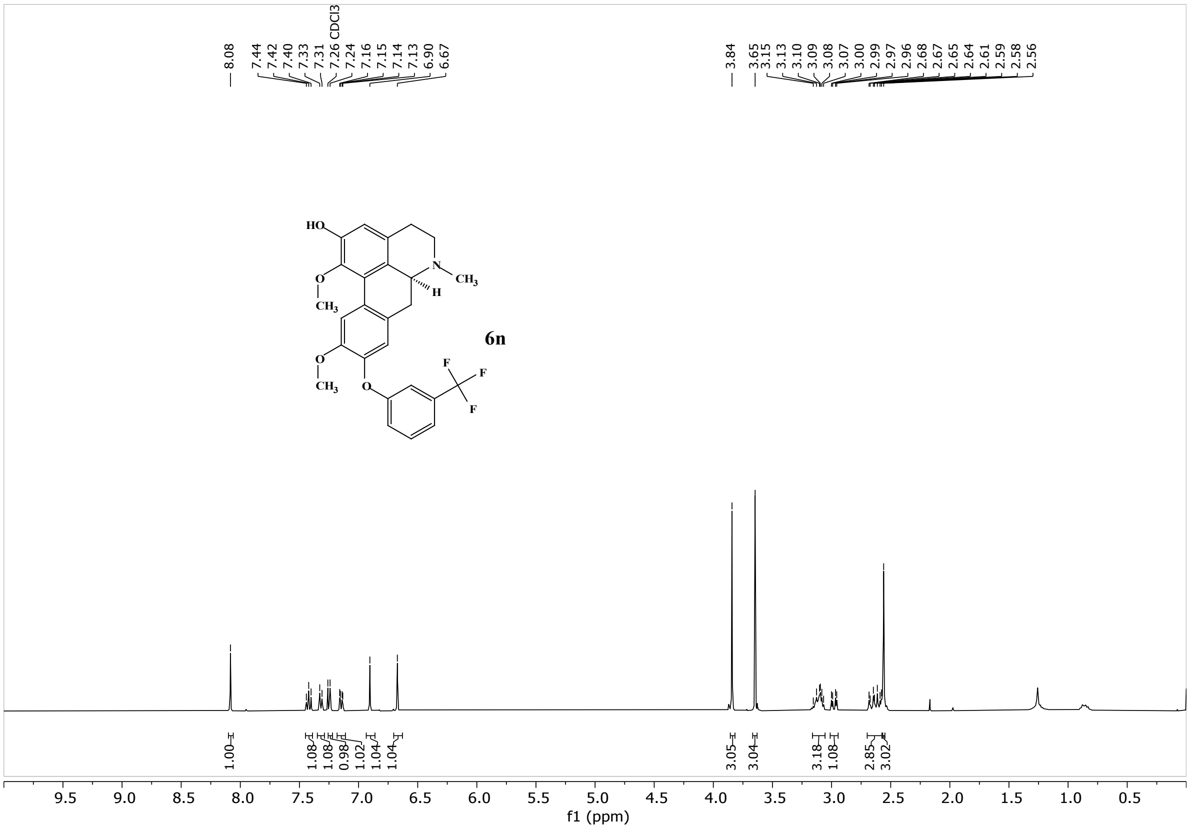

Figure S47. ^{1}H-NMR spectrum of **6n** ($CDCl_3$, 400 MHz).

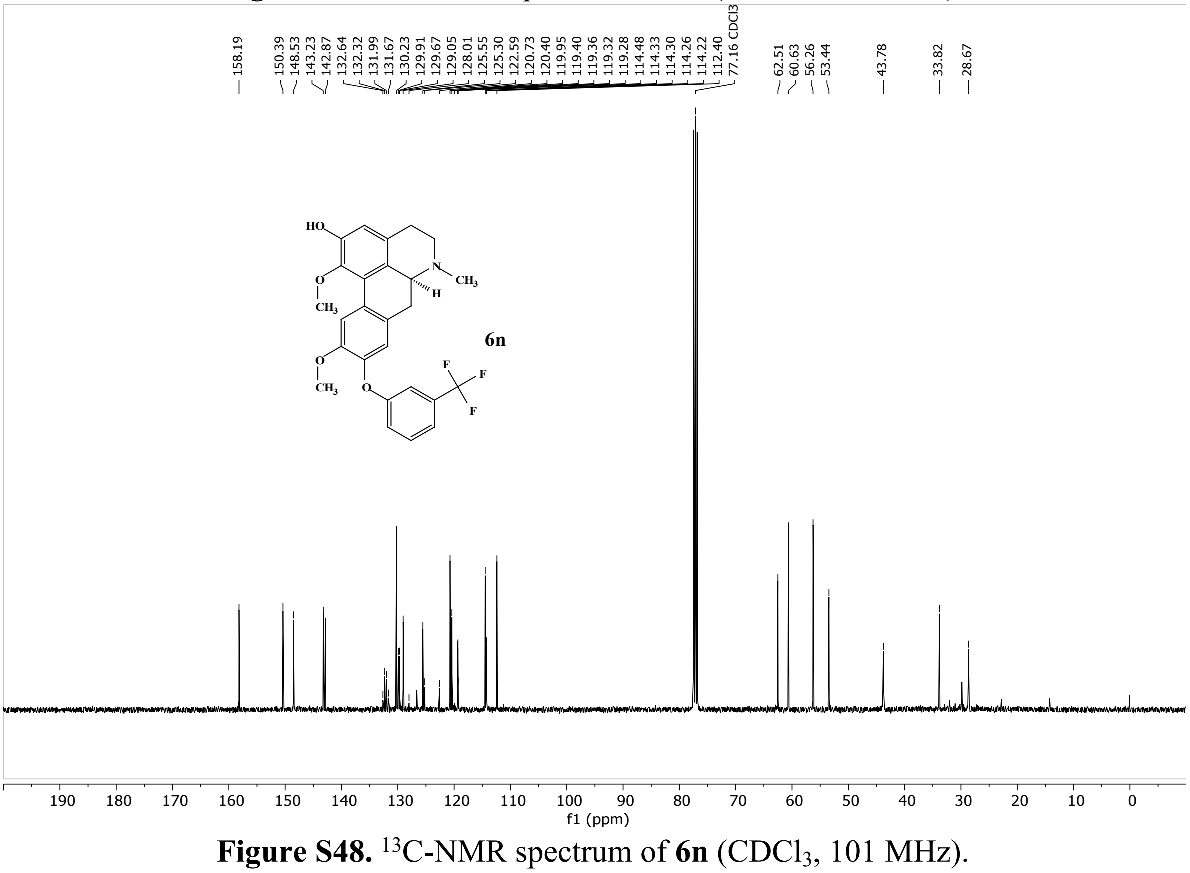

Figure S48. ^{13}C-NMR spectrum of **6n** ($CDCl_3$, 101 MHz).

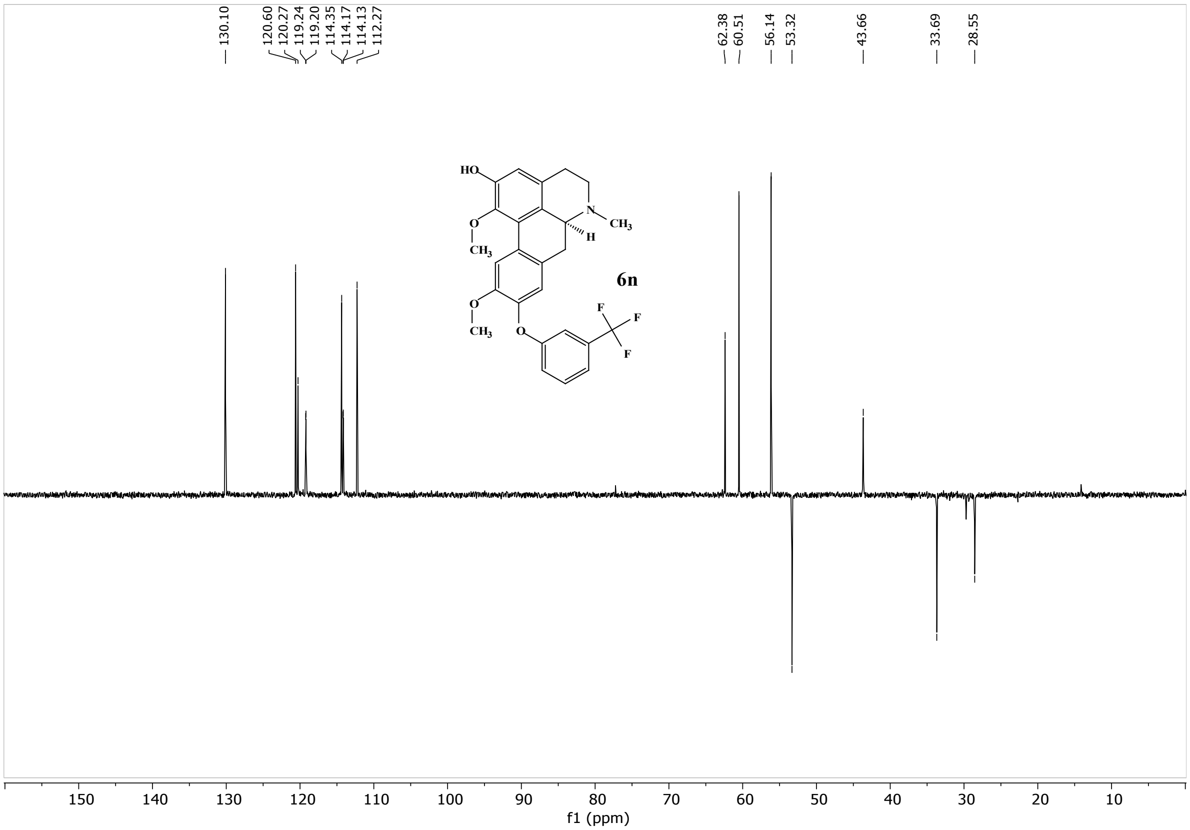

Figure S49. DEPT spectrum of **6n** ($CDCl_3$, 101 MHz).

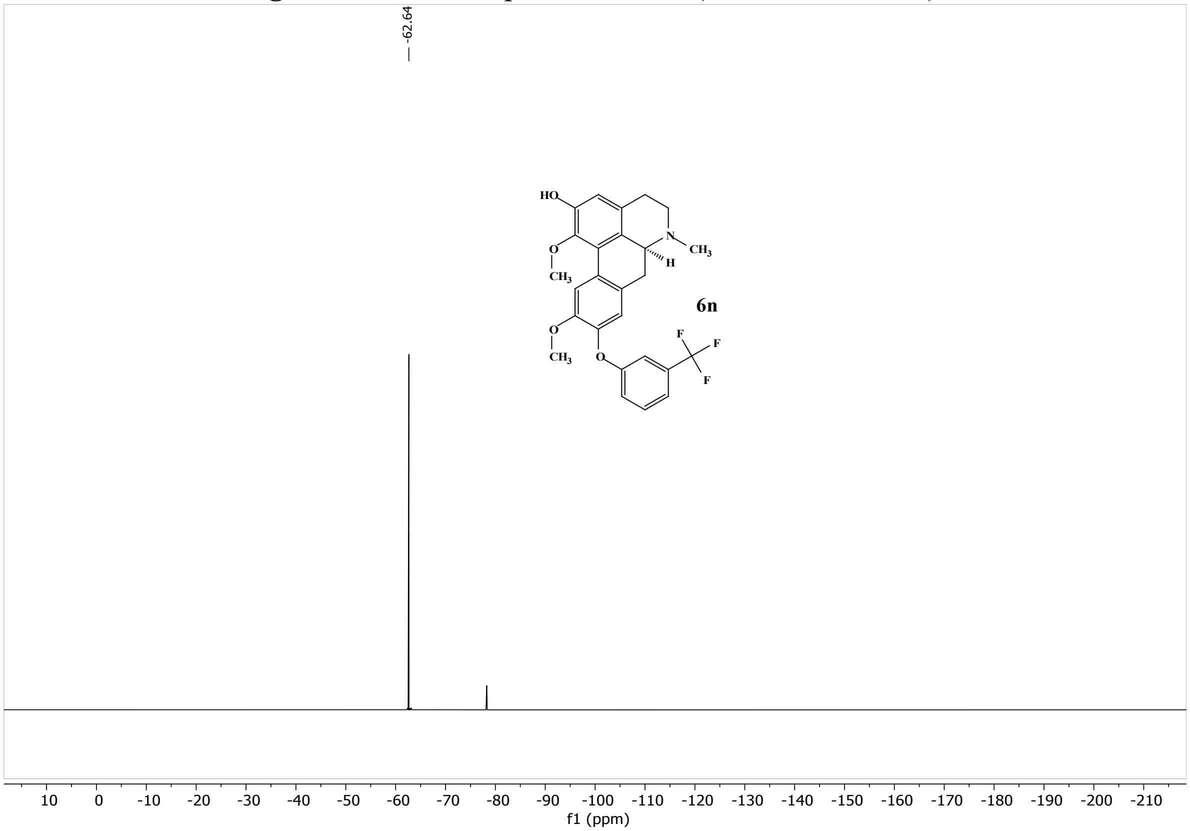

Figure S50. ^{19}F-NMR spectrum of **6n** ($CDCl_3$, 376 MHz).

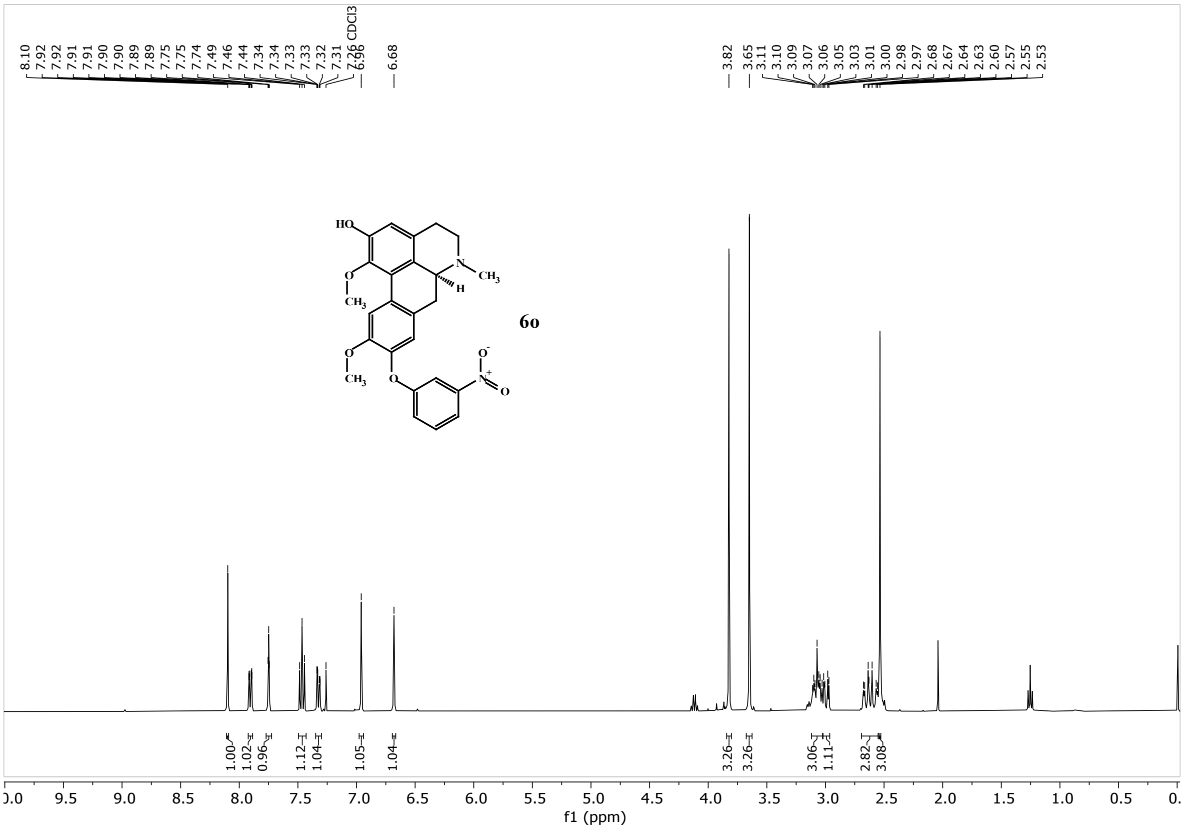

Figure S51. ^{1}H-NMR spectrum of **6o** ($CDCl_3$, 400 MHz).

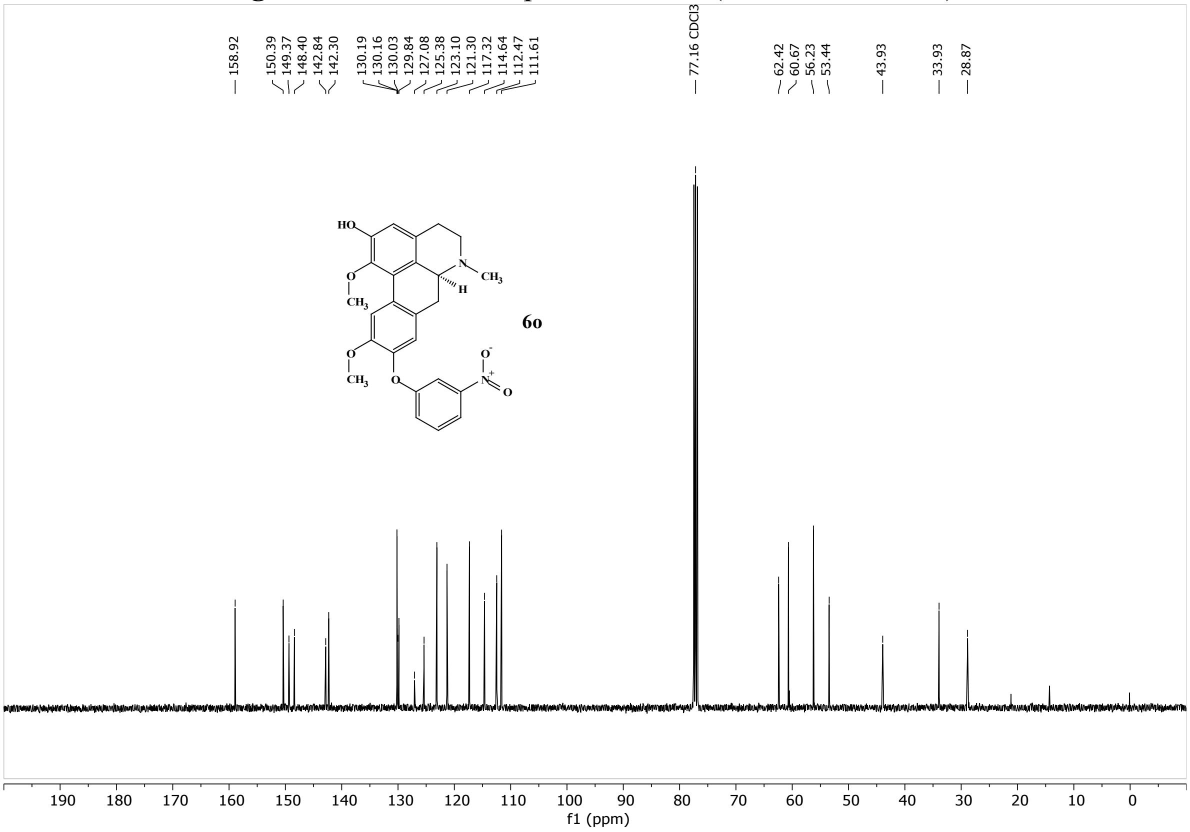

Figure S52. ^{13}C-NMR spectrum of **6o** ($CDCl_3$, 101 MHz).

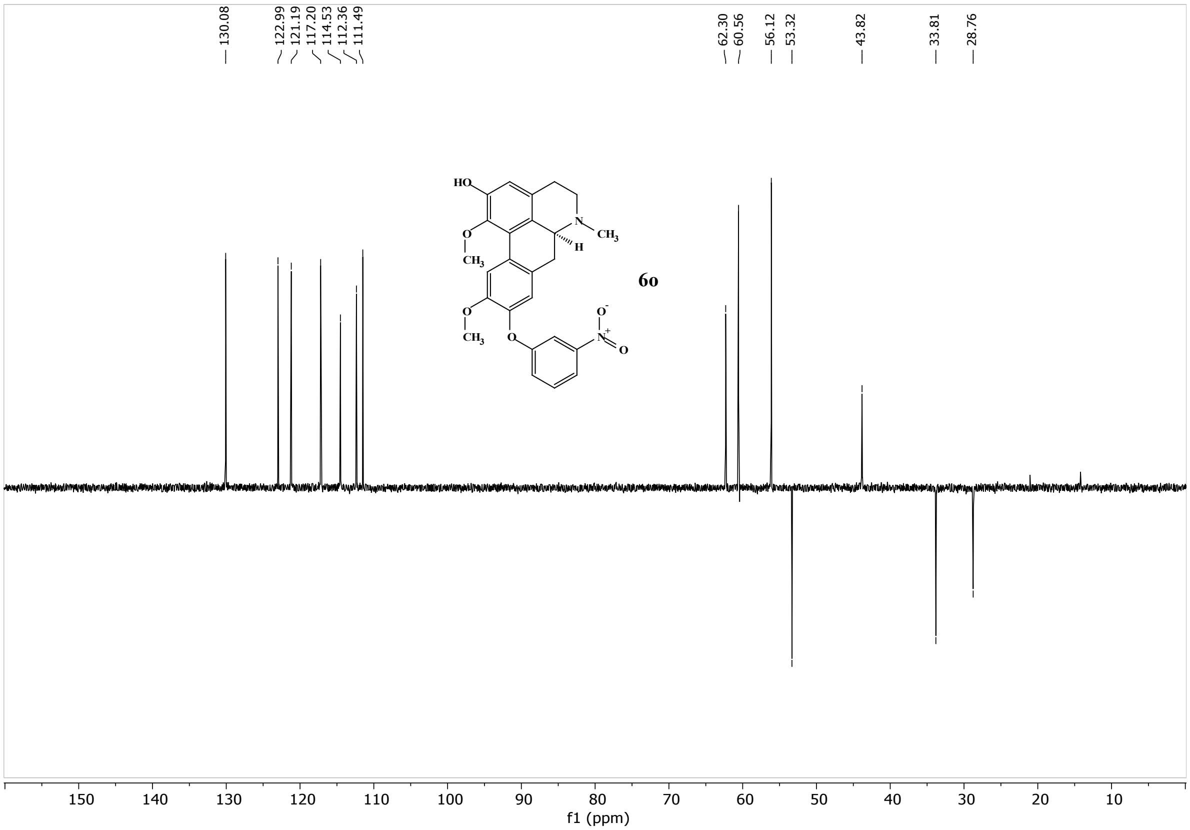

Figure S53. DEPT spectrum of **6o** ($CDCl_3$, 101 MHz).

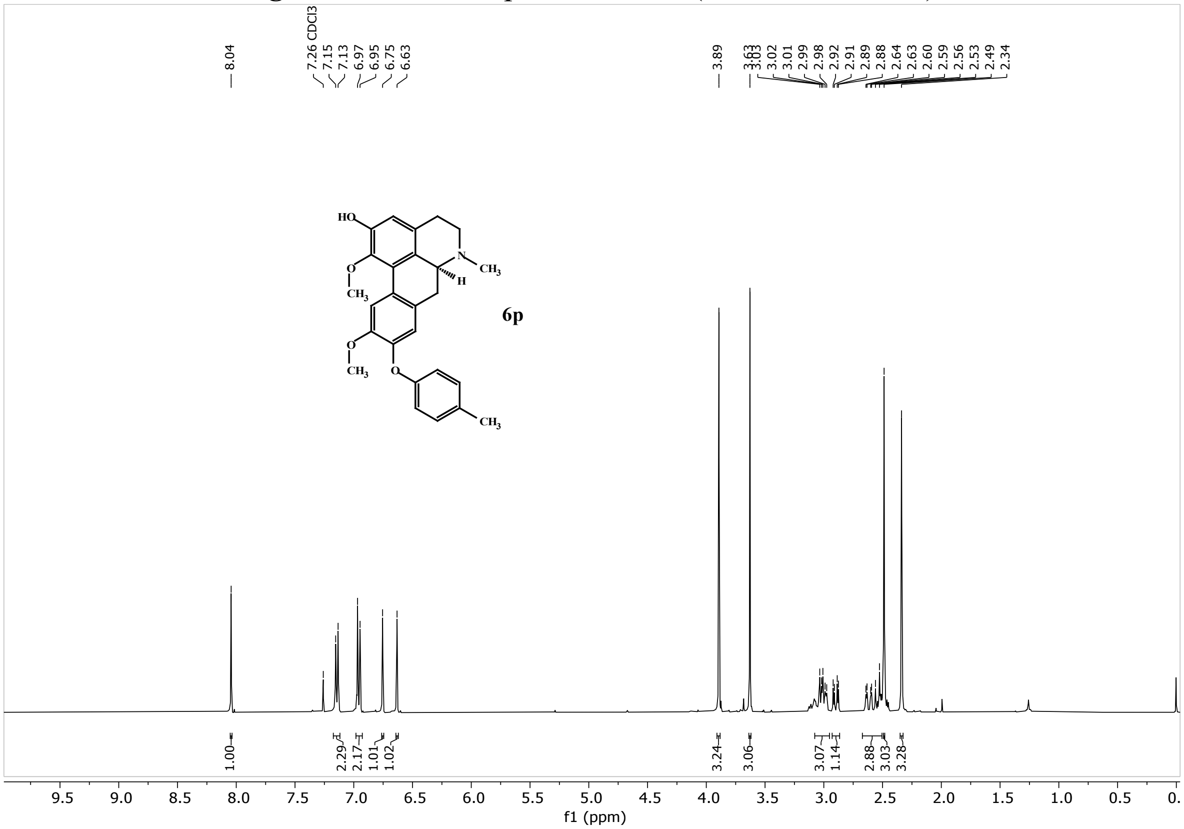

Figure S54. ^{1}H-NMR spectrum of **6p** ($CDCl_3$, 400 MHz).

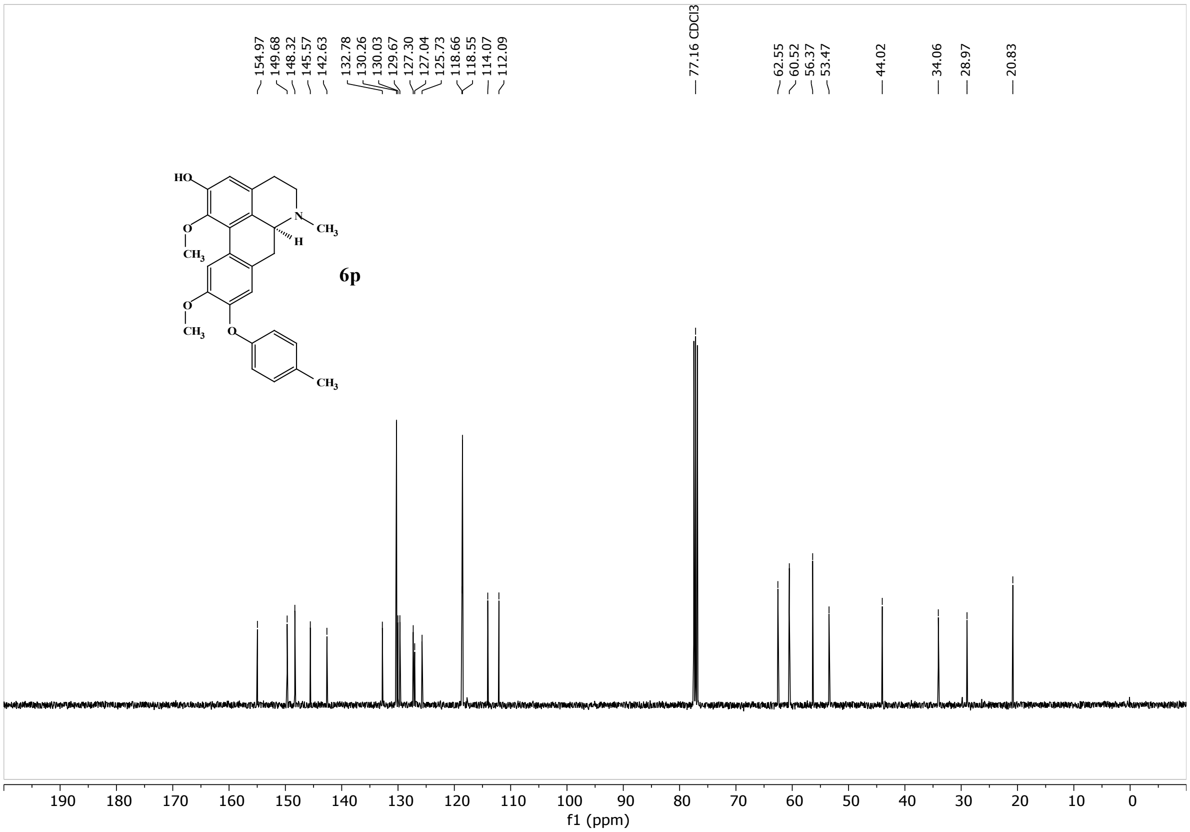

Figure S55. ^{13}C-NMR spectrum of **6p** ($CDCl_3$, 101 MHz).

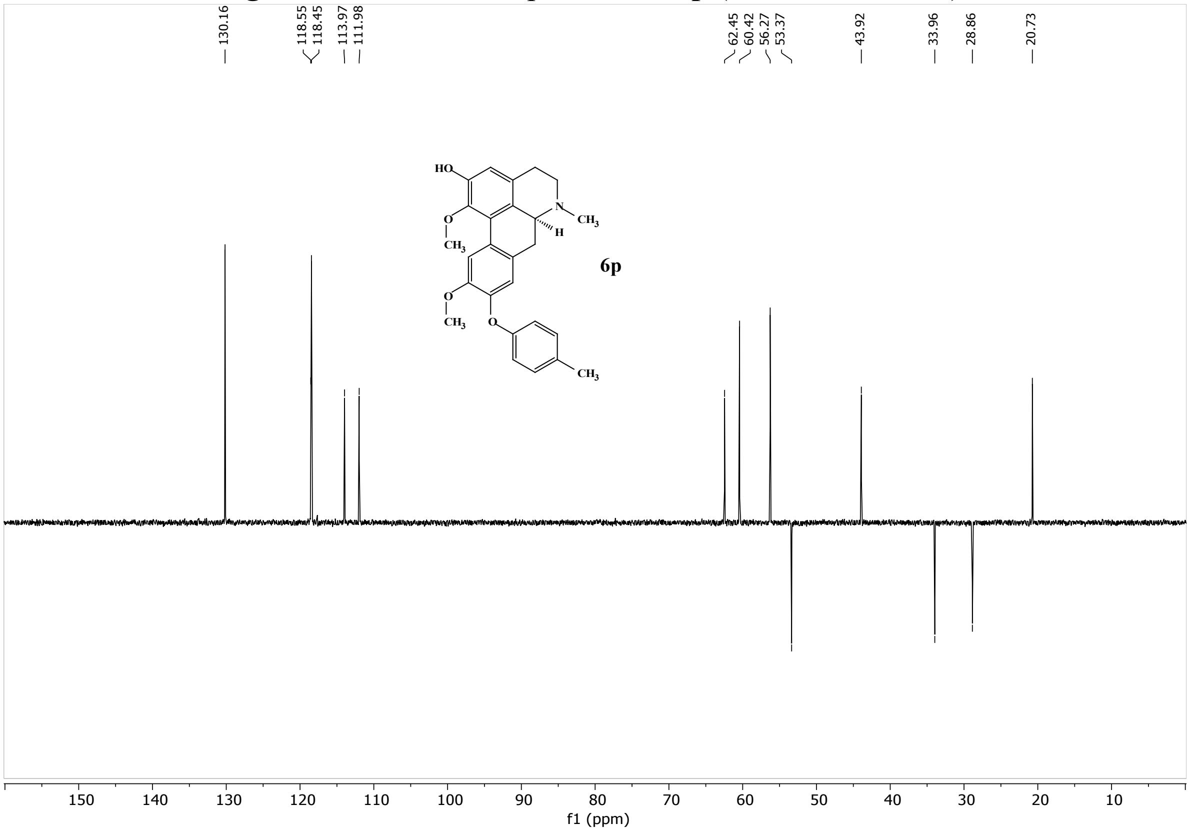

Figure S56. DEPT spectrum of **6p** ($CDCl_3$, 101 MHz).

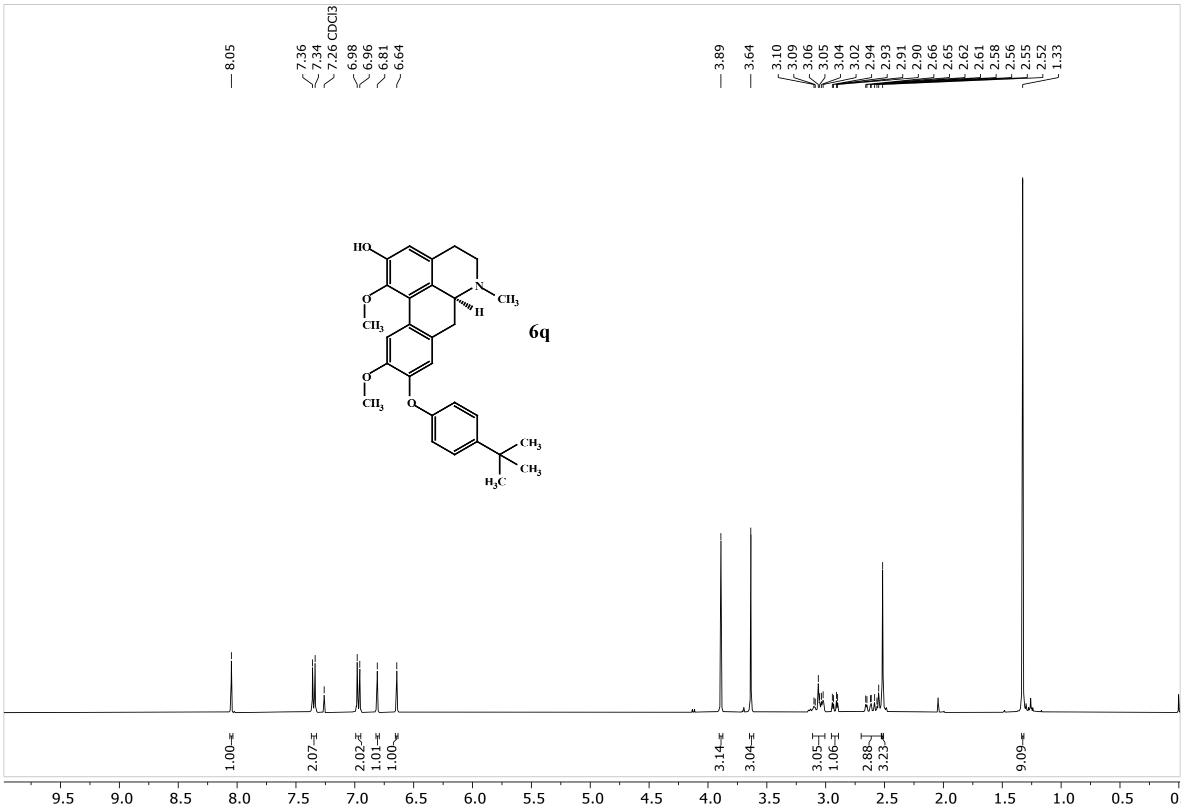

Figure S57. ^{1}H-NMR spectrum of **6q** ($CDCl_3$, 400 MHz).

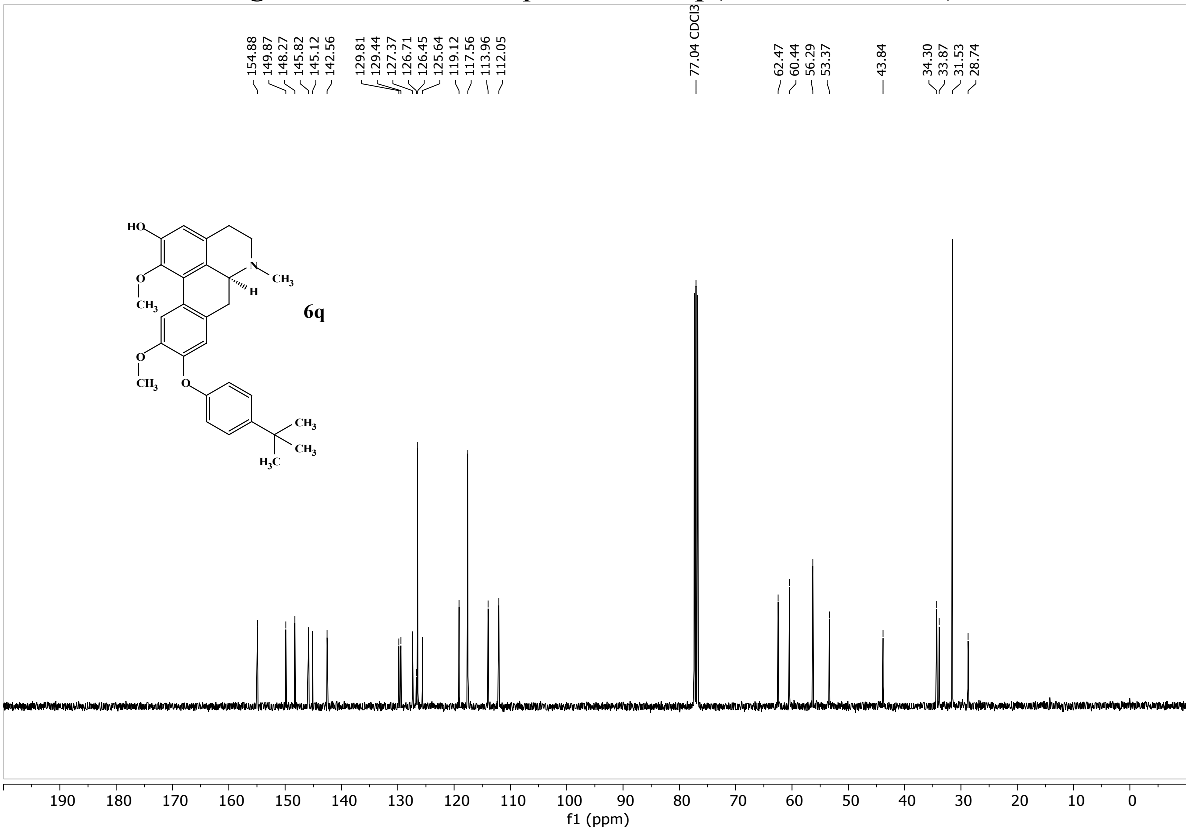

Figure S58. ^{13}C-NMR spectrum of **6q** ($CDCl_3$, 101 MHz).

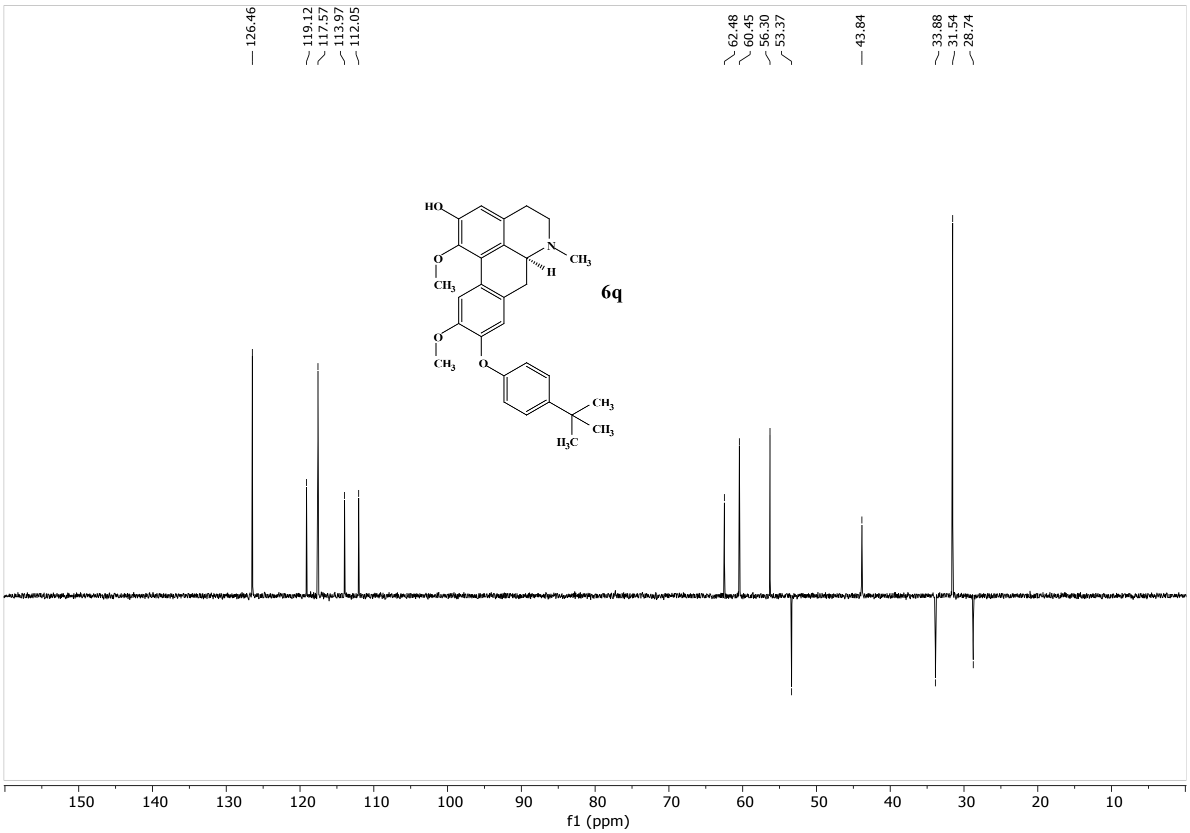

Figure S59. DEPT spectrum of **6q** ($CDCl_3$, 101 MHz).

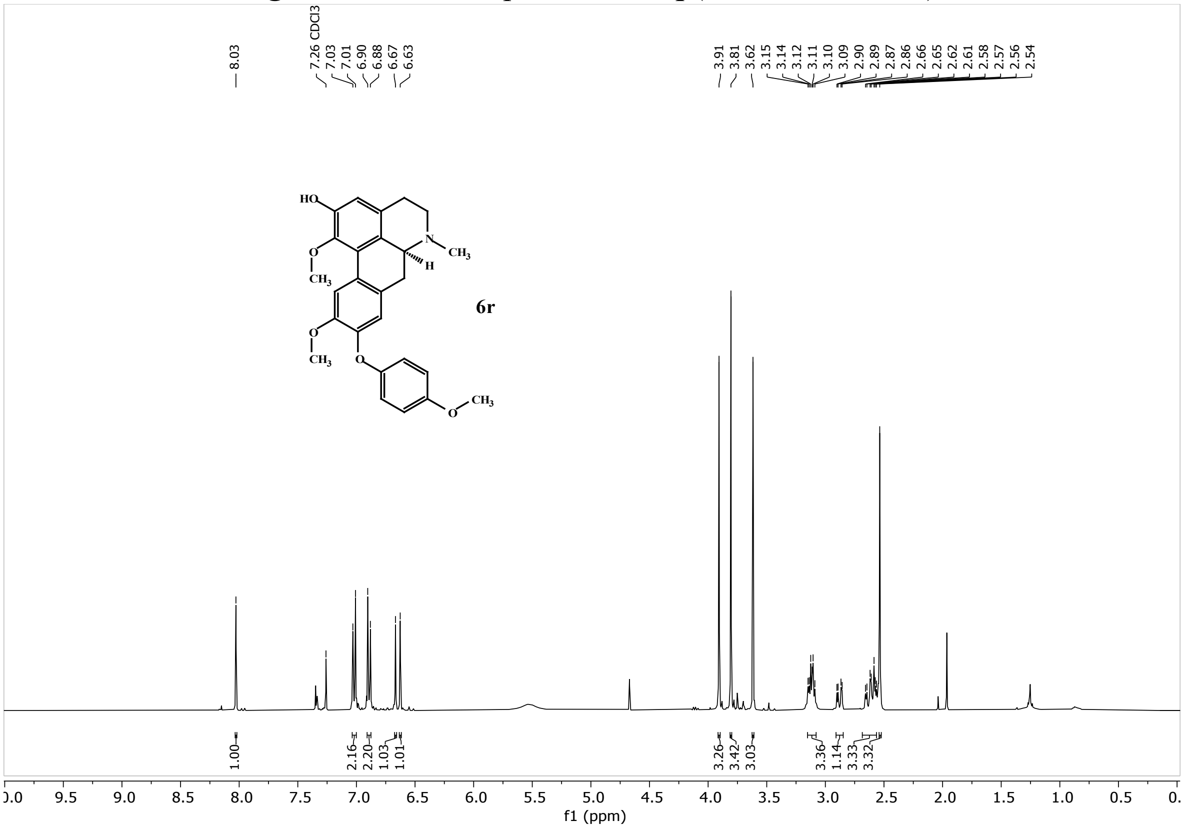

Figure S60. ^{1}H-NMR spectrum of **6r** ($CDCl_3$, 400 MHz).

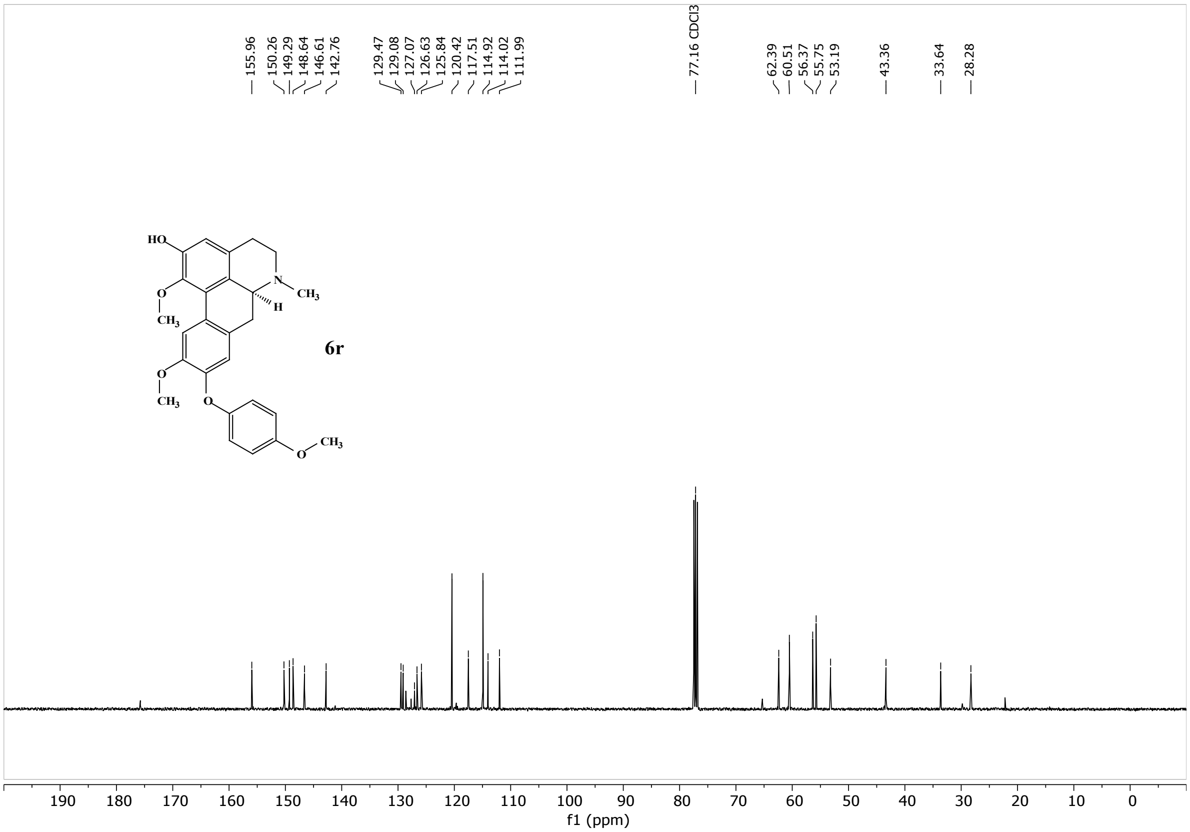

Figure S61. ^{13}C-NMR spectrum of **6r** ($CDCl_3$, 101 MHz).

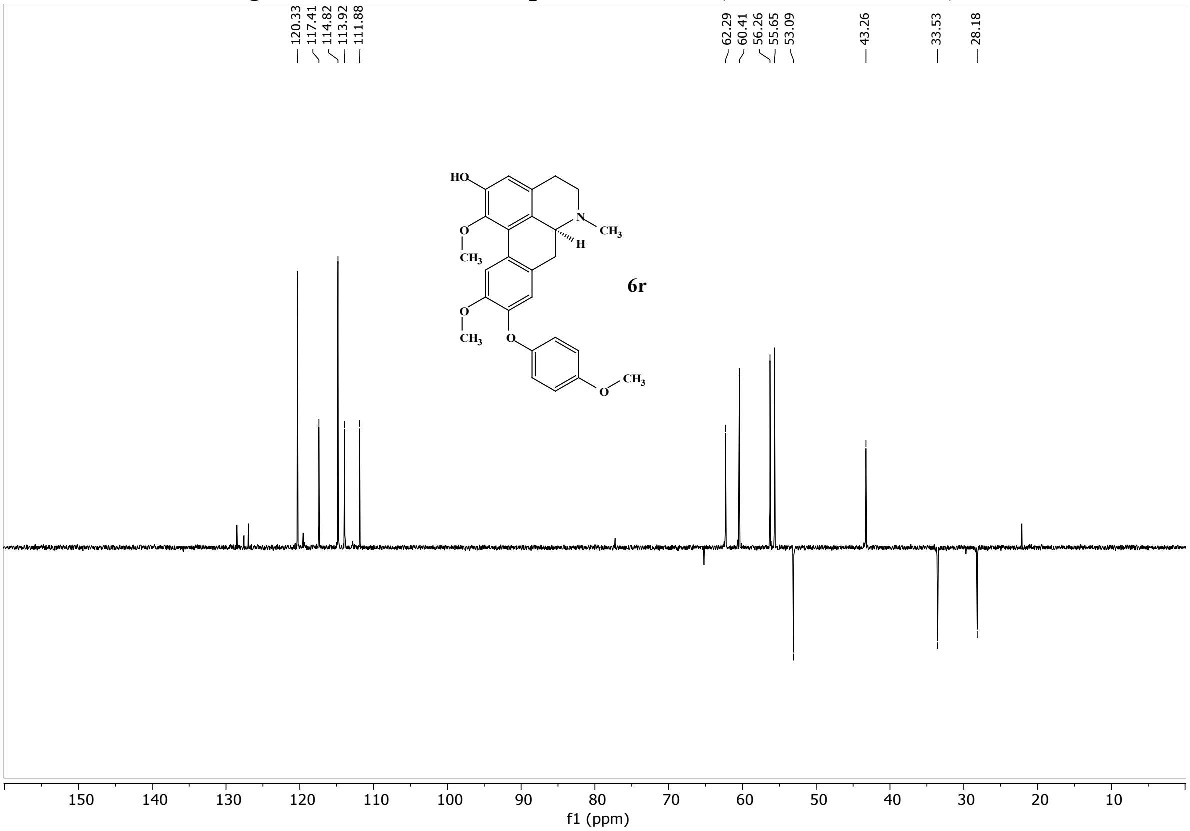

Figure S62. DEPT spectrum of **6r** ($CDCl_3$, 101 MHz).

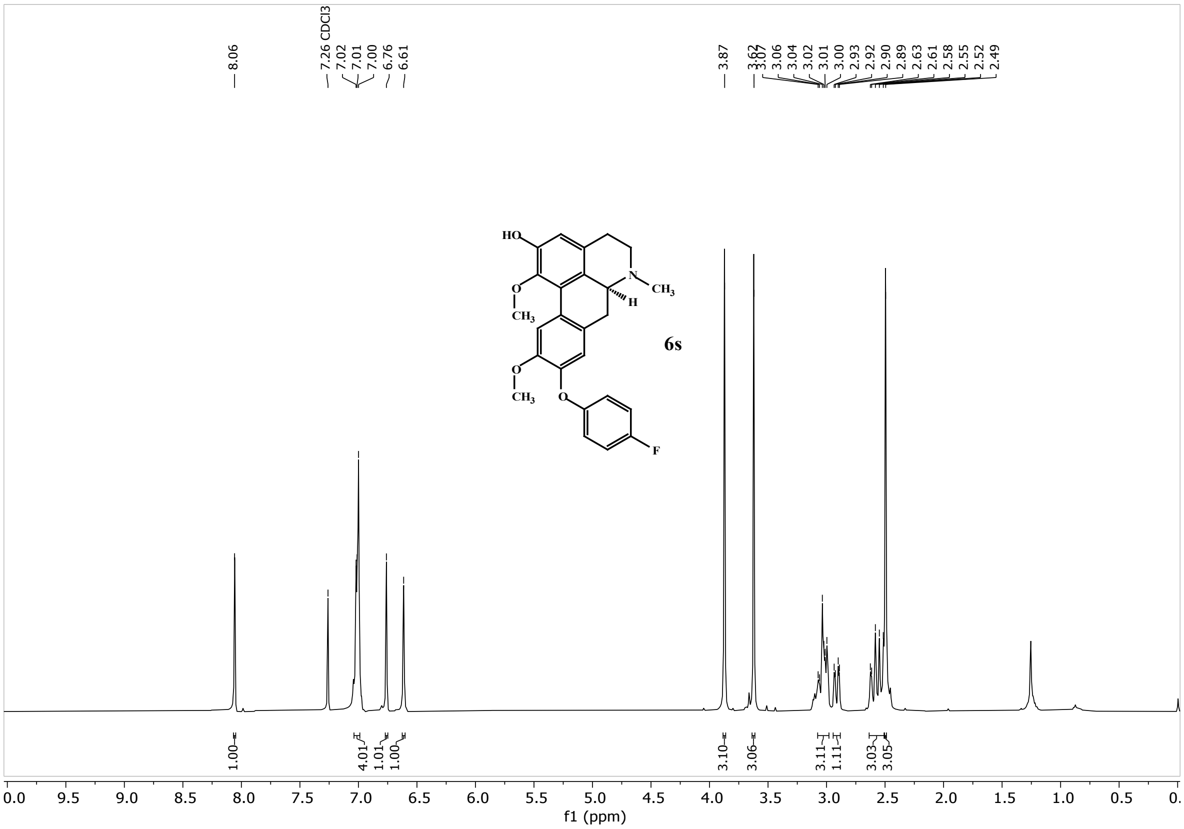

Figure S63. ^{1}H-NMR spectrum of **6s** ($CDCl_3$, 400 MHz).

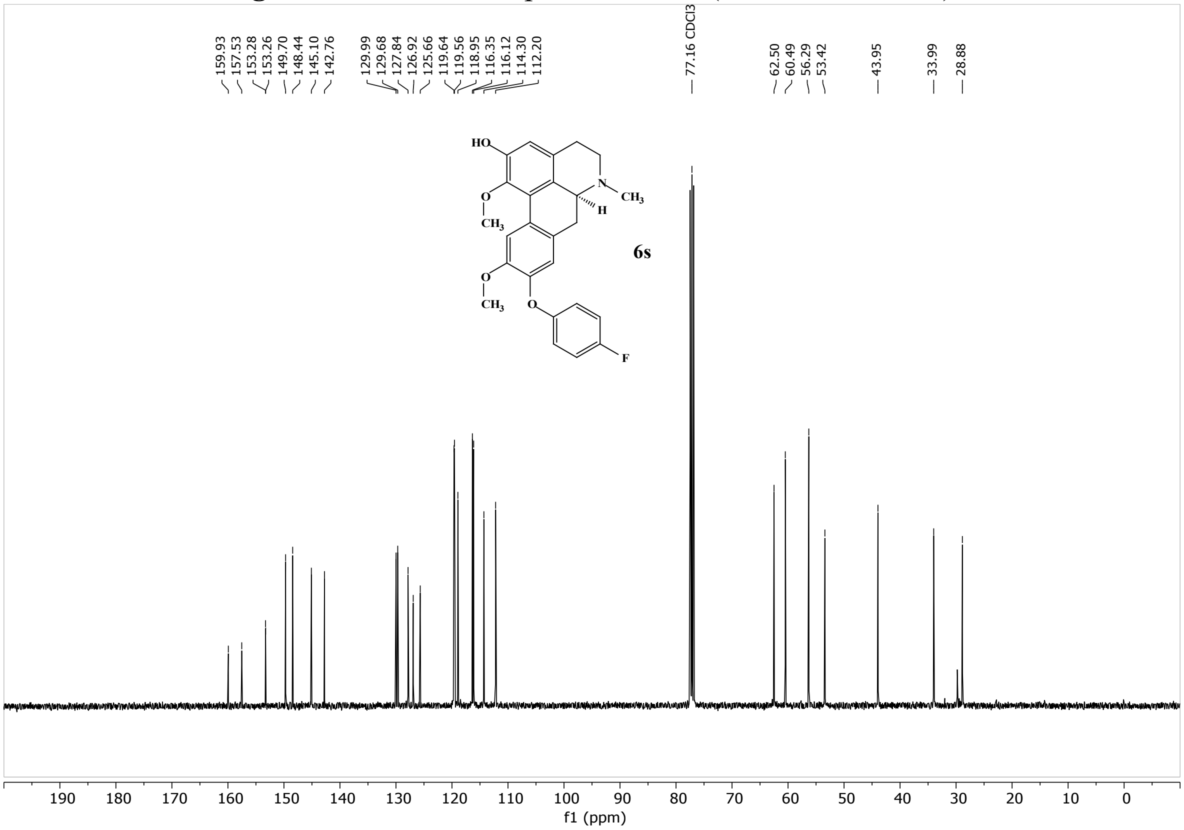

Figure S64. ^{13}C-NMR spectrum of **6s** ($CDCl_3$, 101 MHz).

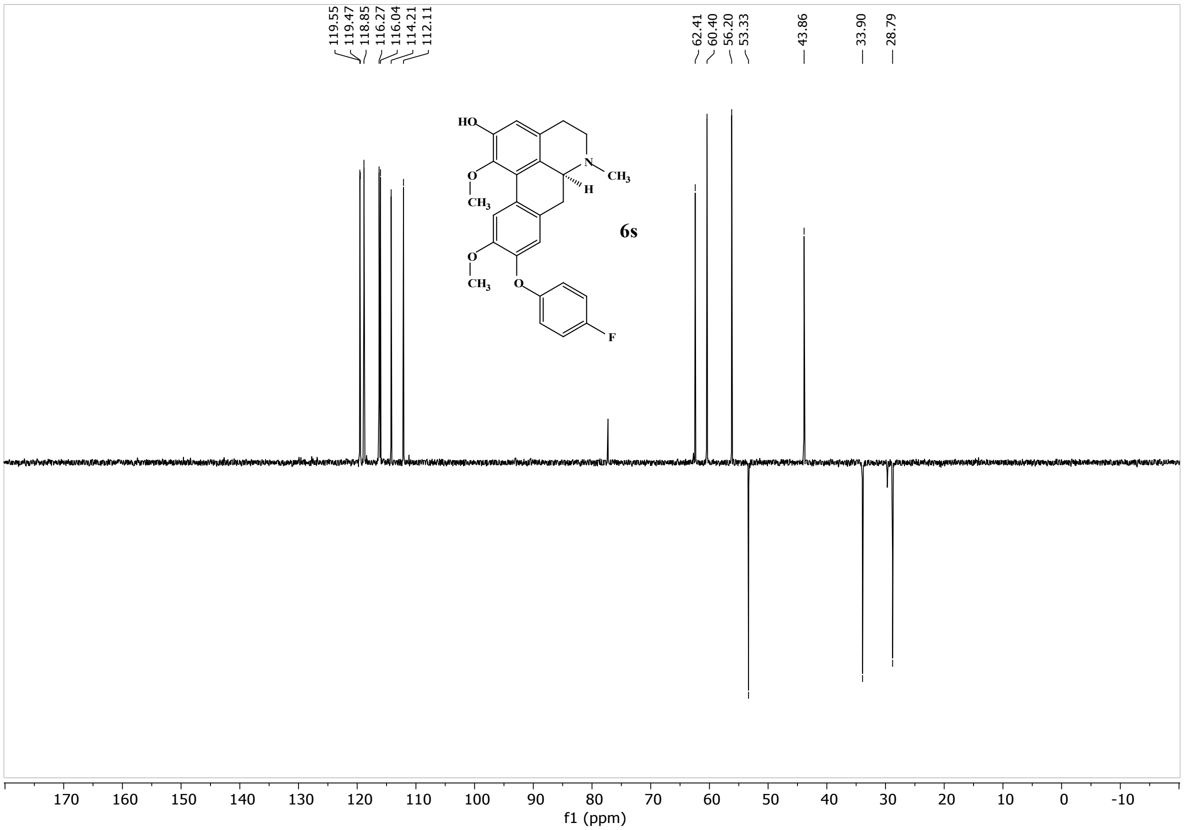

Figure S65. DEPT spectrum of **6s** ($CDCl_3$, 101 MHz).

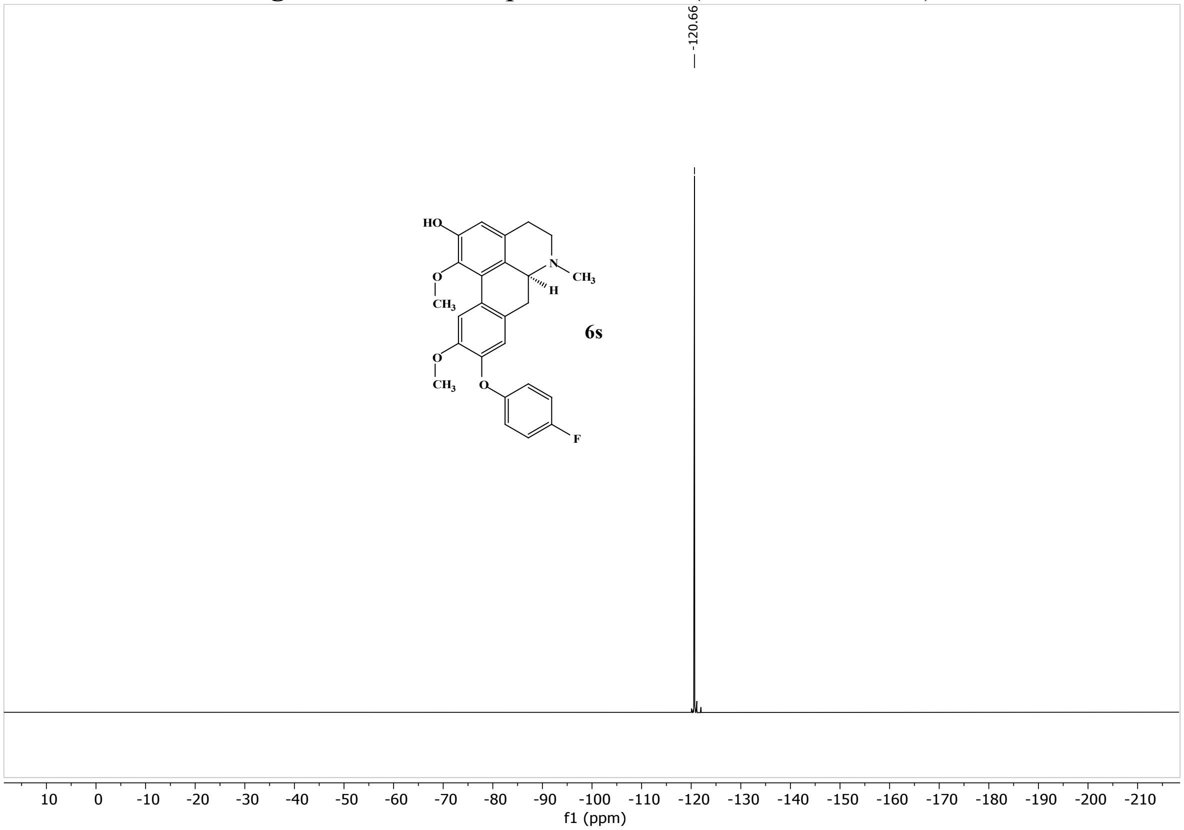

Figure S66. ^{19}F-NMR spectrum of **6s** ($CDCl_3$, 376 MHz).

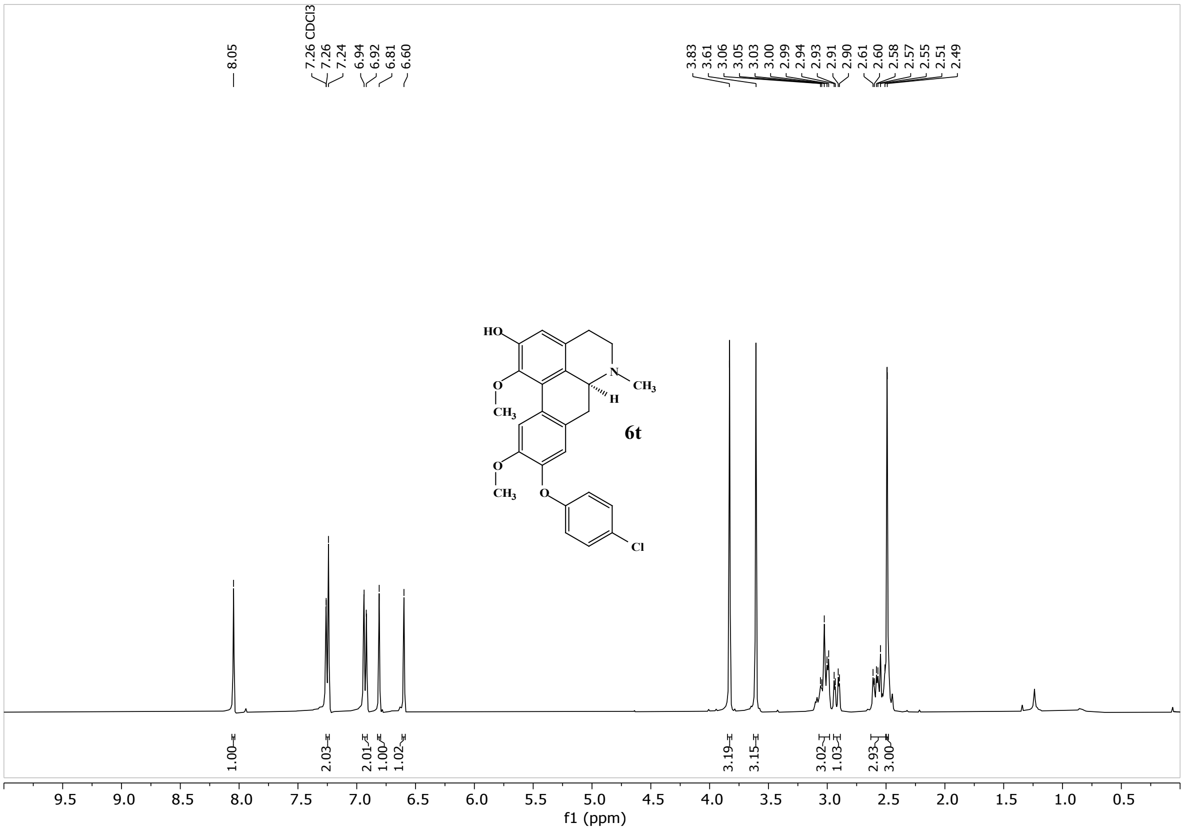

Figure S67. ^{1}H-NMR spectrum of **6t** ($CDCl_3$, 400 MHz).

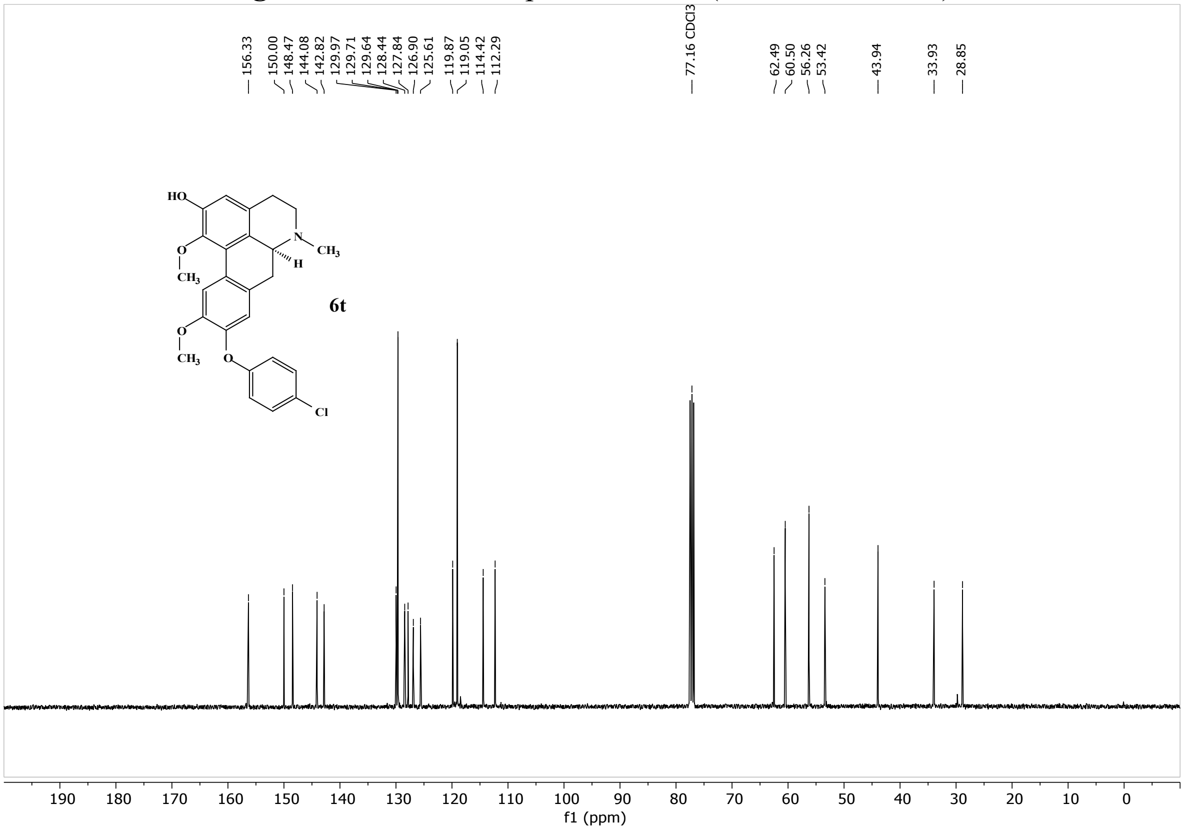

Figure S68. ^{13}C-NMR spectrum of **6t** ($CDCl_3$, 101 MHz).

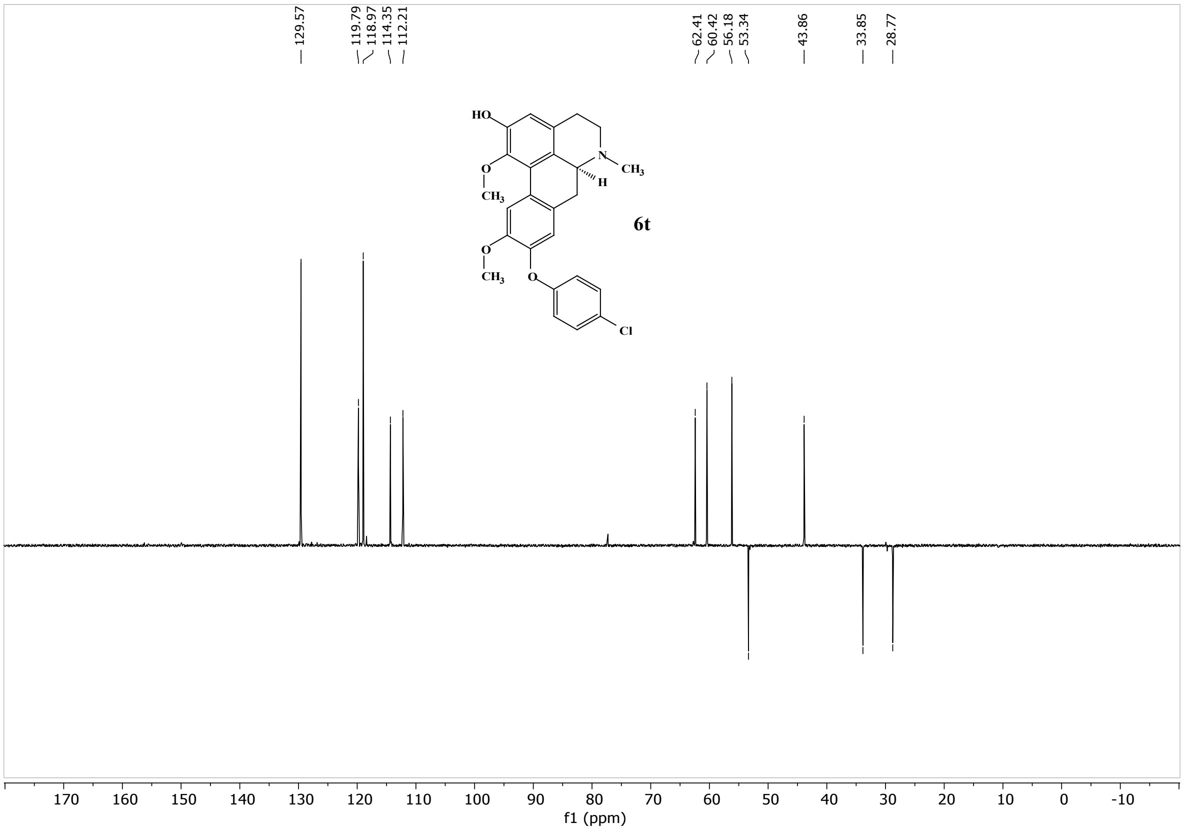

Figure S69. DEPT spectrum of **6t** ($CDCl_3$, 101 MHz).

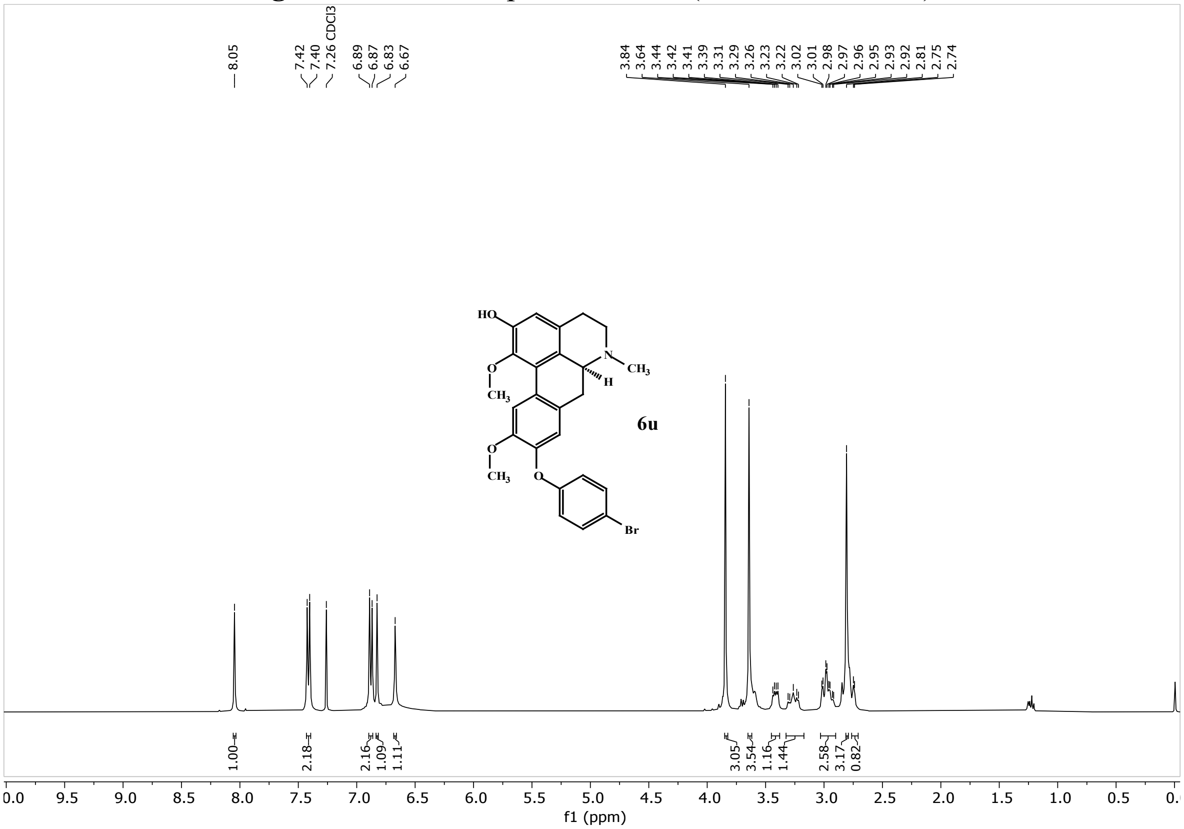

Figure S70. ^{1}H-NMR spectrum of **6u** ($CDCl_3$, 400 MHz).

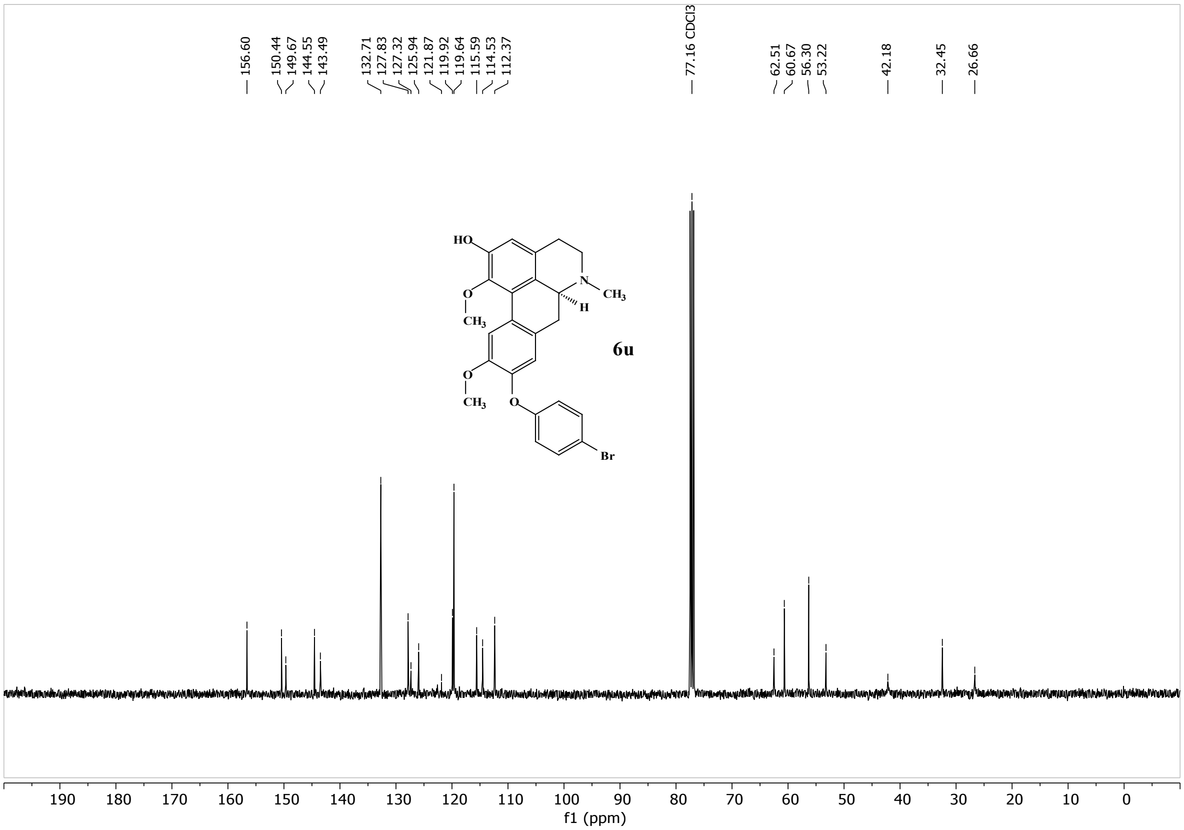

Figure S71. ^{13}C-NMR spectrum of **6u** ($CDCl_3$, 101 MHz).

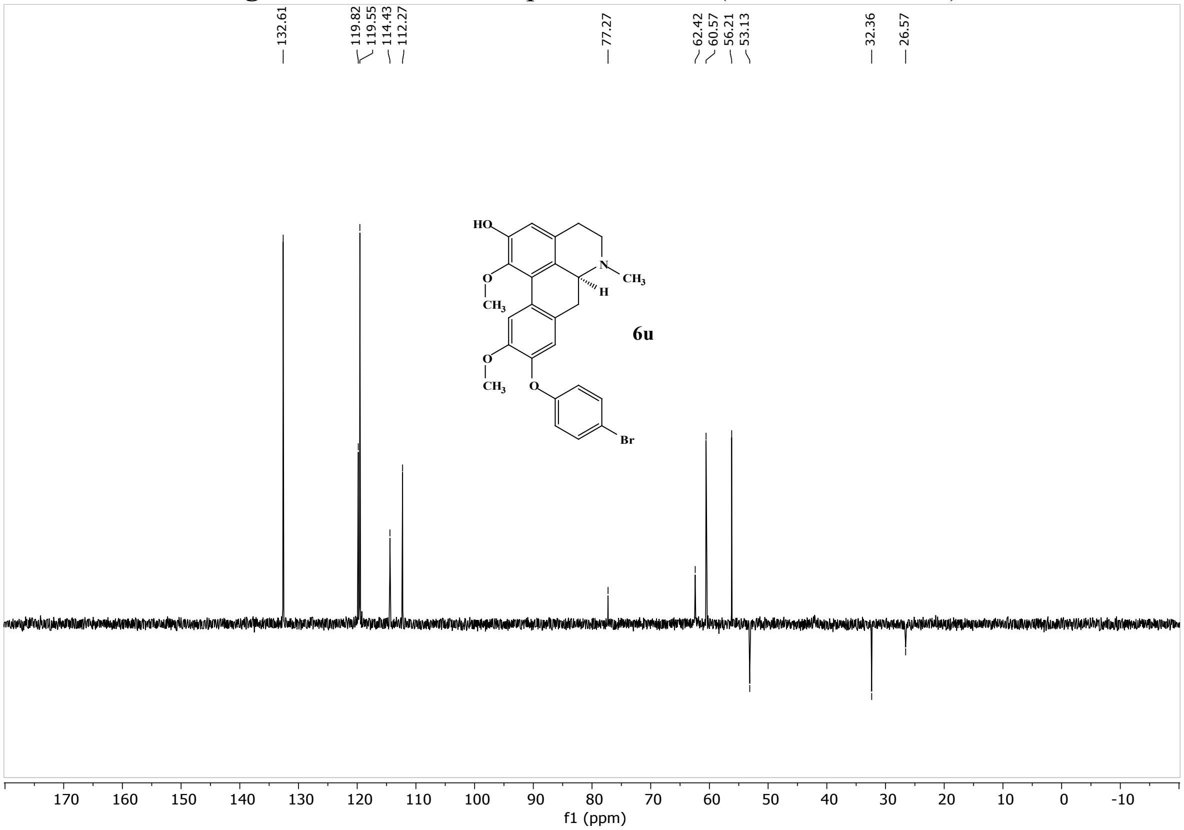

Figure S72. DEPT spectrum of **6u** ($CDCl_3$, 101 MHz).

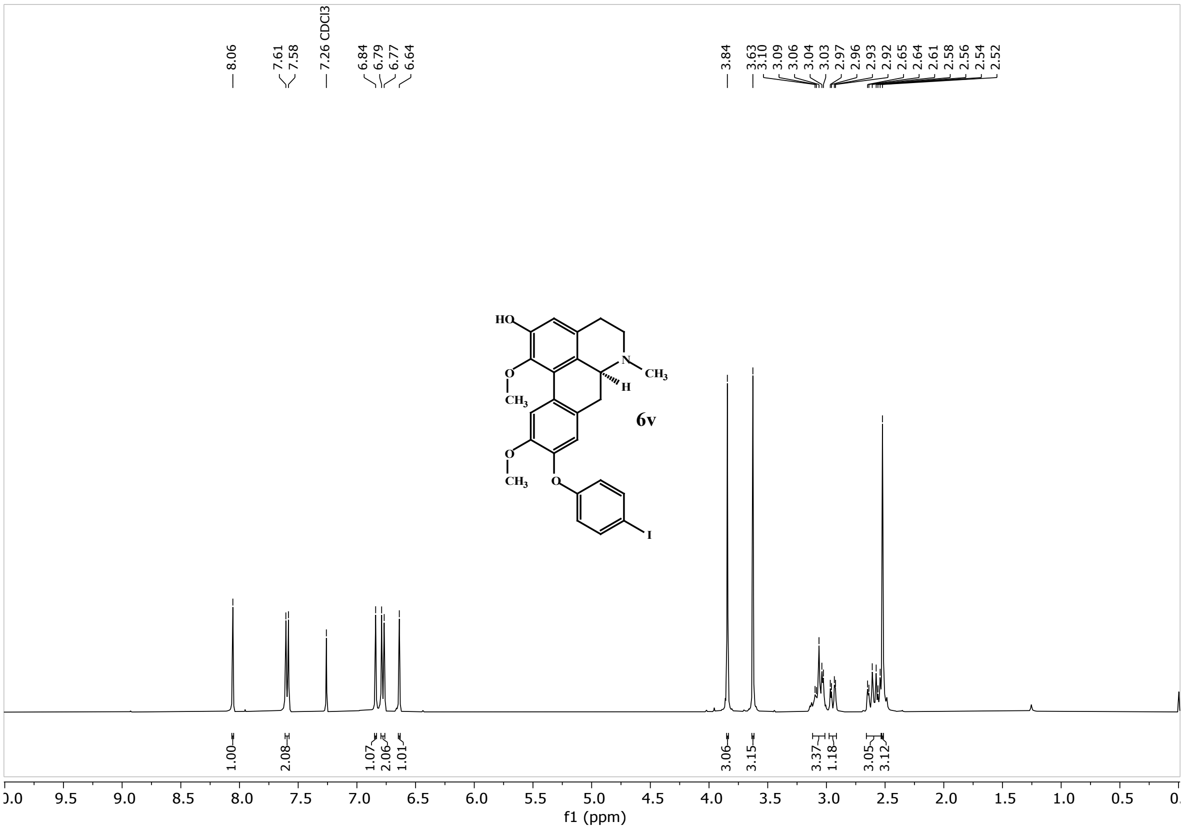

Figure S73. ^{1}H-NMR spectrum of **6v** ($CDCl_3$, 400 MHz).

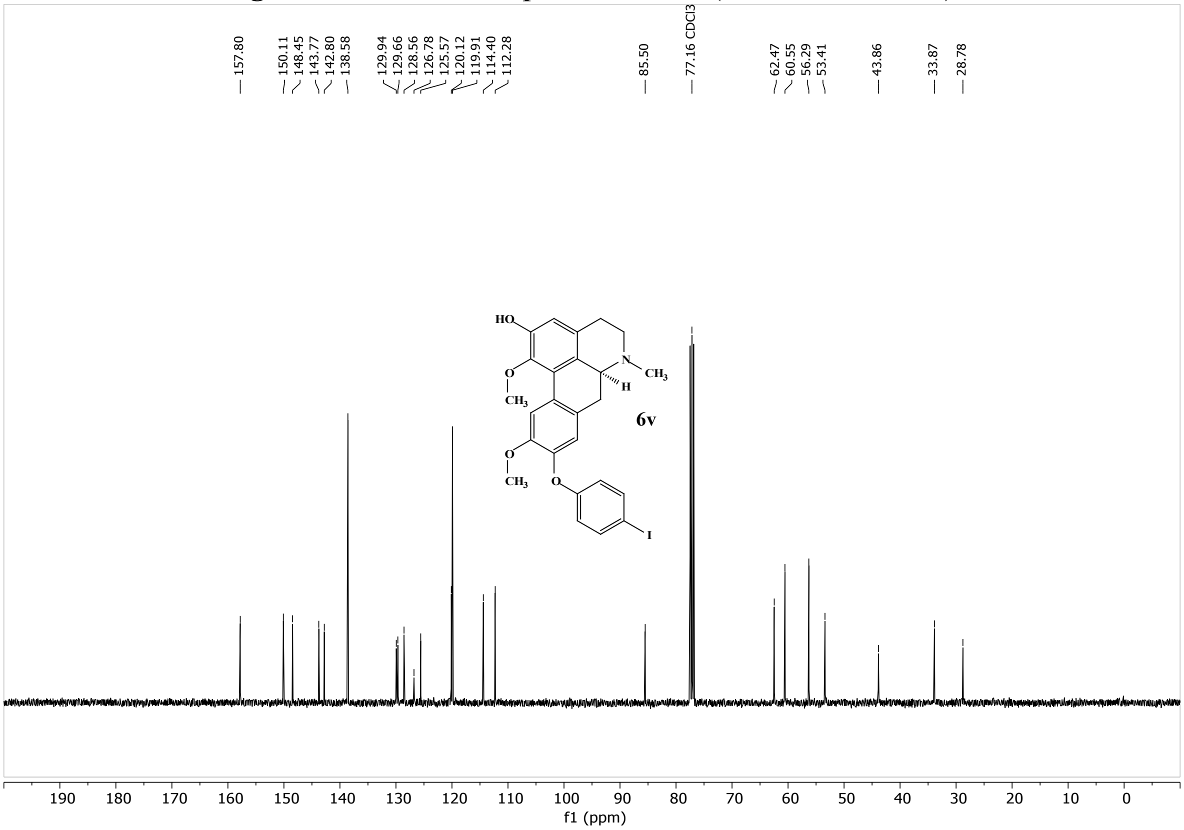

Figure S74. ^{13}C-NMR spectrum of **6v** ($CDCl_3$, 101 MHz).

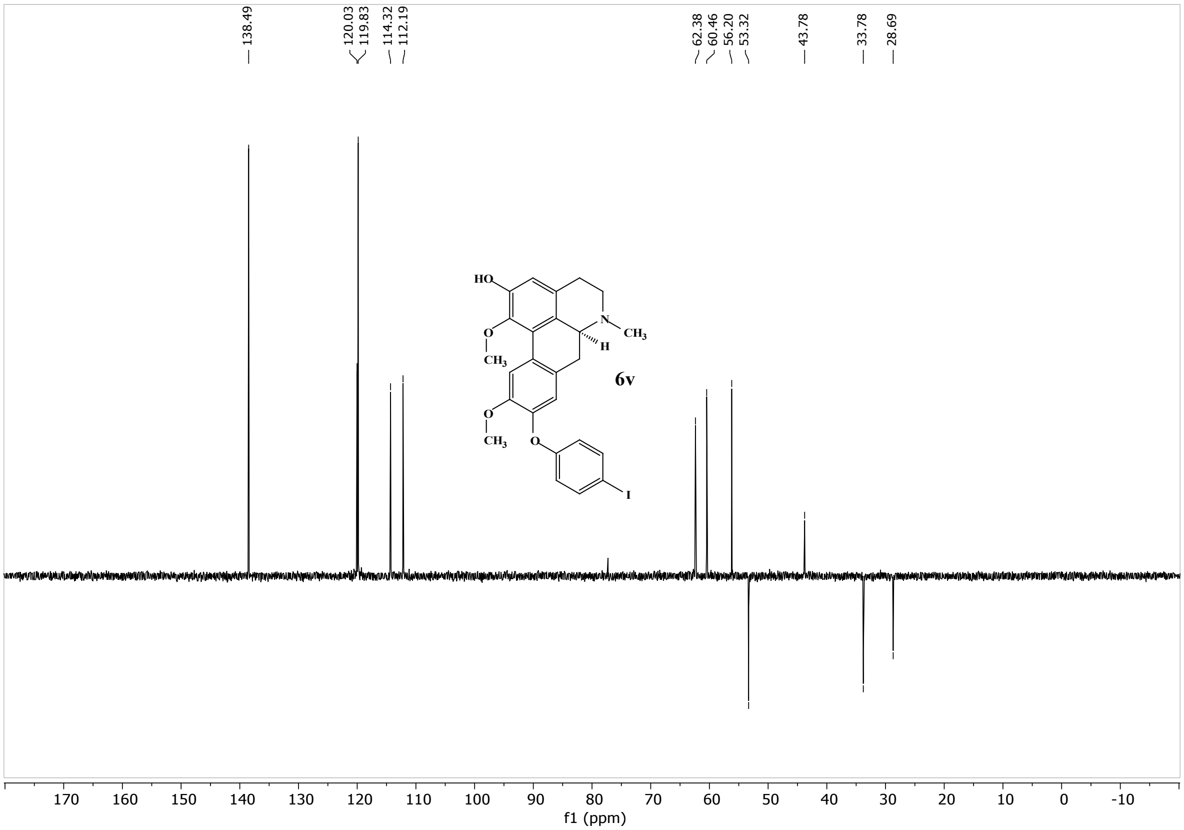

Figure S75. DEPT spectrum of **6v** ($CDCl_3$, 101 MHz).

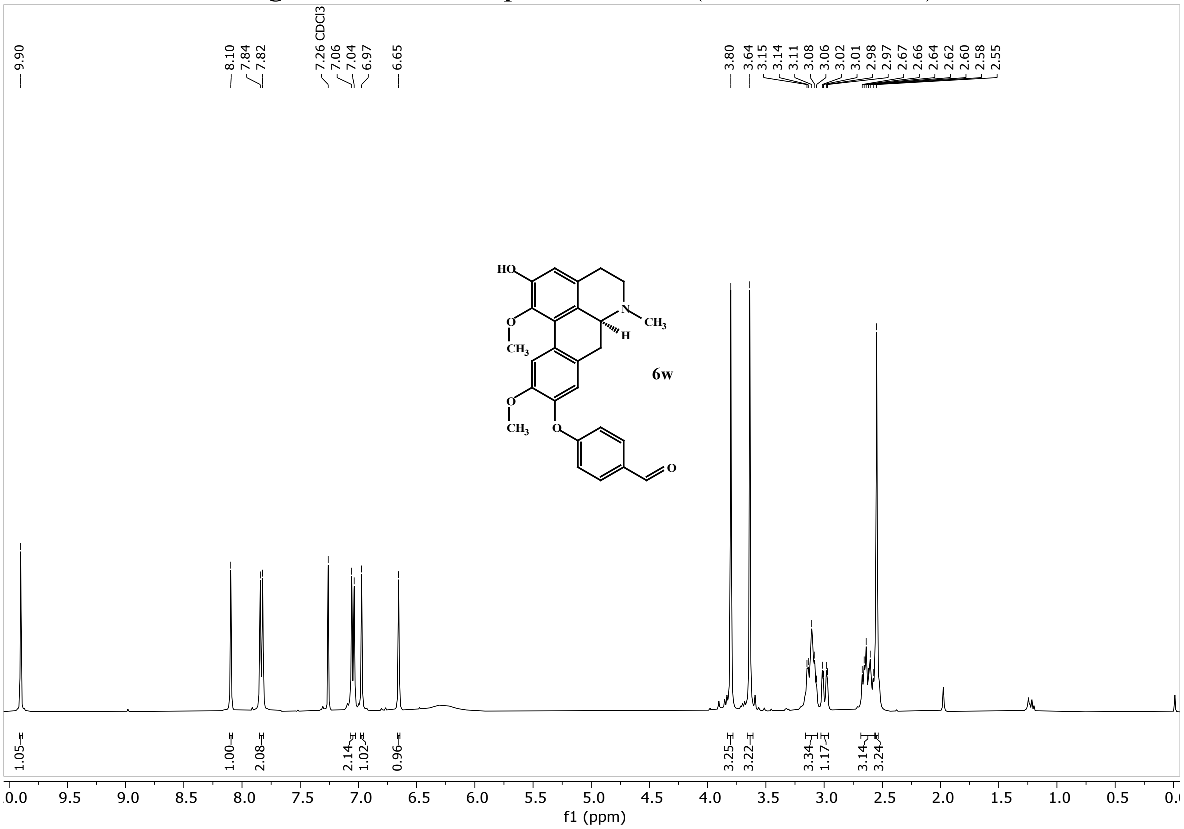

Figure S76. ^{1}H-NMR spectrum of **6w** ($CDCl_3$, 400 MHz).

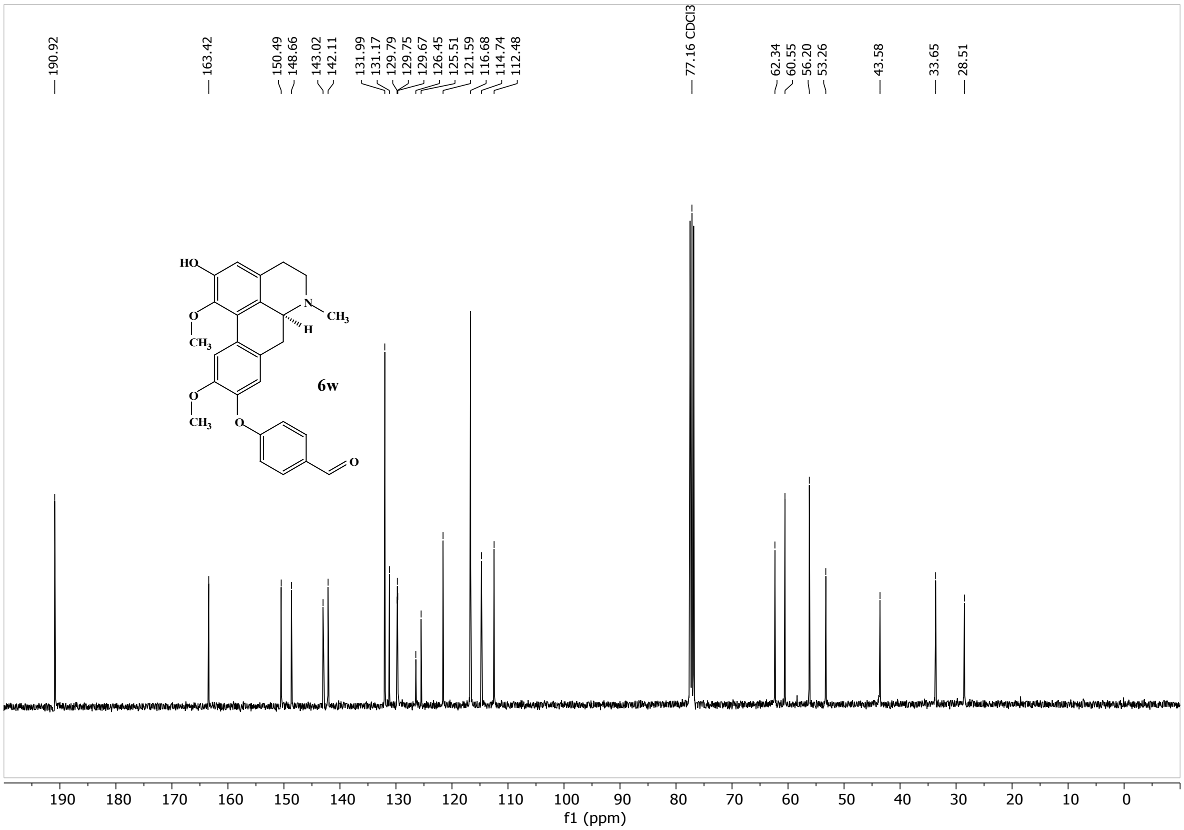

Figure S77. ^{13}C-NMR spectrum of **6w** ($CDCl_3$, 101 MHz).

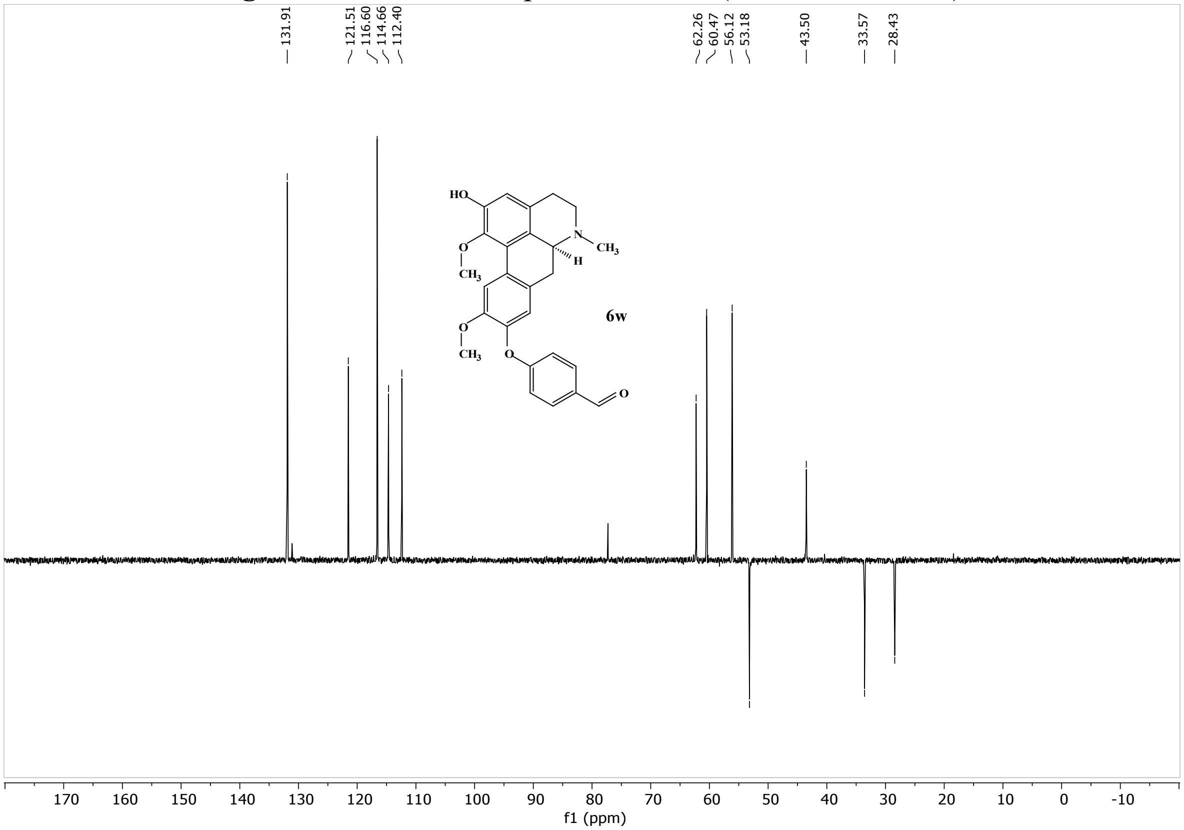

Figure S78. DEPT spectrum of **6w** ($CDCl_3$, 101 MHz).

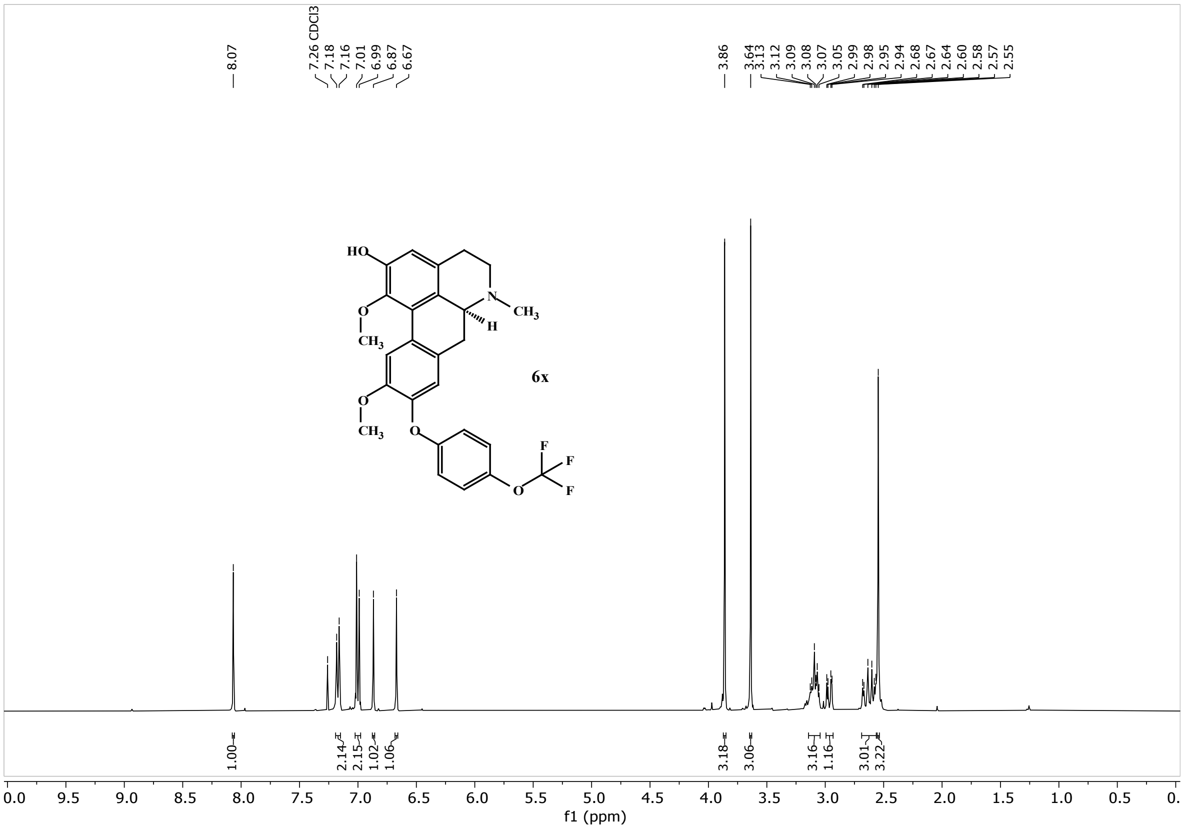

Figure S79. ^{1}H-NMR spectrum of **6x** ($CDCl_3$, 400 MHz).

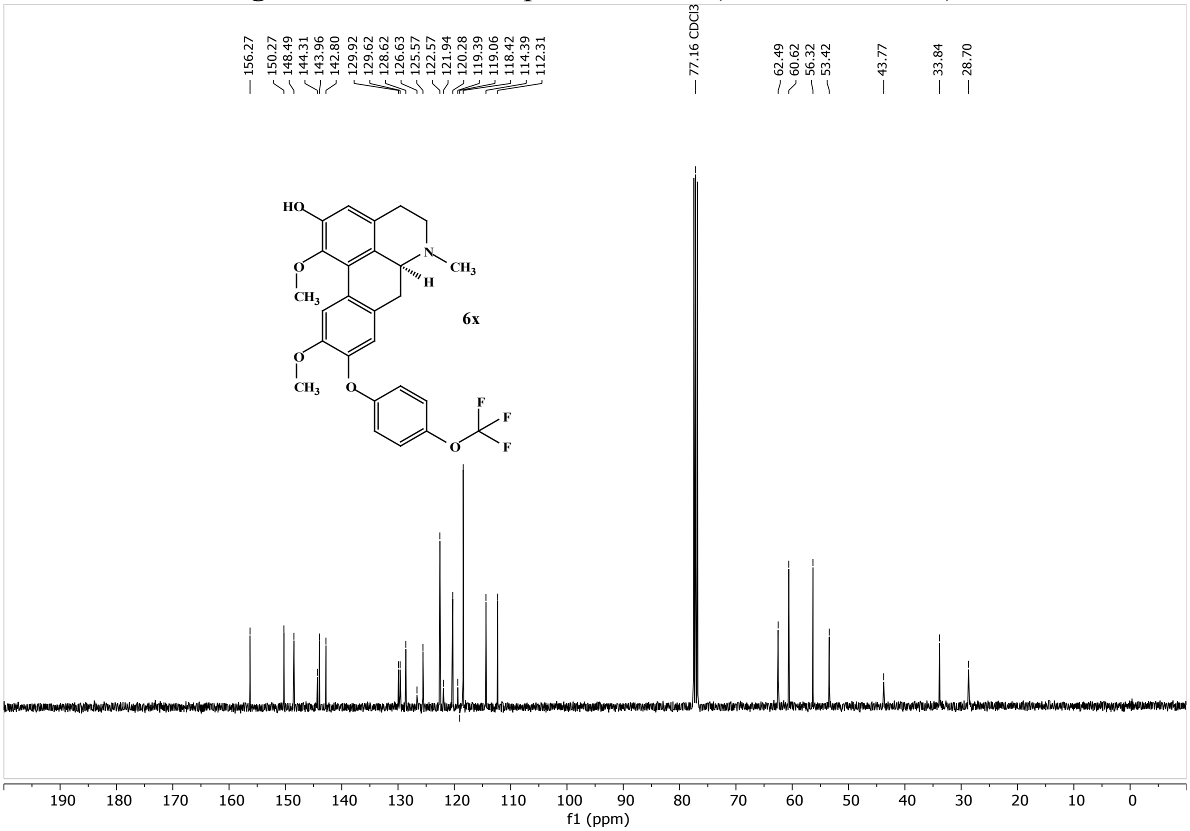

Figure S80. ^{13}C-NMR spectrum of **6x** ($CDCl_3$, 101 MHz).

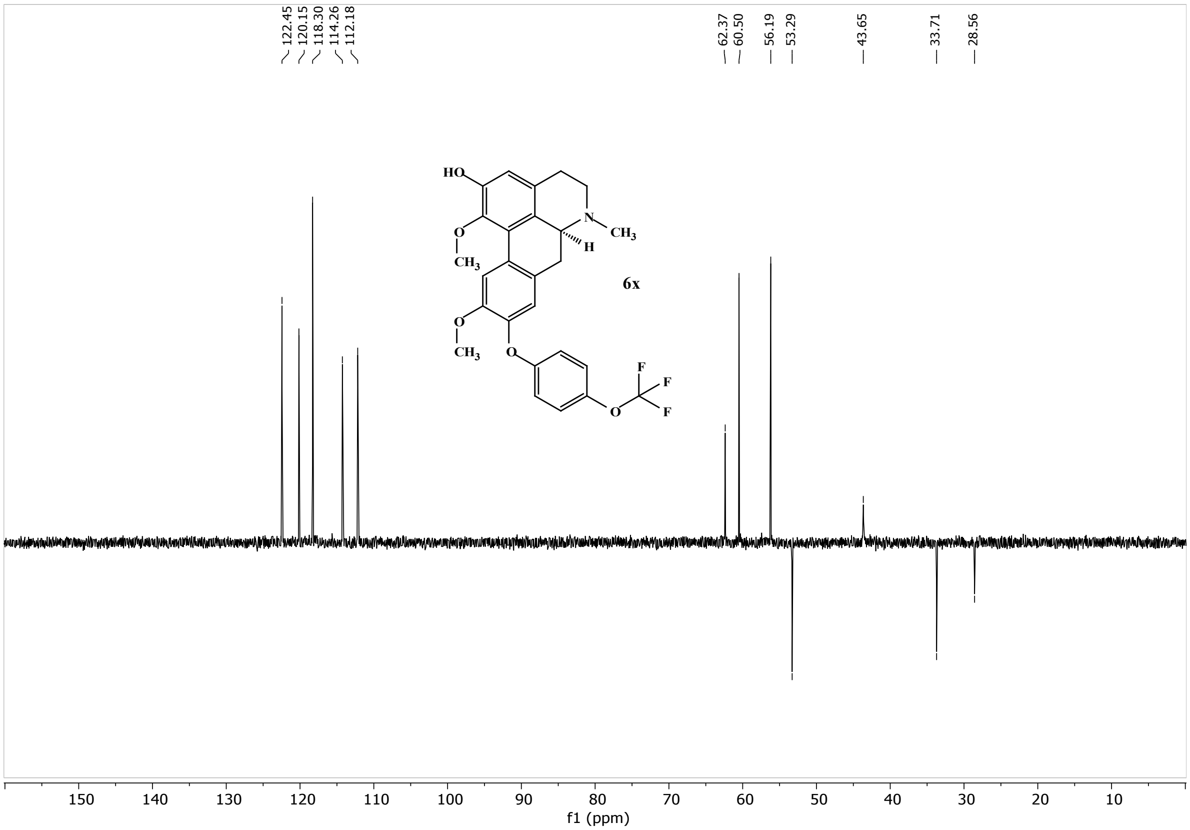

Figure S81. DEPT spectrum of **6x** ($CDCl_3$, 101 MHz).

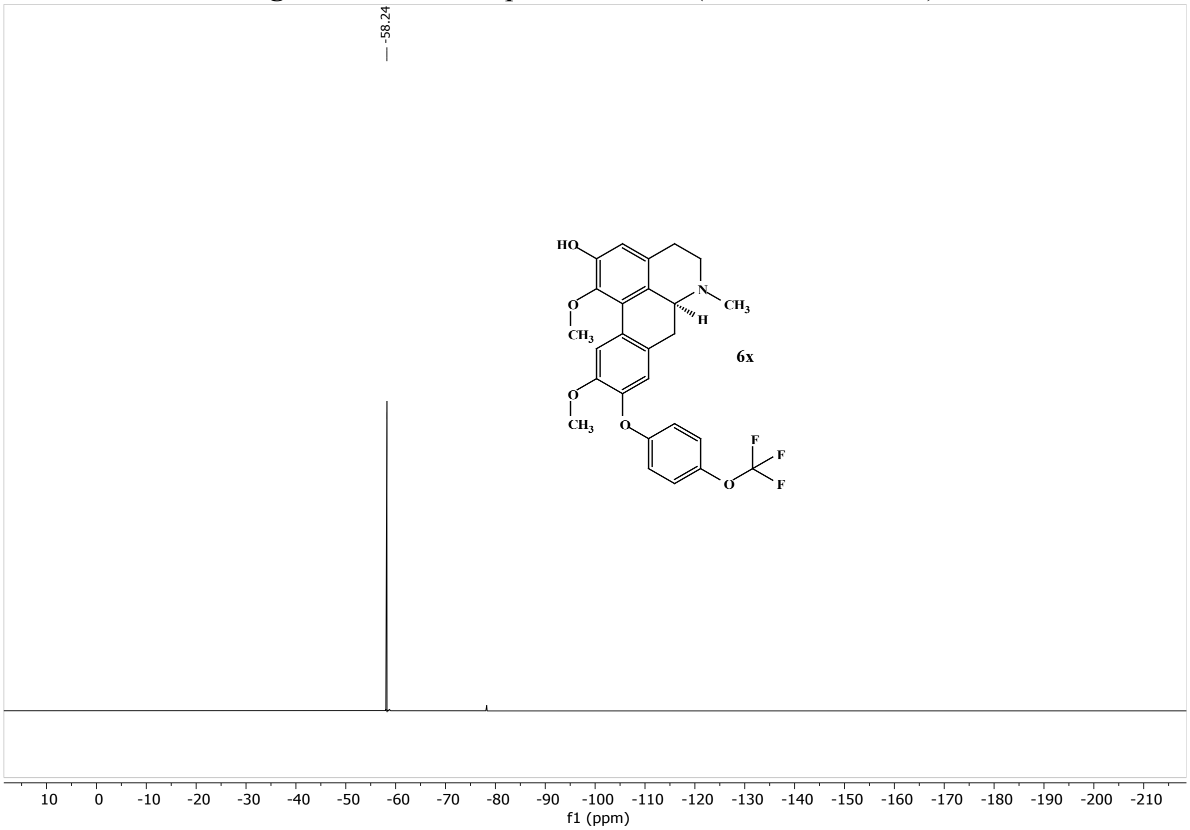

Figure S82. ^{19}F-NMR spectrum of **6x** ($CDCl_3$, 376 MHz).

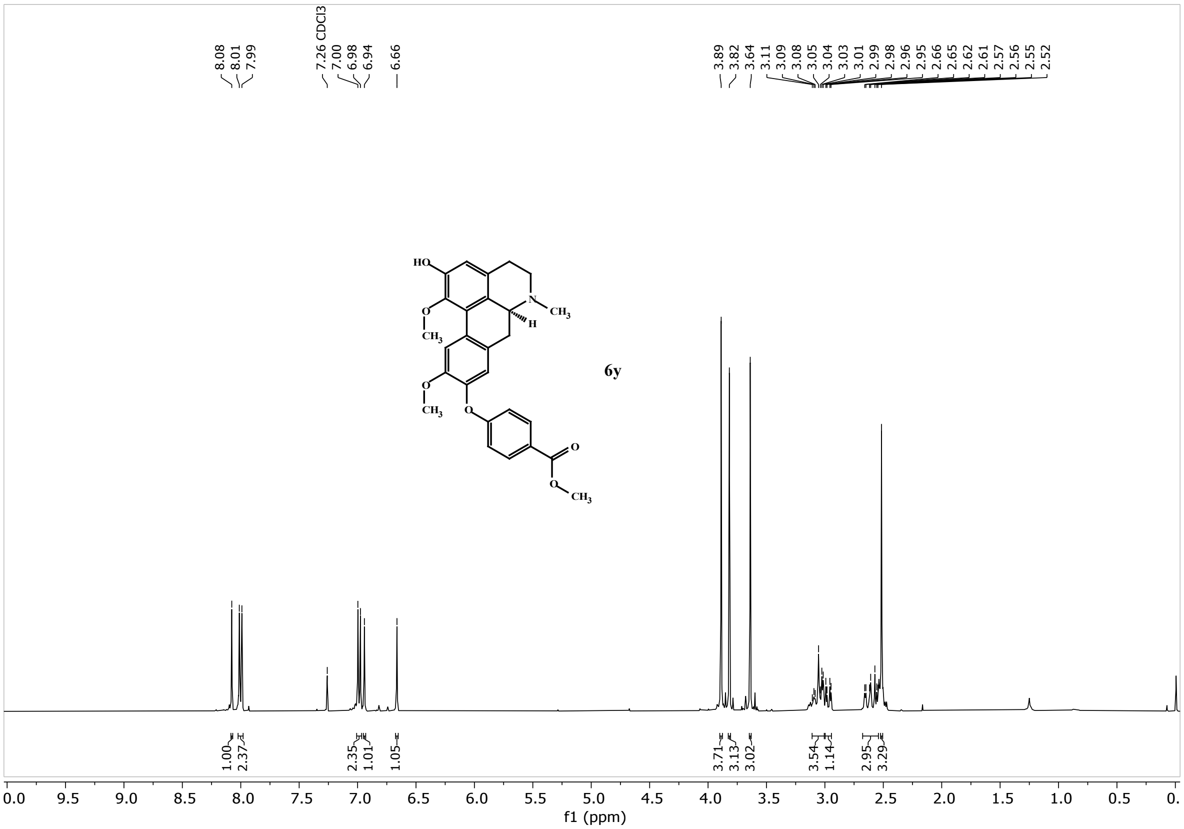

Figure S83. ^{1}H-NMR spectrum of **6y** ($CDCl_3$, 400 MHz).

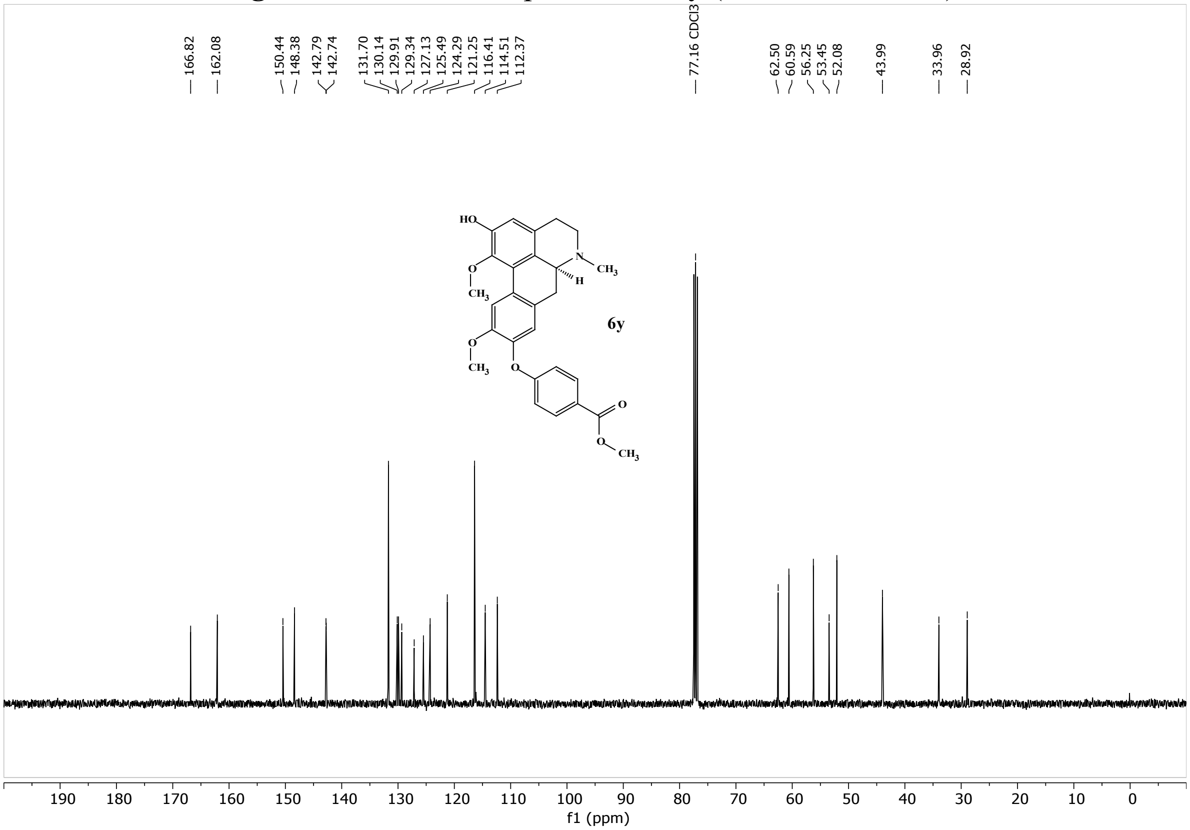

Figure S84. ^{13}C-NMR spectrum of **6y** ($CDCl_3$, 101 MHz).

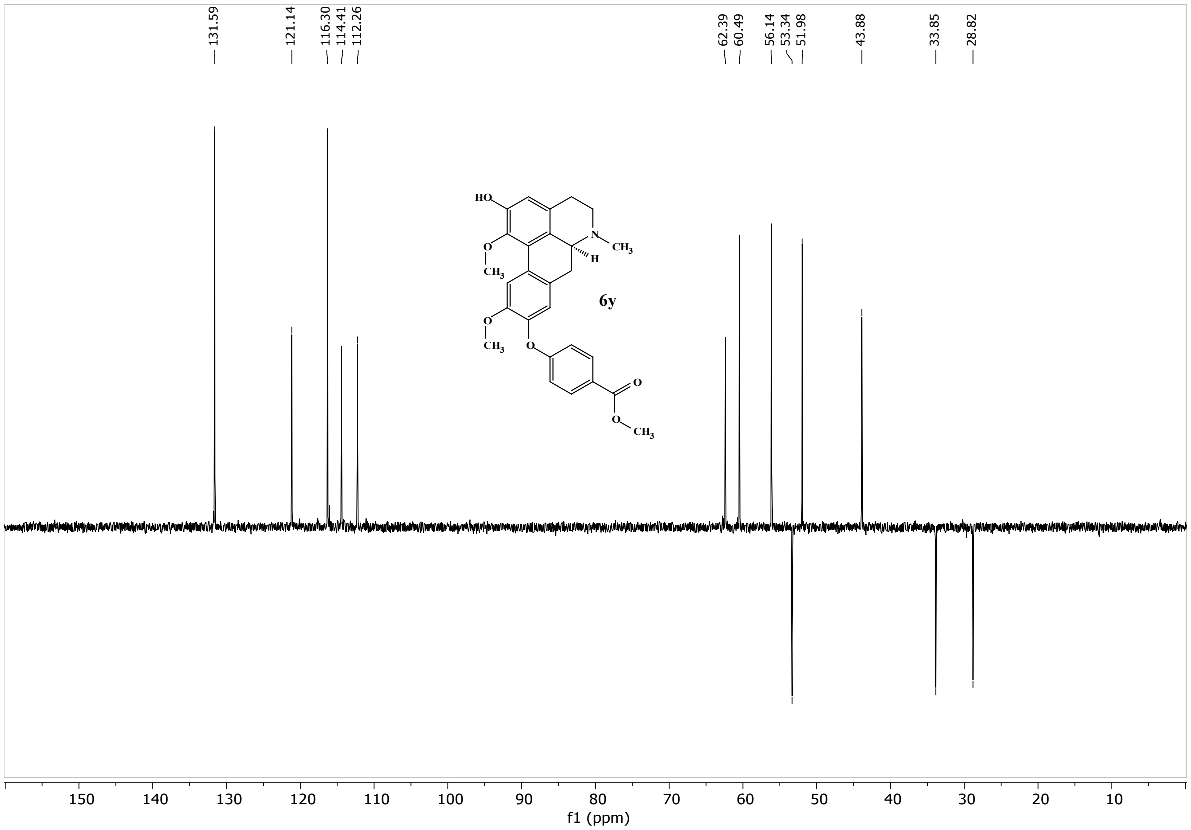

Figure S85. DEPT spectrum of **6y** ($CDCl_3$, 101 MHz).

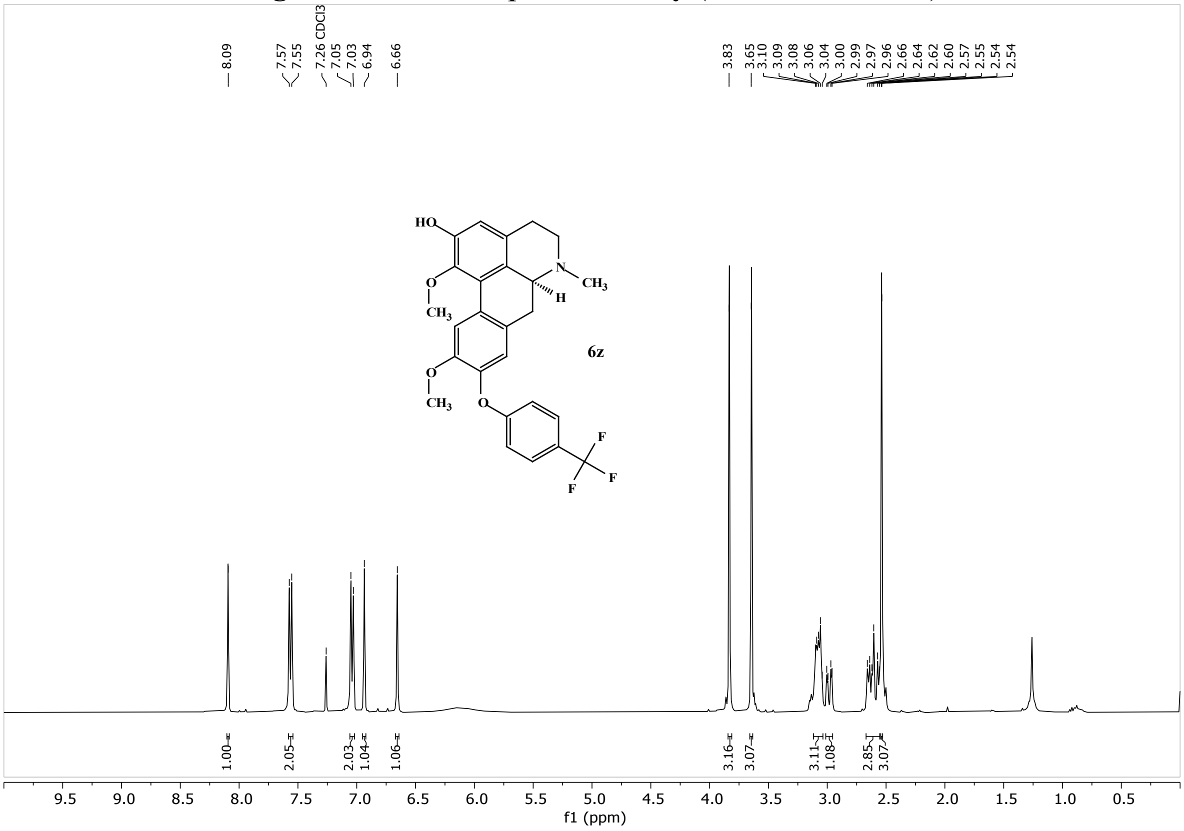

Figure S86. ^{1}H-NMR spectrum of **6z** ($CDCl_3$, 400 MHz).

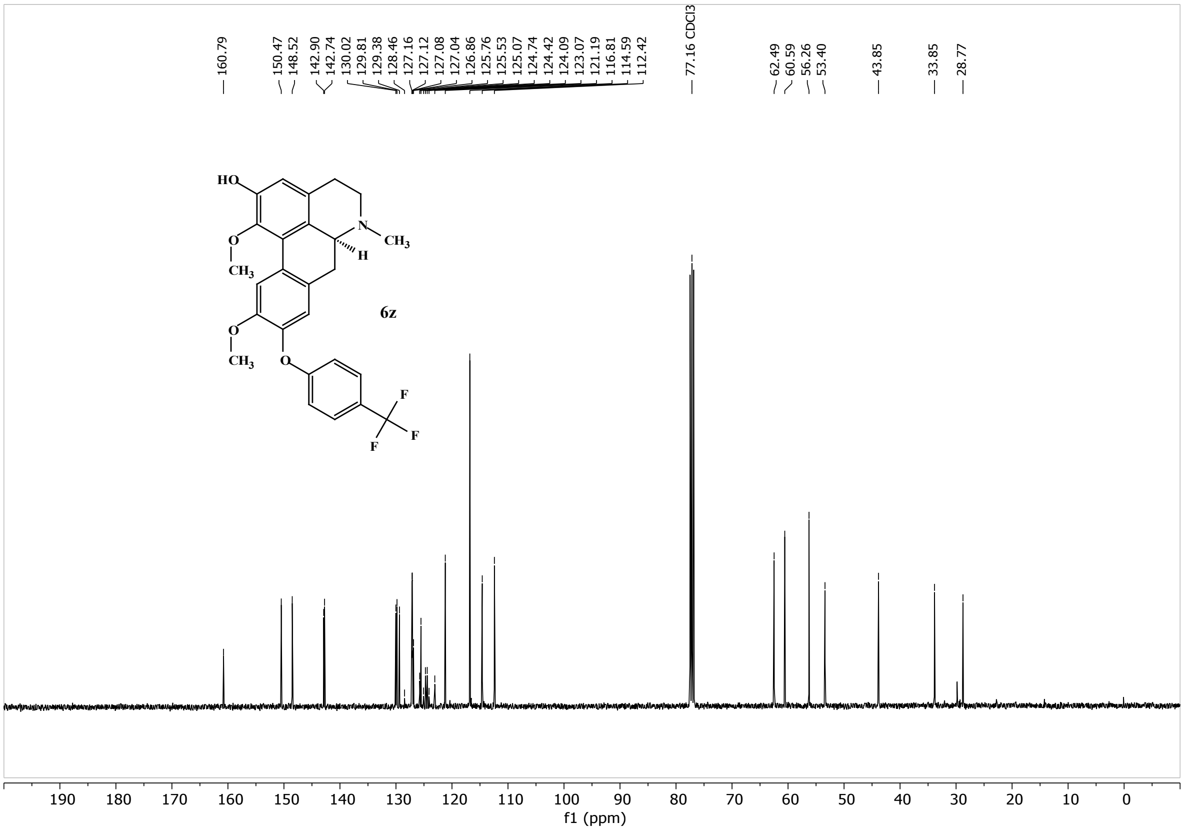

Figure S87. ^{13}C-NMR spectrum of **6z** ($CDCl_3$, 101 MHz).

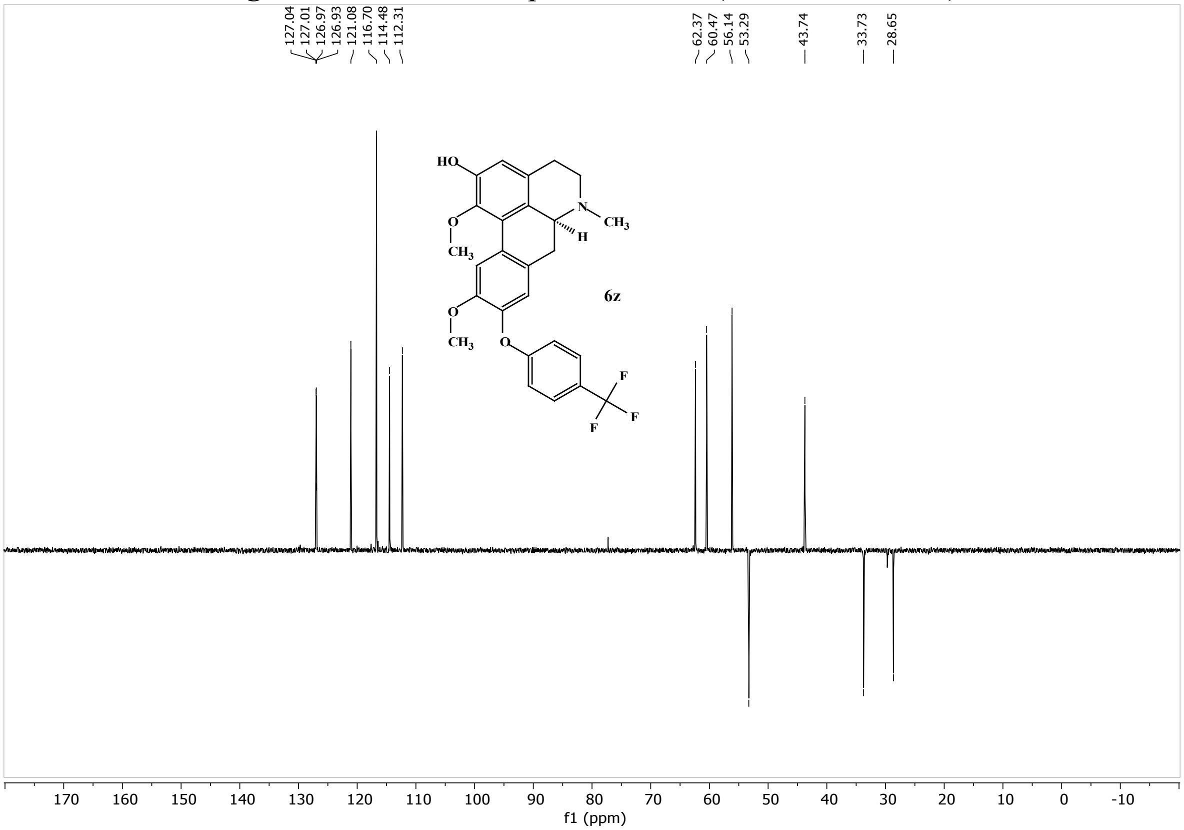

Figure S88. DEPT spectrum of **6z** ($CDCl_3$, 101 MHz).

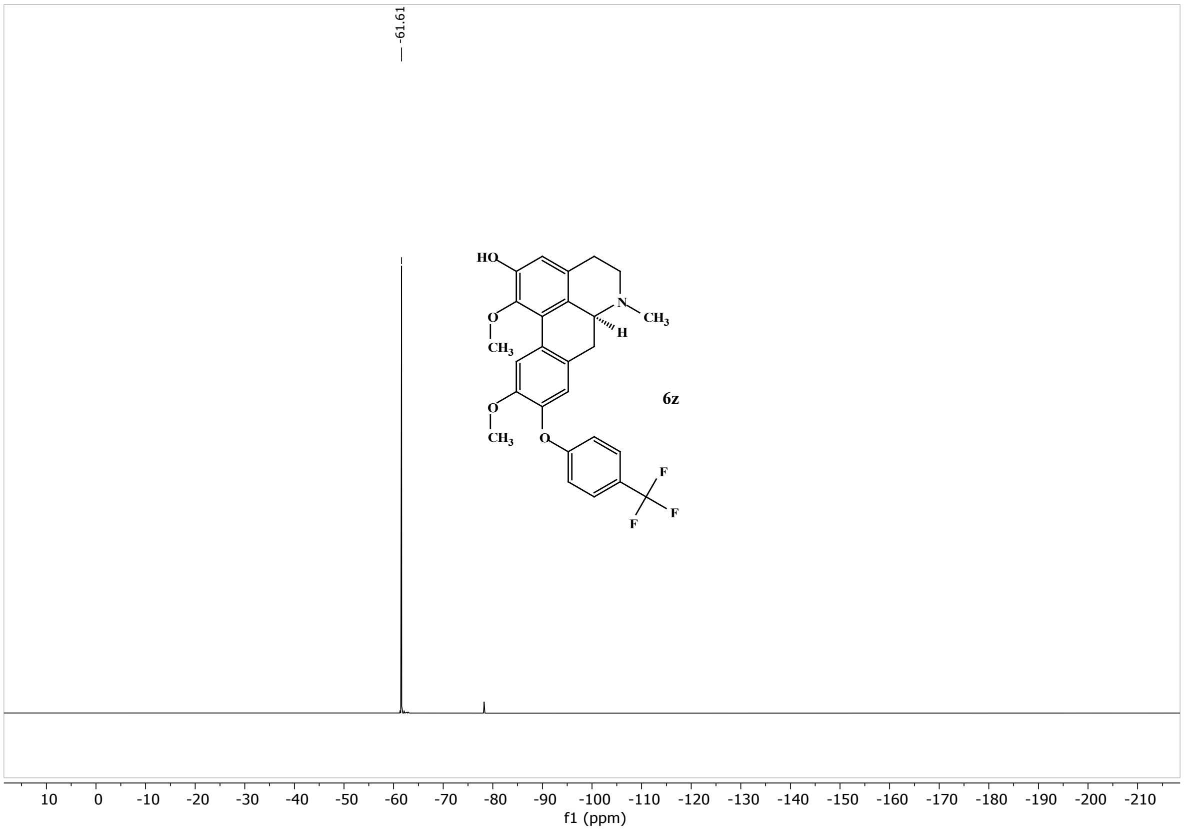

Figure S89. ^{19}F-NMR spectrum of **6z** ($CDCl_3$, 376 MHz).

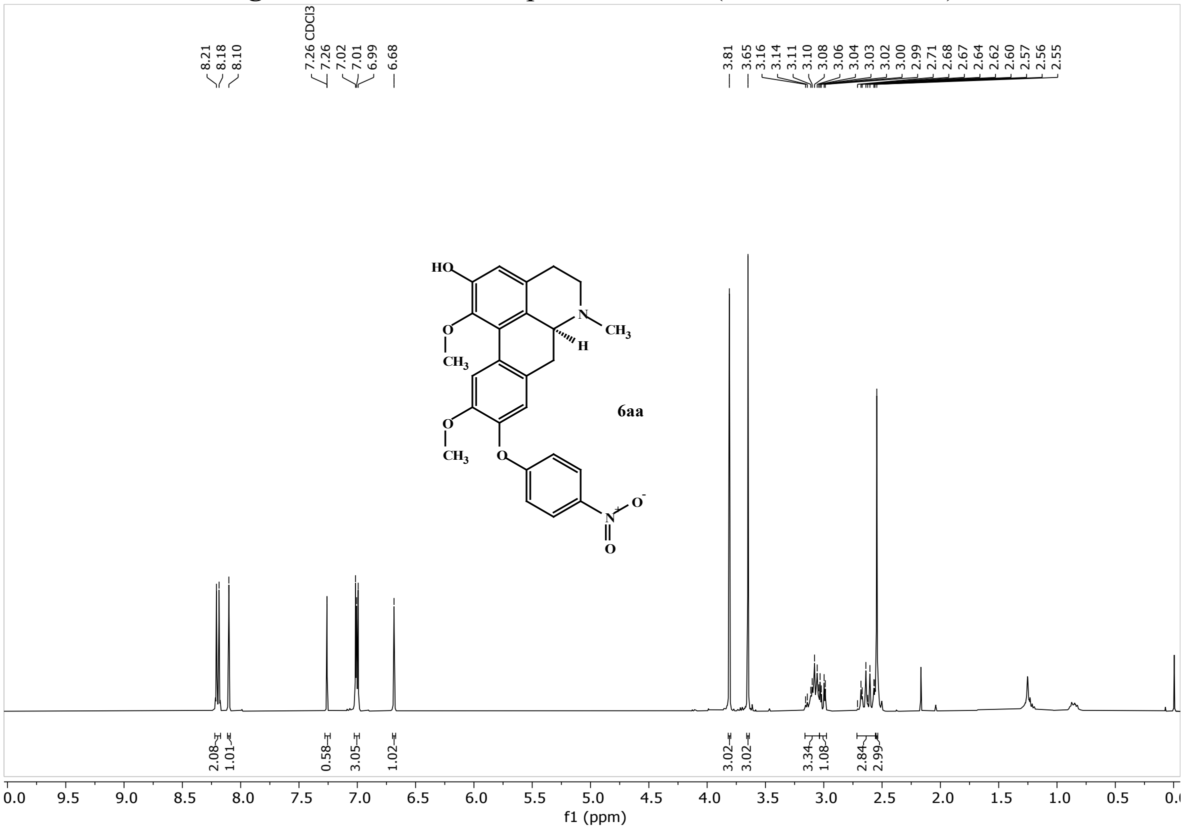

Figure S90. ^{1}H-NMR spectrum of **6aa** ($CDCl_3$, 400 MHz).

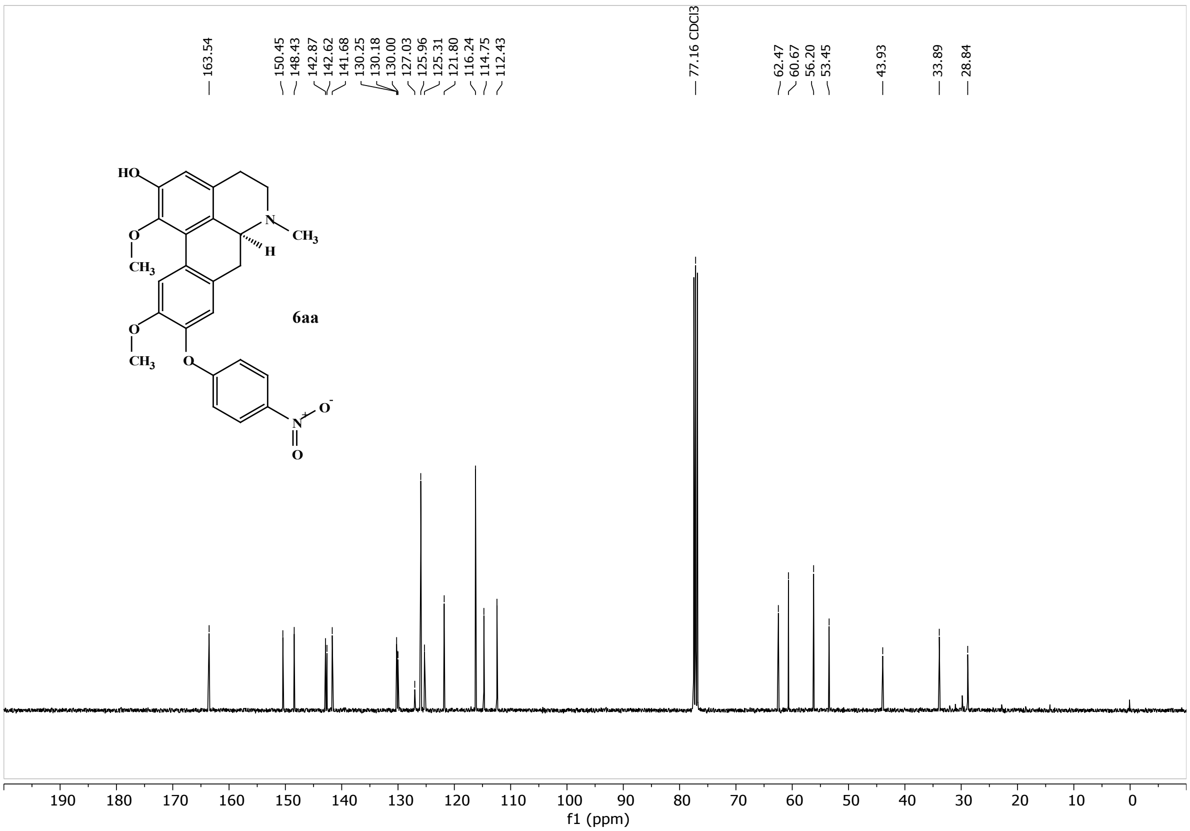

Figure S91. ^{13}C-NMR spectrum of **6aa** ($CDCl_3$, 101 MHz).

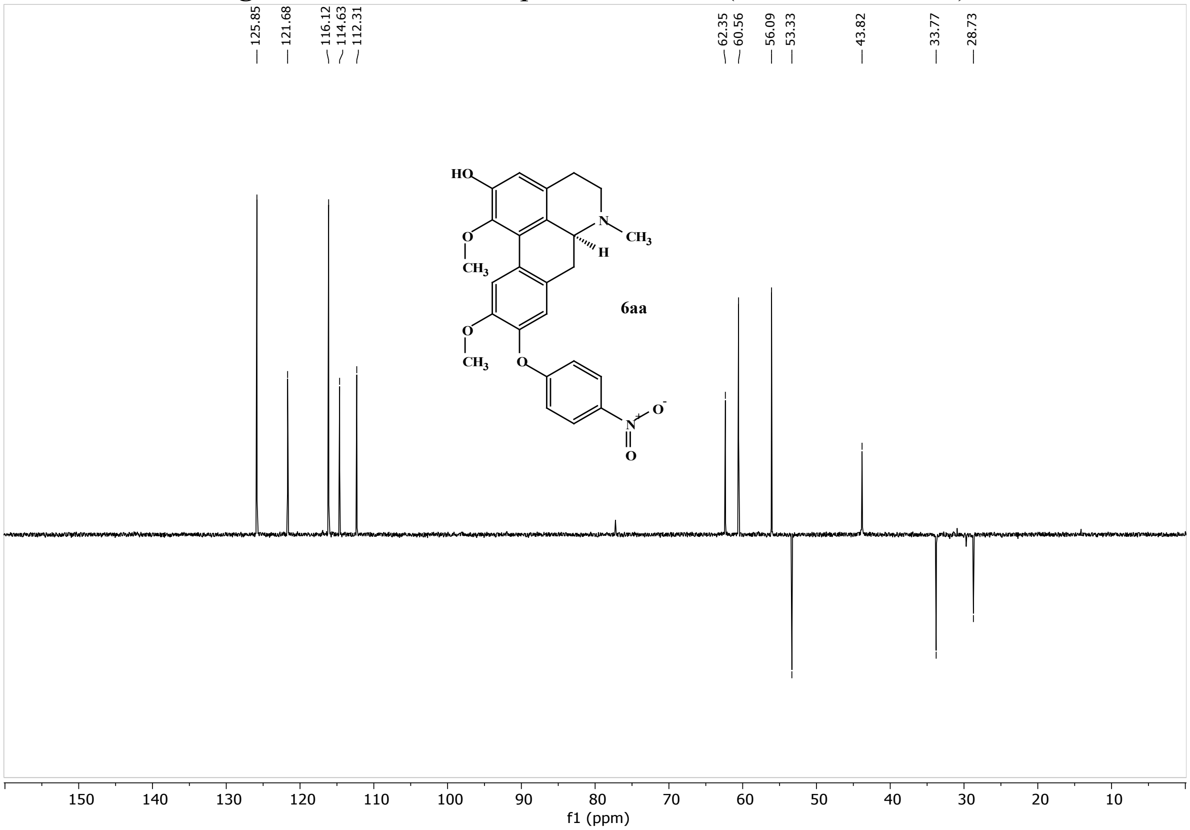

Figure S92. DEPT spectrum of **6aa** ($CDCl_3$, 101 MHz).

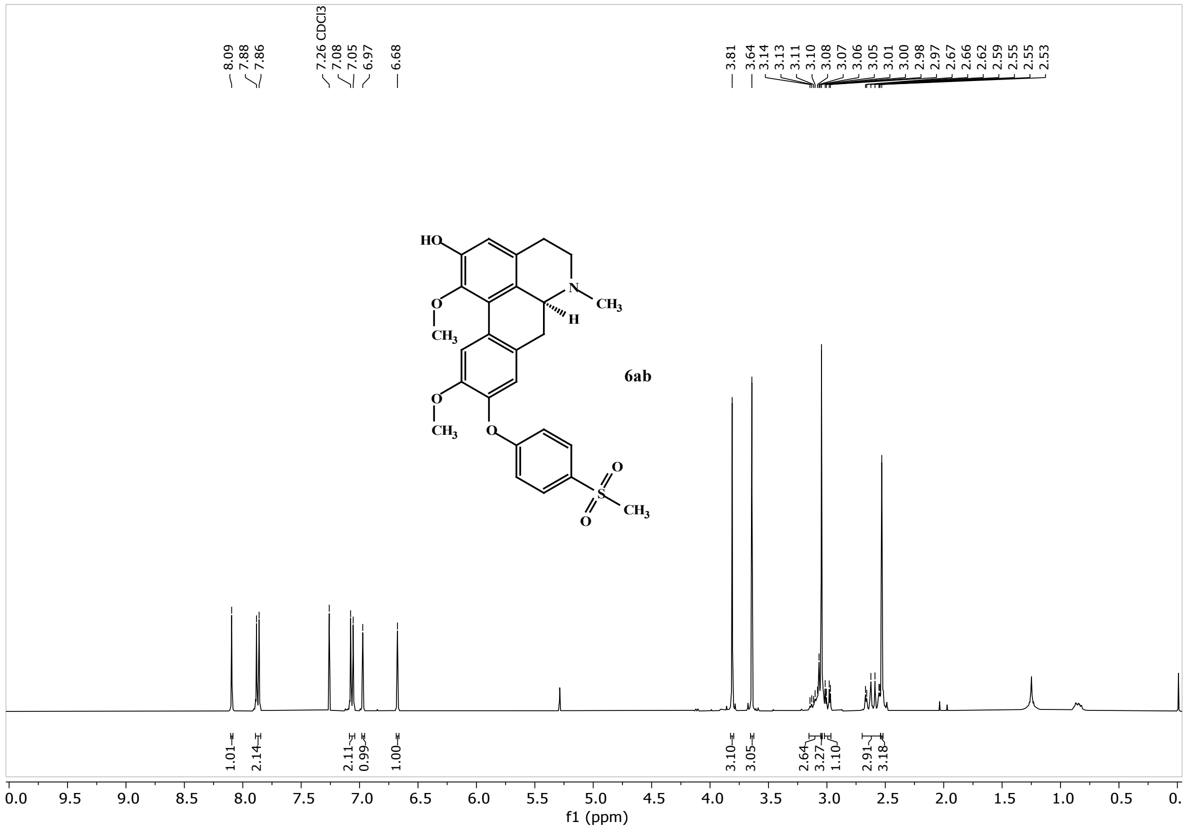

Figure S93. ^{1}H-NMR spectrum of **6ab** ($CDCl_3$, 400 MHz).

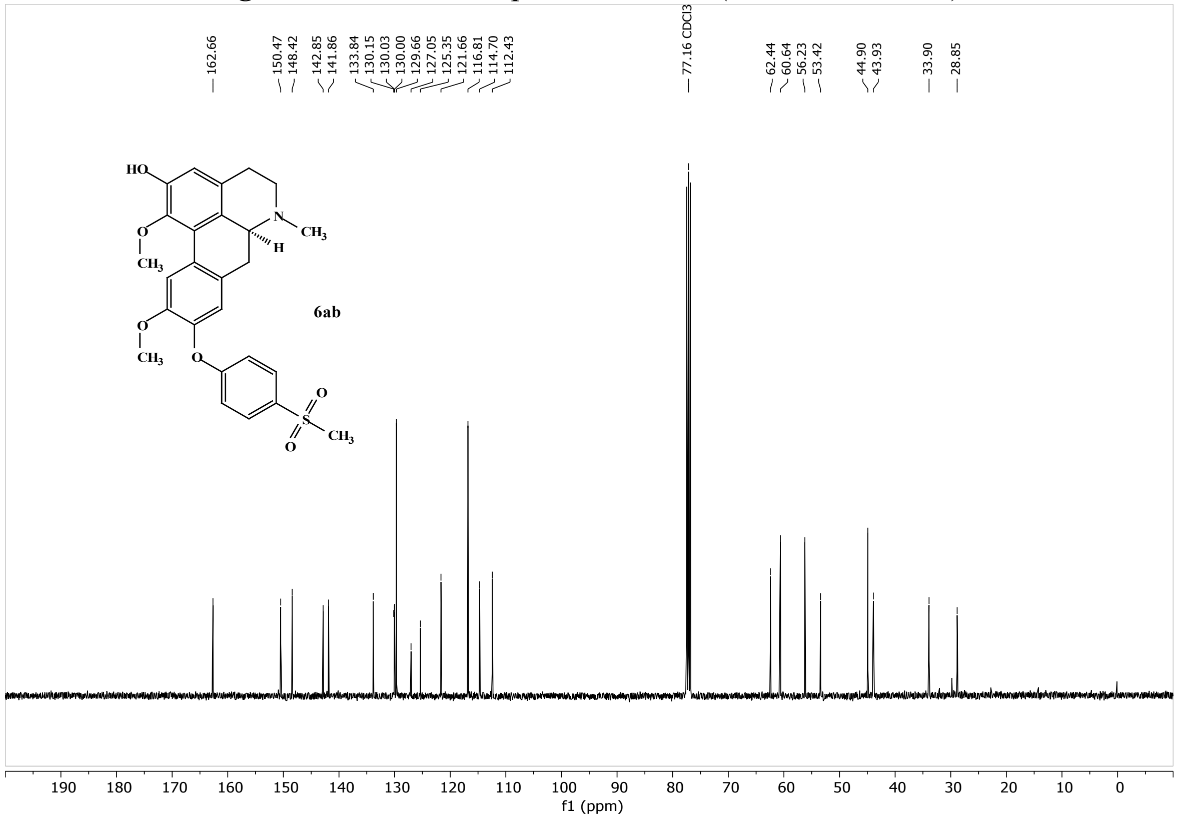

Figure S94. ^{13}C-NMR spectrum of **6ab** ($CDCl_3$, 101 MHz).

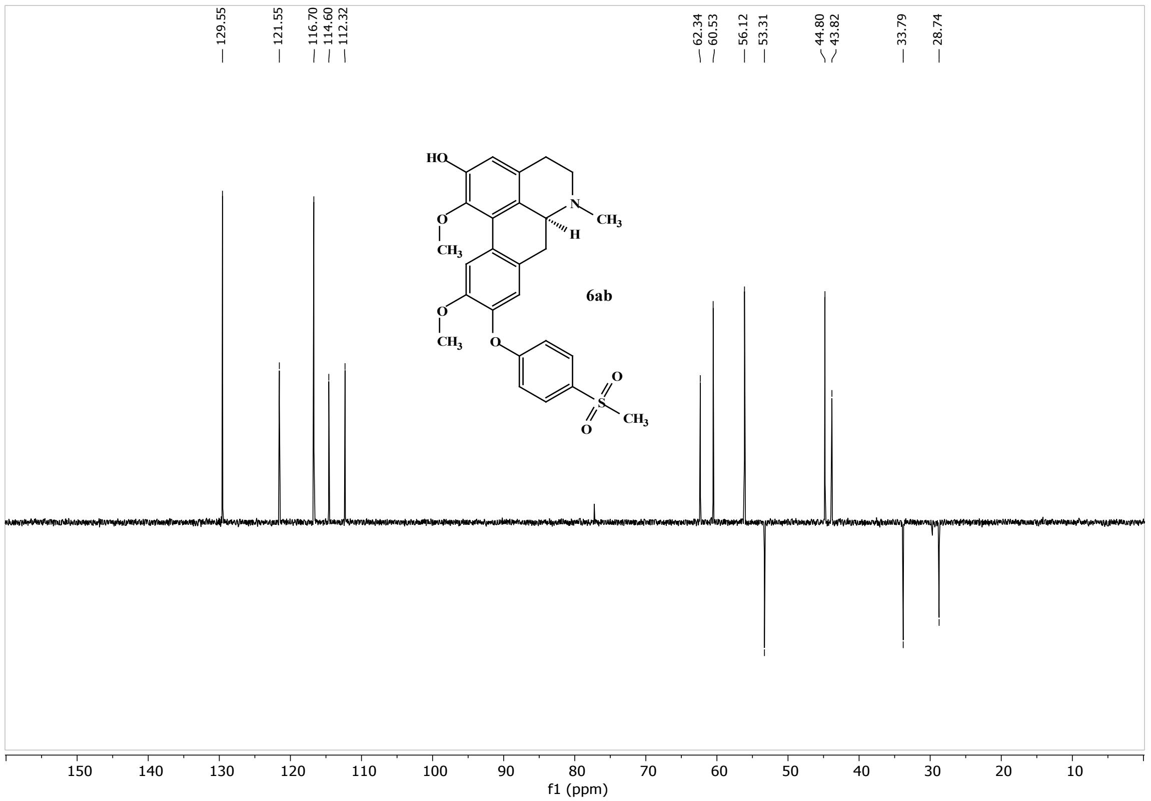

Figure S95. DEPT spectrum of **6ab** ($CDCl_3$, 101 MHz).

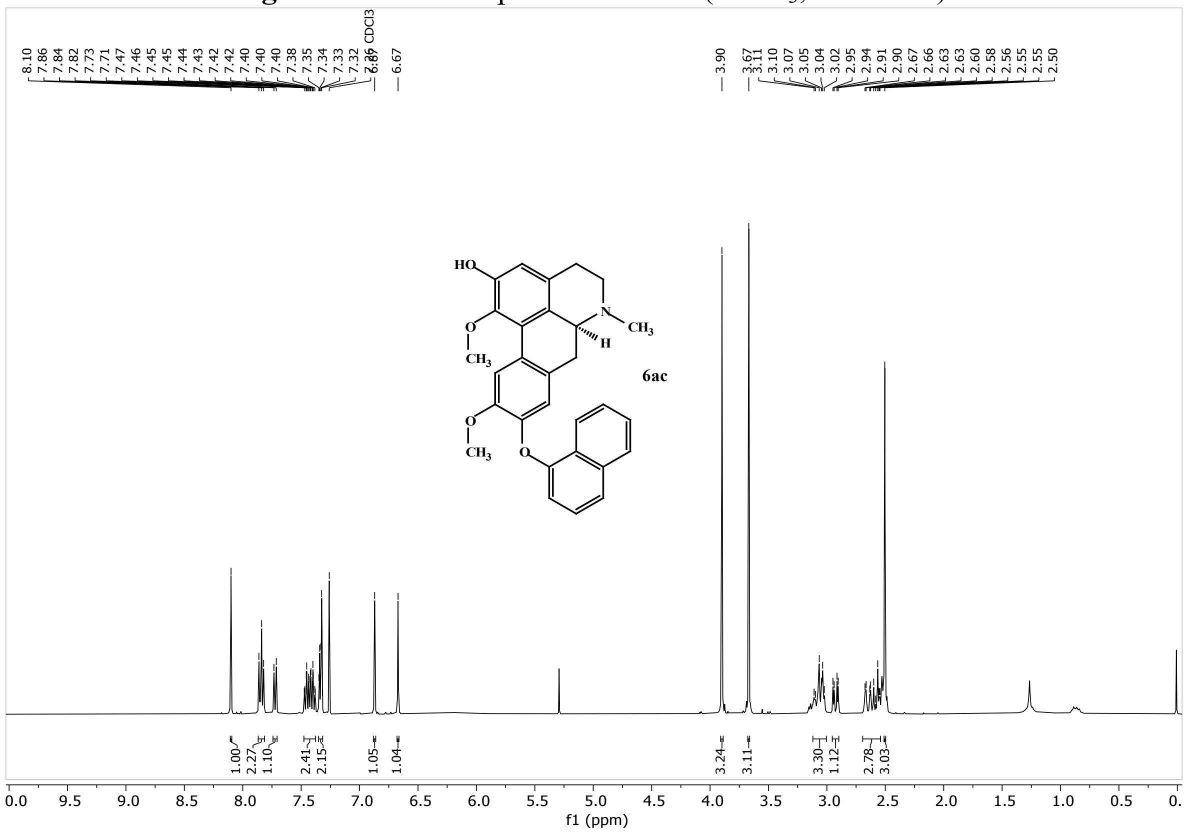

Figure S96. ^{1}H-NMR spectrum of **6ac** ($CDCl_3$, 400 MHz).

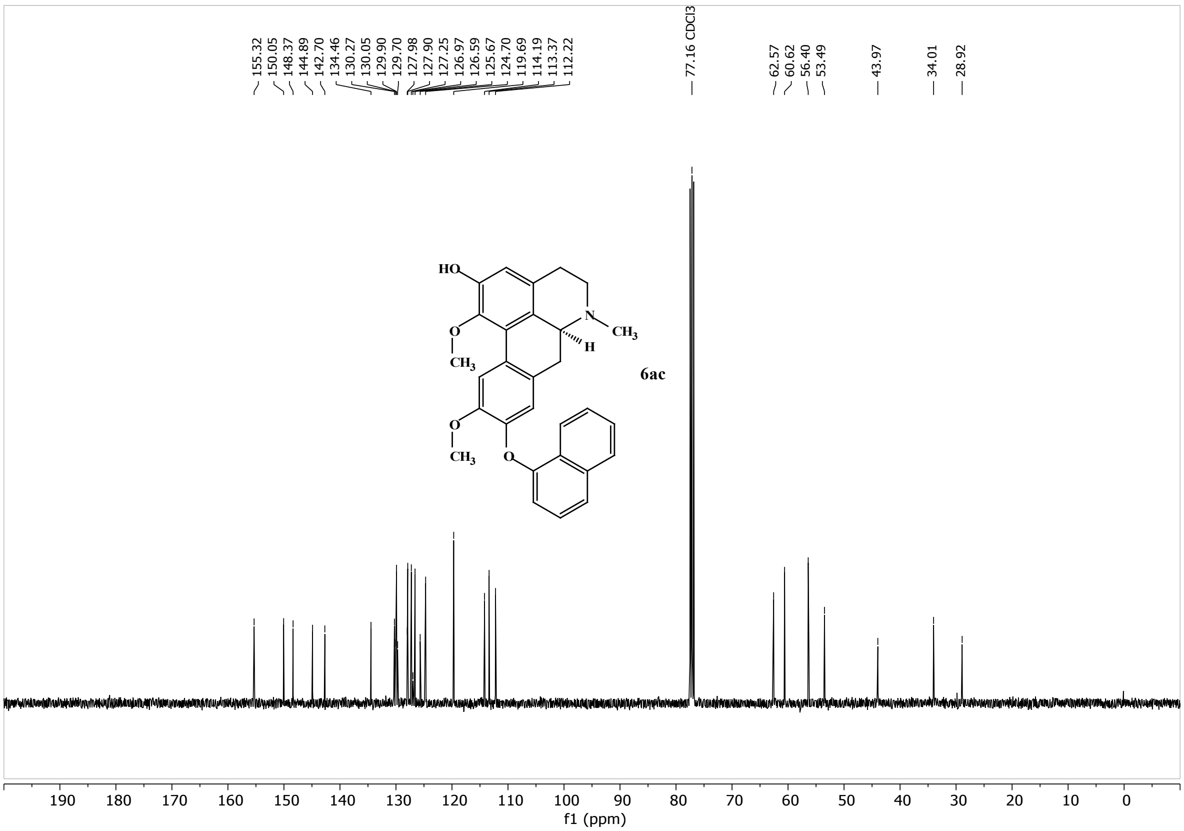

Figure S97. ^{13}C-NMR spectrum of **6ac** ($CDCl_3$, 101 MHz).

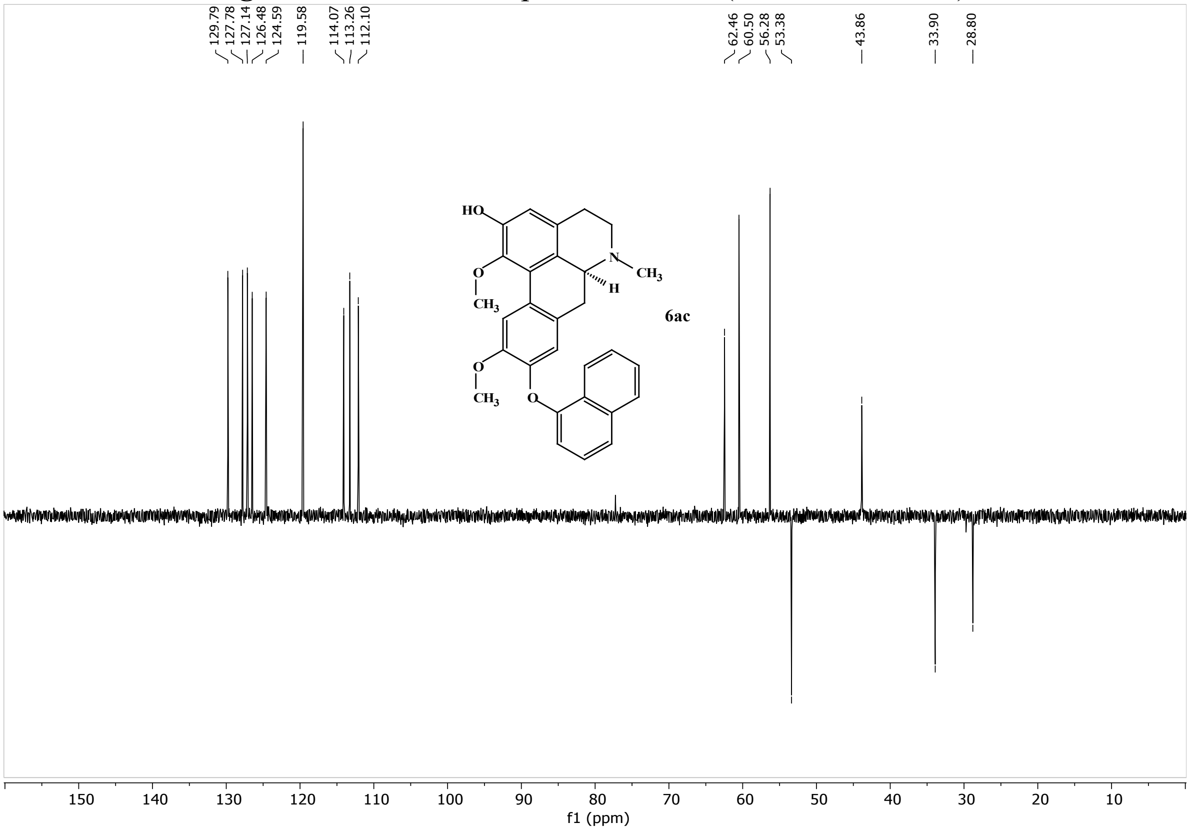

Figure S98. DEPT spectrum of **6ac** ($CDCl_3$, 101 MHz).

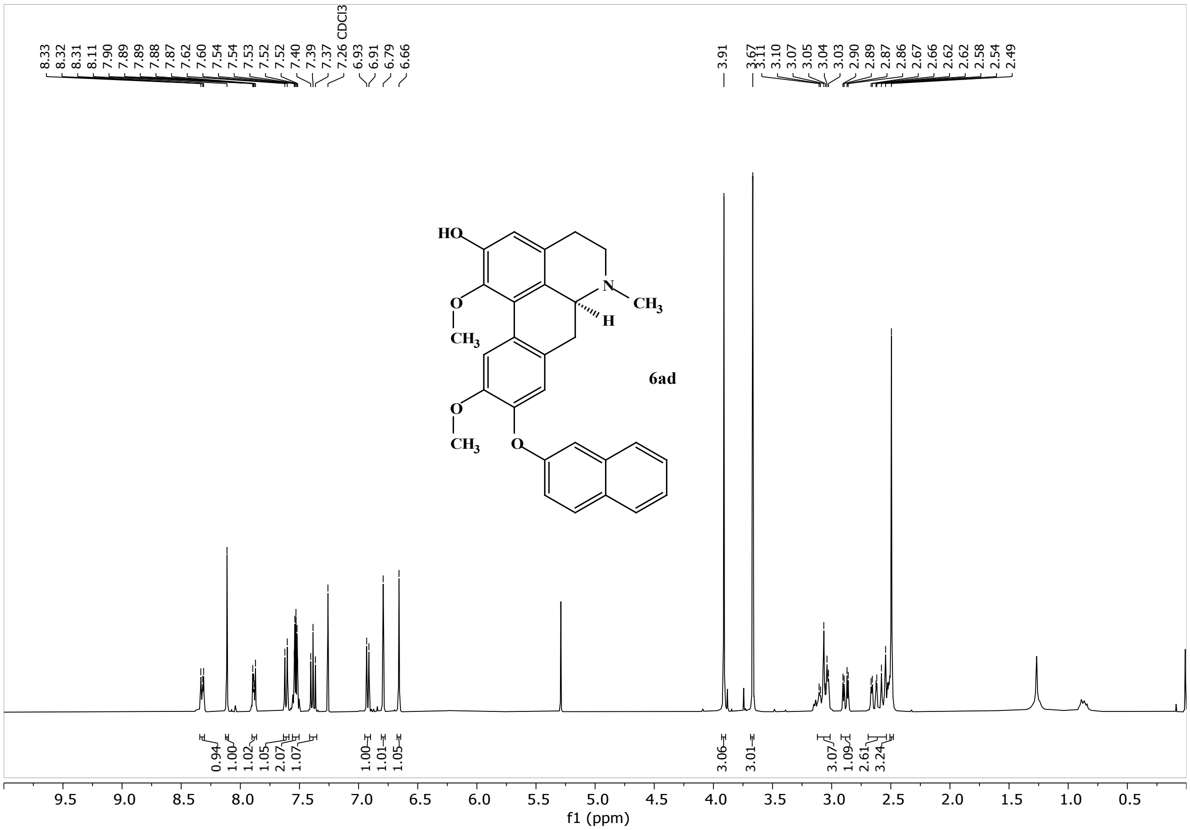

Figure S99. ^{1}H-NMR spectrum of **6ad** ($CDCl_3$, 400 MHz).

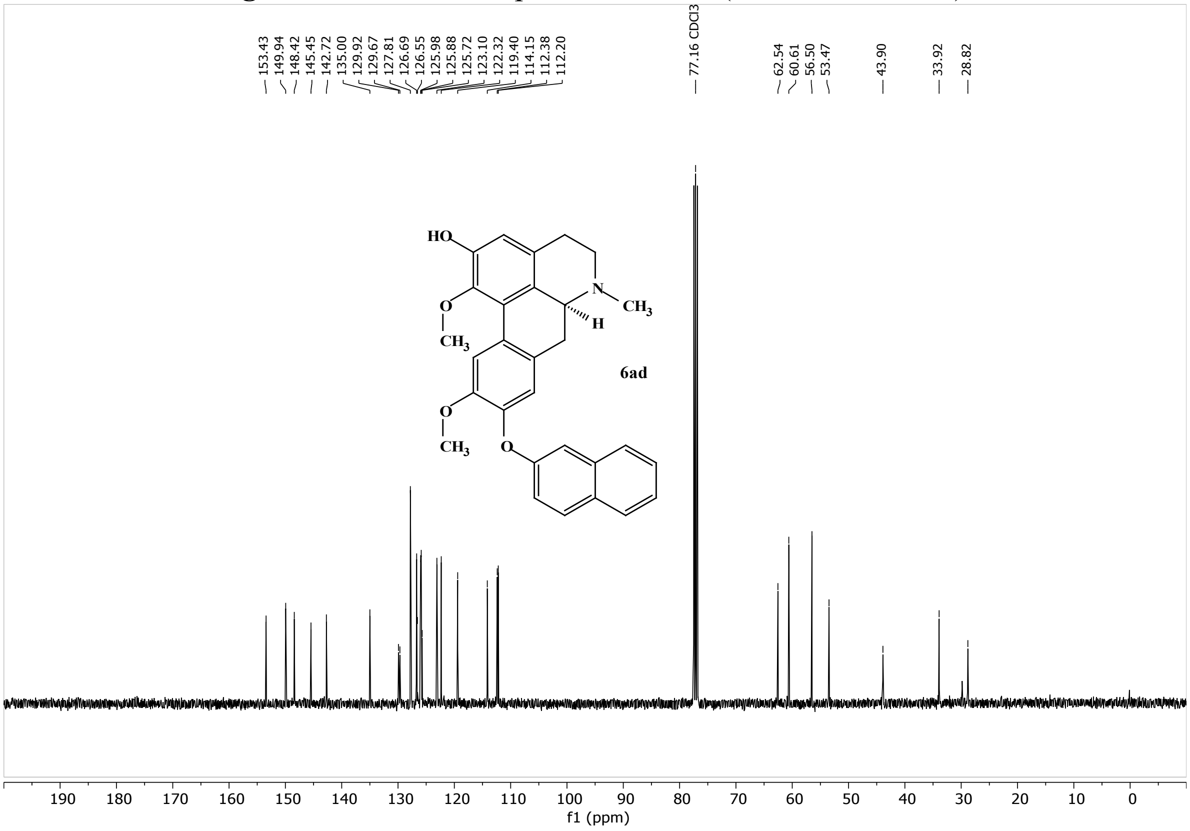

Figure S100. ^{13}C-NMR spectrum of **6ad** ($CDCl_3$, 101 MHz).

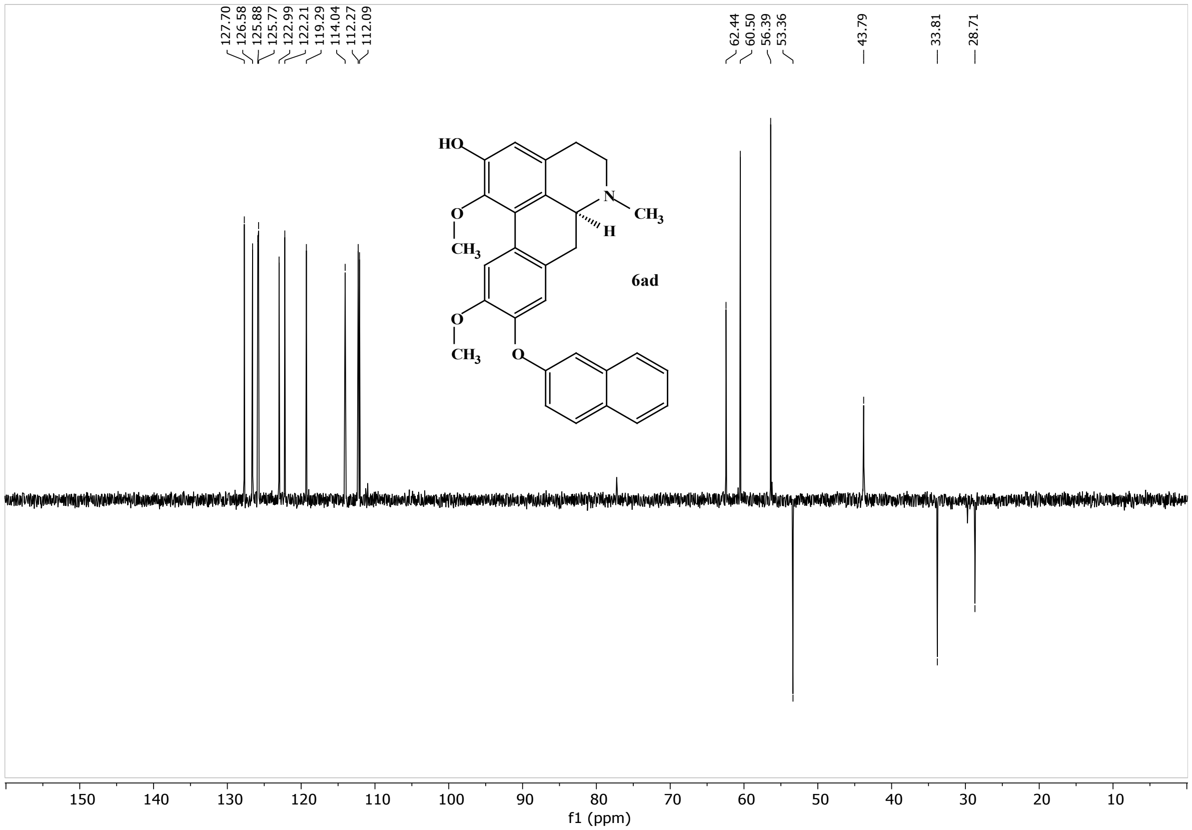

Figure S101. DEPT spectrum of **6ad** ($CDCl_3$, 101 MHz).

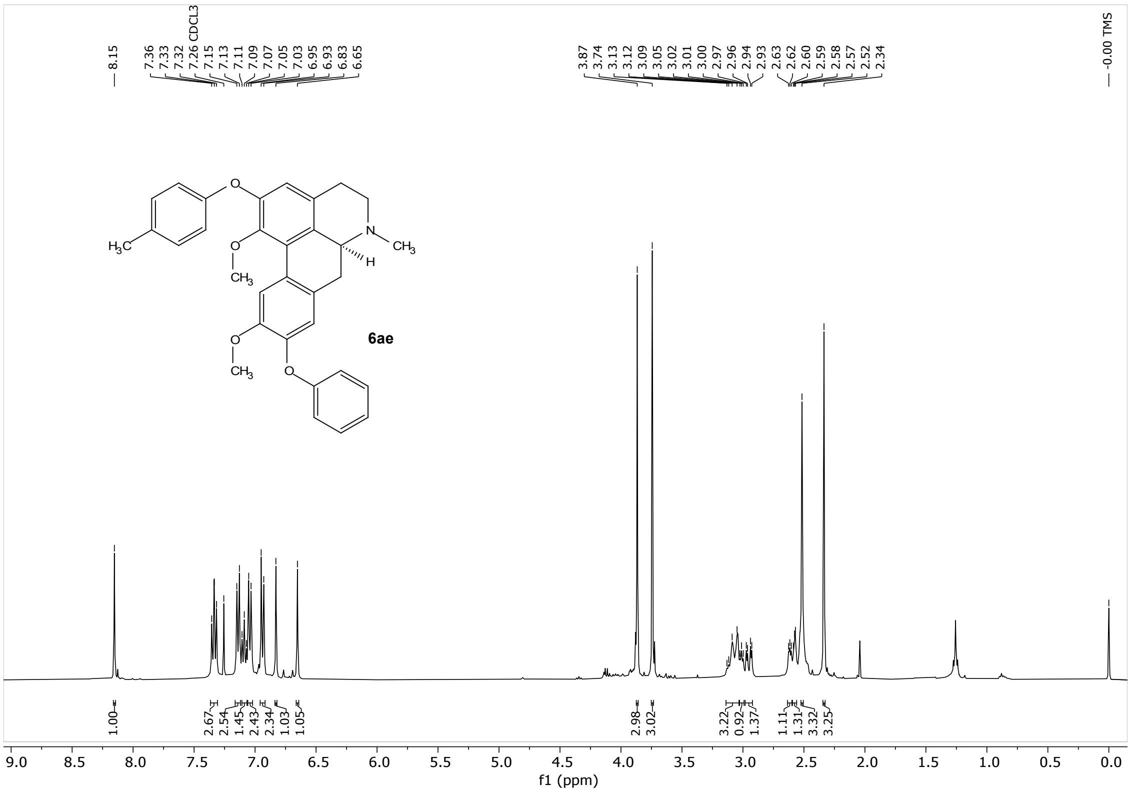

Figure S102. ^{1}H-NMR spectrum of **6ae** ($CDCl_3$, 400 MHz).

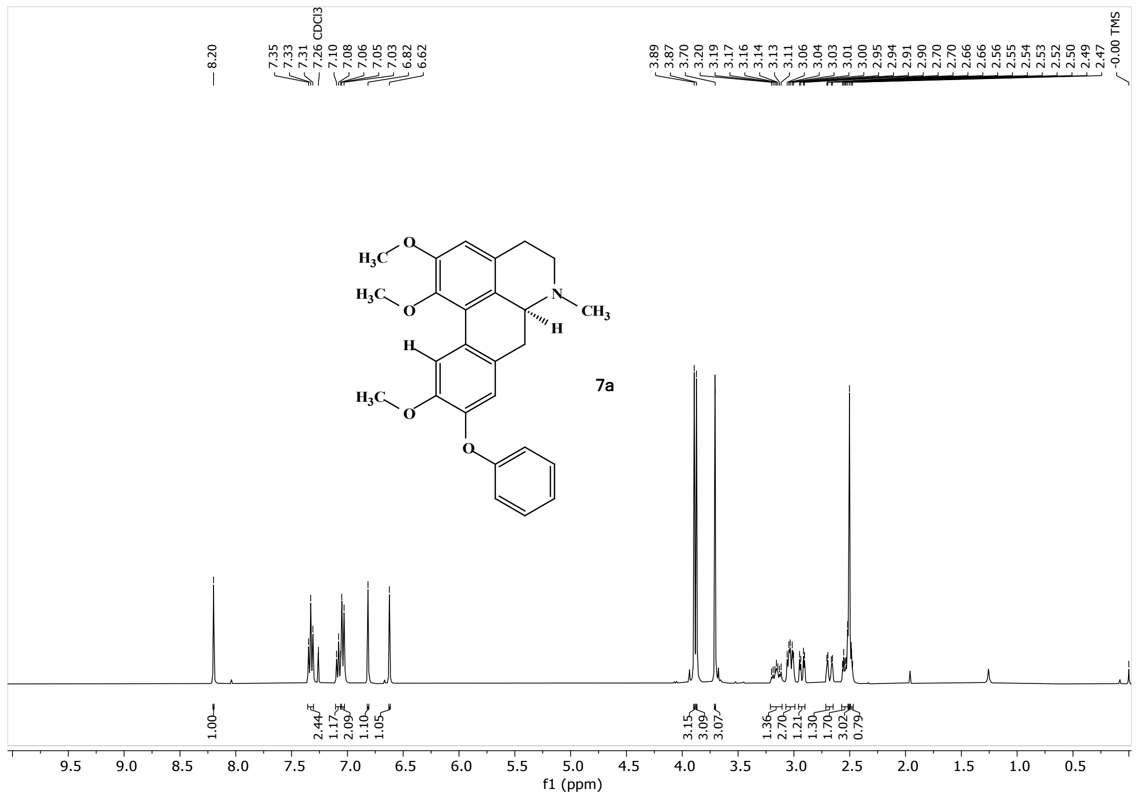

Figure S103. ^{1}H-NMR spectrum of **7a** ($CDCl_3$, 400 MHz).

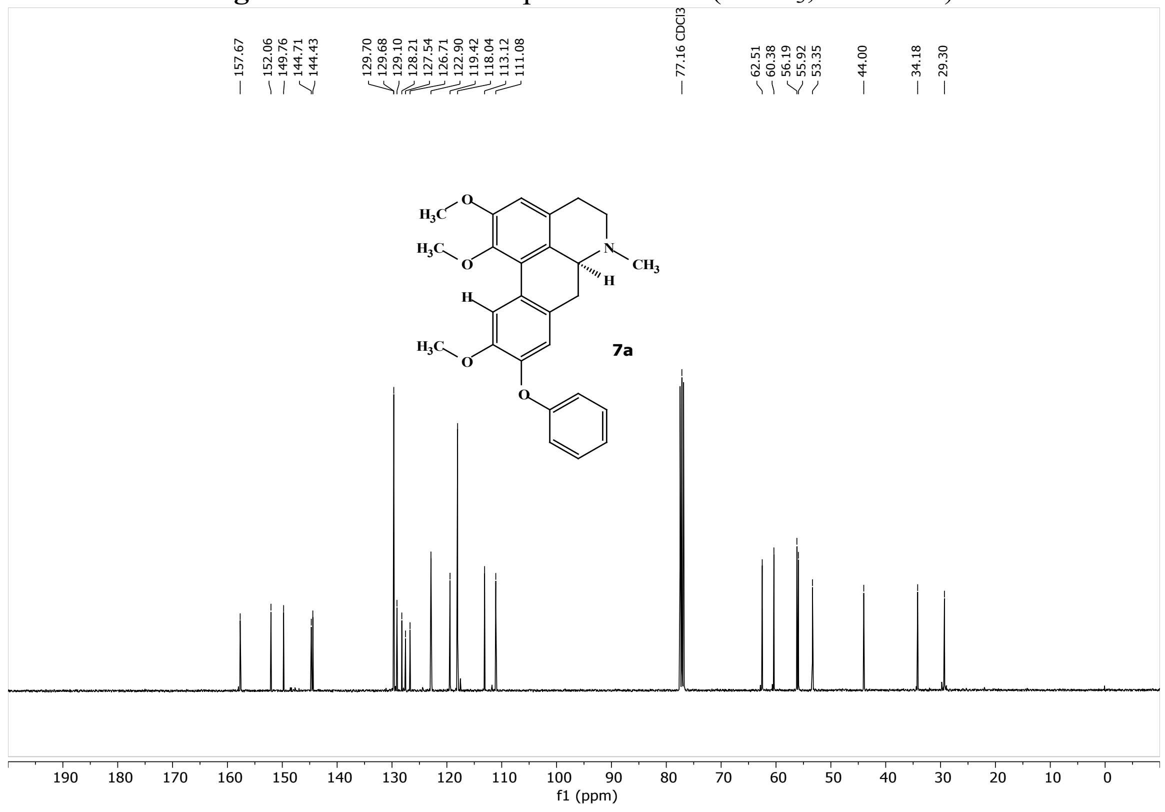

Figure S104. ^{13}C-NMR spectrum of **7a** ($CDCl_3$, 101 MHz).

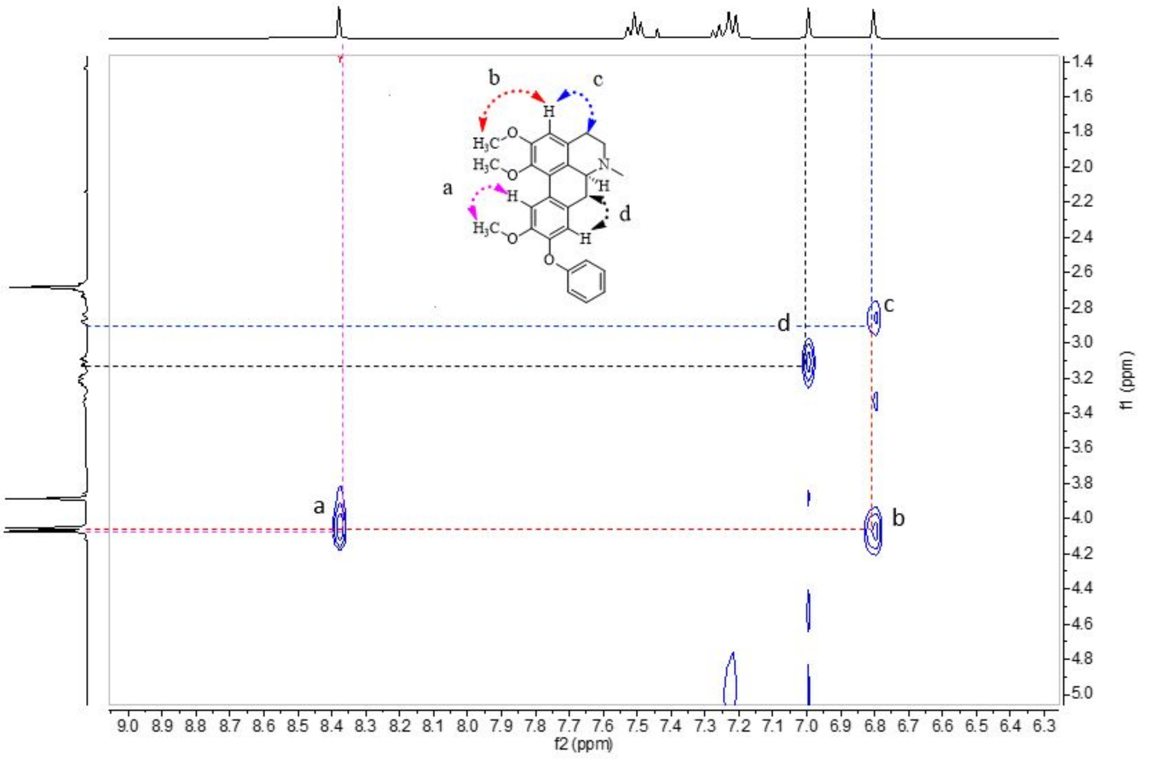

Figure S105. NOESY spectrum of **7a** ($CDCl_3$, 101 MHz).

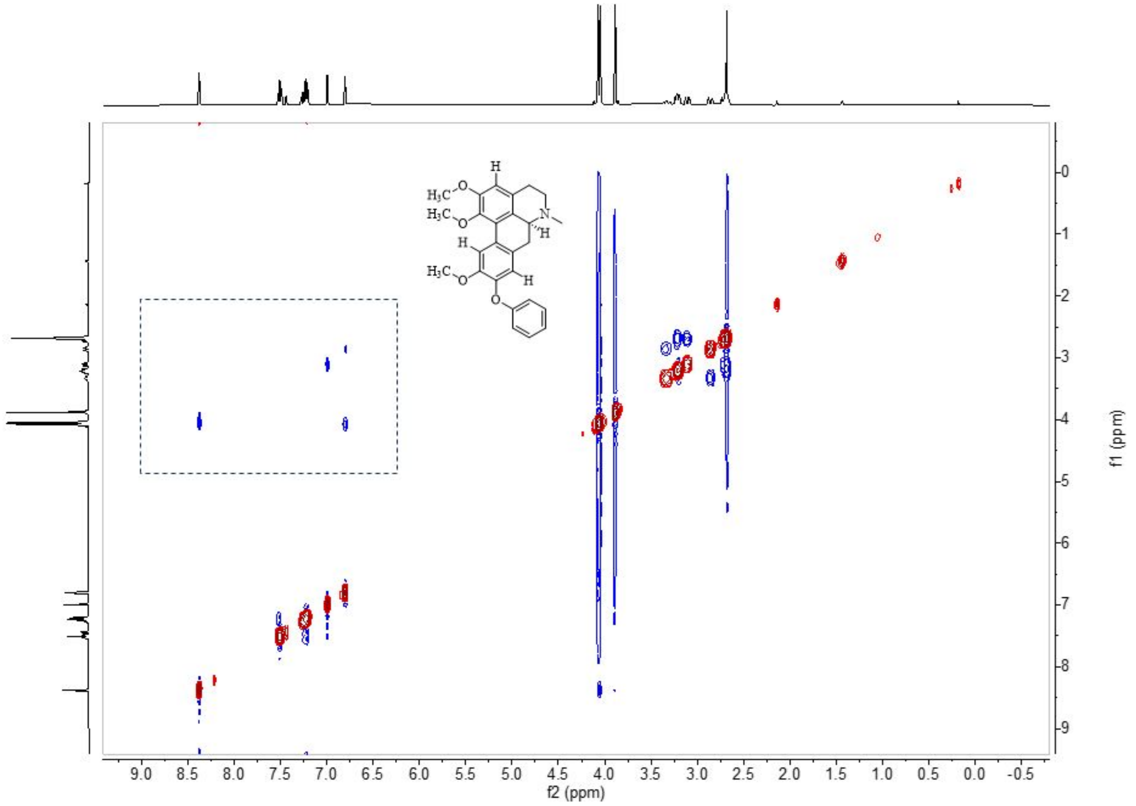

Figure S106. NOESY spectrum of **7a** ($CDCl_3$, 101 MHz).

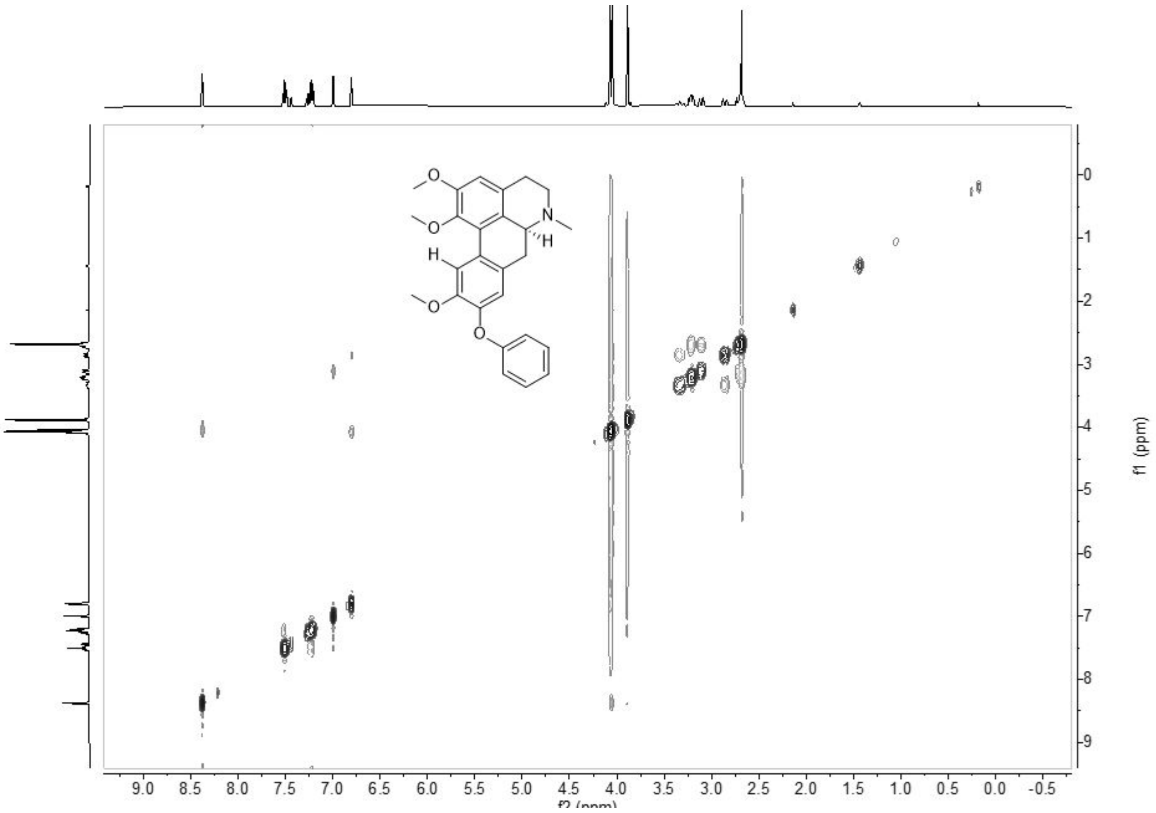

Figure S107. NOESY spectrum of **7a** ($CDCl_3$, 101 MHz).

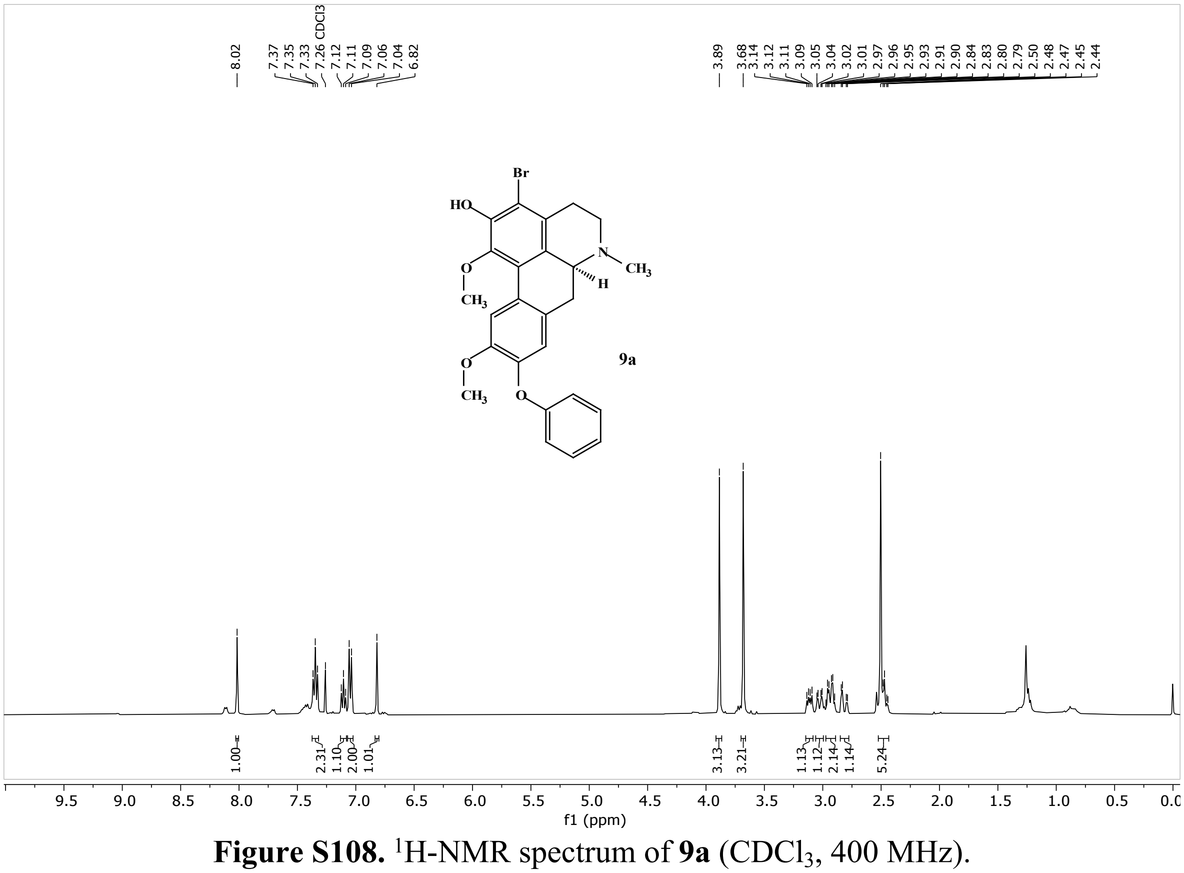

Figure S108. ^{1}H-NMR spectrum of **9a** ($CDCl_3$, 400 MHz).

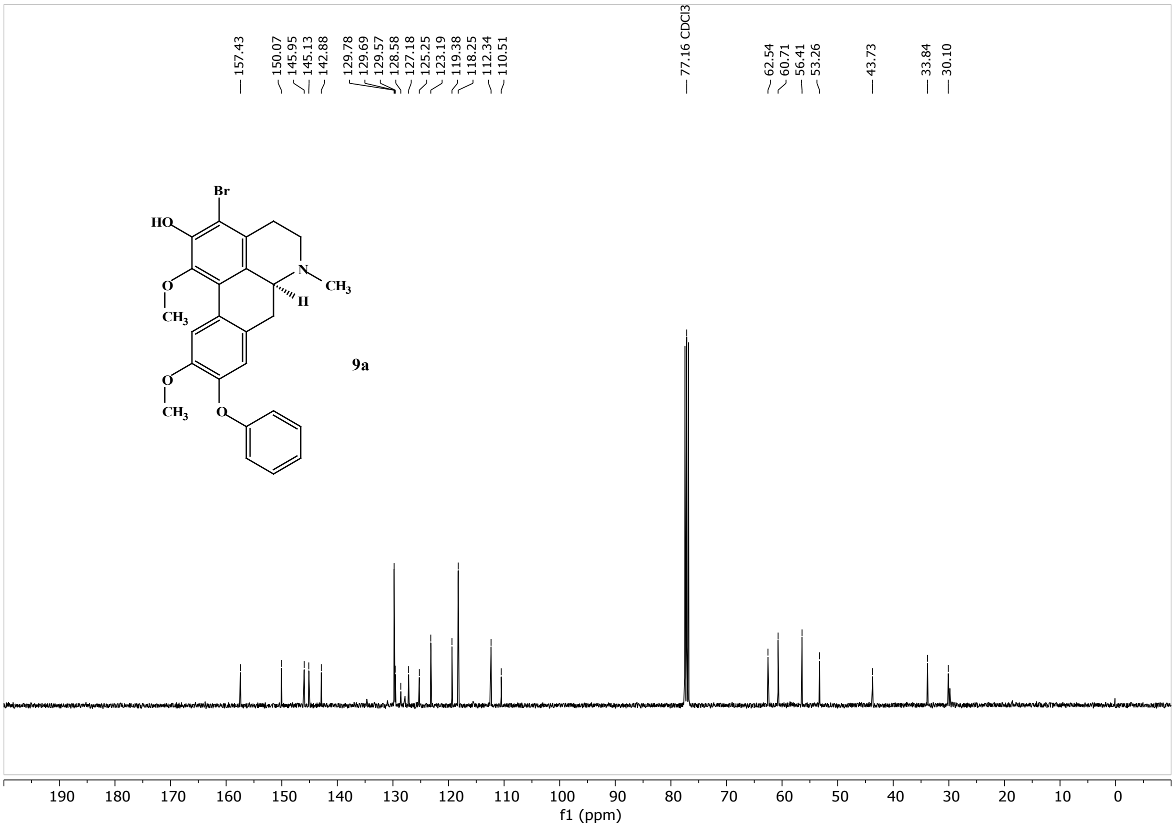

Figure S109. ^{13}C-NMR spectrum of **9a** ($CDCl_3$, 101 MHz).

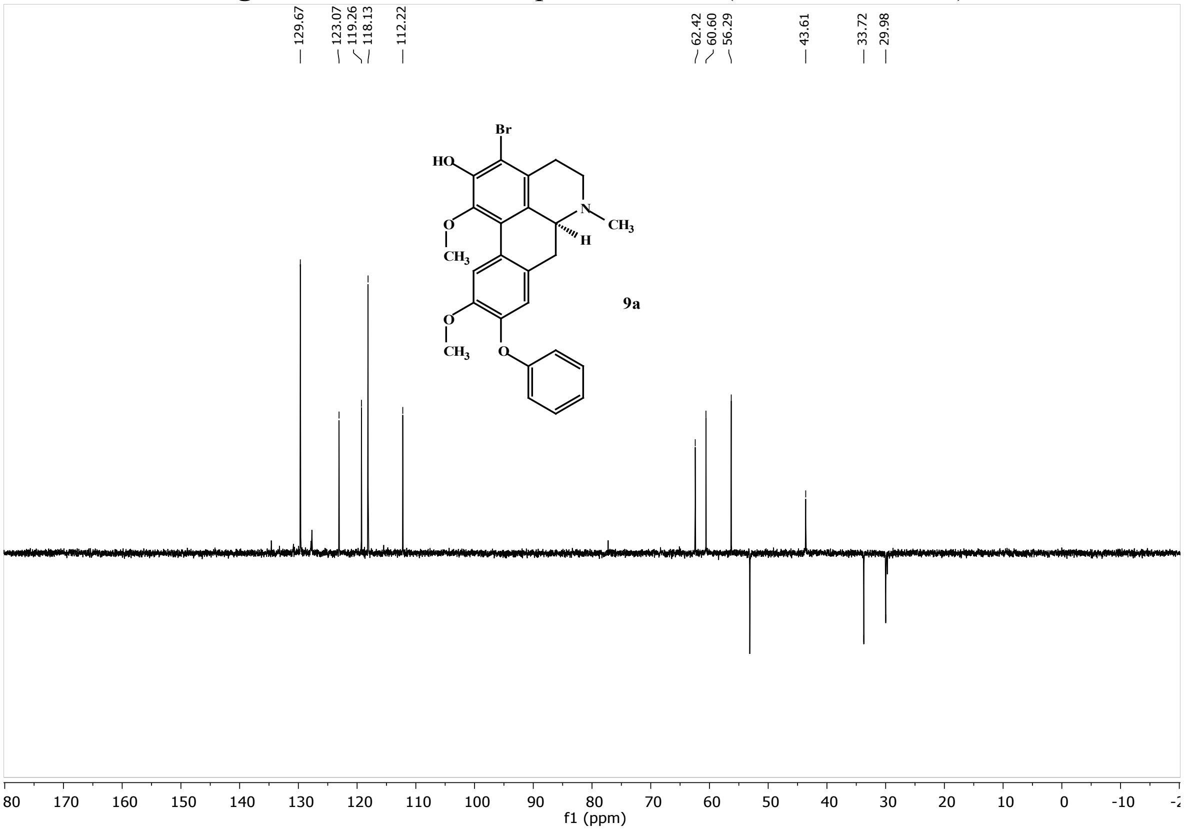

Figure S110. DEPT spectrum of **9a** ($CDCl_3$, 101 MHz).

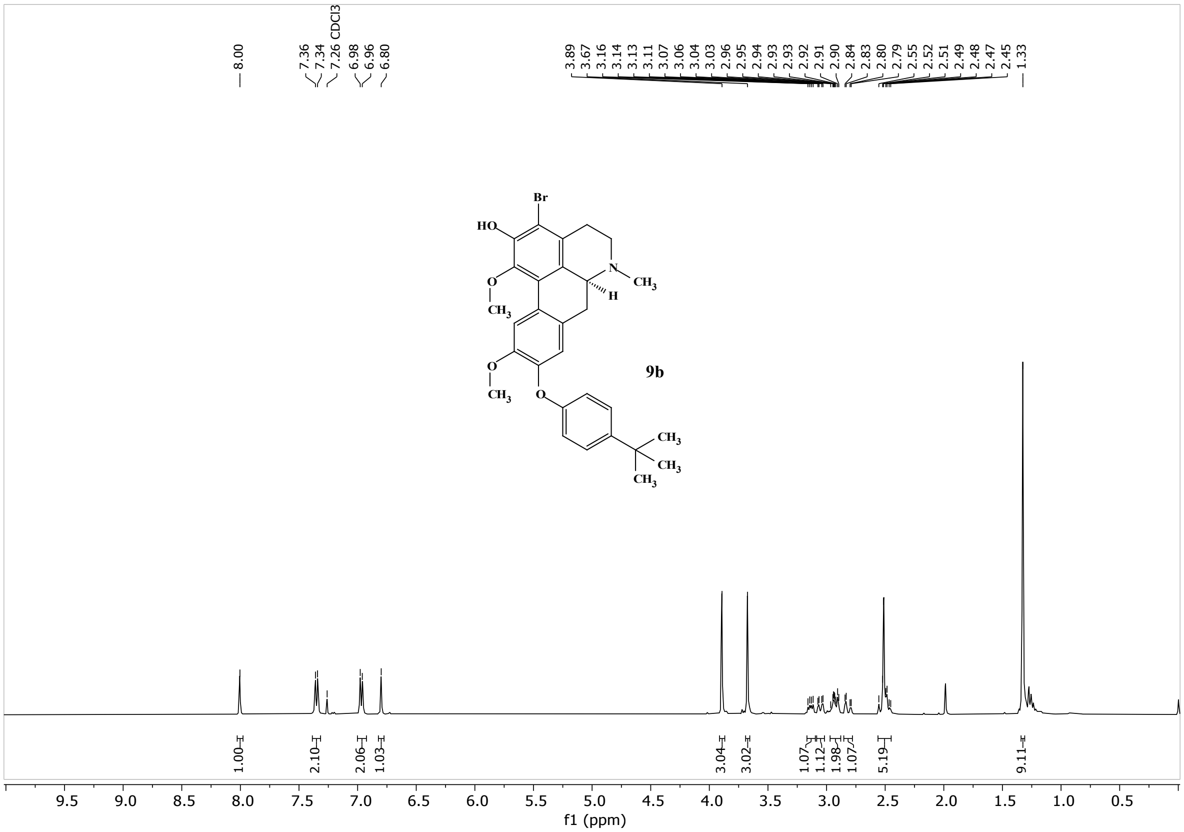

Figure S111. ^{1}H-NMR spectrum of **9b** ($CDCl_3$, 400 MHz).

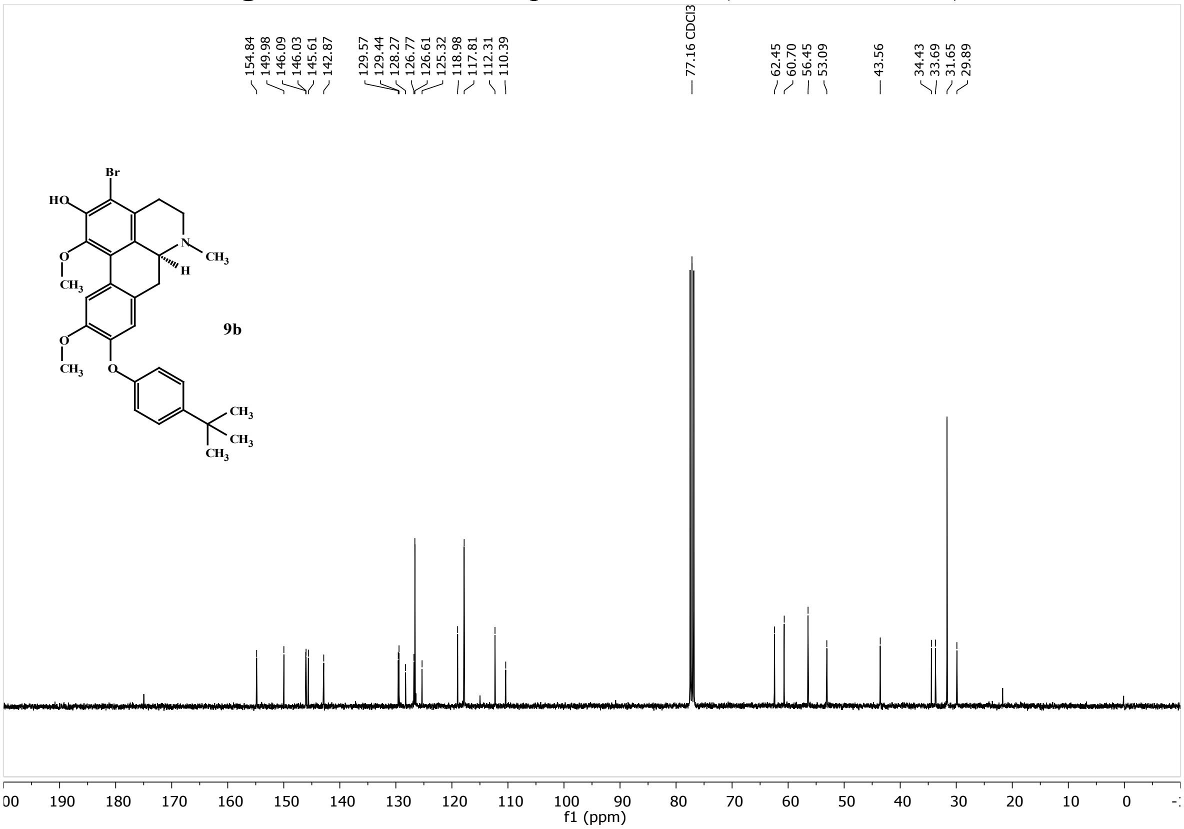

Figure S112. ^{13}C-NMR spectrum of **9b** ($CDCl_3$, 101 MHz).

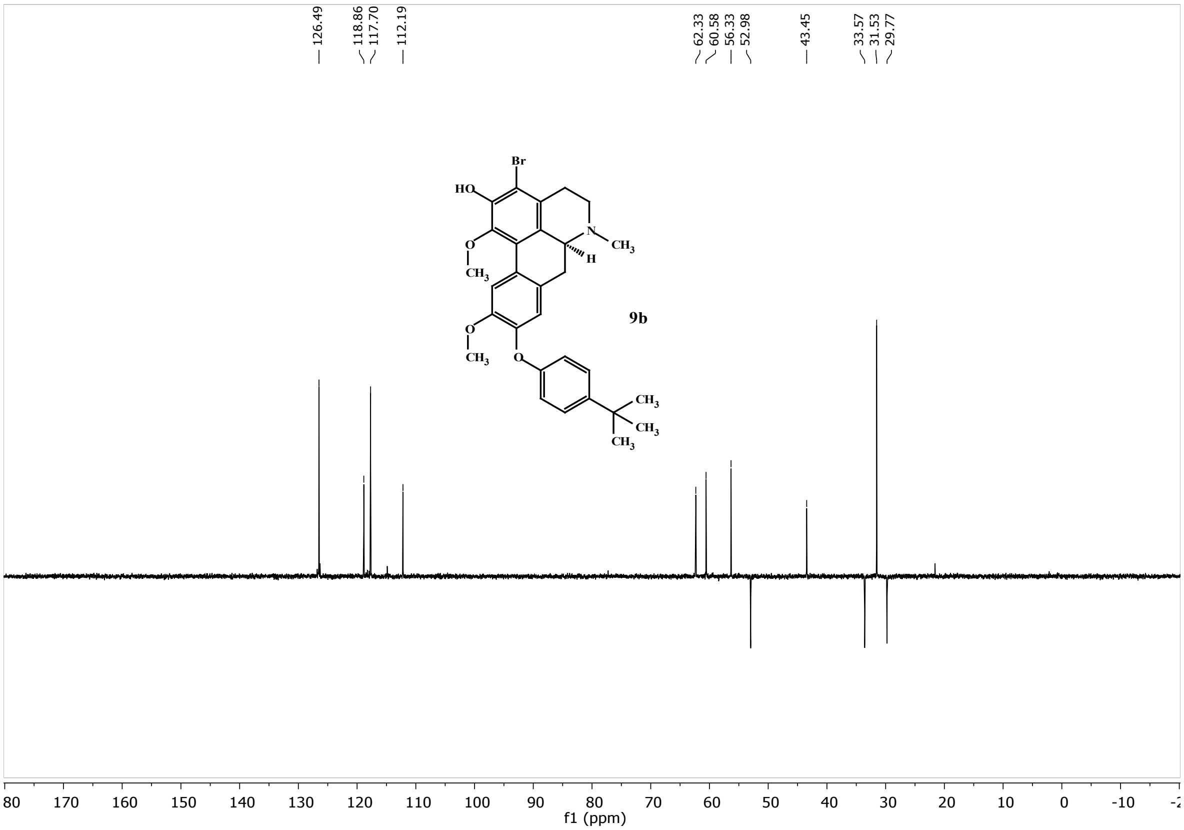

Figure S113. DEPT spectrum of **9b** ($CDCl_3$, 101 MHz).

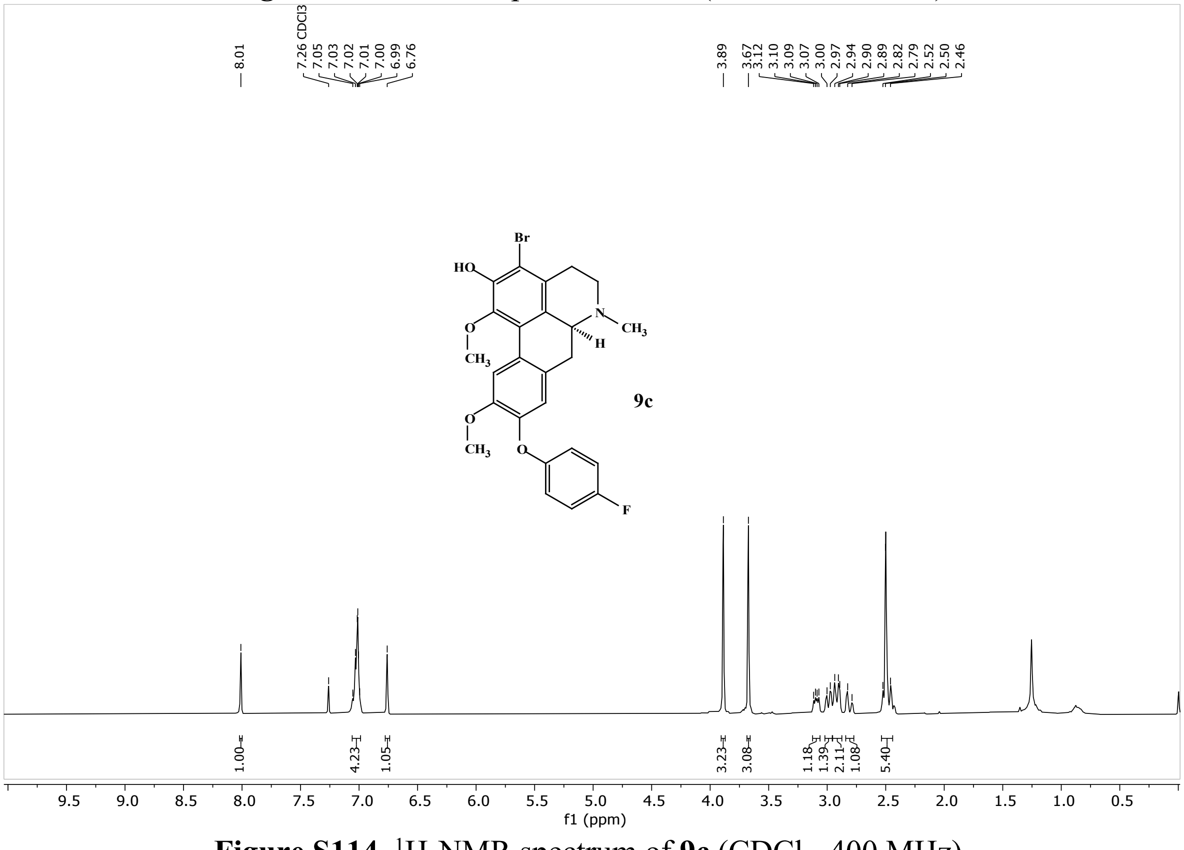

Figure S114. ^{1}H-NMR spectrum of **9c** ($CDCl_3$, 400 MHz).

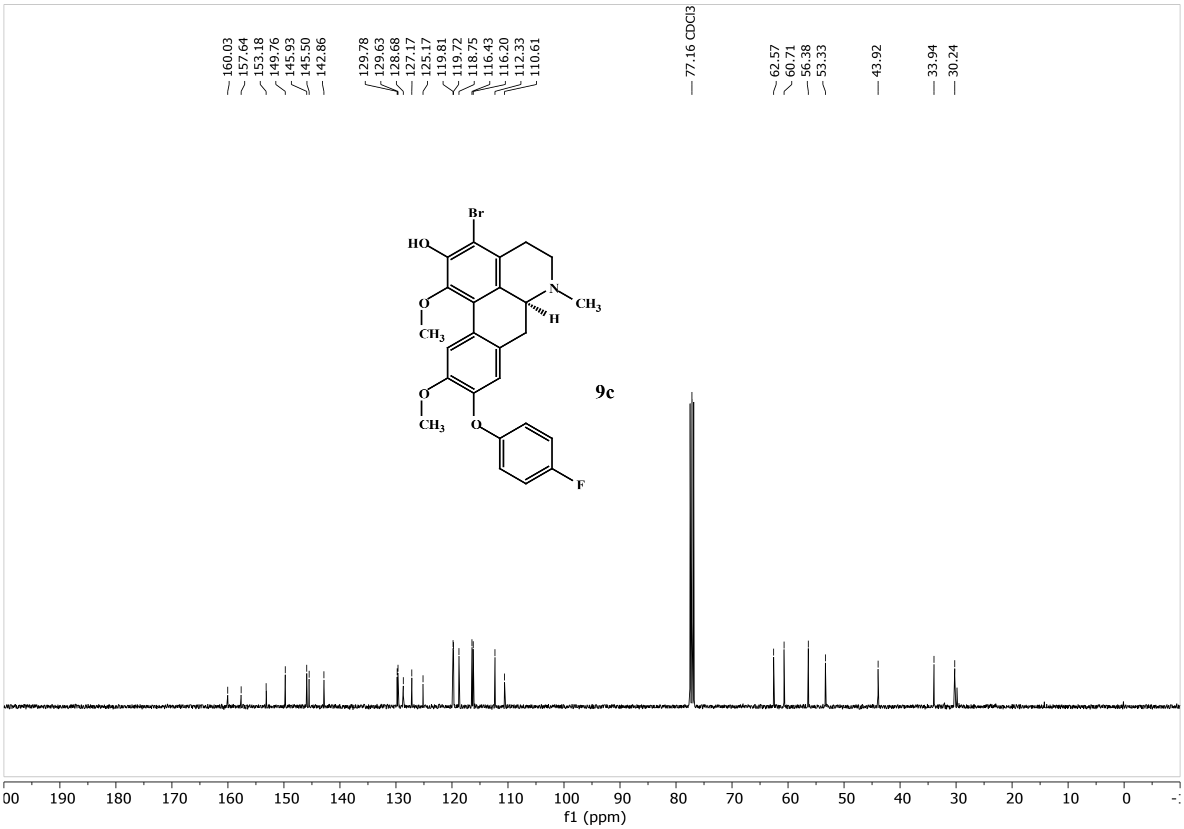

Figure S115. ^{13}C-NMR spectrum of **9c** ($CDCl_3$, 101 MHz).

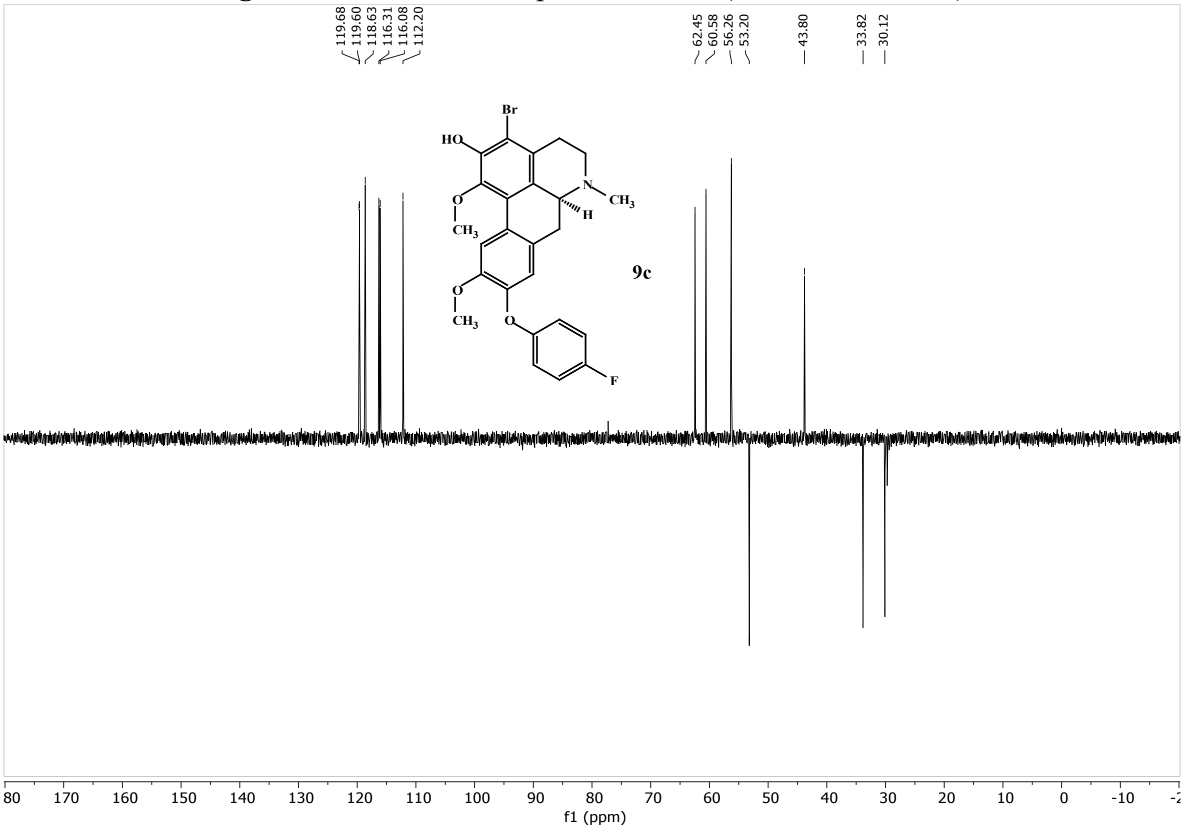

Figure S116. DEPT spectrum of **9c** ($CDCl_3$, 101 MHz).

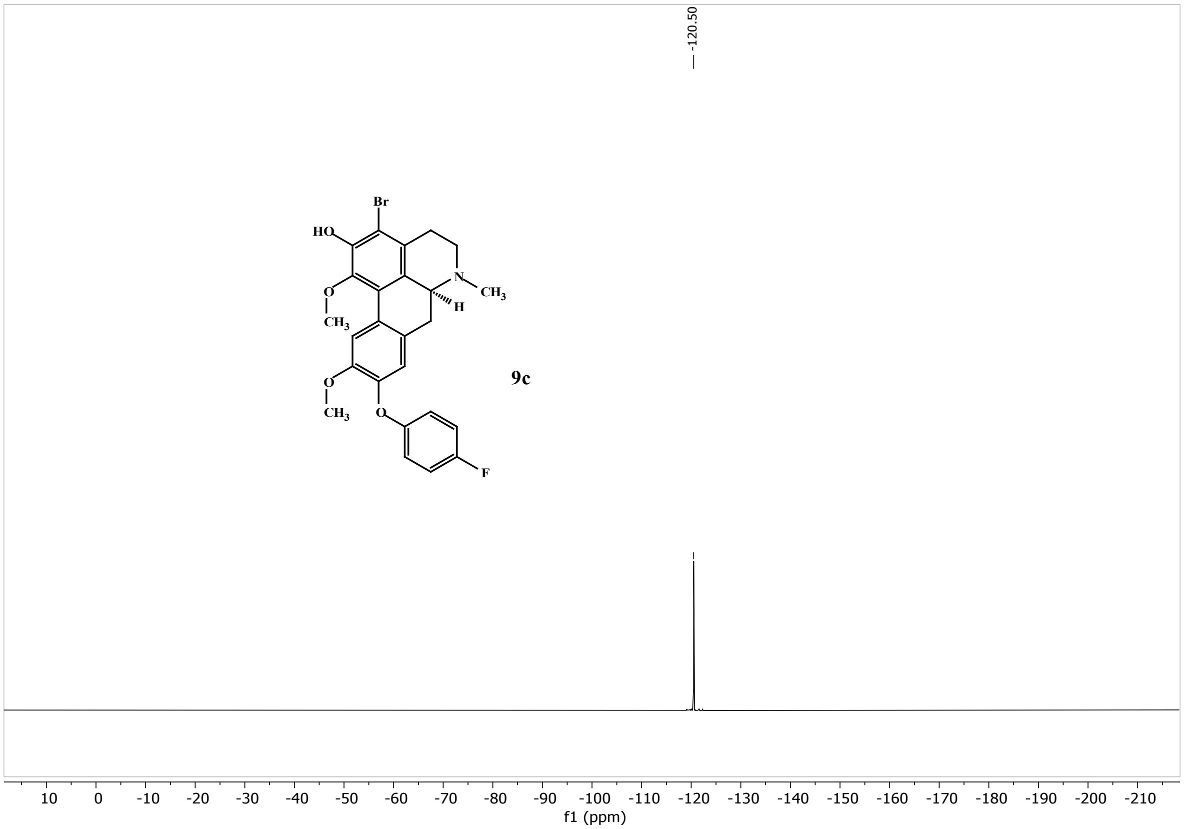

Figure S117. ^{19}F-NMR spectrum of **9c** ($CDCl_3$, 376 MHz).

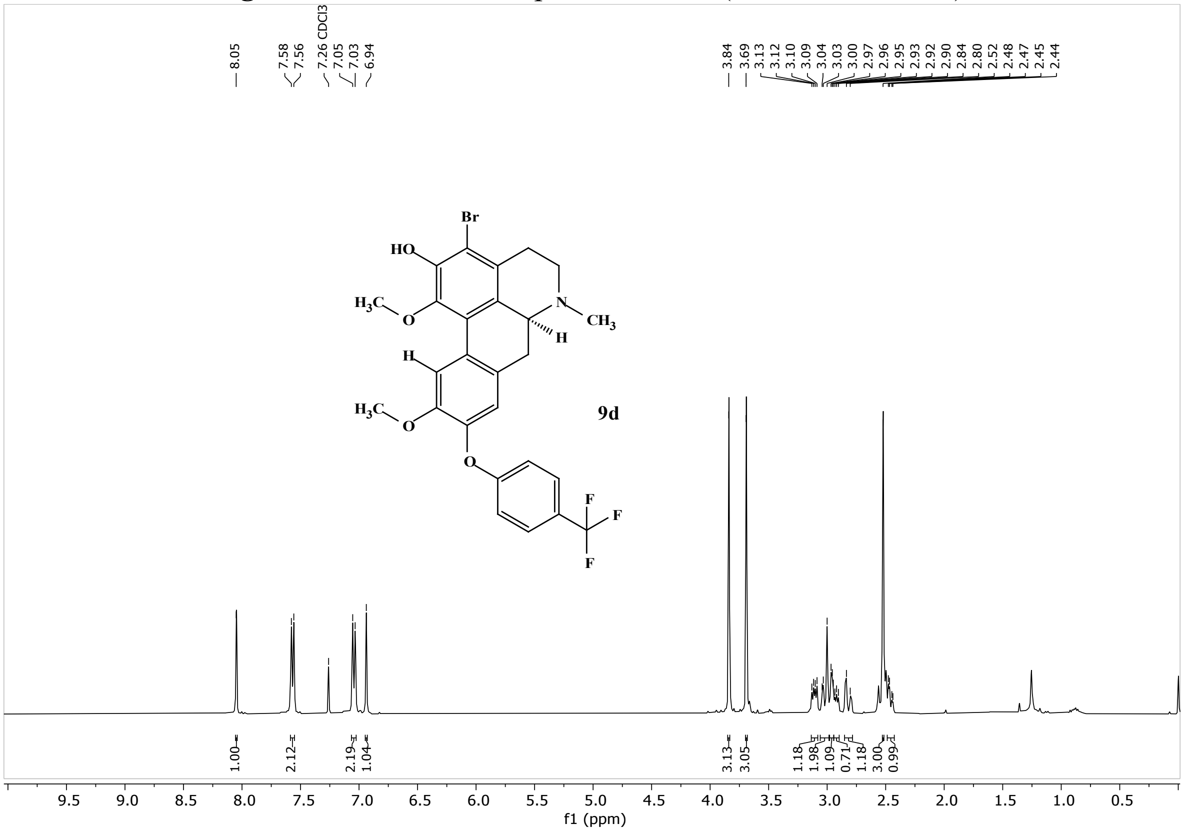

Figure S118. ^{1}H-NMR spectrum of **9d** ($CDCl_3$, 400 MHz).

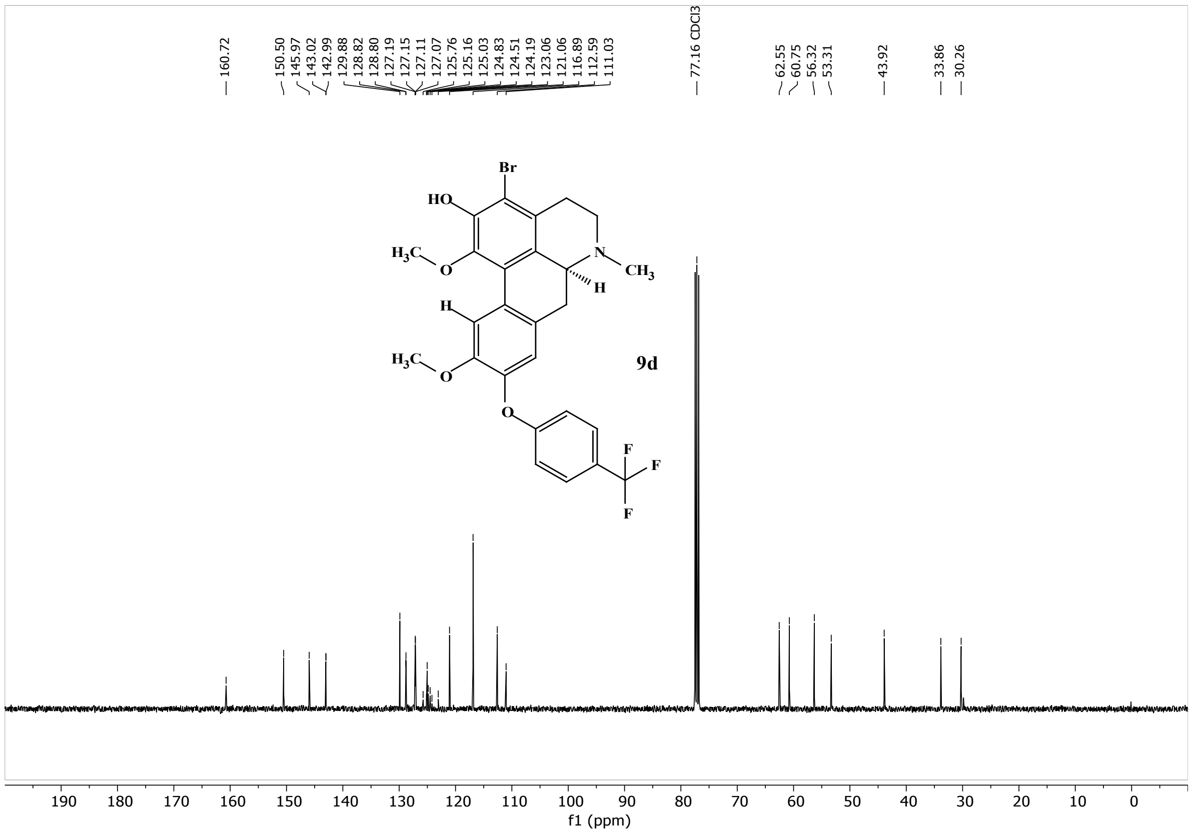

Figure S119. ^{13}C-NMR spectrum of **9d** ($CDCl_3$, 101 MHz).

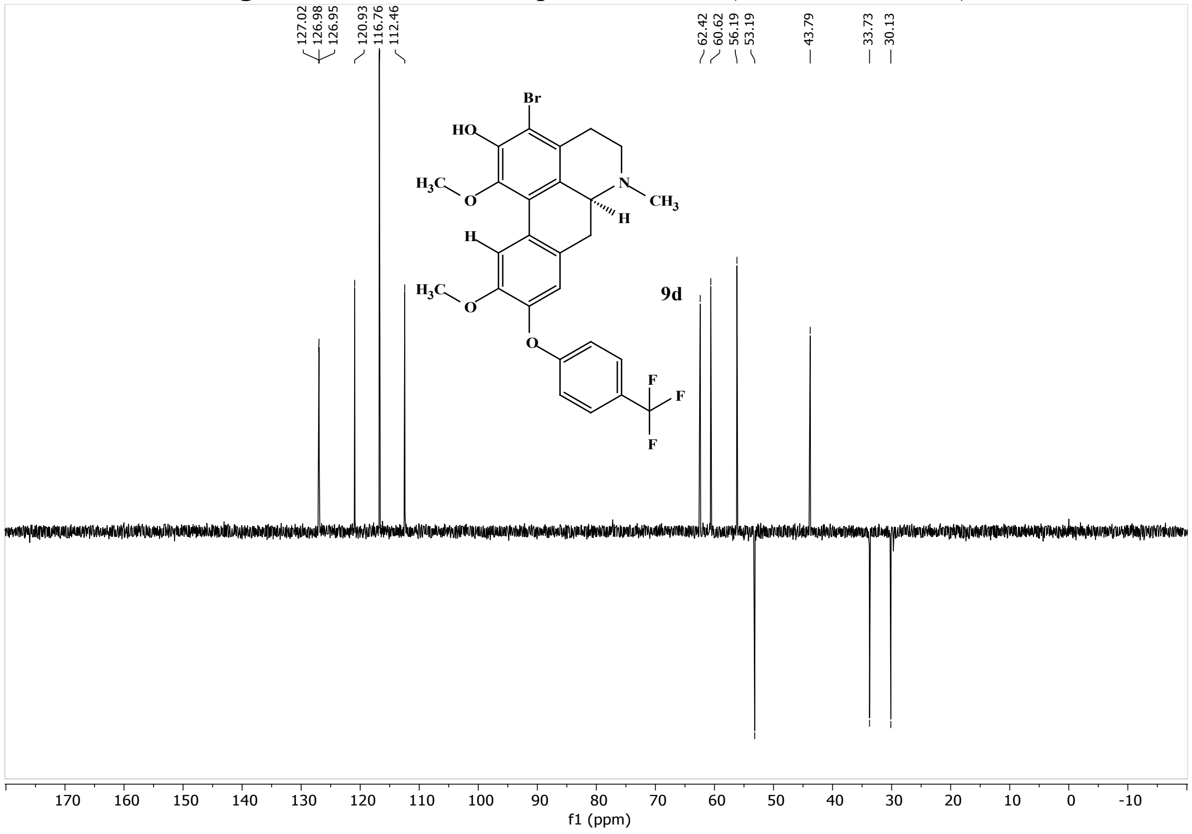

Figure S120. DEPT spectrum of **9d** ($CDCl_3$, 101 MHz).

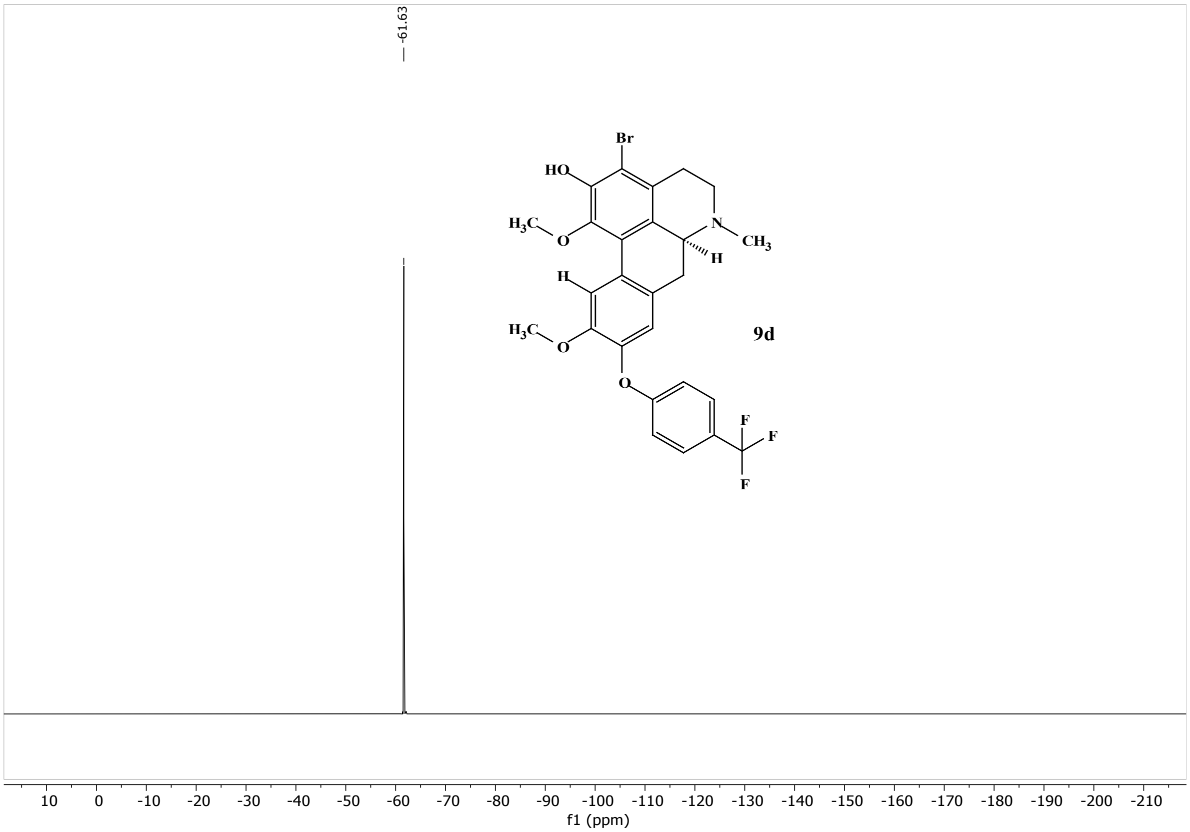

Figure S121. ^{19}F-NMR spectrum of **9d** ($CDCl_3$, 376 MHz).

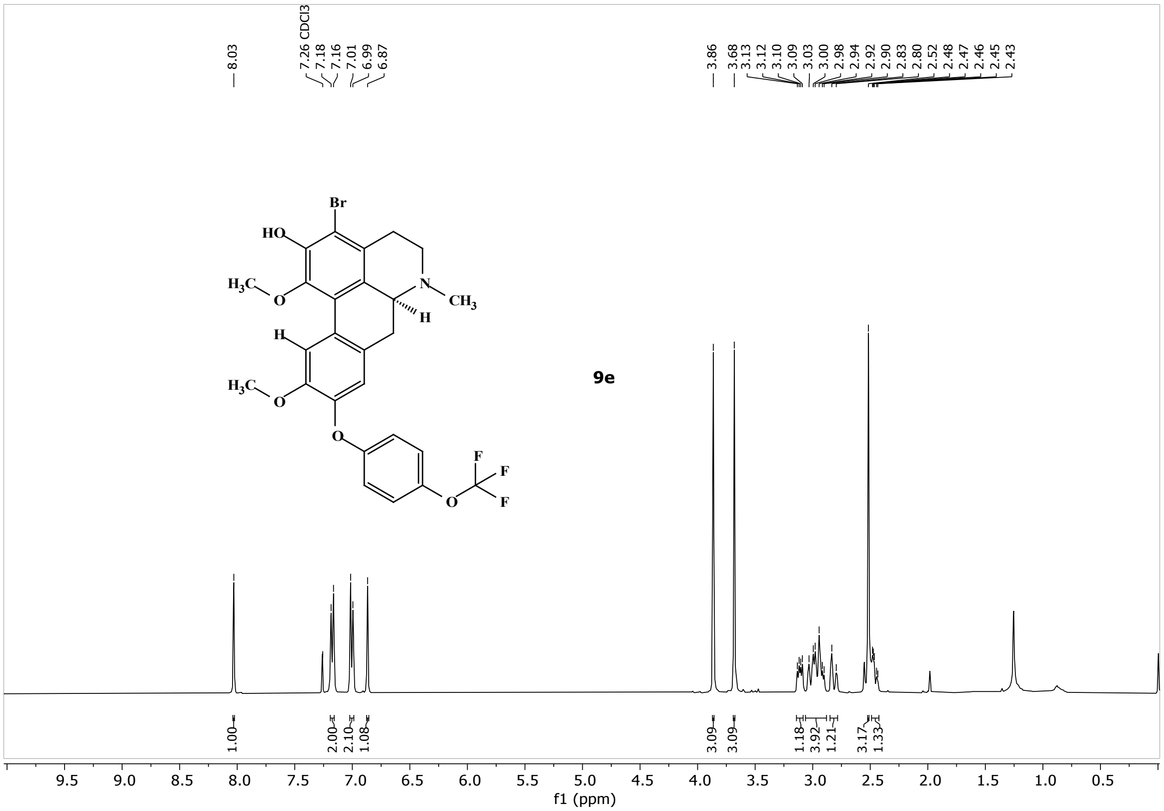

Figure S122. ^{1}H-NMR spectrum of **9e** ($CDCl_3$, 400 MHz).

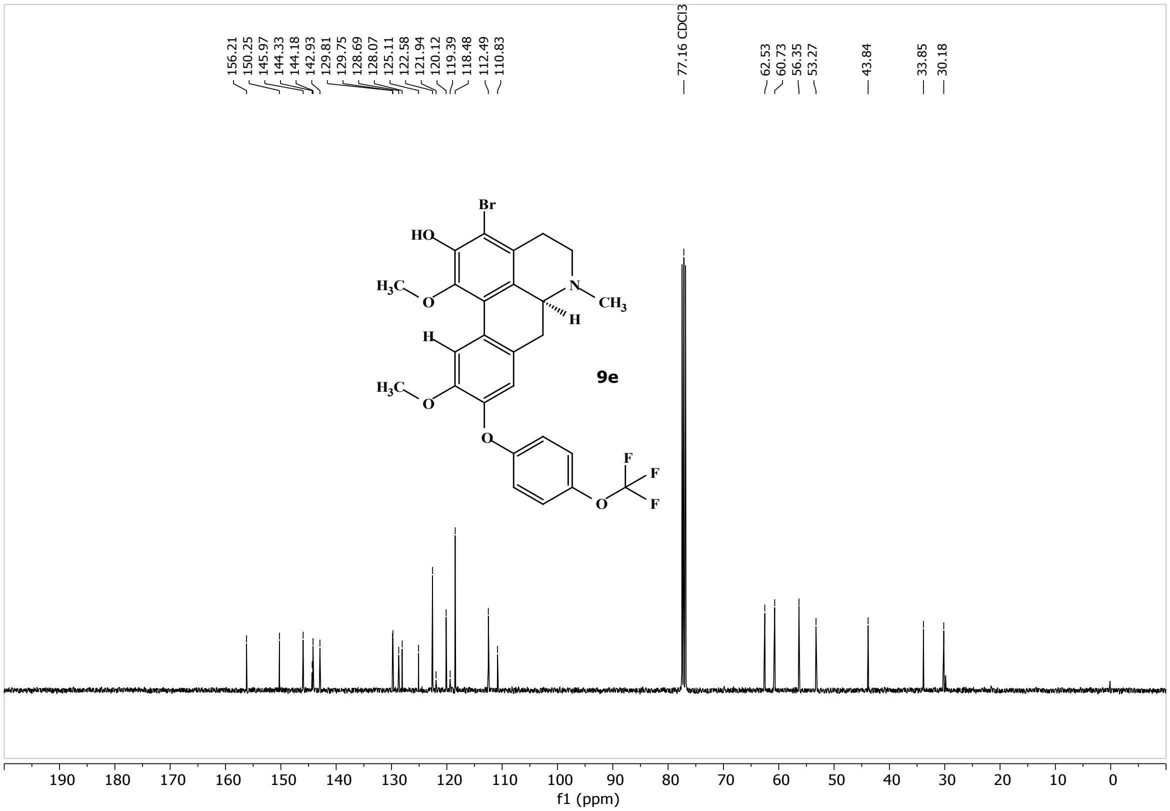

Figure S123. ^{13}C-NMR spectrum of **9e** ($CDCl_3$, 101 MHz).

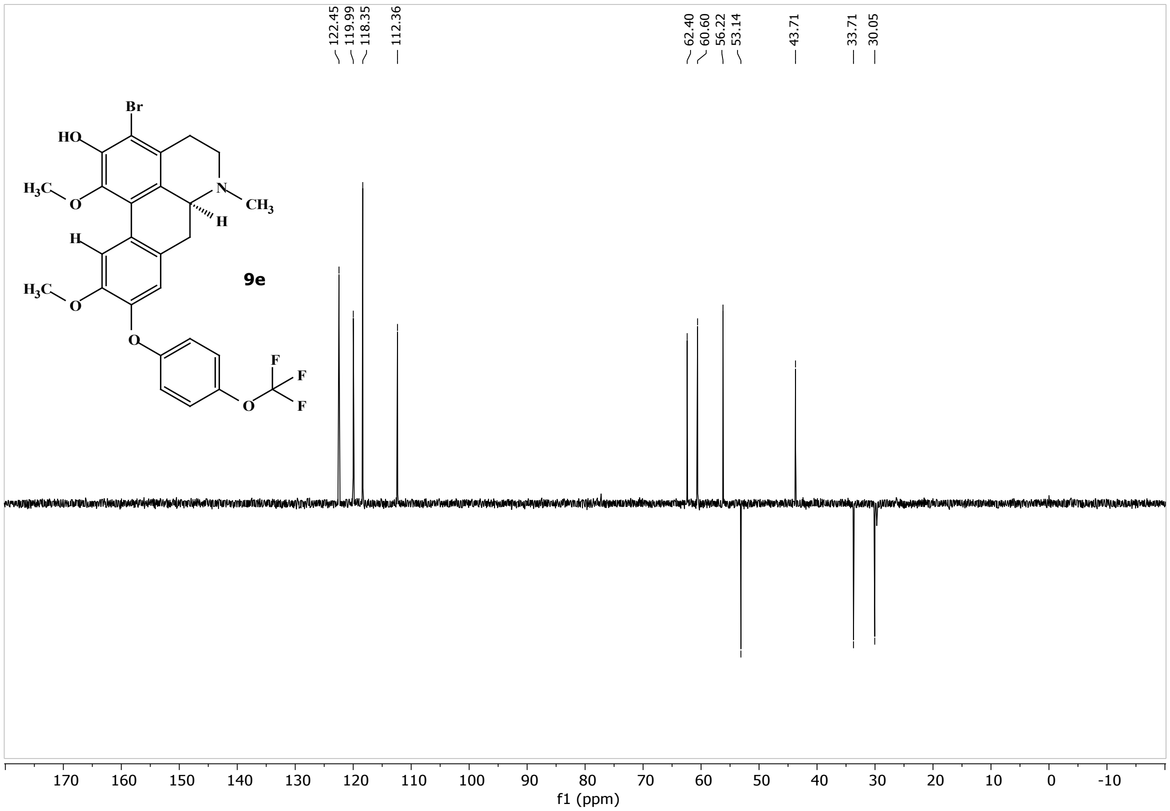

Figure S124. DEPT spectrum of **9e** ($CDCl_3$, 101 MHz).

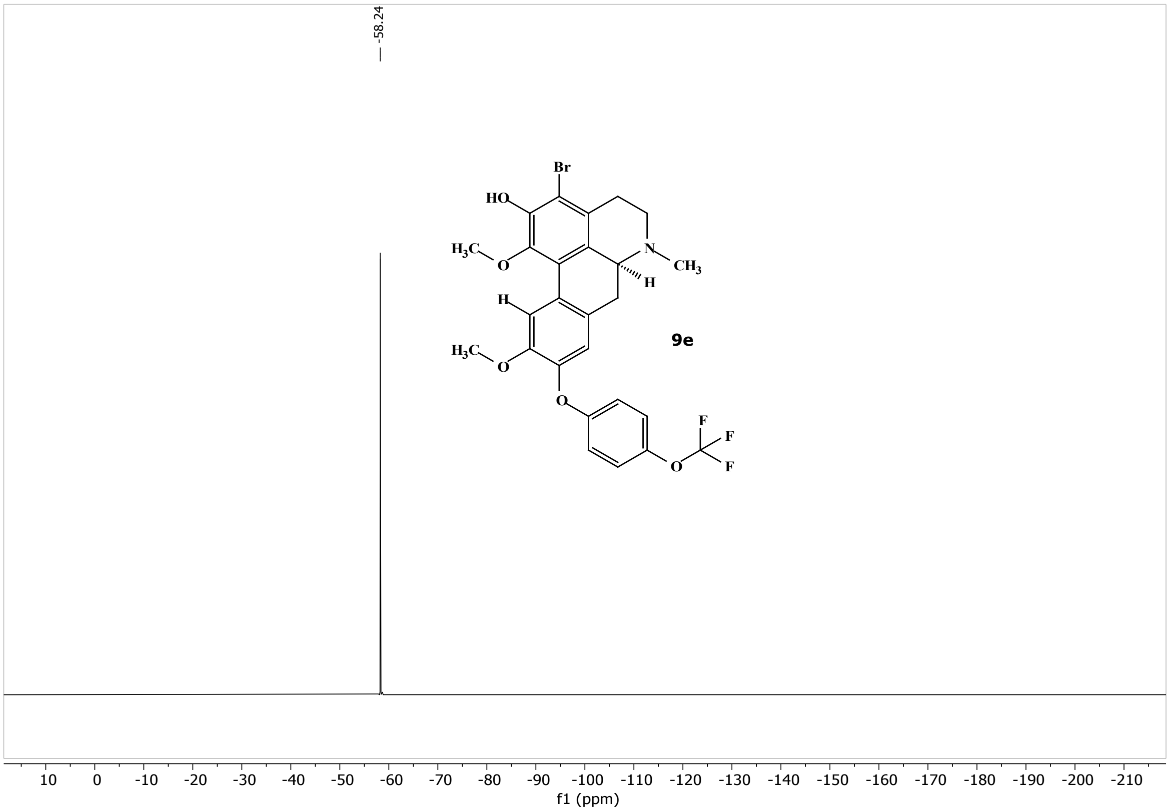

Figure S125. ^{19}F-NMR spectrum of **9c** ($CDCl_3$, 376 MHz).

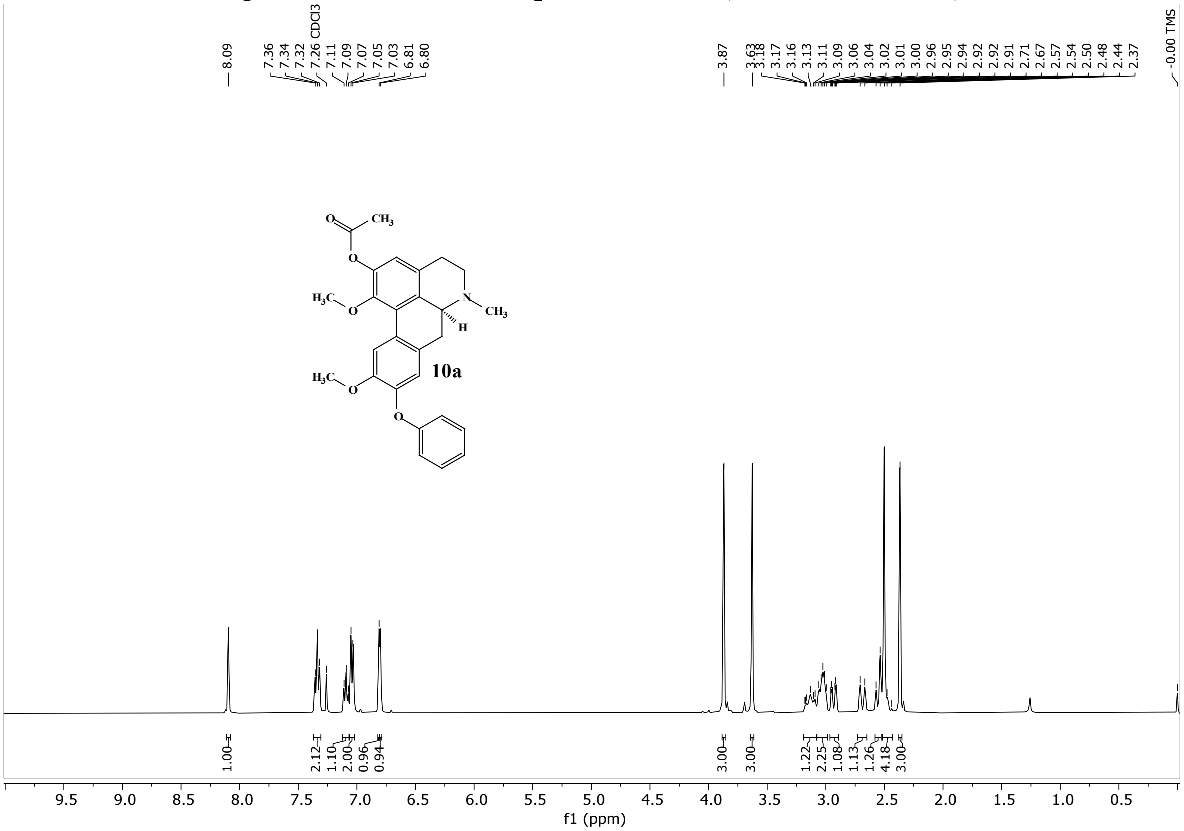

Figure S126. ^{1}H-NMR spectrum of **10a** ($CDCl_3$, 400 MHz).

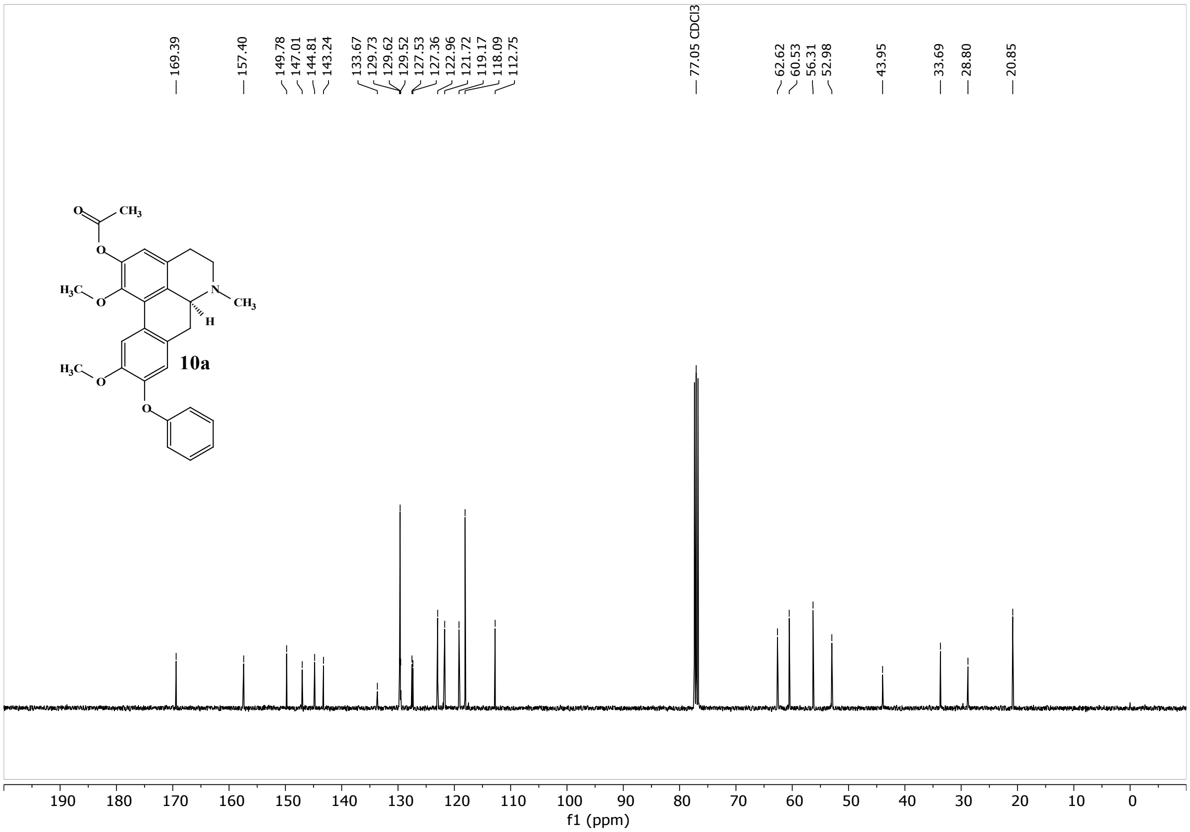

Figure S127. ^{13}C-NMR spectrum of **10a** ($CDCl_3$, 101 MHz).

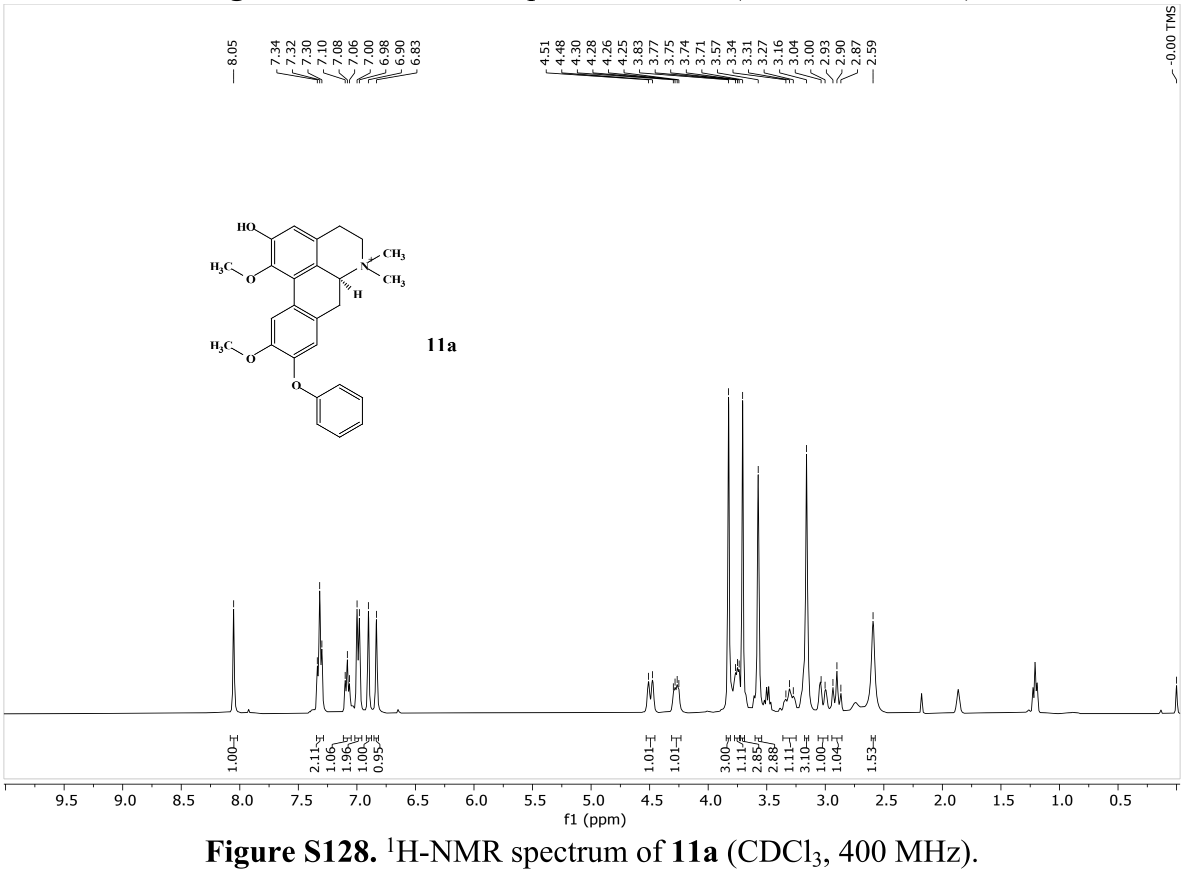

Figure S128. ^{1}H-NMR spectrum of **11a** ($CDCl_3$, 400 MHz).

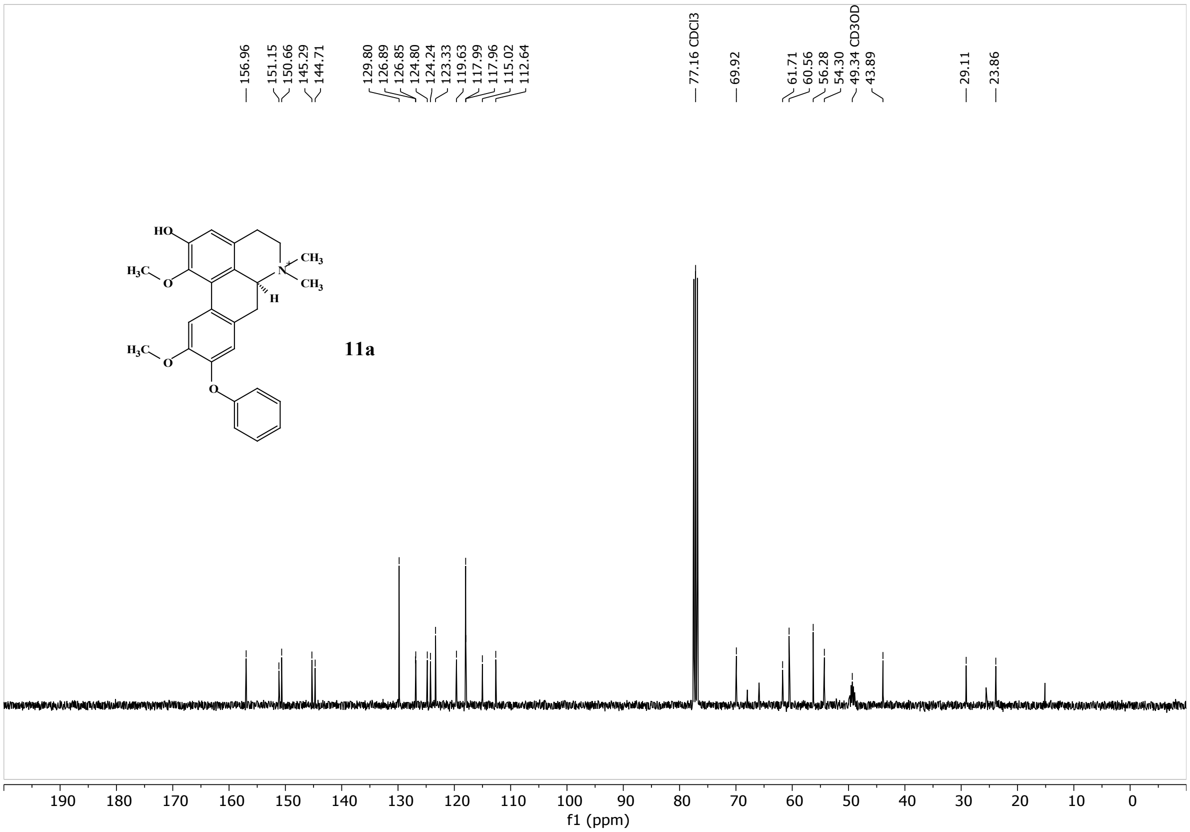

Figure S129. ^{13}C-NMR spectrum of **11a** ($CDCl_3$, 101 MHz).

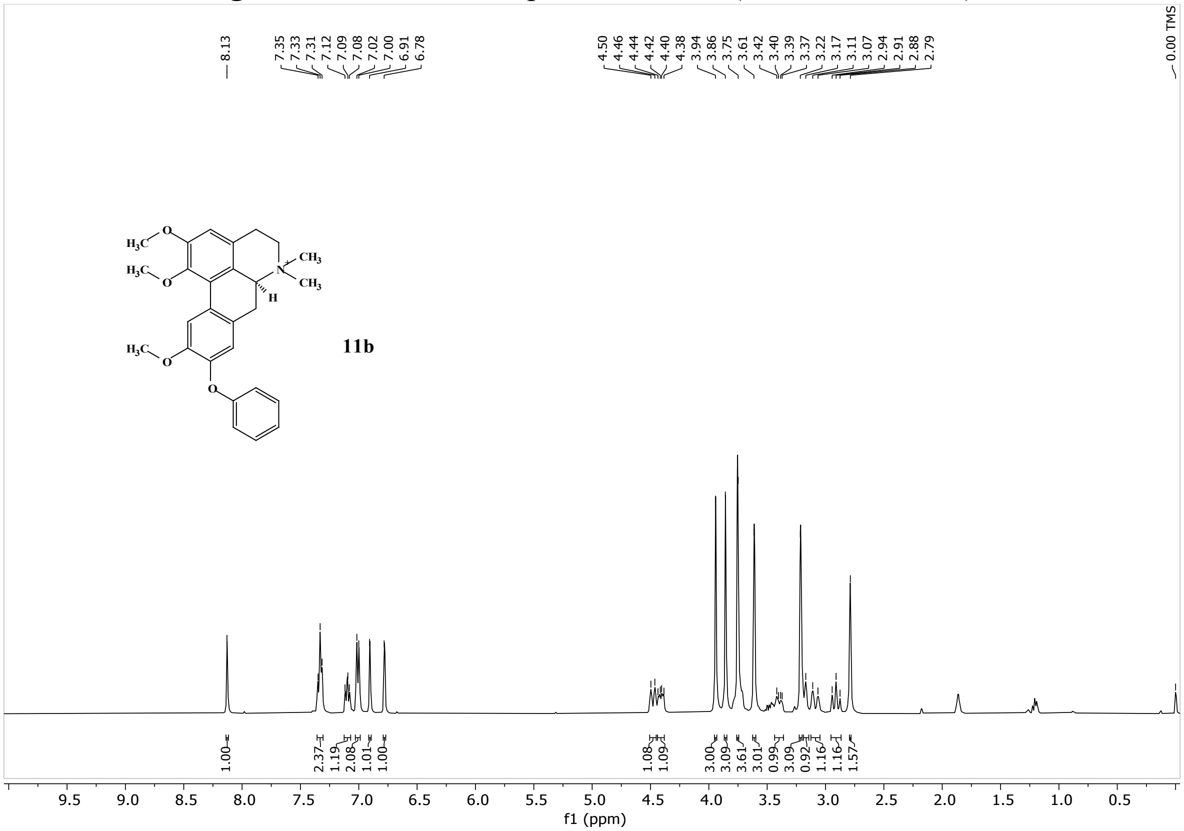

Figure S130. ^{1}H-NMR spectrum of **11b** ($CDCl_3$, 400 MHz).

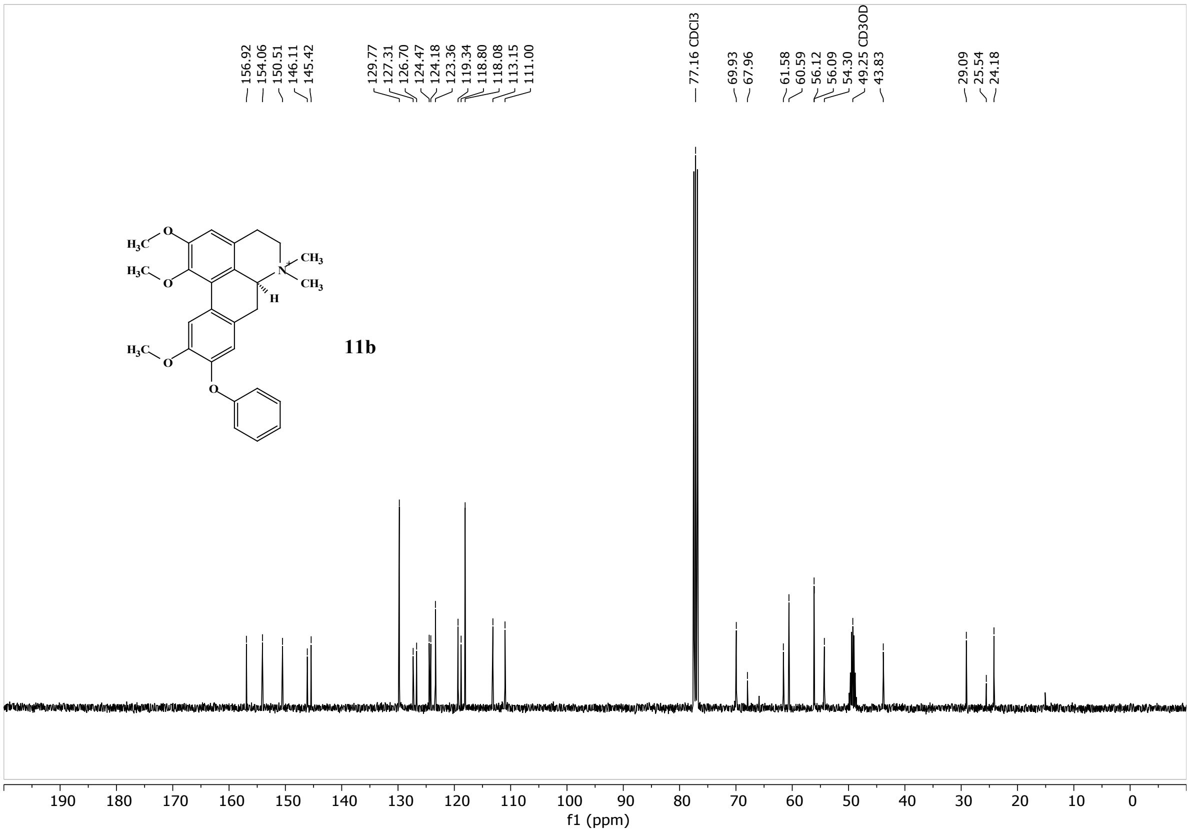

Figure S131. ^{13}C-NMR spectrum of **11b** ($CDCl_3$, 101 MHz).

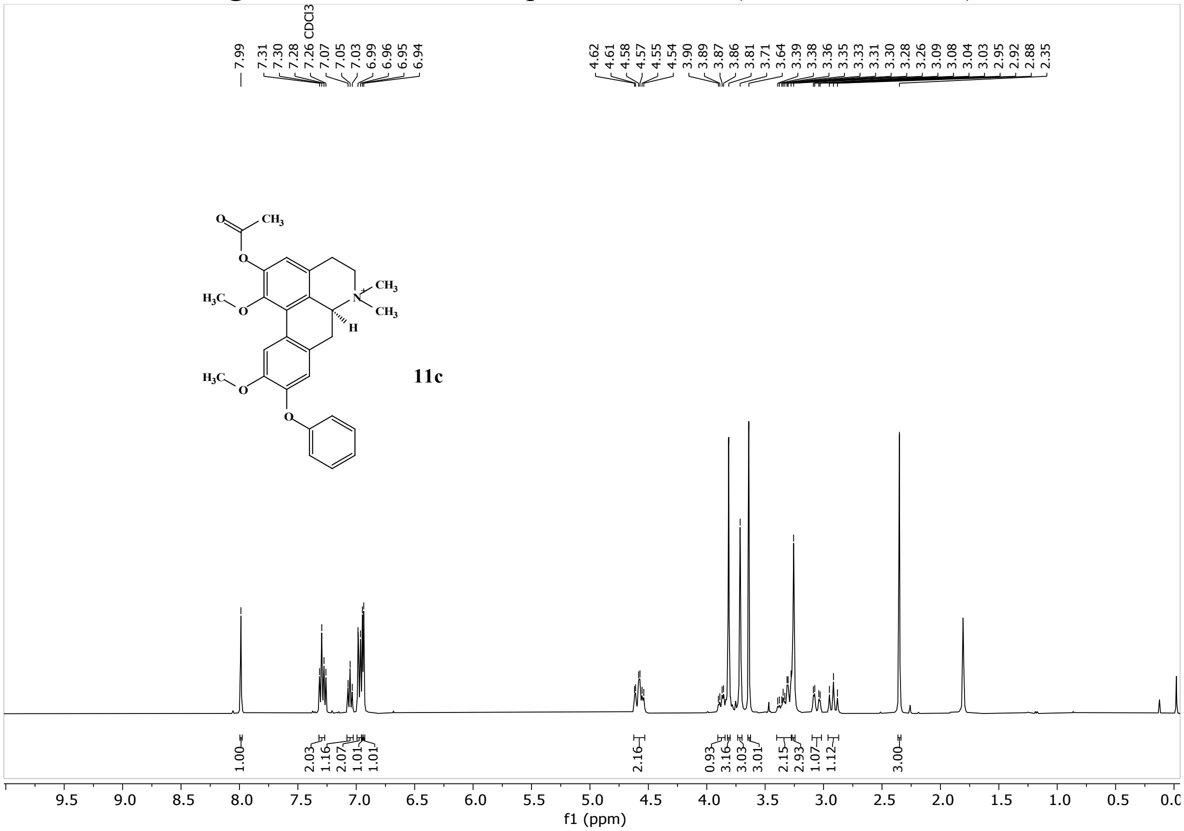

Figure S132. ^{1}H-NMR spectrum of **11c** ($CDCl_3$, 400 MHz).

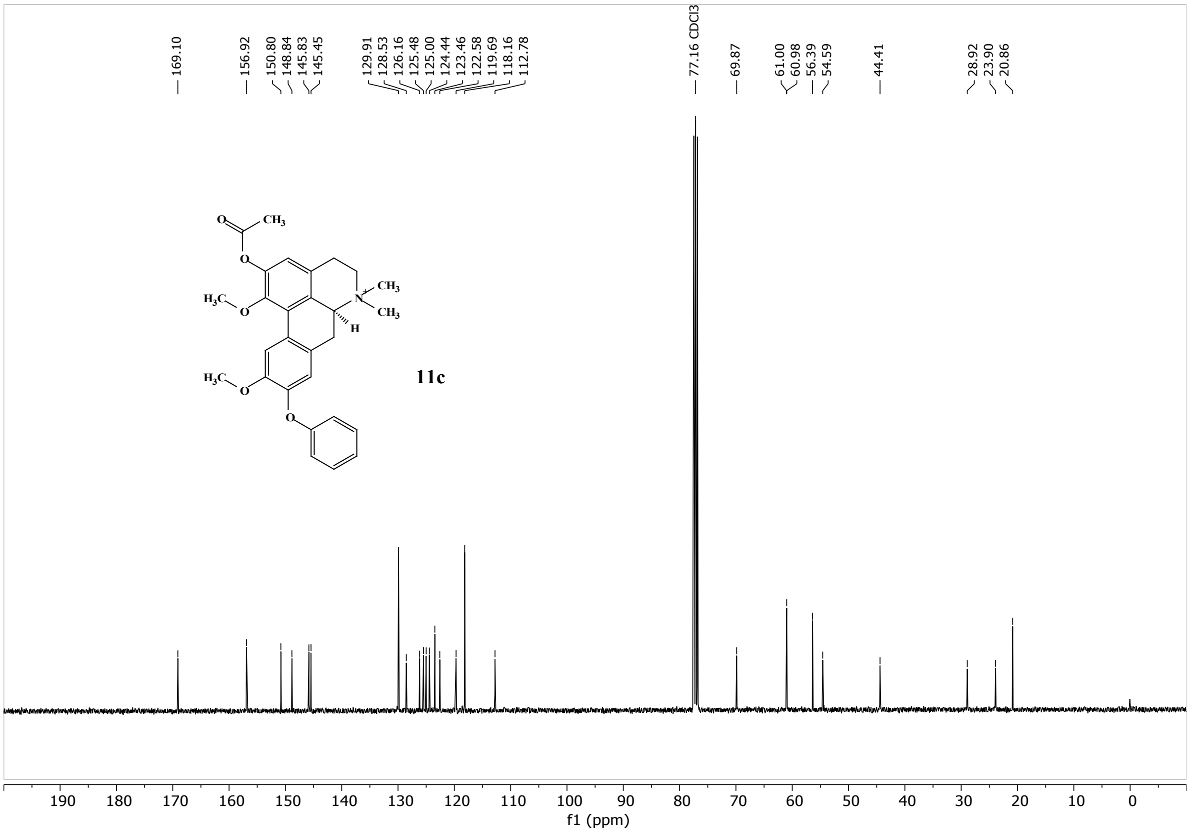

Figure S133. ^{13}C-NMR spectrum of **11c** ($CDCl_3$, 101 MHz).

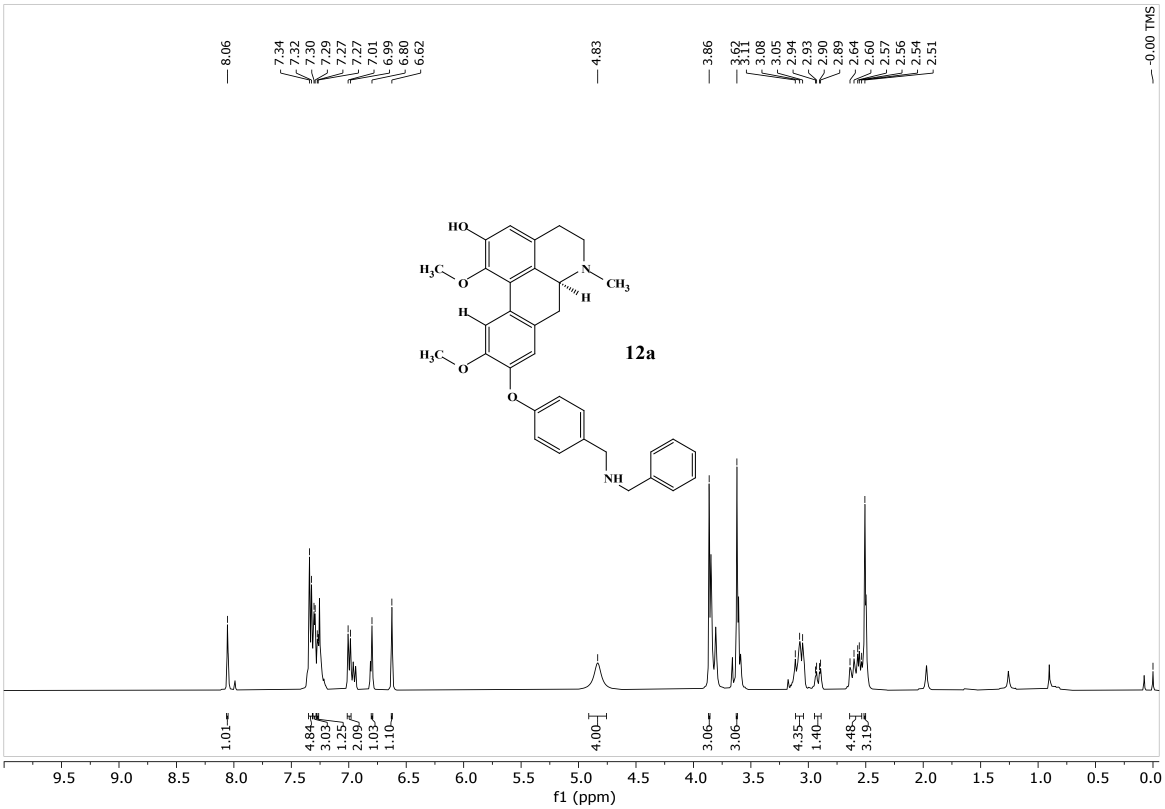

Figure S134. ^{1}H-NMR spectrum of **12a** ($CDCl_3$, 400 MHz).

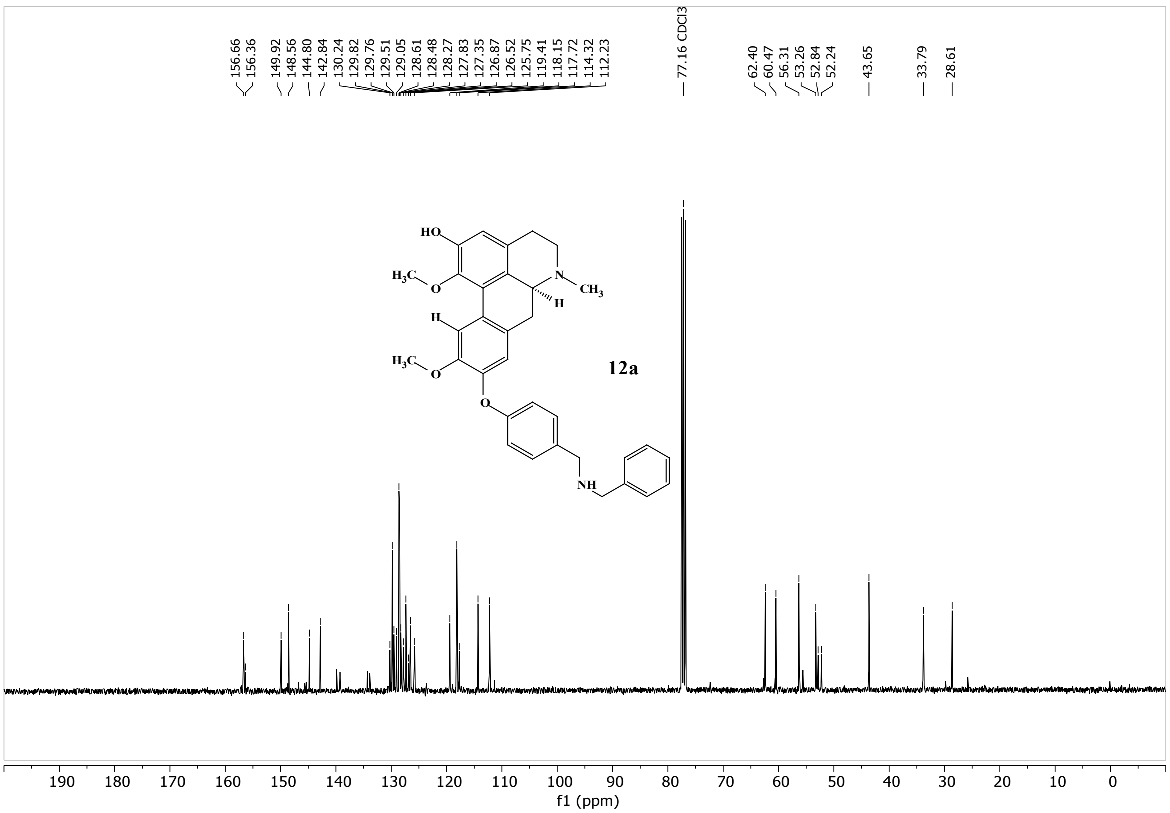

Figure S135. ^{13}C-NMR spectrum of **12a** ($CDCl_3$, 101 MHz).

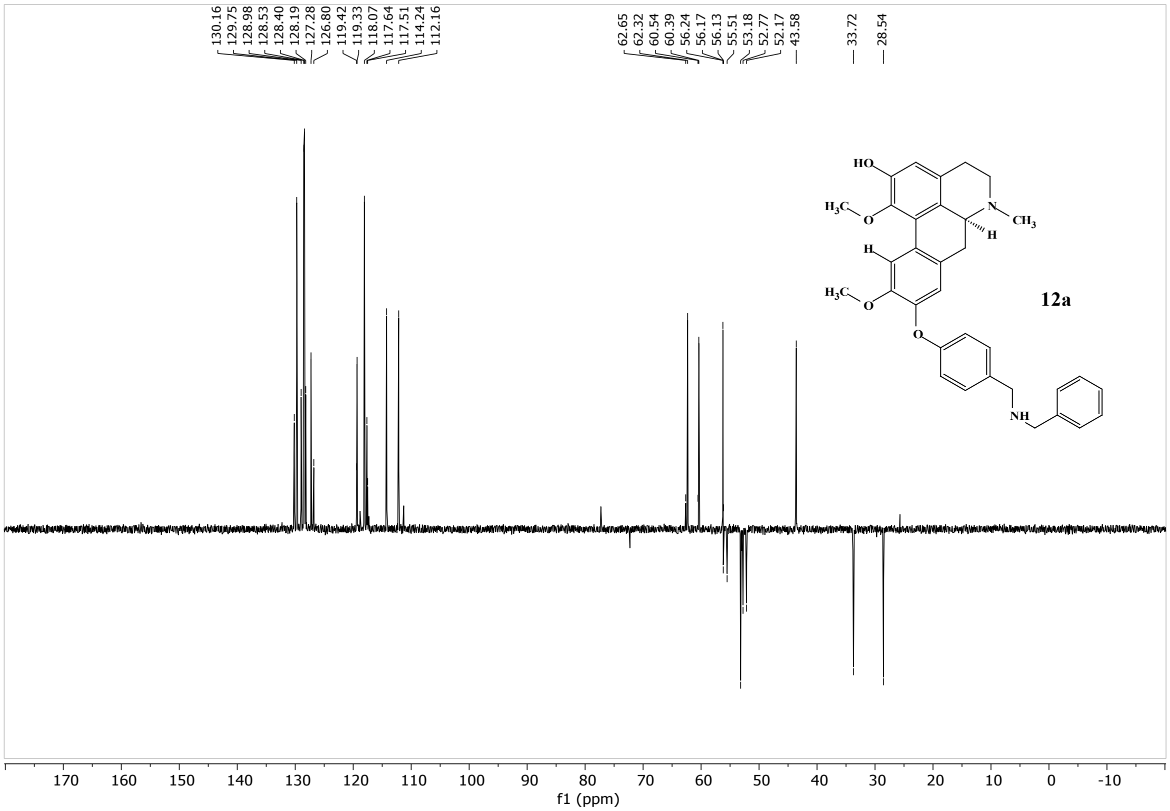

Figure S136. DEPT spectrum of **12a** ($CDCl_3$, 101 MHz).

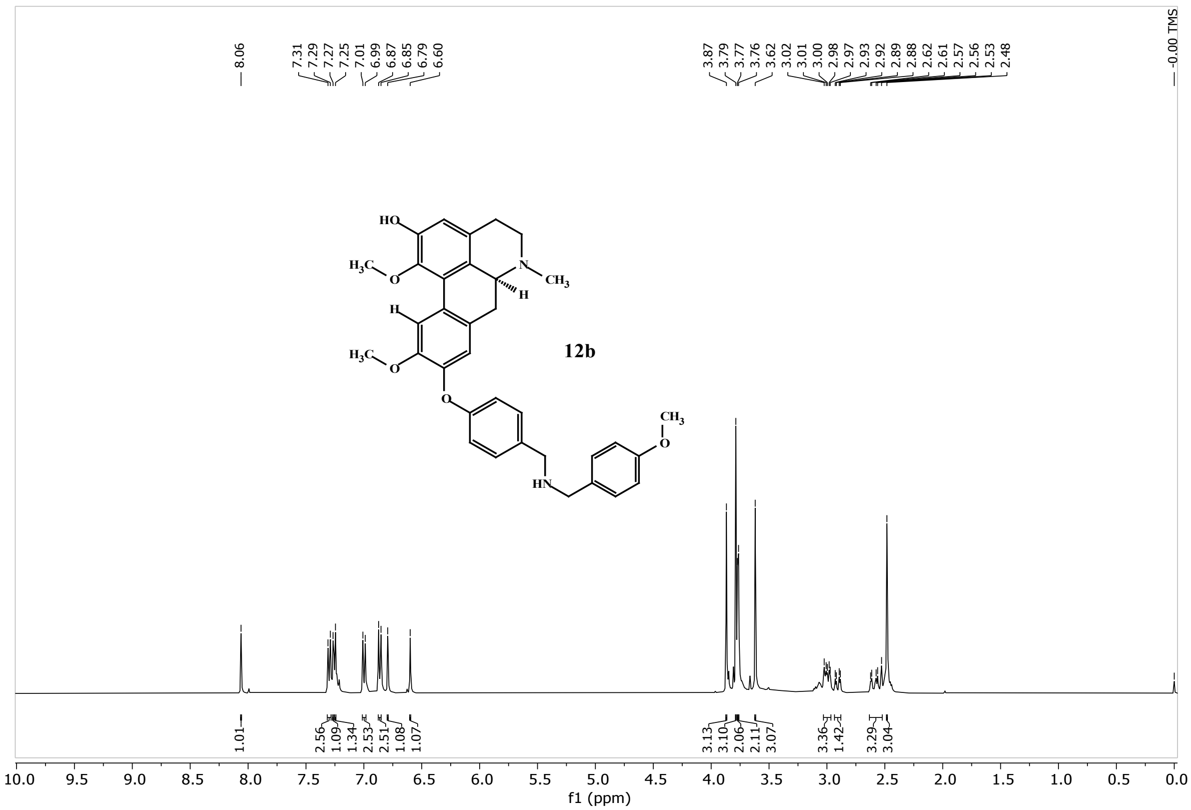

Figure S137. ^{1}H-NMR spectrum of **12b** ($CDCl_3$, 400 MHz).

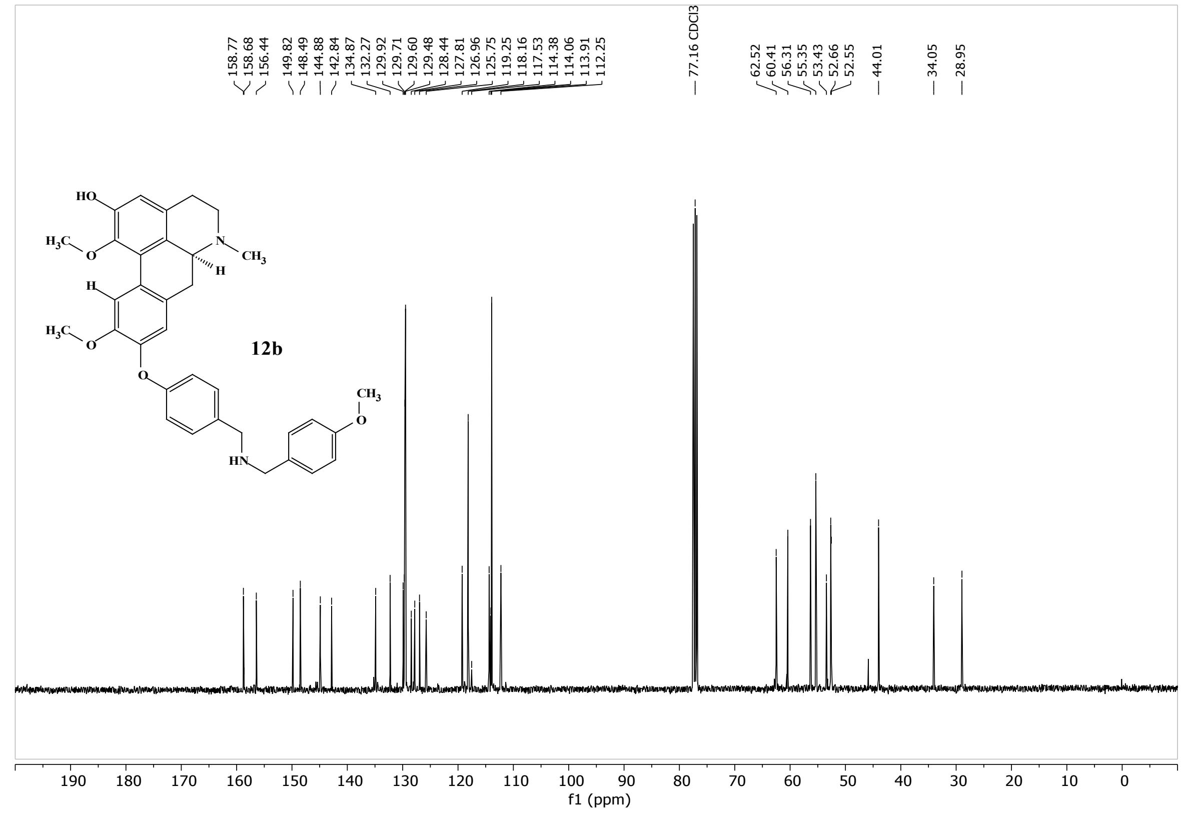

Figure S138. ^{13}C-NMR spectrum of **12b** ($CDCl_3$, 101 MHz).

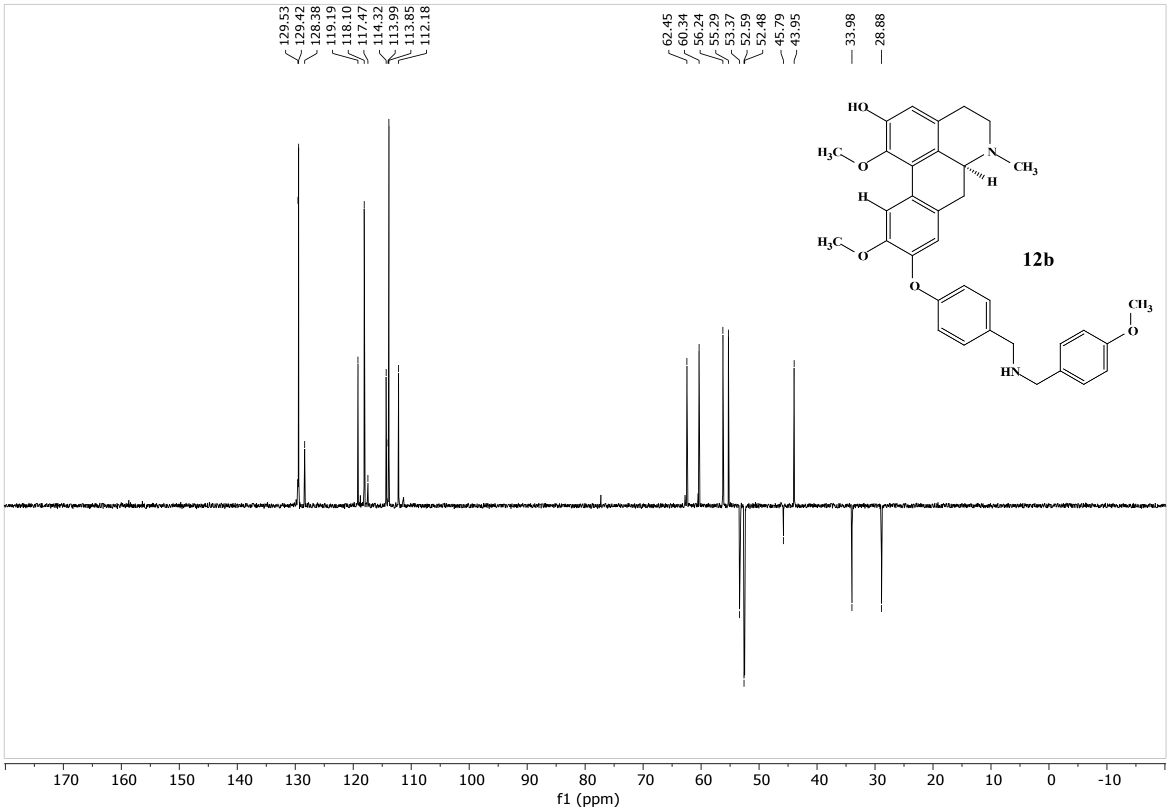

Figure S139. DEPT spectrum of **12b** ($CDCl_3$, 101 MHz).

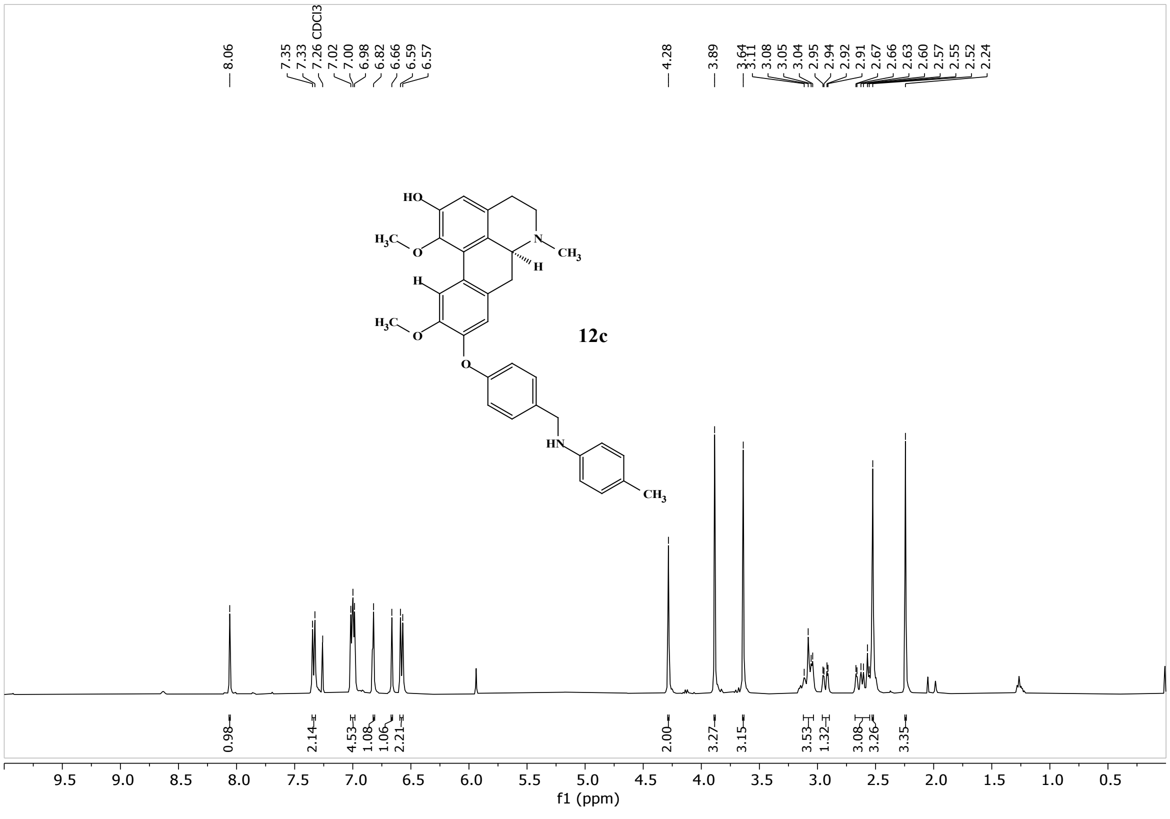

Figure S140. ^{1}H-NMR spectrum of **12c** ($CDCl_3$, 400 MHz).

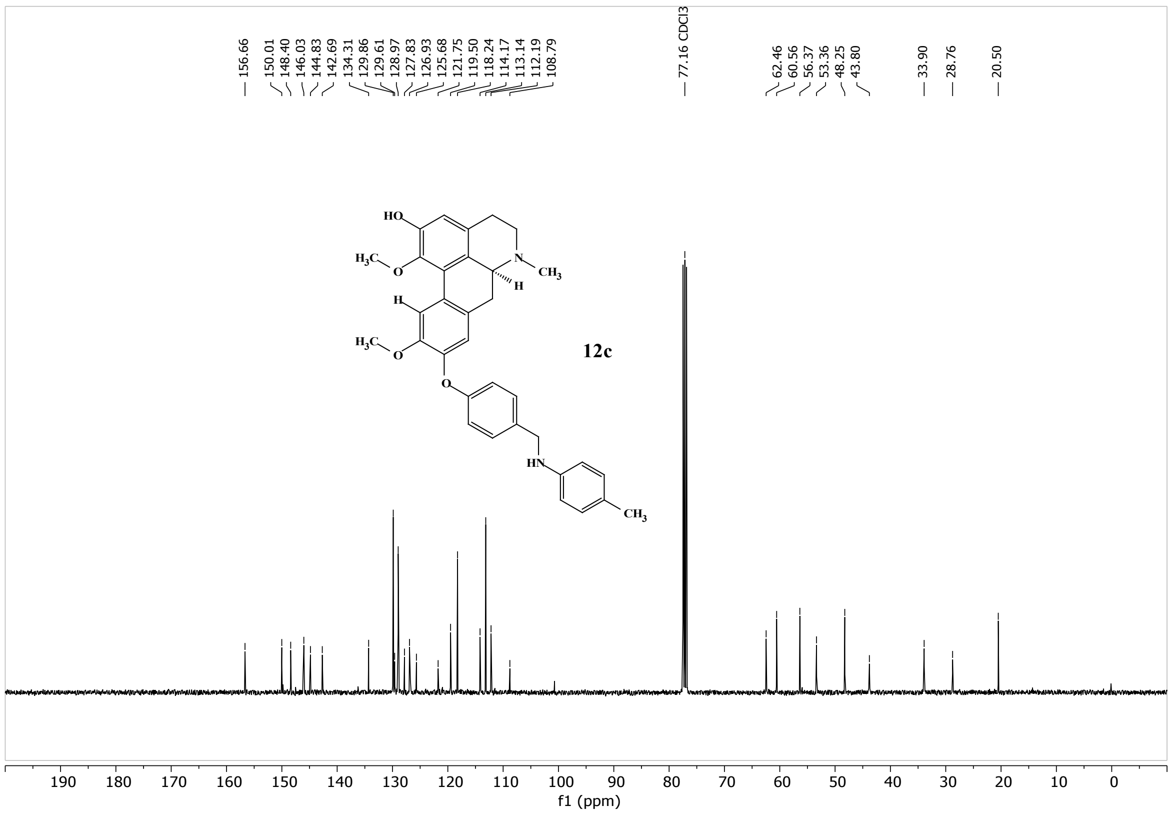

Figure S141. ^{13}C-NMR spectrum of **12c** ($CDCl_3$, 101 MHz).

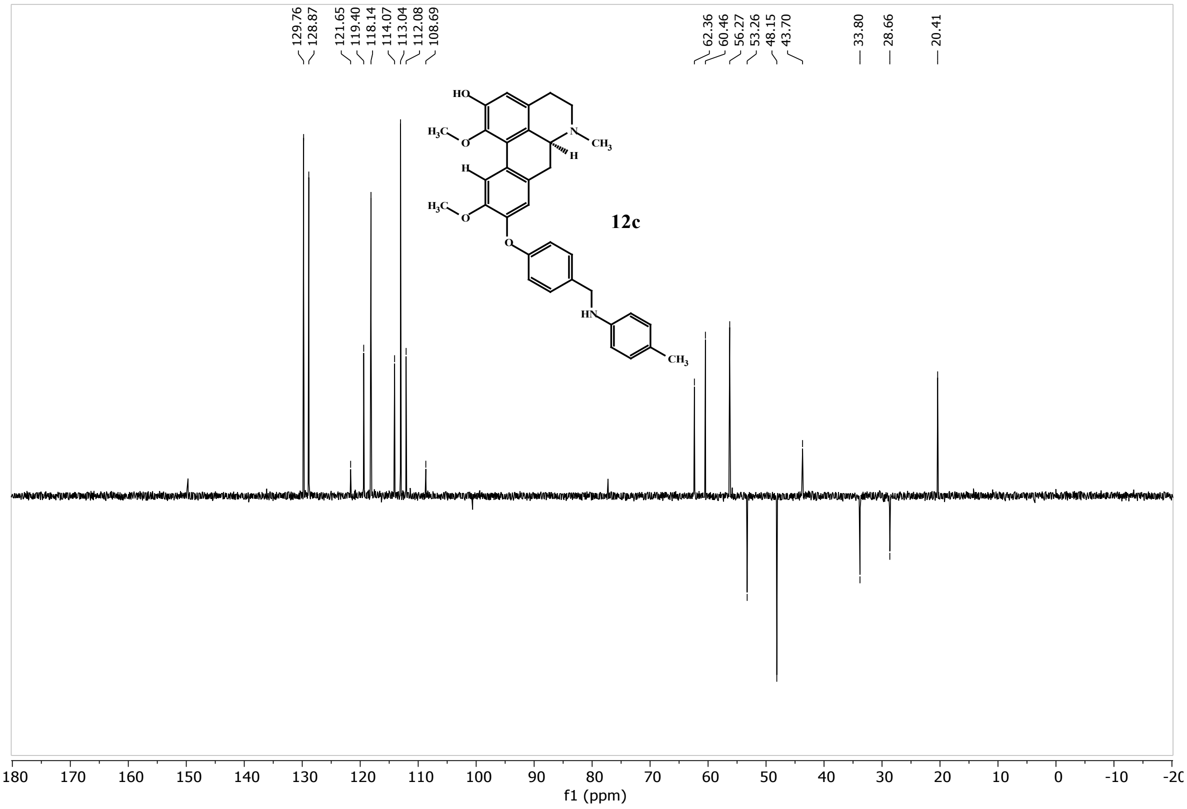

Figure S142. DEPT spectrum of **12c** ($CDCl_3$, 101 MHz).

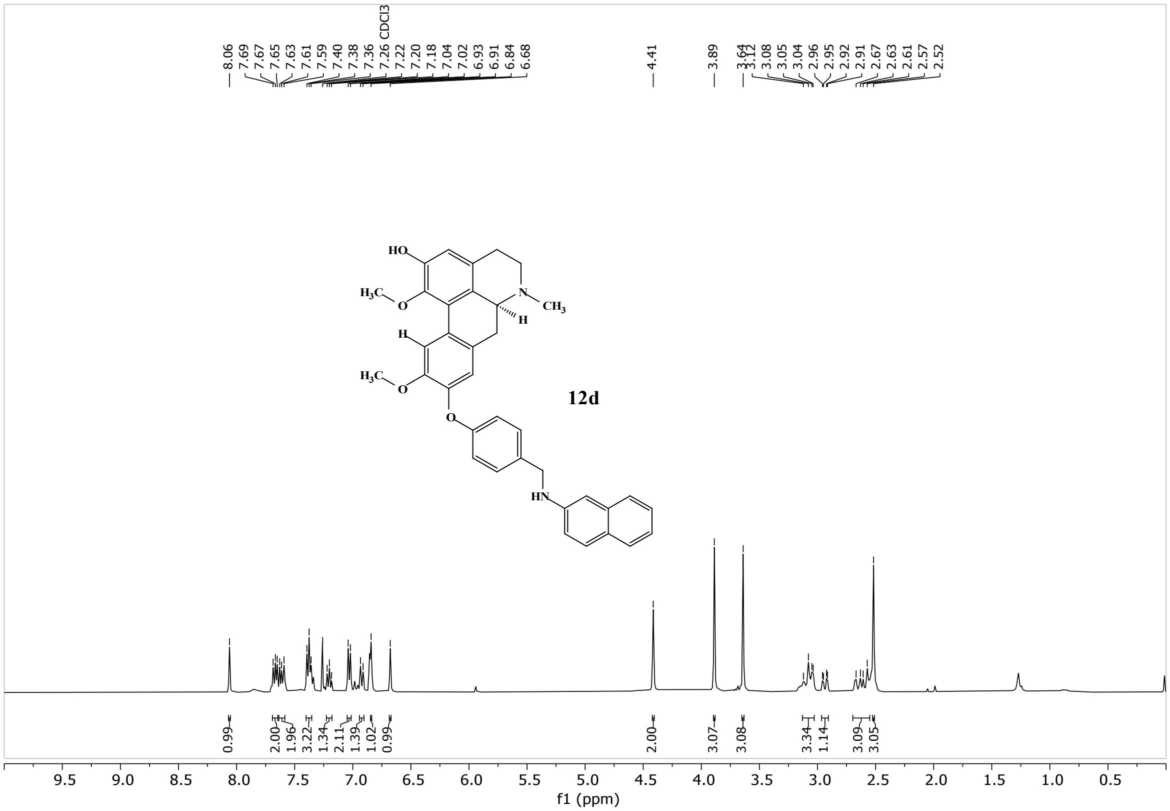

Figure S143. ^{1}H-NMR spectrum of **12d** ($CDCl_3$, 400 MHz).

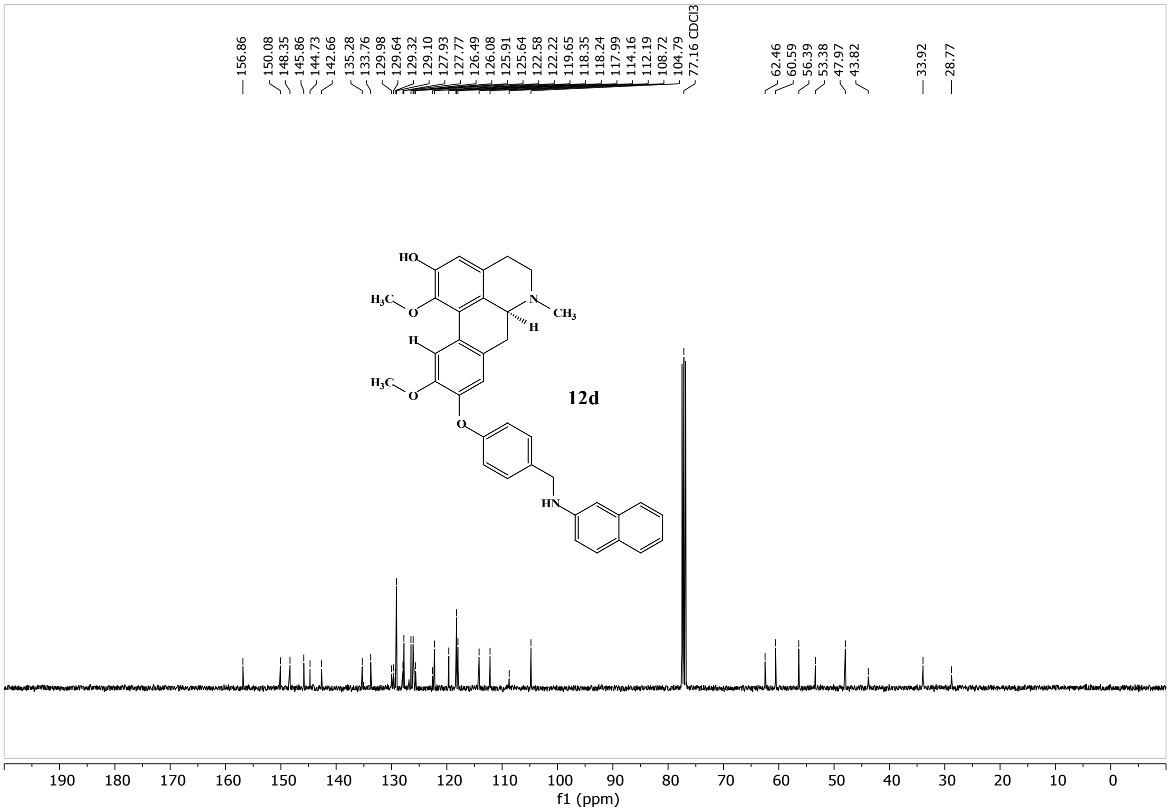

Figure S144. ^{13}C-NMR spectrum of **12d** ($CDCl_3$, 101 MHz).

4.- Cytotoxic Assay

Cytotoxicity assays for the boldine analog were quantified using the resazurin method, a dye to measure redox reactions occurring in the mitochondria of live cells. The cells were treated by tested compounds (in triplicate, six different doses) for 72 h. After this period, a resazurin (Sigma Aldrich St. Louis, MO, USA) solution was added, and the fluorescence of resorufin corresponding to the live cell quantity was measured after 4 h at 544 nm/590 nm (excitation/emission) using a Fluoroskan Ascent microplate reader (Labsystems Oy, Vantaa, Finland). The drug concentration lethal to 50% of the cells (GI_{50}) was calculated from the dose–response curves.

Table S1: Growth Inhibition on MV4-11 cancer cell line.

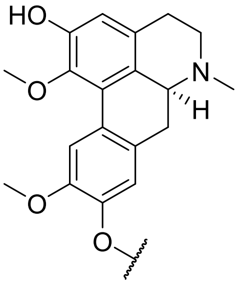

Compound	9-*O*-Substitution	GI_{50} µM
**6a**	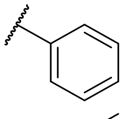	10.6 ± 9.8
**6b**	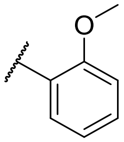	24.4 ± 2.0
**6c**	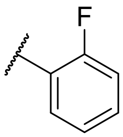	17.2 ± 0.5
**6d**	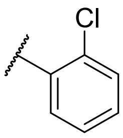	15.6 ± 0.8
**6e**	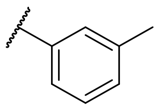	18.8 ± 0.2

**6f**	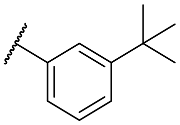	14.9 ± 1.9
**6g**	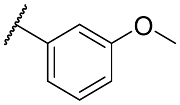	17.2 ± 2.0
**6h**	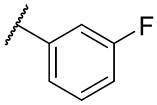	16.2 ± 1.9
**6i**	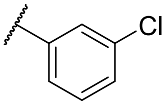	16.2 ± 0.9
**6j**	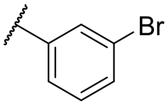	16.2 ± 4.0
**6k**	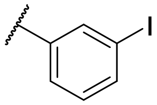	13.6 ± 3.2
**6l**	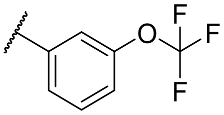	16.3 ± 0.0
**6m**	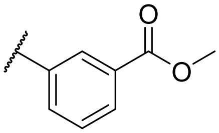	>25.0
**6n**	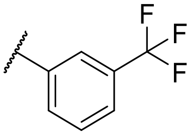	17.7 ± 1.6
**6o**	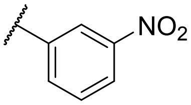	16.5 ± 0.4
**6p**	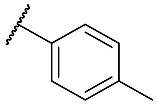	15.1 ± 0.8
**6q**	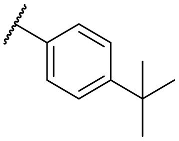	15.8 ± 0.1
**6r**	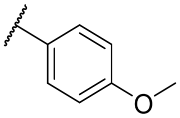	17.7 ± 0.3
**6s**	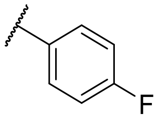	16.0 ± 0.0

**6t**	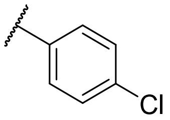	14.2 ± 0.6
**6u**	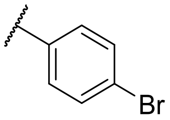	17.4 ± 0.1
**6v**	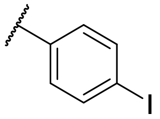	15.3 ± 0.8
**6w**	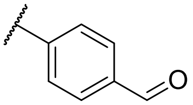	16.5 ± 2.0
**6x**	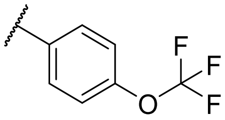	13.5 ± 0.7
**6y**	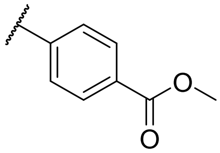	15.3 ± 0.7
**6z**	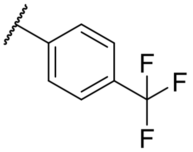	12.1 ± 3.0
**6aa**	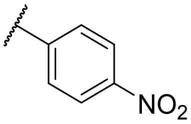	25.2 ± 0.9
**6ab**	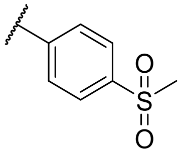	20.6 ± 2.3
**6ac**	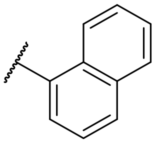	19.1 ± 2.7
**6ad**	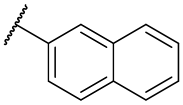	20.7 ± 0.6
**Boldine**	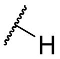	>25.0
**Sorafenib**		0.005 ± 0.002
**Cisplatin**		0.75 ± 0.21
